# A systematic assessment of the association between frequently prescribed medicines and the risk of common cancers: a series of nested case-control studies

**DOI:** 10.1186/s12916-020-01891-5

**Published:** 2021-01-26

**Authors:** R. D. McDowell, C. Hughes, P. Murchie, C. Cardwell

**Affiliations:** 1 Centre for Public Health, Queen’s University, Grosvenor Rd., Belfast, Co. Antrim BT12 6BA UK; 2 School of Pharmacy, Queen’s University, Lisburn Rd, Belfast, Co. Antrim BT9 7BL UK; 3 Division of Applied Health Sciences Section, Section of Academic Primary Care, Foresterhill, Aberdeen, AB24 2ZD UK

**Keywords:** Screening study, Cancer risk, Pharmacoepidemiology

## Abstract

**Background:**

Studies systematically screening medications have successfully identified prescription medicines associated with cancer risk. However, adjustment for confounding factors in these studies has been limited. We therefore investigated the association between frequently prescribed medicines and the risk of common cancers adjusting for a range of confounders.

**Methods:**

A series of nested case-control studies were undertaken using the Primary Care Clinical Informatics Unit Research (PCCIUR) database containing general practice (GP) records from Scotland. Cancer cases at 22 cancer sites, diagnosed between 1999 and 2011, were identified from GP records and matched with up to five controls (based on age, gender, GP practice and date of registration). Odds ratios (OR) and 95% confidence intervals (CI) comparing any versus no prescriptions for each of the most commonly prescribed medicines, identified from prescription records, were calculated using conditional logistic regression, adjusting for comorbidities. Additional analyses adjusted for smoking use. An association was considered a signal based upon the magnitude of its adjusted OR, *p*-value and evidence of an exposure-response relationship. Supplementary analyses were undertaken comparing 6 or more prescriptions versus less than 6 for each medicine.

**Results:**

Overall, 62,109 cases and 276,580 controls were included in the analyses and a total of 5622 medication-cancer associations were studied across the 22 cancer sites. After adjusting for comorbidities 2060 medicine-cancer associations for any prescription had adjusted ORs greater than 1.25 (or less than 0.8), 214 had a corresponding *p*-value less than or equal to 0.01 and 118 had evidence of an exposure-dose relationship hence meeting the criteria for a signal. Seventy-seven signals were identified after additionally adjusting for smoking. Based upon an exposure of 6 or more prescriptions, there were 118 signals after adjusting for comorbidities and 82 after additionally adjusting for smoking.

**Conclusions:**

In this study a number of novel associations between medicine and cancer were identified which require further clinical and epidemiological investigation. The majority of medicines were not associated with an altered cancer risk and many identified signals reflected known associations between medicine and cancer.

**Supplementary Information:**

The online version contains supplementary material available at 10.1186/s12916-020-01891-5.

## Background

Cancer remains a leading cause of disease burden and death [1, 2]. In 2018, there were an estimated 18.1 million new cases of cancer worldwide and 9.5 million deaths, with the yearly incidence estimated to increase to 29.5 million by 2040 [3]. Despite continuing advances in medical research, survival remains low for a range of cancers [4], highlighting the need to avoid precipitating factors.

Certain medicines may possess unintended carcinogenic or chemoprotective properties. For instance, hormone replacement therapy (HRT) has been shown to increase breast cancer risk [5], and aspirin has traditionally been considered to reduce colorectal cancer risk [6] (although recent studies have questioned the perceived protective effect associated with aspirin on cancer risk [7, 8]). Clinical trials conducted during the development of new medications are unlikely to identify medications that alter cancer risk due to the relatively small number of patients exposed to the medication and the generally short duration of follow-up [9]. Spontaneous reporting systems (such as the United Kingdom (UK) Yellow Card Scheme) may also identify medications which cause cancer, but their ability is limited by the long induction time of many cancers and by design they will not identify medicines with a potential chemoprotective effect. Consequently, pharmacoepidemiology, the study of the use and effects of drugs in large numbers of people [10], has proved a valuable tool in the identification of medications which can cause or reduce the risk of cancer.

In traditional pharmacoepidemiology studies, investigators have identified a clinical mechanism whereby a medication may increase cancer risk a priori and then investigated that specific association. However, it is difficult to predict possible carcinogenic mechanisms in advance, particularly as the number of licensed medicines is growing; between 2013 and 2018, the Food and Drug Association licensed, on average, between 40 and 50 new drugs per year [11]. Consequently, studies have been conducted systematically investigating, or screening, large numbers of medicines in relation to cancer risk as a means of complimenting traditional pharmacoepidemiologic practice. Screening studies have the potential to identify medicine-cancer associations which require more detailed study, as well as highlighting associations which are not widely recognised.

To date, studies systematically screening medicines in relation to cancer risk have been conducted among subscribers to Kaiser Permanente healthcare plans in the USA [12–16] as well as more recent population-based studies in Denmark [17], Sweden [18] and Norway [19]. However, these screening studies have had limitations, such as a relatively short follow-up period or a limited number of cancer sites studied [18], and none have controlled for site-specific risk factors or smoking (an accepted risk factor for many cancers [20]). It has been suggested that confounding by smoking is a possible explanation for the positive association observed in some screening studies between certain medicines and cancer, such as respiratory/allergy medicines with lung cancer [16, 21] .Therefore, we systemically assessed the associations between commonly prescribed medicines and cancer risk, adjusting for a wide range of confounders including smoking, using a series of nested case-control studies.

## Methods

### Data source

Data for this study was obtained from Primary Care Clinical Information Unit Research (PCCIUR) [22], a high-quality population-based database of over two million patients registered at 393 general practices in Scotland between 1993 and 2011. PCCIUR data contains up to 20 years of demographic, clinical and diagnostic information and has been widely used in epidemiological research [23–26].

### Study design

A series of retrospective nested case-control studies were conducted using PCCIUR data. Cases were identified based upon a new diagnosis of primary cancer (including only the twenty-two most common cancers in Scotland). Cases were excluded if they had a previous cancer, excluding non-melanoma skin cancer, or they were diagnosed with multiple primary cancers on the date of their first cancer diagnosis (due to uncertainty about the primary cancer and the potential for coding errors). Up to five controls were matched to cases on practice, year of birth (plus or minus five years), gender and year of registration (in categories). The index date within each matched set was defined as the diagnosis date of cancer in the case. Controls were required to be alive and free from cancer (with the exception of non-melanoma skin cancer) on the index date. Both cases and controls were required to have at least 3 years of follow-up data and remain registered with the same general practice over the follow-up period.

Within each matched set, the exposure period, i.e. the period of time over which medicine use was determined, started on either 1 January 1993 (as prescriptions before this time were less likely to be electronically recorded) or the most recent GP registration date if this occurred after January 1993. This ensured that all members within each matched set had the same exposure period. The exposure period ended 1 year before the index date, to reduce the risk of reverse causality and exclude medications that are unlikely to have had sufficient time to cause the cancer [27, 28].

### Classification and definition of medicine

Prescription entries were extracted from PCCIUR, and over 99% were converted to a generic name, formulation and route of administration. Foods, nutritional supplements, homoeopathic items and emollients (which are pharmaceutically inert) were excluded from the analyses. Single-agent medicines were grouped together if the active substance was the same and patient indications were similar (e.g. hydrocortisone). A distinction was made between low-dose aspirin (defined as 75 mg or less in the UK [29]) and high dose-aspirin (over 75 mg); low-dose aspirin is not usually considered a non-steroidal anti-inflammatory drug (NSAID) in the UK [30]. This distinction between low and high dose aspirin can be noted in observational studies [31] (including analyses examining associations between aspirin and cancer risk [32]) and clinical trials [33]. Combination drugs of two or more medicines with different pharmaceutical effects were split into their component parts and considered as two or more separate medicines. Where combination drugs comprised active and inert medicines or agents not affecting physiology, or active medicines combined with other substances used to enhance the effect of the active ingredient (e.g. clavulanic acid which enhances the effect of penicillin in co-amoxiclav), only the active medicines were considered for association. All forms of insulin were grouped together as insulin. Systemic formulations of medicines (oral and parenteral formulations, together with all topical items applied for a systemic effect) and local formulations (all topical items applied for a local effect) were analysed separately. These groupings of medications were reviewed independently by a GP and pharmacist.

### Covariates

The following comorbidities, based upon published read codes for the Charlson Comorbidity Index (CCI) [34], were identified prior to or during the exposure period: diabetes, myocardial infarction, coronary heart disease, heart failure, peripheral vascular disease, dementia, cerebrovascular disease, chronic obstructive pulmonary disease, osteoporosis, rheumatological disease, renal disease, liver disease, irritable bowel disease, human immunodeficiency viruses (HIV) and hemiplegia/paraplegia. Additional risk factors relevant to specific cancers were identified from the literature. For example, studies have shown a reduced risk of prostate cancer in patients with Parkinson’s disease [35], an increased risk of lung cancer in patients with tuberculosis [36] and a 30% increased relative risk of kidney cancer among women undergoing a hysterectomy [37]. These additional risk factors were independently reviewed by a pharmacist and a GP to determine which could be considered confounders between medicine use and cancer risk and were extracted from PCCIUR where they were recorded. These are listed in Table 2 as potential site-specific confounders. Smoking status (non-smoker, current smoker, former smoker) and alcohol consumption (non-drinker, light or moderate drinker, heavy drinker) were determined from the most recent smoking or alcohol record prior to or during the exposure period.

### Statistical analysis

Analyses were conducted for each of the 250 medicines most commonly prescribed (three or more times) within the exposure period in the matched controls for each cancer site. Where a number of medicines were equally prevalent at the 250 rank, all were studied. Descriptive statistics were used to summarise the cases and controls. Conditional logistic regression was used to calculate odds ratios (OR) and 95% confidence intervals (CI) for the association between any prescription and each cancer. The matched design accounted for age (± 5 years), GP practice, gender and year of registration, and the adjusted model contained age (in years, allowing for the fact that patients were matched in age bands rather by calendar year) and comorbidities. Analyses were repeated additionally adjusting for smoking, and were restricted to the 77.9% of patients (*n* = 263,615) with a smoking record before the end of the exposure period. These supplementary analyses additionally adjusted for smoking status rather than both smoking and alcohol use as smoking status was recorded for a greater number of patients than alcohol use (67.5%, *n* = 228,425) and has been shown to be well recorded in primary care [38]. Body mass index (BMI) could not be controlled for as it was only recorded for one-quarter of PCCIUR patients. Exposure-response analyses were conducted calculating ORs for low and high use compared with none, with low/high use based upon numbers of prescriptions equal to or below/above the median (among the control patients who were users), respectively. To illustrate, for a medicine associated with an increased risk of cancer and where the median number of items among users without cancer was 4, we required the odds ratio, comparing use of 1–4 items of medicine to none, to be less than the odds ratio comparing the use of 5 or more items to none. For a medicine associated with a decreased cancer risk and with the same median number of items, we required the odds ratio comparing use of 1–4 items of medicine to none to be larger than the odds ratio comparing use of 5 or more items to none. This approach to quantifying exposure-response relationships is found in other screening studies [16].

Supplementary analyses were undertaken with an exposure of six or more prescriptions (v less than six items), using the exposure-response analysis based on the median.

### Definition of signal

For each set of analyses undertaken, the following criteria were used to identify signals, i.e. medicines deemed worthy of further consideration, as no accepted definitions of a signal exist [16, 17, 19, 21]: (step 1) an adjusted OR for the association between the medicine and cancer risk greater than 1.25 (or less than 0.80); (step 2) an OR in 1 of statistical significance at the 1% level; (step 3) evidence of an exposure-response such that the OR comparing low to none was less extreme than the OR comparing high to none.

### Sensitivity analyses

A number of sensitivity analyses were undertaken as follows: (1) the period of time before the index date during which prescriptions were not counted was increased from 1 year to 2 years to reduce the risk of reverse causation; (2) matched sets where the case had a new primary cancer diagnosis in a different cancer site within 12 months of the index date were excluded to allow for possible misclassification of the original cancer site; (3) adjustments were made for comorbidities, smoking and alcohol status for the 221,570 (65.4%) patients with available data. Additionally, analyses adjusting for comorbidities and smoking were rerun using multiple imputation with chained equations (MICE) techniques to impute smoking status. This is a simulation-based method appropriate for handling missing data when it is assumed that such values are missing at random or missing completely at random. Ordered logit models were used with age, gender, deprivation and comorbidities for the imputations, stratified by case-control status, and used 25 imputations.

### Medication-wide association study (MWAS) plots

Results from the primary analyses, estimating associations between medicine use and cancer, adjusting for comorbidities, were depicted graphically using medication-wide association study (MWAS) plots. MWAS plots display the *p* values for the associations against the medicines grouped by British National Formulary (BNF) chapter [39].

All analyses were undertaken using Stata 15 [40].

## Results

### Descriptive statistics

The study included 62,019 cases (29,653 males and 32,366 females) and 276,580 matched controls. The most common cancers were breast (12,269), lung (9409), colorectal (8674) and prostate (7471). Overall, 53,533 cases (86.3%) had at least four matched controls. The median exposure period was 8.1 years in cases and controls (inter-quartile range 5.5 to 11.0). The overall characteristics of cases and controls are shown in Table 1.

**Table 1:** Characteristics of cases and controls

Variable	Category	Cases ***n*** (%)	Controls ***n*** (%)
		***n*** = 62,019	***n*** = 276,580
**Length of exposure period (years): median (IQR)**		8.1 (5.5, 11.0)	8.1 (5.5, 11.0)
**Year of diagnosis/index date: median (IQR)**		2005 (2002, 2007)	2005 (2002, 2007)
**Age at diagnosis/index date (years)**	**0–39**	1983 (3.2%)	12,004 (4.3%)
	**40–59**	15,087 (24.3%)	86,385 (31.2%)
	**60–79**	35,294 (56.9%)	143,628 (51.9%)
	**80 +**	9655 (15.6%)	34,563 (12.5%)
**Deprivation quintile**	**1 (least deprived)**	3911 (6.3%)	17,535 (6.3%)
	**2**	12,427 (20.0%)	55,723 (20.1%)
	**3**	5811 (9.4%)	26,166 (9.5%)
	**4**	15,518 (25.0%)	68,902 (24.9%)
	**5 (most deprived)**	16,558 (26.7%)	73,319 (26.5%)
	**Missing**	117 (0.0%)	529 (0.19%)
**Gender**	**Male**	29,653 (47.8%)	128,988 (46.6%)
	**Female**	32,366 (52.2%)	147,592 (53.4%)
Smoking status*	**Never smoked**	20,982 (33.8%)	105,499 (38.1%)
	**Ex-smoker**	12,792 (20.6%)	50,731 (18.3%)
	**Current smoker**	15,877 (25.6%)	57,734 (20.9%)
	**Missing**	12,368 (19.9%)	62,616 (22.6%)
Alcohol consumption*	**Non-drinker**	9451 (15.2%)	40,117 (14.5%)
	**Light/moderate**	30,759 (49.6%)	135,074 (48.8%)
	**Heavy drinker**	2799 (4.5%)	10,225 (3.7%)
	**Missing**	19,010 (30.7%)	91,164 (33.0%)
**Comorbidities diagnosed prior to or during the exposure period**			
**Diabetes**		5428 (8.8%)	20,750 (7.5%)
**Myocardial infarction**		4001 (6.5%)	15,045 (5.6%)
**Coronary heart disease**		9984 (16.1%)	37,739 (13.6%)
**Heart failure**		2155 (3.5%)	7625 (2.8%)
**Peripheral vascular disease**		3225 (5.2%)	10,475 (3.8%)
**Dementia**		516 (0.8%)	3353 (1.2%)
**Cerebrovascular disease**		4372 (7.2%)	16,875 (6.1%)
**Chronic obstructive pulmonary disease**		6498 (10.4%)	19,155 (6.9%)
**Osteoporosis**		1866 (3.0%)	7421 (2.7%)
**Rheumatology**		1578 (2.5%)	6268 (2.3%)
**Renal disease**		2513 (4.1%)	8719 (3.2%)
**Liver disease**		683 (1.1%)	1973 (0.7%)
**Irritable bowel disease**		3533 (5.7%)	15,360 (5.6%)
**Human immunodeficiency virus**		17 (0.0%)	41 (0.0%)
**Hemiplegia/paraplegia**		307 (0.5%)	1324 (0.5%)

*Abbreviations*: *IQR* inter-quartile range

*Most recent record in patient’s clinical history

### Signals

In total, 5622 medicine-cancer associations were investigated across the 22 cancer sites. Of these, 2060 had a comorbidity-adjusted OR for any prescription greater than 1.25 (or less than 0.80), 214 were statistically significant at the 1% level and 118 had an exposure-response relationship with cancer risk. Repeating these analyses additionally adjusting for smoking, 2139 medicine-cancer associations had an OR greater than 1.25 (or less than 0.80), of which 143 were statistically significant at the 1% level and 77 had an exposure-response relationship with cancer. There were 142 unique medicine-cancer signals.

For the supplementary analyses, 2714 medicine-cancer associations had a comorbidity-adjusted OR for six or more medicines greater than 1.25 (or less than 0.80), 138 were statistically significant at the 1% level and 118 had an exposure-response relationship with cancer risk. Repeating these analyses additionally adjusting for smoking, 2926 medicine-cancer associations had an OR greater than 1.25 (or less than 0.80), of which 89 were statistically significant at the 1% level and 82 had an exposure-response relationship with cancer. There were 147 unique medicine-cancer signals.

Across all analyses, there were 231 unique medicine-cancer signals, of which 22 were found in every analysis; 89 signals were only identified with an exposure of at least six prescriptions. One hundred and eighty-six signals were identified after adjusting for comorbidities, of which less than half (85) met the signal criteria after additionally adjusting for smoking. A further 45 signals were only identified in the analyses which controlled for both smoking and comorbidities; 169 signals were associated with an increased cancer risk and 62 with a lower risk of cancer.

The number of signals identified by each criterion for each analysis is listed in Table 2. The signals found are summarised in Table 3, Table 4, Table 5, and Table 6, with full details of the signals given in Additional file 1: Tables S1 to S4. Additional file 2 details potentially relevant clinical or epidemiological references for these signals.

**Table 2:** Overview of signals obtained from screening analyses

Cancer	Main read codes	Potential site-specific confounders	No. patients		Exposure: any prescription						Exposure: ≥ 6 items (***v*** < 6 items)					
					Adjusted for comorbidities^**†**^			Adjusted for comorbidities^**†**^ and smoking			Adjusted for comorbidities^**†**^			Adjusted for comorbidities^**†**^ and smoking		
					No. signals			No. signals			No. signals			No. signals		
			Cases	Controls	Step 1	Step 2	Step 3	Step 1	Step 2	Step 3	Step 1	Step 2	Step 3	Step 1	Step 2	Step 3
**Breast**^**§**^	**B34**	**Oophorectomy, hysterectomy**	12,269	57,064	16	7	4	15	7	4	43	9	7	53	7	6
**Lung**	**B22**	**Pneumonia, tuberculosis**	9489	41,472	38	26	19	28	14	9	67	22	18	67	14	13
**Colorectal**	**B13**	**Gallstones, acromegaly**	8674	37,935	36	13	7	35	12	3	60	12	12	72	9	8
**Prostate**^**§§**^	**B46**	**Vasectomy, prostatitis, Parkinson’s**	7471	32,087	42	11	5	42	6	5	75	15	13	89	9	9
**Bladder**	**B49**	**Kidney transplant**	3229	13,907	61	15	5	70	13	8	89	6	5	110	7	7
**Malignant melanoma**	**B32**	**Parkinson’s, transplant**	2460	11,332	71	8	5	71	3	2	120	3	2	135	0	0
**Oesophagus**	**B10**	**HPV, achalasia**	2402	10,467	76	16	12	81	11	9	126	8	8	146	7	7
**Non-Hodgkin’s lymphoma**	**B60,B62, B6y,** **B6z**	**Helicobacter pylori, transplant, anaemia, coeliac disease, hepatitis B/C, STDs, breast implant**	2077	9395	92	17	11	95	9	4	132	7	6	137	3	3
**Leukaemia**	**B64-B69**	**Anaemia, transplant, neurofibromatosis**	1998	8839	81	6	3	88	4	1	131	4	4	143	6	5
**Oral (inc. head, neck, nasal)**	**B0, B20, B550**	**HPV, transplant**	1467	6717	113	14	8	112	9	4	152	5	5	148	5	4
**Stomach**	**B11**	**Transplant, anaemia, helicobacter pylori, low stomach acid**	1433	6192	84	7	4	91	6	3	129	2	2	143	2	2
**Ovary**^**§**^	**B44**	**Endometriosis, hysterectomy, sterilisation**	1417	6514	93	8	4	100	5	2	126	5	5	136	1	1
**Kidney**	**B4A**	**Tuberous sclerosis, hypertension, hysterectomy, kidney stone, polycystic kidney disease**	1353	6070	90	9	3	94	3	2	137	5	3	145	3	3
**Pancreas**	**B17**	**Peptic ulcer, helicobacter pylori, hepatitis B/C, gallstones, metabolic syndrome**	1069	4729	105	8	2	104	5	1	144	5	4	156	5	4
**Cervix and other gynae**^**§**^	**B454., B450., B41, B441., B420.**	**HPV, STDs, transplant, intra-uterine device**	1039	4844	107	7	3	115	5	1	155	1	1	173	0	0
**Uterus**^**§**^	**B40, B43**	**Polycystic ovarian syndrome, hyperinsulinemia, use of IUD**	1010	4700	103	14	10	103	4	3	139	14	10	149	5	5
**Larynx**	**B21**	**HPV, transplant**	741	3321	140	12	6	147	5	3	158	4	4	174	0	0
**Brain and CNS**	**B51,B52**	**Neurofibromatosis, tuberous sclerosis**	732	3396	121	2	1	125	2	1	151	2	1	154	1	0
**Myeloma**	**B63**	**MGUS, transplant, anaemia,** **ankylosing spondylitis**	541	2393	137	5	2	142	4	3	142	1	1	157	1	1
**Liver**	**B15**	**Hepatitis B/C, haemochromatosis, gallstones**	528	2334	133	1	0	140	3	0	156	4	3	148	1	1
**Thyroid**	**B53**	**Acromegaly**	341	1614	172	5	2	174	10	6	139	2	2	140	1	1
**Anal**	**B14**	**HPV, transplant, anal fissures, STDs**	279	1258	149	3	2	167	3	3	143	2	2	151	2	2

*Abbreviations*: *HPV* human papilloma virus, *STDs* sexually transmitted diseases, *MGUS* monoclonal gammopathy of undetermined significance, *IUD* intra-uterine device, *Step 1* odds ratio ≥ 1.25 or ≤ 0.80, *Step 2 p* value ≤ 0.01, *Step 3* evidence of an exposure-response relationship, *CNS* cental nervous system

^†^Comorbidities include diabetes, myocardial infarction, coronary heart disease, heart failure, peripheral vascular disease, dementia, cerebrovascular disease, chronic obstructive pulmonary disease, osteoporosis, rheumatological disease, renal disease, liver disease, irritable bowel disease, human immunodeficiency viruses and hemiplegia/paraplegia, plus potential site-specific confounders

^§^Analyses restricted to females only

^§§^Analyses restricted to males only

**Table 3:** Signals associated with increased cancer risk (exposure: any prescription)

Cancer	Adjusted for comorbidities^**†**^		Adjusted for comorbidities^**†**^ and smoking	
**Breast**^**§**^	Estrogen-HRT (OR = 1.26) Metronidazole* (OR = 1.25)	Progestogen-HRT (OR = 1.28)	Bisoprolol (OR = 1.30) Metronidazole* (OR = 1.26)	Progestogen-HRT (OR = 1.25)
**Lung**	Amoxicillin (OR = 1.40) Azathioprine (OR = 1.56) Cimetidine (OR = 1.39) Clarithromycin (OR = 1.34) Codeine (OR = 1.31) Dihydrocodeine (OR = 1.29) Folic acid (OR = 1.49)^§^	Ipratropium* (OR = 1.58) Nitrazepam (OR = 1.35) Nystatin (OR = 1.27) Paracetamol (OR = 1.34) Salbutamol* (OR = 1.44) Salmeterol* (OR = 1.34) Tiotropium* (OR = 1.75)	Amoxicillin (OR = 1.29) Azathioprine (OR = 1.76) Cimetidine (OR = 1.35) Ipratropium * (OR = 1.43)	Nitrazepam (OR = 1.34) Salbutamol* (OR = 1.35) Salmeterol* (OR = 1.44) Tiotropium* (OR = 1.63)
**Colorectal**	Allopurinol (OR = 1.27)	Prednisolone* (OR = 1.31)	Allopurinol (OR = 1.26)	
**Prostate**^**§§**^	Cerivastatin (OR = 1.43)	Clioquinol* (OR = 1.30)	Alfuzosin (OR = 1.66)	Clioquinol* (OR = 1.34)
**Bladder**	Celecoxib (OR = 1.40) Dexamethasone* (OR = 1.30)	Nicotine (OR = 2.04) Trimethoprim (OR = 1.96)	Cefalexin (OR = 1.37) Ciprofloxacin (OR = 1.40) Nicotine (OR = 1.54) Phenoxymethylpenicillin (OR = 1.30)	Quinine (OR = 1.28) Ranitidine (OR = 1.26) Trimethoprim (OR = 1.92)
**Malignant melanoma**	Chloramphenicol* (OR = 1.28) Clopidogrel (OR = 1.60)	Flucloxacillin (OR = 1.28)	Clopidogrel (OR 1.63)	
**Oesophagus**	Alginic acid (OR = 1.40) Azathioprine (OR = 2.47) Cisapride (OR = 2.03) Gramicidin* (OR = 1.52) Lansoprazole (OR = 1.35) Nicotine (OR = 1.50)	Nystatin (OR = 1.58) Nystatin* (OR = 1.29) Omeprazole (OR = 1.39) Triamcinolone* (OR = 1.47) Vitamin B (OR = 1.36)	Alginic acid (OR = 1.38) Azathioprine (OR = 3.39) Ipratropium* (OR = 1.45) Lansoprazole (OR = 1.33)	Nystatin (OR = 1.65) Nystatin* (OR = 1.36) Omeprazole (OR = 1.29)
**Non-Hodgkin’s lymphoma**	Amoxicillin (OR = 1.34) Betamethasone* (OR = 1.37) Chlorhexidine* (OR = 1.67) Clarithromycin (OR = 1.33) Clobetasol* (OR = 1.73)	Erythromycin (OR = 1.26) Hydroxyzine (OR = 1.75) Methotrexate (OR = 2.93) Prednisolone (OR = 1.37) Prochlorperazine (OR = 1.30)	Betamethasone* (OR = 1.26) Clobetasol* (OR = 1.72)	Erythromycin (OR = 1.28)
**Leukaemia**	Allopurinol (OR = 1.57) Amoxicillin (OR = 1.29)	Trimethoprim (OR = 1.26)	Oxybutynin (OR = 1.93)	
**Oral (inc. head, neck, nasal)**	Codeine (OR = 1.29) Flucloxacillin (OR = 1.36) Folic acid (OR = 2.52)^§^	Nystatin (OR = 2.06) Vitamin B (OR = 2.75) Vitamin D (OR = 1.69)	Clobetasone* (OR = 1.41) Nystatin (OR = 1.92)	Vitamin B (OR = 2.33)
**Stomach**	Cimetidine (OR = 1.44) Clarithromycin (OR = 1.35)	Digoxin (OR = 1.49) Vitamin B (OR = 1.54)	Codeine (OR = 1.35) Nicotinates* (OR = 2.09)	Vitamin B (OR = 1.55)
**Ovary**^**§**^	Phenytoin (OR = 3.24)	Rabeprazole (OR = 2.18)	Phenytoin (OR = 3.86)	
**Kidney**	Amoxicillin (OR = 1.28) Hydrochlorothiazide (OR = 1.93)	Perindopril (OR = 1.63)	Hydrochlorothiazide (OR = 2.21)	Perindopril (OR = 1.76)
**Pancreas**	Betamethasone* (OR = 1.34)	Nicotine (OR = 1.94)	Amoxicillin (OR = 1.31)	
**Cervix and other gynae**^**§**^	Dihydrocodeine (OR = 1.39)	Ranitidine (OR = 1.37)	–	
**Uterus**^**§**^	Atenolol (OR = 1.39) Bendroflumethiazide (OR = 1.27) Bisoprolol (OR = 1.82) Doxazosin (OR = 1.68)	Enalapril (OR = 1.66) Ibuprofen* (OR = 1.53) Mefenamic acid (OR = 1.72) Tranexamic acid (OR = 1.87)	Chlortalidone (OR = 2.14)	Enalapril (OR = 2.00)
**Larynx**	Benzydamine* (OR = 2.33) Dextropropoxyphene (OR = 1.51)	Paracetamol (OR = 1.45) Vitamin B (OR = 2.40)	Dextropropoxyphene (OR = 1.54) Paracetamol (OR = 1.40)	Vitamin B (OR = 1.80)
**Brain and CNS**	Carbamazepine (OR = 2.20)		Zinc oxide* (OR = 1.97)	
**Myeloma**	Amoxicillin (OR = 1.53)	Ciprofloxacin (OR = 1.73)	Amoxicillin (OR = 1.45) Paracetamol (OR = 1.51)	Clioquinol* (OR = 2.52)
**Thyroid**	Gabapentin (OR = 5.13)	Levothyroxine (OR = 2.18)	Clarithromycin (OR = 2.10) Flucloxacillin (OR = 1.70) Folic acid (OR = 2.77)^§^	Gabapentin (OR = 5.84) Levothyroxine (OR = 2.21) Mefenamic acid (OR = 2.30)
**Anal**	Clonidine (OR = 3.87)	Doxazosin (OR = 2.48)	Clonidine (OR = 4.49) Doxazosin (OR = 2.76)	Theophylline (OR = 9.08)

*Abbreviations*: *OR* odds ratio, *HRT* hormone replacement therapy, *CNS* central nervous system

^†^Comorbidities include diabetes, myocardial infarction, coronary heart disease, heart failure, peripheral vascular disease, dementia, cerebrovascular disease, chronic obstructive pulmonary disease, osteoporosis, rheumatological disease, renal disease, liver disease, irritable bowel disease, human immunodeficiency viruses and hemiplegia/paraplegia, plus potential site-specific confounders

*Medicines applied for local effect, all other medicines systemic

^§^Analyses restricted to females only

^§§^Analyses restricted to males only

**Table 4:** Signals associated with decreased cancer risk (exposure: any prescription)

Cancer	Adjusted for comorbidities^**†**^	Adjusted for comorbidities^**†**^ and smoking
**Breast**^**§**^	Trazodone (OR = 0.79)	Trazodone (OR = 0.74)
**Lung**	Fluocinolone* (OR = 0.67) Levodopa (OR = 0.50) Nitrofurantoin (OR = 0.74) Progestogen-contraceptive (OR = 0.62)^§^ Risperidone (OR = 0.55)	Fluocinolone* (OR = 0.66)
**Colorectal**	Levodopa (OR = 0.60) Lofepramine (OR = 0.71) Meloxicam (OR = 0.76) Metoclopramide (OR = 0.78) Nitrofurantoin (OR = 0.71)	Metoclopramide (OR = 0.77) Nitrofurantoin (OR = 0.66)
**Prostate**^**§§**^	Levodopa (OR = 0.47) Risperidone (OR = 0.37) Senna (OR = 0.76)	Levodopa (OR = 0.44) Risperidone (OR = 0.38) Senna (OR = 0.77)
**Bladder**	Iron (OR = 0.77)	Sotalol (OR = 0.19)
**Malignant melanoma**	Dipyridamole (OR = 0.42) Gliclazide (OR = 0.51)	Gliclazide (OR = 0.49)
**Oesophagus**	Estrogen-HRT (OR = 0.59)^§^	Diazepam (OR = 0.73) Estrogen-HRT (OR = 0.61)^§^
**Non-Hodgkin’s lymphoma**	Progestogen-contraceptive (OR = 0.56)^§^	Progestogen-contraceptive (OR = 0.49)^§^
**Oral (inc. head, neck, nasal)**	Doxycycline (OR = 0.45) Ispaghula (OR = 0.63)	Doxycycline (OR = 0.43)
**Ovary**^**§**^	Loratadine (OR = 0.64) Progestogen-contraceptive (OR = 0.55)	Progestogen-contraceptive (OR = 0.56)
**Cervix and other gynae**^**§**^	Terbinafine (OR = 0.18)	Terbinafine (OR = 0.20)
**Uterus**^**§**^	Estrogen-HRT (OR = 0.73) Progestogen-contraceptive (OR = 0.37)	Progestogen-contraceptive (OR = 0.37)
**Larynx**	Doxazosin (OR = 0.36) Terbinafine (OR = 0.32)	–

*Abbreviations*: *OR* odds ratio, *HRT* hormone replacement therapy

^†^Comorbidities include diabetes, myocardial infarction, coronary heart disease, heart failure, peripheral vascular disease, dementia, cerebrovascular disease, chronic obstructive pulmonary disease, osteoporosis, rheumatological disease, renal disease, liver disease, irritable bowel disease, human immunodeficiency viruses and hemiplegia/paraplegia, plus potential site-specific confounders

*Medicines applied for local effect, all other medicines systemic

^§^Analyses restricted to females only

^§§^Analyses restricted to males only

**Table 5:** Signals associated with increased cancer risk (exposure: ≥ 6 prescriptions)

Cancer	Adjusted for comorbidities^**†**^		Adjusted for comorbidities^**†**^ and smoking	
**Breast**^**§**^	Estrogen-HRT (OR = 1.36) Metronidazole* (OR = 2.21)	Progestogen-HRT (OR = 1.50)	Estrogen-HRT (OR = 1.32) Metronidazole* (OR = 2.36)	Progestogen-HRT (OR = 1.48)
**Lung**	Amitriptyline (OR = 1.27) Amoxicillin (OR = 1.44) Azathioprine (OR = 1.88) Cimetidine (OR = 1.46) Diazepam (OR = 1.29) Dihydrocodeine (OR = 1.40) Folic acid (OR = 1.50)^§^ Ipratropium* (OR = 1.63)	Nitrazepam (OR = 1.49) Quinine (OR = 1.25) Salbutamol* (OR = 1.41) Salmeterol* (OR = 1.37) Temazepam (OR = 1.33) Tiotropium* (OR = 1.64) Vitamin D* (OR = 1.45)	Amitriptyline (OR = 1.25) Amoxicillin (OR = 1.36) Azathioprine (OR = 2.30) Cimetidine (OR = 1.37) Dihydrocodeine (OR = 1.28) Fluticasone* (OR = 1.41) Ipratropium* (OR = 1.56)	Nitrazepam (OR = 1.58) Phenoxymethylpenicillin (OR = 2.89) Quinine (OR = 1.31) Salbutamol* (OR = 1.36) Salmeterol* (OR = 1.51) Tiotropium* (OR = 1.52)
**Colorectal**	Allopurinol (OR = 1.35) Aminophylline (OR = 1.93) Bisacodyl (OR = 1.79) Dipyridamole (OR = 1.45)	Mesalazine (OR = 1.65) Perindopril (OR = 1.31) Phenytoin (OR = 1.58)	Allopurinol (OR = 1.33) Aminophylline (OR = 1.89) Dipyridamole (OR = 1.44) Tiotropium* (OR = 1.49)	
**Prostate**^**§§**^	Cerivastatin (OR = 1.59)	Clioquinol* (OR = 2.06)	Alfuzosin (OR = 1.63) Atorvastatin (OR = 1.25)	Clioquinol* (OR = 2.09)
**Bladder**	Amoxicillin (OR = 1.39) Nicotine (OR = 2.59)	Trimethoprim (OR = 1.80)	Amoxicillin (OR = 1.53) Cefalexin (OR = 1.99) Celecoxib (OR = 1.78)	Ranitidine (OR = 1.28) Trimethoprim (OR = 2.04)
**Malignant melanoma**	Clopidogrel (OR = 1.86)		–	
**Oesophagus**	Alginic acid (OR = 1.52) Beclometasone* (OR = 1.37) Cisapride (OR = 2.89) Lansoprazole (OR = 1.67)	Omeprazole (OR = 1.45) Risedronate sodium (OR = 2.54) Vitamin B (OR = 1.54) Warfarin (OR = 1.43)	Alginic acid (OR = 1.53) azathioprine (OR = 4.15) Lansoprazole (OR = 1.59)	Omeprazole (OR = 1.40) Prednisolone (OR = 1.68) Risedronate sodium (OR = 2.69)
**Non-Hodgkin’s** **lymphoma**	Amoxicillin (OR = 1.46) Azathioprine (OR = 2.58) Gabapentin (OR = 2.86)	Methotrexate (OR = 3.41) Phenoxymethylpenicillin (OR = 3.77)	Amoxicillin (OR = 1.50)	Gabapentin (OR = 3.41)
**Leukaemia**	Allopurinol (OR = 1.68) Oxybutynin (OR = 2.35)	Oxytetracycline (OR = 2.67) Paracetamol (OR = 1.26)	Metoclopramide (OR = 2.61) Oxybutynin (OR = 2.69)	Oxytetracycline (OR = 3.01) Paracetamol (OR = 1.34)
**Oral (inc. head, neck, nasal)**	Betamethasone* (OR = 1.69) Folic acid (OR = 3.79) ^§^ Vitamin A (OR = 3.84)	Vitamin B (OR = 3.09) Vitamin C (OR = 4.43)	Hydrochlorothiazide (OR = 2.56) Hydrocortisone* (OR = 1.66)	Vitamin B (OR = 2.55) Vitamin C (OR = 3.63)
**Stomach**	Cimetidine (OR = 1.52)		Chlorphenamine (OR = 4.72)	
**Ovary**^**§**^	Cyclopenthiazide (OR = 3.31) Phenytoin (OR = 3.87)	Vitamin D* (OR = 2.66)	Phenytoin (OR = 4.31)	
**Kidney**	Hydrochlorothiazide (OR = 2.26) Nystatin* (OR = 2.25)	Perindopril (OR = 1.87)	Hydrochlorothiazide (OR = 2.61)	Perindopril (OR = 1.94)
**Pancreas**	Clopidogrel (OR = 2.14) Hydrochlorothiazide (OR = 1.84)	Metoclopramide (OR = 3.08) Nicotine (OR = 3.61)	Clopidogrel (OR = 2.37) Lorazepam (OR = 3.59)	Methotrexate (OR = 4.39) Quinine (OR = 1.78)
**Cervix & other gynae**^**§**^	Cimetidine (OR = 1.87)			
**Uterus**^**§**^	Atenolol (OR = 1.40) Bendroflumethiazide (OR = 1.33) Bismuth* (OR = 6.56) Dextropropoxyphene (OR = 1.43) Doxazosin (OR = 2.21)	Enalapril (OR = 1.78) Losartan (OR = 2.00) Peru balsam* (OR = 6.56) Salicylic acid* (OR = 4.31)	Bendroflumethiazide (OR = 1.32) Doxazosin (OR = 1.88)	Enalapril (OR = 2.35) Loperamide (OR = 2.88) Salicylic acid* (OR = 6.85)
**Larynx**	Cimetidine (OR = 2.12)	Vitamin B (OR = 2.20)	–	
**Brain and CNS**	Carbamazepine (OR = 3.02)		–	
**Myeloma**	Mometasone* (OR = 3.45)		Chlortalidone (OR = 3.06)	
**Liver**	Furosemide (OR = 1.98) Mometasone* (OR = 3.82)	Pravastatin (OR = 2.26)	Furosemide (OR = 2.48)	
**Thyroid**	Levothyroxine (OR = 2.30)	Progestogen-HRT (OR = 2.52)^§^	Progestogen-HRT (OR = 2.55) ^§^	
**Anal**	Doxazosin (OR = 3.19)	Nicotine (OR = 9.95)	Bisoprolol (OR = 3.30)	Nicotine (OR = 8.07)

*Abbreviations*: *OR* odds ratio, *HRT* hormone replacement therapy, *CNS* central nervous system

^†^Comorbidities include diabetes, myocardial infarction, coronary heart disease, heart failure, peripheral vascular disease, dementia, cerebrovascular disease, chronic obstructive pulmonary disease, osteoporosis, rheumatological disease, renal disease, liver disease, irritable bowel disease, human immunodeficiency viruses and hemiplegia/paraplegia, plus potential site-specific confounders

*Medicines applied for local effect, all other medicines systemic

^§^Analyses restricted to females only

^§§^Analyses restricted to males only

**Table 6:** Signals associated with decreased cancer risk (exposure: ≥ 6 prescriptions)

Cancer	Adjusted for comorbidities^**†**^	Adjusted for comorbidities^**†**^ and smoking
**Breast**^**§**^	Carbamazepine (OR = 0.70) Iron (OR = 0.78) Prednisolone (OR = 0.78) Trimethoprim (OR = 0.72)	Iron (OR = 0.72) Trazodone (OR = 0.62) Trimethoprim (OR = 0.71)
**Lung**	Benzalkonium* (OR = 0.49) Dimeticone* (OR = 0.46) Levodopa (OR = 0.38)	
**Colorectal**	Diclofenac (OR = 0.71) Lactulose (OR = 0.78) Misoprostol (OR = 0.69) Naproxen (OR = 0.72) Senna (OR = 0.73)	Diclofenac (OR = 0.72) Lactulose (OR = 0.77) Naproxen (OR = 0.71) Oxybutynin (OR = 0.60)
**Prostate**^**§§**^	Benzalkonium* (OR = 0.36) Bumetanide (OR = 0.57) Citalopram (OR = 0.59) Dimeticone* (OR = 0.36) Furosemide (OR = 0.70) Iron (OR = 0.72) Lactulose (OR = 0.68) Lidocaine* (OR = 0.35) Risperidone (OR = 0.27) Senna (OR = 0.70) Vitamin D (OR = 0.66)	Calcium (OR = 0.67) Citalopram (OR = 0.53) Furosemide (OR = 0.74) Lactulose (OR = 0.70) Senna (OR = 0.67) Vitamin D (OR = 0.60)
**Bladder**	Iron (OR = 0.64) Lactulose (OR = 0.73)	Pantoprazole (OR = 0.27) Sotalol (OR = 0.25)
**Malignant melanoma**	Gliclazide (OR = 0.51)	–
**Oesophagus**	–	Aspirin high dose (OR = 0.52)
**Non-Hodgkin’s** **lymphoma**	Vitamin B (OR = 0.51)	Vitamin B (OR = 0.51)
**Leukaemia**	–	Calcium (OR = 0.56)
**Oral (inc. head, neck, nasal)**	–	–
**Stomach**	Finasteride (OR = 0.41)^§§^	Diclofenac* (OR = 0.18)
**Ovary**^**§**^	Nifedipine (OR = 0.56) Progestogen-contraceptive (OR = 0.44)	–
**Kidney**	–	Digoxin (OR = 0.43)
**Uterus**^**§**^	Estrogen-HRT (OR = 0.68)	–
**Larynx**	Diclofenac (OR = 0.48) Doxazosin (OR = 0.31)	–

*Abbreviations*: *OR* odds ratio, *HRT* hormone replacement therapy

^†^Comorbidities include diabetes, myocardial infarction, coronary heart disease, heart failure, peripheral vascular disease, dementia, cerebrovascular disease, chronic obstructive pulmonary disease, osteoporosis, rheumatological disease, renal disease, liver disease, irritable bowel disease, human immunodeficiency viruses and hemiplegia/paraplegia, plus potential site-specific confounders

*Medicines applied for local effect, all other medicines systemic

^§^Analyses restricted to females only

^§§^Analyses restricted to males only

### MWAS plots

**Fig. 1:**
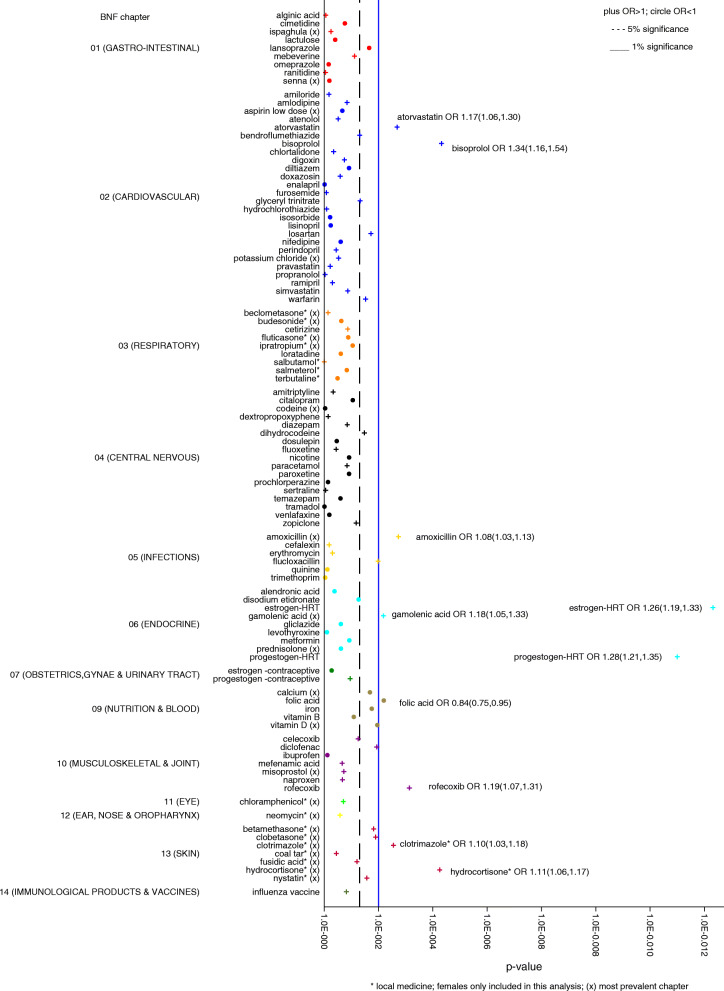
MWAS plot: breast cancer, comorbidity adjusted analysis (exposure: any prescription)

An MWAS plot for the most frequently prescribed medicines analysed in the most prevalent cancer site (breast) is given for illustrative purposes in Fig. 1. MWAS plots for all the medicines studied in each cancer site, with an exposure of any prescription, are found in Additional file 3: Fig. S1 & S2.

### Sensitivity analyses

Increasing the lag-time from 1 year to 2 years, or removing the 1155 matched sets where cases had an additional primary cancer diagnosis within 12 months of the index date, had a minimal effect on the estimated ORs and *p* values. Results obtained using multiple imputation for the comorbidity and smoking adjusted analyses were similar to those obtained using the 77.9% of patients who had available smoking data. These sensitivity analyses are detailed in Additional file 1: Tables S5 to S8.

## Discussion

### Principal findings

Using a population-based database, we conducted an exploratory set of analyses, systematically screening medicines frequently prescribed in relation to their potential carcinogenic or chemo-preventative properties for commonly diagnosed cancers, adjusting for relevant comorbidities and smoking. The vast majority of medicines did not meet the criteria for our definition of a signal. From these analyses, we identified 231 signals potentially worthy of further consideration. The majority of these signals (169) were associated with increased cancer risk, the remainder a reduced cancer risk and covered a variety of medicine types. Adjusting for smoking in addition to comorbidities identified 45 signals not identified when adjusting for comorbidities only.

### Context of other studies

This study follows the principles established in other screening studies to identify potential signals, namely by identifying effect sizes of interest, which are of statistical significance and where there is an exposure-response relationship between medicine and cancer. However, this study adjusts more extensively for comorbid conditions than previous screening studies, by using individual conditions and includes smoking in the analyses. Low availability of data on lifestyle factors is a limitation of current screening studies which the literature recognises [17].

Due to differences between studies, such as country of location, time of study, medicine licencing and grouping of cancers studied, it is not always possible to compare results directly between screening papers. However, as with other studies which have taken place to date, the vast majority of medicines are not associated with an increased risk of cancer. This should provide some reassurance to both patients and clinicians. Of those signals which have been identified, broadly speaking they can be divided into three groups. Firstly, there are signals which replicate well-known associations in the literature between medicine use and cancer risk, such as the increased risk of breast cancer associated with HRT medicine (Tables 3 and 5), [5], the reduced risk of oesophageal cancer with HRT medicine (Table 4) [41] and the reduced risk of colorectal cancer associated with some NSAIDs (e.g. diclofenac, naproxen (Table 6)) [42]. As such, our results provide reassurance that the study design and methodology employed are appropriate and informative.

Secondly, there are signals for which the relationship is unlikely to be causal. This may be due to a variety of factors, such as a chance finding due to multiple testing, reverse causation (e.g. tamoxifen and breast cancer) or omission of other appropriate confounders (such as BMI, a risk factor for cancers such as liver and colon [38]). Finally, there are some signals which merit further consideration. These include, for example, some antiplatelet/anticoagulant medicines and upper gastrointestinal cancer (warfarin and oesophageal cancer, clopidogrel and pancreatic cancer, Table 4). Both medicines are commonly prescribed, are intended for long-term use and can cause inflammation, [43, 44] a well-known risk factor for cancer. Possible mechanisms for a harmful association between clopidogrel and cancer include indirect modulation of the tumour growth, long-term platelet inhibition or instability of platelet-tumour cell aggregates [45]. As with all other signals, these need to be evaluated carefully in relation to clinical plausibility and causality [21], including application of the Bradford Hill criteria [46] in conjunction with more bespoke analyses.

This is the first screening study to adjust for smoking status. We observed that the effect of adjusting for smoking in addition to comorbidities varied with medicines and cancer sites. Some medicine-cancer associations which met the signal criteria after adjusting for comorbidities did not do so after additionally adjusting for smoking (e.g. cerivastatin and prostate cancer, cimetidine and stomach cancer (Table 3)). This is not unexpected for cancer sites where smoking is an important risk factor (e.g. lung, bladder, pancreas, prostate and stomach). Other medicine cancer associations met the signal criteria regardless of whether smoking was controlled for, even if the effect sizes were attenuated to some extent. Overall, the effect of adjusting for smoking varied between medicines and cancer sites, and we speculate that this suggests that smoking can both confound and synergise medicine-cancer associations in highly complex genetic, pharmacological and biological interactions.

In summary, the findings from our analyses highlight the need for additional analyses for signals of interest, tailored to the specific medicines and cancers.

### Strengths and limitations of study

There are a number of strengths to our study. This is the first time PCCIUR data has been used to undertake a systematic screening study determining medicines associated with an altered cancer risk. The PCCIUR is a nationally representative database, covering 15% of Scotland. The comprehensive linking of practice data to Scottish Cancer Registry data means there is a high coverage of cancer cases and a relatively long follow-up period of patients. Thorough cleaning and validation of the data has reduced the loss of prescription items due to transcription errors.

A further strength of the study is the incorporation of a wider range of risk factors into the models, including conditions relevant to individual cancer sites and smoking status, which have not been incorporated in any screening studies to-date. The replication of well-known associations between medicines and cancer risk suggests that the study design and methodology are appropriate and hence that other signals which are less-well documented are worthy of consideration in relation to their potential carcinogenic or chemo-preventative properties.

There are a number of limitations to this study. There are alternative ways in which prescriptions can be studied in relation to cancer risk other than by medicine, such as by Anatomical Therapeutic Chemical (ATC) code [47]. For topical medicines, there will be uncertainty as to how much medicine was administered, and absorption will vary due to factors such as the patient, the site of application, the formulation, the agent and the medical condition [48].

For our analyses, we grouped cancers together by cancer site as has been done in some other screening studies [12–16, 18]. This is useful in giving an overview by cancer site; however, histological subtypes of cancer each may vary in relation to their causal relationship with medicine, e.g. oesophageal adenocarcinoma and squamous cell carcinoma have different aetiologies and risk factors [28]. Smoking data were based upon primary care records, which have been shown to be reasonably accurate [49], but there remains the possibility of misclassification of smoking status. We did not have access to detailed smoking data, such as the quantity of cigarettes a patient smoked or the length of time they were a smoker. Where changes in the odds ratios after additionally adjusting for smoking are not as expected, smoking may possibly act as a proxy for other characteristics of an unhealthy lifestyle, such as lack of exercise or stress [50].

There are also a number of limitations to the statistical analyses. The large number of medicines studied within each cancer site increases the probability of type one error and undoubtedly some of the signals identified are false-positives. Although we used a 1% significance level, we did not apply a more stringent method to control for multiple testing, such as the Bonferroni correction [51] or false discovery rate control [52], as these would have reduced the likelihood of identifying true associations and because our analyses were exploratory in nature. This is similar to previous screening studies that have not applied any corrections for multiple testing [17] and consistent with arguments against multiple testing in general [53]. Due to the number of medicine-cancer associations investigated, it was not possible to create a set of bespoke confounders for each of the associations investigated nor to undertake more advanced handling of missing data. The use of median splits to categorise users as low users or high users can result in spurious results [54]; however, dose-response relationships are not necessarily linear and indeed these may often be non-linear [55]. Finally, it is inevitable that some of our analyses will be underpowered.

### Implications for policy and research

Results from our study show that the vast majority of prescribed medicines are not associated with an increase in the risk of cancer. This should provide reassurance to both patients and clinicians. However, given the increasing volume and consumption of medicines, the identification of medicines with cancer-limiting or cancer-increasing potential remains a global priority.

We recommend that researchers with expertise in specific cancers and/or medications examine the individual signals we have identified to prioritise those worthy of further investigation, either in preclinical studies and/or other prescribing databases. Medicines which are more likely to be prescribed long-term and/or prescribed to a greater number of people could be prioritised. We think more detailed analyses of specific medicine-cancer associations identified within PCCIUR data are of value. These analyses could include additional relevant confounding factors not included in this paper, consideration of daily defined doses (DDDs) (where these are available) and more sophisticated ways of analysis and addressing missing data could be considered [56]. Finally, additional screening studies should be conducted to attempt to identify further signals and which would allow us to validate our findings.

The medicine-cancer associations identified in our study require replication elsewhere. Should these associations be replicated, the use of medicines not previously known to increase the risk of cancer may require reconsideration of current licencing and use of such medicines. Medicines with chemo-preventative properties may warrant further study in clinical trials with a view to repurposing.

## Conclusions

This screening study has examined associations between medicine use and cancer risk in a sample of Scottish patients. The majority of medications are not associated with an altered risk of common cancers. There are novel candidate medicines which may have chemo-preventative or carcinogenic properties. Further analyses of such medicines are warranted.

## Supplementary Information

**Additional file 1: Table S1.** Comorbidity adjusted signals: exposure any prescription. **Table S2.** Comorbidity & smoking adjusted signals: exposure any prescription. **Table S3.** Comorbidity adjusted signals: exposure > = 6 prescriptions. **Table S4.** Comorbidity & smoking adjusted signals: exposure > = 6 prescriptions. **Table S5.** Sensitivity analyses for comorbidity adjusted signals: exposure any prescription. **Table S6.** Sensitivity analyses for comorbidity & smoking adjusted signals: exposure any prescription. **Table S7.** Sensitivity analyses for comorbidity adjusted signals: exposure > = 6 prescriptions. **Table S8.** Sensitivity analyses for comorbidity & smoking adjusted signals: exposure > = 6 prescriptions.

**Additional file 2.** Sample clinical or epidemiological references for identified signals.

**Additional file 3: Fig. S1.** MWAS plots for comorbidity adjusted analyses: exposure any prescription. **Fig. S2.** MWAS plots for comorbidity & smoking adjusted analyses: exposure any prescription.

## Data Availability

The datasets analysed in this study are not publicly available and were used under license. Requests for PCCIUR data should be directed in the first instance to Katie Wilde (Research Manager), email: k.wilde@abdn.ac.uk. Access to results for medicines not published in this paper should be directed to the corresponding author: Dr. Chris Cardwell, Senior Lecturer in Medical Statistics, Centre for Public Health, Queen’s University Belfast, email: c.cardwell@qub.ac.uk.

## Abbreviations

ATC: Anatomical Therapeutic Chemical
BMI: Body mass index
CCI: Charlson Comorbidity Index
CI: Confidence interval
EMA: European Medicines Agency
GP: General practitioner
HRT: Hormone replacement therapy
MI: Multiple imputation
MWAS: Medication-wide association study
NSAID: Non-steroidal anti-inflammatory drug
PCCIUR: Primary Care Clinical Information Unit Research
OR: Odds ratio
UK: United Kingdom

## References

[1] World Health Organisation (2020). The top 10 causes of death.

[2] World Health Organisation (2020). Cancer.

[3] International Agency for Research on Cancer (2020). Global Cancer Observatory.

[4] Cancer Research UK (2020). Cancer survival for common cancers.

[5] Collaborative Group on Hormonal Factors in Breast Cancer (2019). Type and timing of menopausal hormone therapy and breast cancer risk: individual participant meta-analysis of the worldwide epidemiological evidence. Lancet..

[6] Qiao Y, Yang T, Gan Y, Li W, Wang C, Gong Y (2018). Associations between aspirin use and the risk of cancers: a meta-analysis of observational studies. BMC Cancer.

[7] McNeil JJ, Nelson MR, Woods RL, Lockery JE, Wolfe R, Reid CM (2018). Effect of aspirin on all-cause mortality in the healthy elderly. N Engl J Med.

[8] Bowman L, Mafham M, Wallendszus K, Stevens W, Buck G, Barton J (2018). Effects of aspirin for primary prevention in persons with diabetes mellitus. N Engl J Med.

[9] Duijnhoven RG, Straus SMJM, Raine JM, de Boer A, Hoes AW, De Bruin ML (2013). Number of patients studied prior to approval of new medicines: a database analysis. PLoS Med.

[10] West-Strum D, Ynag Y, West-Strum D (2011). Chapter 1. Introduction to Pharmacoepidemiology. Understanding Pharmacoepidemiology.

[11] Jarvis L (2019). The new drugs of 2018. Chem Eng News.

[12] Friedman GD, Ury HK (1980). Initial screening for carcinogenicity of commonly used drugs. J Natl Cancer Inst.

[13] Friedman GD, Ury HK (1983). Screening for possible drug carcinogenicity: second report of findings. J Natl Cancer Inst.

[14] Selby J, Friedman G, Fireman B (1989). Screening prescription drugs for possible carcinogenicity: eleven to fifteen years of follow-up. Cancer Res.

[15] van den Eeden SK, Friedman GD (1995). Prescription drug screening for subsequent carcinogenicity. Pharmacoepidemiol Drug Saf.

[16] Friedman GD, Udaltsova N, Chan J, Quesenberry CP, Habel LA (2009). Screening pharmaceuticals for possible carcinogenic effects: initial positive results for drugs not previously screened. Cancer Causes Control.

[17] Pottegård A, Friis S, Christensen R dePont, Habel LA, Gagne JJ, Hallas J. Identification of associations between prescribed medications and cancer: a nationwide screening study. EBioMedicine. 2016;7:73–79. doi:10.1016/j.ebiom.2016.03.018. 10.1016/j.ebiom.2016.03.018 PMC4909325 27322460

[18] Patel CJ, Ji J, Sundquist J, Ioannidis JPA, Sundquist K (2016). Systematic assessment of pharmaceutical prescriptions in association with cancer risk: a method to conduct a population-wide medication-wide longitudinal study. Sci Rep.

[19] Andreassen BK, Støer NC, Martinsen JI, Ursin G, Weiderpass E, Thoresen GH (2019). Identification of potential carcinogenic and chemopreventive effects of prescription drugs: a protocol for a Norwegian registry-based study. BMJ Open.

[20] Jacob L, Freyn M, Kalder M, Dinas K, Kostev K (2018). Impact of tobacco smoking on the risk of developing 25 different cancers in the UK: a retrospective study of 422,010 patients followed for up to 30 years. Oncotarget..

[21] Pottegård A, Hallas J, Wang SV, Gagne JJ (2018). Identifying signals of interest when screening for drug-outcome associations in health care data. Br J Clin Pharmacol.

[22] University of Aberdeen. Primary Care Clinical Informatics Unit Research, The Institute of Applied Health Sciences. https://www.abdn.ac.uk/iahs/research/primary-care/pcciur/index.php. Accessed 18 Feb 2019.

[23] Busby J, Murchie P, Murray L, Iversen L, Lee AJ, Spence A (2017). The effect of medications which cause inflammation of the gastro-oesophageal tract on cancer risk: a nested case–control study of routine Scottish data. Int J Cancer.

[24] Macfarlane TV, Lefevre K, Watson MC (2014). Aspirin and non-steroidal anti-inflammatory drug use and the risk of upper aerodigestive tract cancer. Br J Cancer.

[25] Tran KT, McMenamin C, Hicks B, Murchie P, Thrift AP, Coleman HG (2018). Proton pump inhibitor and histamine-2 receptor antagonist use and risk of liver cancer in two population-based studies. Aliment Pharmacol Ther.

[26] Spence AD, Busby J, Murchie P, Kunzmann AT, McMenamin ÚC, Coleman HG (2018). Medications that relax the lower oesophageal sphincter and risk of oesophageal cancer: an analysis of two independent population-based databases. Int J Cancer.

[27] Pottegård A, Hallas J (2017). New use of prescription drugs prior to a cancer diagnosis. Pharmacoepidemiol Drug Saf.

[28] Pottegård A, Friis S, Stürmer T, Hallas J, Bahmanyar S (2018). Considerations for pharmacoepidemiological studies of drug-cancer associations. Basic Clin Pharmacol Toxicol.

[29] National Health Service UK. Aspirin: low dose to prevent heart attacks and stroke - NHS. https://www.nhs.uk/medicines/low-dose-aspirin/. Accessed 17 Sep 2020.

[30] National Health Service UK. NSAIDs. https://www.nhs.uk/conditions/nsaids/. Accessed 18 Sep 2020.

[31] Minar E, Ahmadi A, Koppensteiner R, Maca T, Stümpflen A, Ugurluoglu A (1995). Comparison of effects of high-dose and low-dose aspirin on restenosis after femoropopliteal percutaneous transluminal angioplasty. Circulation..

[32] Skriver C, Dehlendorff C, Borre M, Brasso K, Sørensen HT, Hallas J (2016). Low-dose aspirin or other nonsteroidal anti-inflammatory drug use and prostate cancer risk: a nationwide study. Cancer Causes Control.

[33] Yu J, Mehran R, Dangas GD, Claessen BE, Baber U, Xu K (2012). Safety and efficacy of high- versus low-dose aspirin after primary percutaneous coronary intervention in ST-segment elevation myocardial infarction: the HORIZONS-AMI (Harmonizing Outcomes with Revascularization and Stents in Acute Myocardial Infarction) trial. JACC Cardiovasc Interv.

[34] Khan NF, Perera R, Harper S, Rose PW (2010). Adaptation and validation of the Charlson Index for Read/OXMIS coded databases. BMC Fam Pract.

[35] Jespersen CG, Nørgaard M, Borre M (2016). Parkinson’s disease and risk of prostate cancer: a Danish population-based case-control study, 1995-2010. Cancer Epidemiol.

[36] Hong S, Mok Y, Jeon C, Jee SH, Samet JM (2016). Tuberculosis, smoking and risk for lung cancer incidence and mortality. Int J Cancer.

[37] Karami S, Daugherty SE, Purdue MP (2014). Hysterectomy and kidney cancer risk: a meta-analysis. Int J Cancer.

[38] Bhaskaran K, Douglas I, Forbes H, Dos-Santos-Silva I, Leon DA, Smeeth L (2014). Body-mass index and risk of 22 specific cancers: a population-based cohort study of 5·24 million UK adults. Lancet..

[39] Joint Formulary Committee (2020). British National Formulary.

[40] StataCorp (2017). Stata Statistical Software: Release 15.

[41] Lagergren K, Lagergren J, Brusselaers N (2014). Hormone replacement therapy and oral contraceptives and risk of oesophageal adenocarcinoma: a systematic review and meta-analysis. Int J Cancer.

[42] Thun MJ, Jane Henley S, Patrono C (2002). Nonsteroidal anti-inflammatory drugs as anticancer agents: mechanistic, pharmacologic, and clinical issues. J Natl Cancer Inst.

[43] Zografos GN, Georgiadou D, Thomas D, Kaltsas G, Digalakis M (2009). Drug-induced esophagitis. Dis Esophagus.

[44] Lai SW, Lin CL, Liao KF (2015). Actively using clopidogrel correlates with an increased risk of acute pancreatitis in Taiwan. Int J Cardiol.

[45] Serebruany VL (2009). Platelet inhibition with prasugrel and increased cancer risks: potential causes and implications. Am J Med.

[46] Hill AB (1965). The environment and disease: association or causation?. Proc R Soc Med.

[47] World Health Organisation. The Anatomical Therapeutic Chemical Classification System with Defined Daily Doses (ATC/DDD). WHO. 2010. https://www.who.int/classifications/atcddd/en/. Accessed 17 Feb 2020.

[48] Barkin RL (2015). Topical nonsteroidal anti-inflammatory drugs: the importance of drug, delivery, and therapeutic outcome. Am J Ther.

[49] Atkinson MD, Kennedy JI, John A, Lewis KE, Lyons RA, Brophy ST, et al. Development of an algorithm for determining smoking status and behaviour over the life course from UK electronic primary care records. BMC Med Inform Decis Mak. 2017;17:2. 10.1186/s12911-016-0400-6 PMC5217540 28056955

[50] Lohse T, Rohrmann S, Bopp M, Faeh D. Heavy smoking is more strongly associated with general unhealthy lifestyle than obesity and underweight. PLoS One. 2016;11. 10.1371/journal.pone.0148563. 10.1371/journal.pone.0148563 PMC4765891 26910775

[51] Bonferroni C (1936). Teoria statistica delle classi e calcolo delle probabilit. Pubbl del R Ist Super di Sci Econ e Commer di Firenze.

[52] Benjamini Y, Hochberg Y. Controlling the false discovery rate: a practical and powerful approach to multiple testing. J R Stat Soc Ser B. 1995.

[53] Rothman KJ (1990). No adjustments are needed for multiple comparisons. Epidemiology..

[54] DeCoster J, Gallucci M, Iselin A-MR (2011). Best practices for using median splits, artificial categorization, and their continuous alternatives. J Exp Psychopathol.

[55] Goldsmith JR, Kordysh E. Why dose-response relationships are often non-linear and some consequences. J Expo Anal Environ Epidemiol. 3:259–76 http://www.ncbi.nlm.nih.gov/pubmed/8260836. Accessed 1 May 2020. 8260836

[56] Wang SV, Kulldorff M, Glynn RJ, Gagne JJ, Pottegård A, Rothman KJ (2018). Reuse of data sources to evaluate drug safety signals: when is it appropriate?. Pharmacoepidemiol Drug Saf.

