# Supplementary material for: A systematic assessment of the association between frequently prescribed medicines and the risk of common cancers: a series of nested case-control studies
Source: BMC Med. 2021 Jan 26;19:22. doi: 10.1186/s12916-020-01891-5 (PMC7836181; doi:10.1186/s12916-020-01891-5)
Supplement: Supplementary file 1 — Additional file 1: Table S1. Comorbidity adjusted signals: exposure any prescription. Table S2. Comorbidity & smoking adjusted signals: exposure any prescription. Table S3. Comorbidity adjusted signals: exposure > = 6 prescriptions. Table S4. Comorbidity & smoking adjusted signals: exposure > = 6 prescriptions. Table S5. Sensitivity analyses for comorbidity adjusted signals: exposure any prescription. Table S6. Sensitivity analyses for comorbidity & smoking adjusted signals: exposure any prescription. Table S7. Sensitivity analyses for comorbidity adjusted signals: exposure > = 6 prescriptions. Table S8. Sensitivity analyses for comorbidity & smoking adjusted signals: exposure > = 6 prescriptions. [file 12916_2020_1891_MOESM1_ESM.pdf]

Supplementary tables

Index of Legends

Table S1: Comorbidity adjusted signals: exposure any prescription

Table S2: Comorbidity & smoking adjusted signals: exposure any prescription

Table S3: Comorbidity adjusted signals: exposure >=6 prescriptions

Table S4: Comorbidity & smoking adjusted signals: exposure >=6 prescriptions

Table S5: Sensitivity analyses for comorbidity adjusted signals: exposure any prescription

Table S6: Sensitivity analyses for comorbidity & smoking adjusted signals: exposure any prescription

Table S7: Sensitivity analyses for comorbidity adjusted signals: exposure >=6 prescriptions

Table S8: Sensitivity analyses for comorbidity & smoking adjusted signals: exposure >=6 prescriptions

Table S1: Comorbidity† adjusted signals: exposure any prescription

Cancer	Medicine	No. (%) cases	No. (%) controls	OR (95%CI)	p-value	Median ††	Low use v non-use: OR (95%CI)	High use v non-use: OR (95%CI)
**Signals associated with increased cancer risk**								
**Breast §**	**estrogen-HRT**	2,524(20.6%)	10,619(18.6%)	1.26(1.19,1.33)	<0.001	7	1.15(1.07,1.23)	1.41(1.30,1.52)
**Breast §**	**metronidazole***	188(1.5%)	745(1.3%)	1.25(1.06,1.48)	0.007	1	1.24(1.01,1.52)	1.29(0.98,1.69)
**Breast §**	**progestogen-HRT**	2,339(19.1%)	9,706(17.0%)	1.28(1.21,1.35)	<0.001	4	1.07(1.00,1.16)	1.51(1.40,1.62)
**Lung**	**amoxicillin**	4,380(46.2%)	15,145(36.5%)	1.40(1.33,1.48)	<0.001	2	1.32(1.24,1.40)	1.62(1.50,1.75)
**Lung**	**azathioprine**	66(0.7%)	165(0.4%)	1.56(1.14,2.13)	0.005	11	1.08(0.66,1.75)	2.09(1.39,3.14)
**Lung**	**cimetidine**	880(9.3%)	2,685(6.5%)	1.39(1.27,1.51)	<0.001	4	1.30(1.16,1.47)	1.48(1.31,1.66)
**Lung**	**clarithromycin**	1,108(11.7%)	3,083(7.4%)	1.34(1.23,1.46)	<0.001	1	1.34(1.21,1.47)	1.35(1.18,1.55)
**Lung**	**codeine**	3,510(37.0%)	12,131(29.3%)	1.31(1.24,1.38)	<0.001	3	1.28(1.20,1.37)	1.33(1.24,1.43)
**Lung**	**dihydrocodeine**	1,833(19.3%)	5,911(14.3%)	1.29(1.21,1.38)	<0.001	2	1.22(1.12,1.33)	1.37(1.25,1.49)
**Lung**	**folic acid §**	273(6.5%)	719(3.8%)	1.49(1.27,1.75)	<0.001	6	1.37(1.11,1.70)	1.64(1.31,2.06)
**Lung**	**ipratropium***	1,086(11.4%)	1,658(4.0%)	1.58(1.43,1.74)	<0.001	8	1.40(1.23,1.59)	1.77(1.56,2.02)
**Lung**	**nitrazepam**	231(2.4%)	624(1.5%)	1.35(1.14,1.60)	<0.001	10	1.17(0.91,1.50)	1.52(1.22,1.91)
**Lung**	**nystatin**	429(4.5%)	1,117(2.7%)	1.27(1.12,1.44)	<0.001	1	1.21(1.04,1.41)	1.40(1.14,1.72)
**Lung**	**paracetamol**	5,379(56.7%)	19,230(46.4%)	1.34(1.27,1.42)	<0.001	7	1.31(1.23,1.39)	1.39(1.30,1.48)
**Lung**	**salbutamol***	2,372(25.0%)	5,381(13.0%)	1.44(1.34,1.54)	<0.001	9	1.37(1.26,1.50)	1.52(1.39,1.66)
**Lung**	**salmeterol***	901(9.5%)	1,704(4.1%)	1.34(1.21,1.48)	<0.001	12	1.30(1.14,1.49)	1.37(1.20,1.57)
**Lung**	**tiotropium***	516(5.4%)	593(1.4%)	1.75(1.52,2.02)	<0.001	7	1.73(1.43,2.09)	1.78(1.46,2.16)
**Colorectal**	**allopurinol**	298(3.4%)	950(2.5%)	1.27(1.11,1.46)	0.001	17	1.07(0.87,1.32)	1.48(1.23,1.78)
**Colorectal**	**prednisolone***	223(2.6%)	728(1.9%)	1.31(1.12,1.54)	0.001	1	1.31(1.06,1.62)	1.32(1.04,1.66)
**Prostate §§**	**cerivastatin**	86(1.2%)	271(0.8%)	1.43(1.10,1.87)	0.008	8	1.17(0.80,1.73)	1.74(1.21,2.50)
**Prostate §§**	**clioquinol***	245(3.3%)	851(2.7%)	1.30(1.11,1.52)	0.001	1	1.23(1.01,1.49)	1.43(1.12,1.83)
**Bladder**	**celecoxib**	144(4.5%)	446(3.2%)	1.40(1.14,1.73)	0.002	2	1.30(0.98,1.71)	1.55(1.15,2.09)
**Bladder**	**dexamethasone***	203(6.3%)	677(4.9%)	1.30(1.10,1.54)	0.003	1	1.29(1.03,1.61)	1.32(1.02,1.70)
**Bladder**	**nicotine**	152(4.7%)	362(2.6%)	2.04(1.65,2.51)	<0.001	2	1.76(1.35,2.28)	2.65(1.90,3.70)
**Bladder**	**trimethoprim**	562(17.4%)	1,388(10.0%)	1.96(1.73,2.21)	<0.001	1	1.81(1.56,2.09)	2.22(1.87,2.65)
**Malignant melanoma**	**chloramphenicol***	230(9.3%)	850(7.5%)	1.28(1.08,1.51)	0.003	1	1.22(1.01,1.48)	1.42(1.08,1.88)
**Malignant melanoma**	**clopidogrel**	53(2.2%)	160(1.4%)	1.60(1.13,2.27)	0.008	6	1.31(0.79,2.18)	1.89(1.21,2.96)
**Malignant melanoma**	**flucloxacillin**	349(14.2%)	1,317(11.6%)	1.28(1.11,1.46)	<0.001	1	1.24(1.06,1.46)	1.36(1.08,1.71)
**Oesophagus**	**alginic acid**	335(13.9%)	995(9.5%)	1.40(1.22,1.62)	<0.001	5	1.26(1.03,1.54)	1.56(1.29,1.89)
**Oesophagus**	**azathioprine**	22(0.9%)	36(0.3%)	2.47(1.41,4.35)	0.002	9	2.37(1.06,5.29)	2.58(1.17,5.68)
**Oesophagus**	**cisapride**	30(1.2%)	61(0.6%)	2.03(1.27,3.25)	0.003	3	2.00(1.04,3.84)	2.07(1.06,4.03)
**Oesophagus**	**gramicidin***	61(2.5%)	174(1.7%)	1.52(1.11,2.09)	0.008	1	1.52(1.05,2.19)	1.54(0.86,2.76)
**Oesophagus**	**lansoprazole**	363(15.1%)	1,192(11.4%)	1.35(1.17,1.55)	<0.001	4	1.10(0.91,1.33)	1.65(1.38,1.98)
**Oesophagus**	**nicotine**	97(4.0%)	300(2.9%)	1.50(1.16,1.94)	0.002	2	1.48(1.08,2.02)	1.54(1.03,2.30)
**Oesophagus**	**nystatin**	86(3.6%)	230(2.2%)	1.58(1.21,2.06)	0.001	1	1.47(1.05,2.06)	1.78(1.16,2.72)
**Oesophagus**	**nystatin***	191(8.0%)	643(6.1%)	1.29(1.08,1.55)	0.005	1	1.29(1.02,1.63)	1.30(0.99,1.70)
**Oesophagus**	**omeprazole**	490(20.4%)	1,596(15.2%)	1.39(1.23,1.57)	<0.001	6	1.26(1.06,1.48)	1.54(1.31,1.81)
**Oesophagus**	**triamcinolone***	88(3.7%)	267(2.6%)	1.47(1.13,1.91)	0.004	1	1.39(0.99,1.93)	1.61(1.07,2.42)
**Oesophagus**	**vitamin B**	174(7.2%)	514(4.9%)	1.36(1.12,1.65)	0.002	5	1.20(0.92,1.56)	1.56(1.20,2.04)

Table S1 (cont)

Cancer	Medicine	No. (%) cases	No. (%) controls	OR (95%CI)	p-value	Median ††	Low use v non-use: OR (95%CI)	High use v non-use: OR (95%CI)
**Non-Hodgkin's lymphoma**	**amoxicillin**	798(38.4%)	3,040(32.4%)	1.34(1.19,1.50)	<0.001	2	1.28(1.13,1.45)	1.55(1.29,1.85)
**Non-Hodgkin's lymphoma**	**betamethasone***	354(17.0%)	1,209(12.9%)	1.37(1.19,1.57)	<0.001	1	1.27(1.06,1.53)	1.47(1.22,1.78)
**Non-Hodgkin's lymphoma**	**chlorhexidine***	59(2.8%)	142(1.5%)	1.67(1.20,2.31)	0.002	1	1.45(0.97,2.17)	2.21(1.28,3.81)
**Non-Hodgkin's lymphoma**	**clarithromycin**	167(8.0%)	586(6.2%)	1.33(1.09,1.63)	0.005	1	1.28(1.02,1.62)	1.45(1.03,2.02)
**Non-Hodgkin's lymphoma**	**clobetasol***	59(2.8%)	151(1.6%)	1.73(1.25,2.38)	0.001	1	1.56(1.01,2.40)	1.96(1.22,3.15)
**Non-Hodgkin's lymphoma**	**erythromycin**	248(11.9%)	918(9.8%)	1.26(1.07,1.49)	0.006	1	1.17(0.95,1.43)	1.44(1.12,1.85)
**Non-Hodgkin's lymphoma**	**hydroxyzine**	35(1.7%)	81(0.9%)	1.75(1.16,2.66)	0.008	1	1.45(0.82,2.57)	2.22(1.20,4.09)
**Non-Hodgkin's lymphoma**	**methotrexate**	19(0.9%)	30(0.3%)	2.93(1.50,5.69)	0.002	11	2.74(1.11,6.78)	3.12(1.28,7.56)
**Non-Hodgkin's lymphoma**	**prednisolone**	199(9.6%)	605(6.4%)	1.37(1.13,1.66)	0.001	2	1.31(1.02,1.67)	1.45(1.10,1.91)
**Non-Hodgkin's lymphoma**	**prochlorperazine**	176(8.5%)	614(6.5%)	1.30(1.08,1.58)	0.007	1	1.20(0.94,1.53)	1.46(1.11,1.93)
**Leukaemia**	**allopurinol**	80(4.0%)	221(2.5%)	1.57(1.20,2.06)	0.001	14	1.38(0.93,2.05)	1.75(1.22,2.52)
**Leukaemia**	**amoxicillin**	700(35.0%)	2,743(31.0%)	1.29(1.14,1.45)	<0.001	2	1.27(1.12,1.45)	1.33(1.11,1.60)
**Leukaemia**	**trimethoprim**	228(11.4%)	855(9.7%)	1.26(1.06,1.49)	0.010	1	1.10(0.89,1.36)	1.53(1.20,1.97)
**Oral (inc. Head,neck & nasal)**	**codeine**	467(31.8%)	1,646(24.5%)	1.29(1.12,1.49)	<0.001	3	1.23(1.04,1.47)	1.37(1.13,1.65)
**Oral (inc. Head,neck & nasal)**	**flucloxacillin**	219(14.9%)	793(11.8%)	1.36(1.13,1.62)	0.001	1	1.29(1.05,1.59)	1.52(1.13,2.05)
**Oral (inc. Head,neck & nasal)**	**folic acid §**	51(10.9%)	81(3.8%)	2.52(1.64,3.89)	<0.001	4	1.50(0.79,2.82)	4.03(2.27,7.17)
**Oral (inc. Head,neck & nasal)**	**nystatin**	68(4.6%)	137(2.0%)	2.06(1.49,2.85)	<0.001	1	1.87(1.26,2.77)	2.50(1.46,4.27)
**Oral (inc. Head,neck & nasal)**	**vitamin B**	214(14.6%)	308(4.6%)	2.75(2.22,3.41)	<0.001	4	2.31(1.72,3.11)	3.23(2.43,4.29)
**Oral (inc. Head,neck & nasal)**	**vitamin D**	103(7.0%)	213(3.2%)	1.69(1.24,2.30)	0.001	6	1.56(1.06,2.31)	1.85(1.22,2.80)
**Stomach**	**cimetidine**	133(9.3%)	411(6.6%)	1.44(1.15,1.79)	0.001	4	1.32(0.98,1.79)	1.56(1.15,2.11)
**Stomach**	**clarithromycin**	147(10.3%)	466(7.5%)	1.35(1.08,1.69)	0.009	1	1.25(0.97,1.63)	1.59(1.10,2.29)
**Stomach**	**digoxin**	75(5.2%)	216(3.5%)	1.49(1.10,2.01)	0.010	12	1.41(0.93,2.16)	1.56(1.04,2.32)
**Stomach**	**vitamin B**	144(10.0%)	308(5.0%)	1.54(1.21,1.95)	<0.001	5	1.54(1.12,2.10)	1.54(1.12,2.13)
**Ovary §**	**phenytoin**	14(1.0%)	24(0.4%)	3.24(1.62,6.50)	0.001	27	2.48(0.97,6.31)	4.67(1.62,13.46)
**Ovary §**	**rabeprazole**	18(1.3%)	45(0.7%)	2.18(1.22,3.89)	0.009	2	1.93(0.84,4.42)	2.44(1.11,5.36)
**Kidney**	**amoxicillin**	541(40.0%)	2,108(34.7%)	1.28(1.11,1.48)	0.001	2	1.20(1.02,1.40)	1.57(1.26,1.94)
**Kidney**	**hydrochlorothiazide**	44(3.3%)	90(1.5%)	1.93(1.30,2.86)	0.001	13	1.50(0.86,2.62)	2.50(1.45,4.31)
**Kidney**	**perindopril**	63(4.7%)	132(2.2%)	1.63(1.16,2.30)	0.005	10	1.46(0.92,2.32)	1.85(1.15,2.97)
**Pancreas**	**betamethasone***	205(19.2%)	716(15.1%)	1.34(1.11,1.62)	0.003	1	1.19(0.93,1.52)	1.52(1.18,1.96)
**Pancreas**	**nicotine**	60(5.6%)	151(3.2%)	1.94(1.38,2.73)	<0.001	2	1.91(1.24,2.94)	1.97(1.18,3.28)
**Cervix & other gynae §**	**dihydrocodeine**	136(13.1%)	487(10.1%)	1.39(1.11,1.73)	0.004	2	1.31(0.98,1.74)	1.49(1.10,2.03)
**Cervix & other gynae §**	**ranitidine**	109(10.5%)	374(7.7%)	1.37(1.08,1.73)	0.010	3	1.15(0.83,1.60)	1.64(1.18,2.26)
**Uterus §**	**atenolol**	236(23.4%)	810(17.2%)	1.39(1.15,1.67)	0.001	17	1.26(0.99,1.60)	1.54(1.21,1.96)
**Uterus §**	**bendroflumethiazide**	284(28.1%)	1,064(22.6%)	1.27(1.07,1.51)	0.005	14	1.08(0.86,1.34)	1.53(1.22,1.90)
**Uterus §**	**bisoprolol**	41(4.1%)	112(2.4%)	1.82(1.21,2.74)	0.004	15	1.65(0.97,2.80)	2.07(1.16,3.71)
**Uterus §**	**doxazosin**	47(4.7%)	121(2.6%)	1.68(1.17,2.43)	0.005	9	1.24(0.73,2.13)	2.23(1.37,3.64)
**Uterus §**	**enalapril**	58(5.7%)	149(3.2%)	1.66(1.18,2.33)	0.003	14	1.16(0.69,1.94)	2.23(1.43,3.47)
**Uterus §**	**ibuprofen***	73(7.2%)	221(4.7%)	1.53(1.14,2.06)	0.005	1	1.49(1.04,2.15)	1.60(1.01,2.54)

Table S1 (cont)

Cancer	Medicine	No. (%) cases	No. (%) controls	OR (95%CI)	p-value	Median ††	Low use v non-use: OR (95%CI)	High use v non-use: OR (95%CI)
**Uterus §**	**mefenamic acid**	53(5.2%)	171(3.6%)	1.72(1.22,2.42)	0.002	1	1.52(0.93,2.47)	1.92(1.22,3.03)
**Uterus §**	**tranexamic acid**	39(3.9%)	116(2.5%)	1.87(1.25,2.79)	0.002	1	1.65(0.99,2.76)	2.26(1.23,4.14)
**Larynx**	**benzydamine***	26(3.5%)	50(1.5%)	2.33(1.34,4.04)	0.003	1	1.60(0.81,3.17)	5.09(2.00,12.98)
**Larynx**	**dextropropoxyphene**	146(19.7%)	423(12.7%)	1.51(1.19,1.92)	0.001	4	1.43(1.04,1.97)	1.59(1.16,2.17)
**Larynx**	**paracetamol**	366(49.4%)	1,255(37.8%)	1.45(1.20,1.76)	<0.001	5	1.41(1.13,1.77)	1.50(1.18,1.92)
**Larynx**	**vitamin B**	81(10.9%)	134(4.0%)	2.40(1.72,3.36)	<0.001	6	2.19(1.40,3.40)	2.66(1.68,4.21)
**Brain and CNS**	**carbamazepine**	25(3.4%)	61(1.8%)	2.20(1.34,3.62)	0.002	6	1.43(0.69,2.97)	3.37(1.72,6.62)
**Myeloma**	**amoxicillin**	227(42.0%)	833(34.8%)	1.53(1.21,1.92)	<0.001	2	1.45(1.13,1.85)	1.77(1.26,2.49)
**Myeloma**	**ciprofloxacin**	46(8.5%)	131(5.5%)	1.73(1.17,2.55)	0.006	1	1.72(1.10,2.68)	1.77(0.88,3.55)
**Thyroid**	**gabapentin**	7(2.1%)	7(0.4%)	5.13(1.71,15.37)	0.003	2	4.60(1.08,19.56)	5.93(1.12,31.26)
**Thyroid**	**levothyroxine**	33(9.7%)	79(4.9%)	2.18(1.37,3.48)	0.001	18	1.57(0.79,3.13)	2.87(1.57,5.24)
**Anal**	**clonidine**	9(3.2%)	13(1.0%)	3.87(1.52,9.85)	0.005	2	3.86(1.33,11.22)	3.89(0.57,26.72)
**Anal**	**doxazosin**	17(6.1%)	37(2.9%)	2.48(1.27,4.86)	0.008	11	1.68(0.62,4.57)	3.37(1.42,7.97)
**Signals associated with decreased cancer risk**								
**Breast §**	**trazodone**	187(1.5%)	1,042(1.8%)	0.79(0.67,0.93)	0.005	3	0.80(0.64,0.99)	0.78(0.61,0.99)
**Lung**	**fluocinolone***	74(0.8%)	460(1.1%)	0.67(0.52,0.88)	0.003	2	0.70(0.51,0.98)	0.62(0.40,0.96)
**Lung**	**levodopa**	35(0.4%)	244(0.6%)	0.50(0.35,0.73)	<0.001	19	0.66(0.41,1.05)	0.34(0.18,0.64)
**Lung**	**nitrofurantoin**	110(1.2%)	555(1.3%)	0.74(0.60,0.93)	0.008	1	0.75(0.57,0.98)	0.73(0.51,1.06)
**Lung**	**progestogen-contraceptive §**	53(1.3%)	435(2.3%)	0.62(0.45,0.86)	0.004	4	0.70(0.48,1.03)	0.51(0.31,0.85)
**Lung**	**risperidone**	27(0.3%)	229(0.6%)	0.55(0.36,0.84)	0.005	9	0.59(0.34,1.02)	0.50(0.27,0.95)
**Colorectal**	**levodopa**	40(0.5%)	242(0.6%)	0.60(0.43,0.85)	0.004	22	0.71(0.45,1.11)	0.49(0.29,0.84)
**Colorectal**	**lofepramine**	74(0.9%)	421(1.1%)	0.71(0.55,0.92)	0.009	3	0.71(0.50,1.01)	0.71(0.48,1.03)
**Colorectal**	**meloxicam**	141(1.6%)	781(2.1%)	0.76(0.63,0.92)	0.005	2	0.78(0.60,1.01)	0.74(0.57,0.97)
**Colorectal**	**metoclopramide**	235(2.7%)	1,256(3.3%)	0.78(0.68,0.91)	0.001	1	0.92(0.77,1.11)	0.60(0.47,0.77)
**Colorectal**	**nitrofurantoin**	91(1.0%)	482(1.3%)	0.71(0.56,0.91)	0.006	1	0.79(0.60,1.05)	0.58(0.39,0.88)
**Prostate §§**	**levodopa**	42(0.6%)	248(0.8%)	0.47(0.30,0.72)	0.001	17	0.53(0.32,0.87)	0.39(0.21,0.73)
**Prostate §§**	**risperidone**	12(0.2%)	122(0.4%)	0.37(0.20,0.70)	0.002	8	0.40(0.18,0.89)	0.35(0.13,0.91)
**Prostate §§**	**senna**	301(4.0%)	1,378(4.3%)	0.76(0.66,0.87)	<0.001	2	0.87(0.73,1.04)	0.63(0.51,0.78)
**Bladder**	**iron**	196(6.1%)	855(6.1%)	0.77(0.64,0.91)	0.003	2	0.84(0.66,1.06)	0.70(0.55,0.89)
**Malignant melanoma**	**dipyridamole**	12(0.5%)	105(0.9%)	0.42(0.22,0.80)	0.008	13	0.51(0.23,1.13)	0.31(0.11,0.89)
**Malignant melanoma**	**gliclazide**	30(1.2%)	200(1.8%)	0.51(0.32,0.80)	0.004	16	0.57(0.33,1.00)	0.44(0.23,0.85)
**Oesophagus**	**estrogen-HRT §**	70(9.0%)	509(15.2%)	0.59(0.43,0.79)	<0.001	8	0.63(0.43,0.93)	0.54(0.36,0.81)
**Non-Hodgkin's lymphoma**	**progestogen-contraceptive §**	38(3.6%)	298(6.3%)	0.56(0.36,0.86)	0.008	4	0.64(0.39,1.05)	0.44(0.23,0.85)
**Oral (inc. Head,neck & nasal)**	**doxycycline**	32(2.2%)	269(4.0%)	0.45(0.30,0.66)	<0.001	1	0.50(0.32,0.80)	0.33(0.15,0.70)
**Oral (inc. Head,neck & nasal)**	**ispaghula**	62(4.2%)	343(5.1%)	0.63(0.47,0.85)	0.002	2	0.72(0.49,1.07)	0.54(0.35,0.83)
**Ovary §**	**loratadine**	46(3.2%)	300(4.6%)	0.64(0.46,0.89)	0.008	1	0.71(0.47,1.08)	0.55(0.32,0.93)
**Ovary §**	**progestogen-contraceptive**	50(3.5%)	414(6.4%)	0.55(0.39,0.78)	0.001	3	0.64(0.42,0.98)	0.45(0.27,0.74)
**Cervix & other gynae §**	**terbinafine**	4(0.4%)	96(2.0%)	0.18(0.07,0.49)	0.001	2	0.23(0.08,0.62)	-

Table S1 (cont)

Cancer	Medicine	No. (%) cases	No. (%) controls	OR (95%CI)	p-value	Median ††	Low use v non-use: OR (95%CI)	High use v non-use: OR (95%CI)
**Uterus §**	**estrogen-HRT**	169(16.7%)	1,102(23.4%)	0.73(0.60,0.89)	0.002	7	0.76(0.59,0.98)	0.70(0.54,0.92)
**Uterus §**	**progestogen-contraceptive**	12(1.2%)	169(3.6%)	0.37(0.19,0.69)	0.002	3	0.41(0.18,0.90)	0.32(0.12,0.82)
**Larynx**	**doxazosin**	13(1.8%)	90(2.7%)	0.36(0.19,0.70)	0.002	14	0.49(0.22,1.12)	0.24(0.09,0.69)
**Larynx**	**terbinafine**	7(0.9%)	96(2.9%)	0.32(0.14,0.75)	0.009	2	0.44(0.18,1.06)	-

† comorbidities include diabetes, myocardial infarction, coronary heart disease, heart failure, peripheral vascular disease, dementia, cerebrovascular disease, chronic obstructive pulmonary disease, osteoporosis, rheumatological disease, renal disease, liver disease, irritable bowel disease, human immunodeficiency viruses and hemiplegia/paraplegia, plus potential site-specific confounders; OR odds ratio; CI confidence interval; CNS: central nervous system; medicines systemic unless otherwise indicated* (local); HRT: hormone replacement therapy; § analyses restricted to females only; §§ analyses restricted to males only; low use: patient received more than I item of medicine and <=median number of items; high use: patient received > median number of items of medicine; †† among control users; - no cases with prescription in category

Table S2: Comorbidity† & smoking adjusted signals: exposure any prescription

Cancer	Medicine	No. (%) cases	No. (%) controls	OR (95%CI)	p-value	Median ††	Low use v non-use: OR (95%CI)	High use v non-use: OR (95%CI)
**Signals associated with increased cancer risk**								
**Breast §**	**bisoprolol**	272(2.8%)	908(2.0%)	1.30(1.11,1.51)	0.001	12	1.28(1.03,1.58)	1.31(1.07,1.62)
**Breast §**	**metronidazole***	176(1.8%)	676(1.5%)	1.26(1.06,1.51)	0.010	1	1.25(1.00,1.56)	1.29(0.97,1.72)
**Breast §**	**progestogen (HRT)**	2,050(20.8%)	8,552(19.0%)	1.25(1.17,1.33)	<0.001	4	1.04(0.95,1.13)	1.48(1.37,1.61)
**Lung**	**amoxicillin**	4,019(50.6%)	13,729(41.5%)	1.29(1.21,1.37)	<0.001	2	1.22(1.13,1.31)	1.45(1.33,1.58)
**Lung**	**azathioprine**	59(0.7%)	144(0.4%)	1.76(1.21,2.57)	0.003	11	1.10(0.61,1.99)	2.50(1.54,4.05)
**Lung**	**cimetidine**	786(9.9%)	2,312(7.0%)	1.35(1.22,1.50)	<0.001	4	1.33(1.15,1.53)	1.38(1.20,1.59)
**Lung**	**ipratropium***	992(12.5%)	1,449(4.4%)	1.43(1.27,1.61)	<0.001	8	1.21(1.04,1.41)	1.69(1.45,1.97)
**Lung**	**nitrazepam**	206(2.6%)	511(1.5%)	1.34(1.09,1.65)	0.005	10	1.04(0.77,1.41)	1.66(1.27,2.19)
**Lung**	**salbutamol***	2,148(27.0%)	4,807(14.5%)	1.35(1.24,1.47)	<0.001	9	1.28(1.16,1.42)	1.44(1.29,1.60)
**Lung**	**salmeterol***	858(10.8%)	1,570(4.7%)	1.44(1.28,1.62)	<0.001	12	1.37(1.17,1.60)	1.52(1.31,1.78)
**Lung**	**tiotropium***	512(6.4%)	587(1.8%)	1.63(1.39,1.91)	<0.001	7	1.60(1.30,1.96)	1.66(1.34,2.06)
**Colorectal**	**allopurinol**	244(3.6%)	787(2.7%)	1.26(1.08,1.48)	0.004	17	1.18(0.93,1.50)	1.33(1.08,1.64)
**Prostate §§**	**alfuzosin**	212(3.5%)	451(1.8%)	1.66(1.37,2.01)	<0.001	5	1.65(1.27,2.16)	1.67(1.28,2.18)
**Prostate §§**	**clioquinol***	228(3.8%)	771(3.1%)	1.34(1.13,1.58)	0.001	1	1.29(1.04,1.59)	1.42(1.09,1.85)
**Bladder**	**cefalexin**	331(12.7%)	994(9.3%)	1.37(1.17,1.60)	<0.001	1	1.36(1.12,1.65)	1.39(1.10,1.75)
**Bladder**	**ciprofloxacin**	260(10.0%)	784(7.3%)	1.40(1.18,1.65)	<0.001	1	1.35(1.11,1.66)	1.47(1.14,1.91)
**Bladder**	**nicotine**	147(5.6%)	354(3.3%)	1.54(1.23,1.94)	<0.001	2	1.29(0.97,1.70)	2.13(1.50,3.03)
**Bladder**	**phenoxymethylpenicillin**	186(7.1%)	652(6.1%)	1.30(1.08,1.56)	0.006	1	1.22(1.00,1.50)	1.65(1.12,2.42)
**Bladder**	**quinine**	206(7.9%)	591(5.5%)	1.28(1.06,1.54)	0.009	4	1.14(0.88,1.47)	1.44(1.12,1.86)
**Bladder**	**ranitidine**	392(15.0%)	1,252(11.7%)	1.26(1.10,1.44)	0.001	6	1.20(1.00,1.44)	1.32(1.09,1.59)
**Bladder**	**trimethoprim**	506(19.4%)	1,246(11.6%)	1.92(1.68,2.20)	<0.001	1	1.78(1.51,2.09)	2.16(1.79,2.62)
**Malignant melanoma**	**clopidogrel**	51(2.7%)	153(1.8%)	1.63(1.13,2.36)	0.009	6	1.36(0.80,2.33)	1.89(1.18,3.03)
**Oesophagus**	**alginic acid**	287(15.0%)	844(10.5%)	1.38(1.17,1.63)	<0.001	5	1.23(0.98,1.54)	1.57(1.25,1.96)
**Oesophagus**	**azathioprine**	20(1.0%)	30(0.4%)	3.39(1.79,6.42)	<0.001	9	2.69(1.12,6.47)	4.45(1.74,11.41)
**Oesophagus**	**ipratropium***	125(6.6%)	305(3.8%)	1.45(1.11,1.89)	0.006	8	1.40(1.00,1.95)	1.52(1.04,2.22)
**Oesophagus**	**lansoprazole**	323(16.9%)	1,069(13.3%)	1.33(1.14,1.55)	<0.001	4	1.08(0.87,1.34)	1.61(1.32,1.97)
**Oesophagus**	**nystatin**	83(4.4%)	213(2.7%)	1.65(1.23,2.20)	0.001	1	1.51(1.06,2.16)	1.91(1.21,3.03)
**Oesophagus**	**nystatin***	173(9.1%)	570(7.1%)	1.36(1.12,1.67)	0.002	1	1.35(1.04,1.74)	1.39(1.03,1.87)
**Oesophagus**	**omeprazole**	426(22.3%)	1,428(17.8%)	1.29(1.12,1.48)	<0.001	6	1.14(0.95,1.37)	1.46(1.21,1.75)
**Non-Hodgkin's lymphoma**	**betamethasone***	307(18.9%)	1,057(15.0%)	1.26(1.08,1.48)	0.004	1	1.24(1.01,1.52)	1.29(1.05,1.59)
**Non-Hodgkin's lymphoma**	**clobetasol***	51(3.1%)	132(1.9%)	1.72(1.20,2.46)	0.003	1	1.63(0.99,2.69)	1.82(1.10,3.02)
**Non-Hodgkin's lymphoma**	**erythromycin**	220(13.6%)	804(11.4%)	1.28(1.07,1.54)	0.008	1	1.16(0.93,1.45)	1.50(1.15,1.97)
**Leukaemia**	**oxybutynin**	33(2.3%)	81(1.3%)	1.93(1.21,3.09)	0.006	4	1.23(0.59,2.57)	2.74(1.47,5.10)

Table S2 (cont)

Cancer	Medicine	No. (%) cases	No. (%) controls	OR (95%CI)	p-value	Median ††	Low use v non-use: OR (95%CI)	High use v non-use: OR (95%CI)
**Oral (inc. Head,neck & nasal)**	**clobetasone***	124(10.5%)	440(8.5%)	1.41(1.10,1.81)	0.007	1	1.14(0.83,1.57)	1.90(1.33,2.70)
**Oral (inc. Head,neck & nasal)**	**nystatin**	61(5.2%)	129(2.5%)	1.92(1.33,2.77)	0.001	1	1.70(1.09,2.66)	2.44(1.32,4.51)
**Oral (inc. Head,neck & nasal)**	**vitamin B**	191(16.2%)	260(5.0%)	2.33(1.82,3.00)	<0.001	4	2.01(1.44,2.82)	2.72(1.93,3.84)
**Ovary §**	**phenytoin**	11(1.0%)	19(0.4%)	3.86(1.68,8.89)	0.002	27	3.21(1.02,10.08)	4.81(1.40,16.51)
**Stomach**	**codeine**	425(37.2%)	1,509(31.0%)	1.35(1.15,1.59)	<0.001	3	1.34(1.11,1.63)	1.37(1.11,1.69)
**Stomach**	**nicotinates***	33(2.9%)	83(1.7%)	2.09(1.29,3.39)	0.003	1	1.95(1.06,3.56)	2.33(1.13,4.79)
**Stomach**	**vitamin B**	123(10.8%)	247(5.1%)	1.55(1.18,2.03)	0.002	5	1.45(1.02,2.07)	1.67(1.15,2.44)
**Kidney**	**hydrochlorothiazide**	42(3.8%)	80(1.7%)	2.21(1.45,3.37)	<0.001	13	1.82(1.02,3.25)	2.73(1.51,4.97)
**Kidney**	**perindopril**	62(5.6%)	122(2.6%)	1.76(1.21,2.54)	0.003	10	1.56(0.93,2.61)	1.97(1.20,3.25)
**Pancreas**	**amoxicillin**	429(47.5%)	1,561(41.3%)	1.31(1.09,1.56)	0.003	2	1.31(1.08,1.58)	1.31(1.00,1.71)
**Uterus §**	**chlortalidone**	32(3.8%)	68(1.8%)	2.14(1.32,3.49)	0.002	14	2.06(1.01,4.17)	2.22(1.18,4.16)
**Uterus §**	**enalapril**	57(6.8%)	129(3.3%)	2.00(1.38,2.92)	<0.001	14	1.34(0.75,2.41)	2.67(1.65,4.31)
**Brain and CNS**	**zinc oxide***	28(4.9%)	63(2.5%)	1.97(1.19,3.27)	0.008	1	1.95(1.01,3.77)	2.00(0.96,4.16)
**Larynx**	**dextropropoxyphene**	127(22.1%)	363(14.4%)	1.54(1.14,2.08)	0.004	4	1.44(0.96,2.16)	1.63(1.12,2.38)
**Larynx**	**paracetamol**	324(56.3%)	1,088(43.2%)	1.40(1.10,1.79)	0.007	5	1.39(1.05,1.84)	1.43(1.06,1.92)
**Larynx**	**vitamin B**	74(12.9%)	117(4.6%)	1.80(1.21,2.68)	0.004	6	1.74(1.05,2.90)	1.87(1.08,3.25)
**Myeloma**	**amoxicillin**	202(46.8%)	755(40.3%)	1.44(1.11,1.86)	0.006	2	1.36(1.02,1.80)	1.66(1.14,2.41)
**Myeloma**	**clioquinol***	21(4.9%)	46(2.5%)	2.49(1.30,4.75)	0.006	1	2.19(0.96,4.98)	3.02(1.13,8.03)
**Myeloma**	**paracetamol**	245(56.7%)	877(46.8%)	1.51(1.16,1.96)	0.002	6	1.51(1.12,2.02)	1.51(1.09,2.09)
**Thyroid**	**clarithromycin**	31(11.4%)	98(7.8%)	2.10(1.28,3.46)	0.003	1	1.62(0.88,2.96)	3.49(1.63,7.49)
**Thyroid**	**flucloxacillin**	60(22.1%)	194(15.4%)	1.70(1.16,2.49)	0.006	1	1.60(1.03,2.50)	1.92(1.07,3.44)
**Thyroid**	**folic acid§**	13(6.5%)	34(3.6%)	2.77(1.32,5.82)	0.007	1	2.41(0.93,6.27)	3.41(1.08,10.83)
**Thyroid**	**gabapentin**	7(2.6%)	7(0.6%)	5.84(1.82,18.79)	0.003	2	4.84(1.12,20.87)	8.16(1.14,58.61)
**Thyroid**	**levothyroxine**	28(10.3%)	64(5.1%)	2.21(1.27,3.83)	0.005	18	2.18(0.97,4.90)	2.23(1.12,4.44)
**Thyroid**	**mefenamic acid**	19(7.0%)	58(4.6%)	2.30(1.25,4.25)	0.008	1	1.81(0.86,3.80)	3.78(1.44,9.88)
**Anal**	**clonidine**	8(3.5%)	11(1.1%)	4.49(1.61,12.50)	0.004	2	3.97(1.26,12.54)	7.32(0.72,74.27)
**Anal**	**doxazosin**	17(7.5%)	35(3.6%)	2.76(1.34,5.71)	0.006	11	1.99(0.65,6.11)	3.37(1.40,8.11)
**Anal**	**theophylline**	6(2.6%)	7(0.7%)	9.08(1.78,46.43)	0.008	10	9.04(0.65,126.55)	9.11(1.15,71.84)
**Signals associated with decreased cancer risk**								
**Breast §**	**trazodone**	159(1.6%)	949(2.1%)	0.74(0.62,0.89)	0.001	3	0.75(0.60,0.96)	0.72(0.55,0.95)
**Lung**	**fluocinolone***	67(0.8%)	406(1.2%)	0.65(0.48,0.89)	0.007	2	0.70(0.48,1.02)	0.58(0.34,0.98)
**Colorectal**	**metoclopramide**	210(3.1%)	1,106(3.8%)	0.77(0.65,0.91)	0.002	1	0.91(0.74,1.11)	0.58(0.44,0.76)
**Colorectal**	**nitrofurantoin**	76(1.1%)	430(1.5%)	0.66(0.51,0.87)	0.003	1	0.70(0.51,0.97)	0.59(0.38,0.94)
**Prostate §§**	**levodopa**	33(0.6%)	187(0.8%)	0.44(0.26,0.75)	0.003	17	0.50(0.27,0.92)	0.36(0.17,0.76)
**Prostate §§**	**risperidone**	9(0.2%)	92(0.4%)	0.38(0.18,0.78)	0.009	8	0.43(0.17,1.12)	0.32(0.11,0.97)
**Prostate §§**	**senna**	254(4.3%)	1,150(4.7%)	0.77(0.66,0.90)	0.001	2	0.91(0.75,1.10)	0.61(0.48,0.78)
**Bladder**	**sotalol**	5(0.2%)	82(0.8%)	0.19(0.07,0.52)	0.001	13	0.19(0.05,0.82)	0.18(0.04,0.78)
**Malignant melanoma**	**gliclazide**	26(1.4%)	184(2.2%)	0.49(0.30,0.80)	0.005	16	0.64(0.35,1.16)	0.34(0.16,0.72)

Table S2 (cont)

Cancer	Medicine	No. (%) cases	No. (%) controls	OR (95%CI)	p-value	Median ††	Low use v non-use: OR (95%CI)	High use v non-use: OR (95%CI)
**Oesophagus**	**diazepam**	124(6.5%)	644(8.0%)	0.73(0.58,0.91)	0.005	2	0.78(0.60,1.03)	0.64(0.45,0.91)
**Oesophagus**	**estrogen (HRT) §**	63(10.4%)	463(17.9%)	0.61(0.44,0.84)	0.003	8	0.62(0.41,0.95)	0.59(0.38,0.91)
**Non-Hodgkin's lymphoma**	**progestogen (cont) §**	34(4.0%)	263(7.1%)	0.49(0.30,0.81)	0.005	4	0.56(0.32,0.99)	0.39(0.19,0.82)
**Oral (inc. Head,neck & nasal)**	**doxycycline**	28(2.4%)	243(4.7%)	0.43(0.28,0.67)	<0.001	1	0.49(0.29,0.81)	0.32(0.14,0.74)
**Ovary §**	**progestogen (cont)**	46(4.1%)	368(7.2%)	0.56(0.38,0.81)	0.003	3	0.63(0.39,1.01)	0.48(0.28,0.81)
**Cervix & other gynae §**	**terbinafine**	4(0.5%)	86(2.3%)	0.20(0.07,0.54)	0.002	2	0.25(0.09,0.71)	-
**Uterus §**	**progestogen (cont)**	12(1.4%)	158(4.1%)	0.37(0.19,0.71)	0.003	3	0.41(0.18,0.94)	0.33(0.12,0.85)

† comorbidities include diabetes, myocardial infarction, coronary heart disease, heart failure, peripheral vascular disease, dementia, cerebrovascular disease, chronic obstructive pulmonary disease, osteoporosis, rheumatological disease, renal disease, liver disease, irritable bowel disease, human immunodeficiency viruses and hemiplegia/paraplegia, plus potential site-specific confounders; OR odds ratio; CI confidence interval; CNS: central nervous system; medicines systemic unless otherwise indicated* (local); HRT: hormone replacement therapy; § analyses restricted to females only; §§ analyses restricted to males only; low use: patient received more than I item of medicine and <=median number of items; high use: patient received > median number of items of medicine; †† among control users; - no cases with prescription in category

Table S3: Comorbidity† adjusted signals: exposure >=6 prescriptions

Cancer	Medicine	No. (%) cases	No. (%) controls	OR (95%CI)	p-value	Median ††	Low use v non-use: OR (95%CI)	High use v non-use: OR (95%CI)
**Signals associated with increased cancer risk**								
**Breast §**	**estrogen-HRT**	1,557(12.7%)	6,040(10.6%)	1.36(1.27,1.45)	<0.001	7	1.15(1.07,1.23)	1.41(1.30,1.52)
**Breast §**	**metronidazole***	16(0.1%)	37(0.1%)	2.21(1.21,4.03)	0.010	1	1.24(1.01,1.52)	1.29(0.98,1.69)
**Breast §**	**progestogen-HRT**	1,211(9.9%)	4,226(7.4%)	1.50(1.40,1.62)	<0.001	4	1.07(1.00,1.16)	1.51(1.40,1.62)
**Lung**	**amitriptyline**	440(4.6%)	1,415(3.4%)	1.27(1.13,1.43)	<0.001	3	1.15(1.03,1.28)	1.28(1.14,1.43)
**Lung**	**amoxicillin**	752(7.9%)	1,690(4.1%)	1.44(1.30,1.60)	<0.001	2	1.32(1.24,1.40)	1.62(1.50,1.75)
**Lung**	**azathioprine**	50(0.5%)	105(0.3%)	1.88(1.30,2.70)	0.001	11	1.08(0.66,1.75)	2.09(1.39,3.14)
**Lung**	**cimetidine**	423(4.5%)	1,184(2.9%)	1.46(1.29,1.66)	<0.001	4	1.30(1.16,1.47)	1.48(1.31,1.66)
**Lung**	**diazepam**	286(3.0%)	841(2.0%)	1.29(1.11,1.50)	0.001	2	1.16(1.04,1.29)	1.22(1.08,1.39)
**Lung**	**dihydrocodeine**	720(7.6%)	2,007(4.8%)	1.40(1.27,1.54)	<0.001	2	1.22(1.12,1.33)	1.37(1.25,1.49)
**Lung**	**folic acid §**	139(3.3%)	361(1.9%)	1.50(1.21,1.86)	<0.001	6	1.37(1.11,1.70)	1.64(1.31,2.06)
**Lung**	**ipratropium***	713(7.5%)	942(2.3%)	1.63(1.45,1.84)	<0.001	8	1.40(1.23,1.59)	1.77(1.56,2.02)
**Lung**	**nitrazepam**	157(1.7%)	360(0.9%)	1.49(1.21,1.84)	<0.001	10	1.17(0.91,1.50)	1.52(1.22,1.91)
**Lung**	**quinine**	417(4.4%)	1,061(2.6%)	1.25(1.10,1.42)	0.001	5	1.00(0.87,1.14)	1.25(1.10,1.42)
**Lung**	**salbutamol***	1,592(16.8%)	3,199(7.7%)	1.41(1.30,1.53)	<0.001	9	1.37(1.26,1.50)	1.52(1.39,1.66)
**Lung**	**salmeterol***	659(6.9%)	1,175(2.8%)	1.37(1.22,1.53)	<0.001	12	1.30(1.14,1.49)	1.37(1.20,1.57)
**Lung**	**temazepam**	310(3.3%)	835(2.0%)	1.33(1.15,1.54)	<0.001	3	1.08(0.93,1.25)	1.33(1.16,1.53)
**Lung**	**tiotropium***	297(3.1%)	335(0.8%)	1.64(1.37,1.97)	<0.001	7	1.73(1.43,2.09)	1.78(1.46,2.16)
**Lung**	**vitamin D***	76(0.8%)	233(0.6%)	1.45(1.10,1.91)	0.009	3	0.89(0.69,1.15)	1.39(1.09,1.77)
**Colorectal**	**allopurinol**	250(2.9%)	743(2.0%)	1.35(1.16,1.57)	<0.001	17	1.07(0.87,1.32)	1.48(1.23,1.78)
**Colorectal**	**aminophylline**	43(0.5%)	91(0.2%)	1.93(1.31,2.83)	0.001	10	1.10(0.65,1.86)	1.91(1.24,2.95)
**Colorectal**	**bisacodyl**	46(0.5%)	96(0.3%)	1.79(1.24,2.59)	0.002	2	0.84(0.59,1.19)	1.70(1.23,2.35)
**Colorectal**	**dipyridamole**	137(1.6%)	390(1.0%)	1.45(1.17,1.80)	0.001	10	1.12(0.87,1.43)	1.41(1.11,1.80)
**Colorectal**	**mesalazine**	57(0.7%)	135(0.4%)	1.65(1.18,2.30)	0.003	10	0.91(0.57,1.44)	1.80(1.24,2.60)
**Colorectal**	**perindopril**	186(2.1%)	588(1.6%)	1.31(1.10,1.56)	0.003	11	1.17(0.95,1.45)	1.22(0.99,1.51)
**Colorectal**	**phenytoin**	46(0.5%)	147(0.4%)	1.58(1.12,2.22)	0.009	25	1.16(0.72,1.85)	1.45(0.94,2.26)
**Prostate §§**	**cerivastatin**	60(0.8%)	166(0.5%)	1.59(1.16,2.20)	0.004	8	1.17(0.80,1.73)	1.74(1.21,2.50)
**Prostate §§**	**clioquinol***	24(0.3%)	58(0.2%)	2.06(1.24,3.42)	0.005	1	1.23(1.01,1.49)	1.43(1.12,1.83)
**Bladder**	**amoxicillin**	153(4.7%)	448(3.2%)	1.39(1.13,1.72)	0.002	2	1.15(1.04,1.27)	1.50(1.31,1.72)
**Bladder**	**nicotine**	17(0.5%)	30(0.2%)	2.59(1.38,4.85)	0.003	2	1.76(1.35,2.28)	2.65(1.90,3.70)
**Bladder**	**trimethoprim**	49(1.5%)	116(0.8%)	1.80(1.25,2.58)	0.001	1	1.81(1.56,2.09)	2.22(1.87,2.65)
**Malignant melanoma**	**clopidogrel**	34(1.4%)	85(0.8%)	1.86(1.21,2.88)	0.005	6	1.31(0.79,2.18)	1.89(1.21,2.96)
**Oesophagus**	**alginic acid**	184(7.7%)	482(4.6%)	1.52(1.26,1.84)	<0.001	5	1.26(1.03,1.54)	1.56(1.29,1.89)
**Oesophagus**	**beclometasone***	186(7.7%)	561(5.4%)	1.37(1.14,1.65)	0.001	4	0.84(0.68,1.03)	1.29(1.07,1.55)
**Oesophagus**	**cisapride**	15(0.6%)	22(0.2%)	2.89(1.41,5.92)	0.004	3	2.00(1.04,3.84)	2.07(1.06,4.03)
**Oesophagus**	**lansoprazole**	198(8.2%)	519(5.0%)	1.67(1.39,2.01)	<0.001	4	1.10(0.91,1.33)	1.65(1.38,1.98)
**Oesophagus**	**omeprazole**	276(11.5%)	812(7.8%)	1.45(1.24,1.69)	<0.001	6	1.26(1.06,1.48)	1.54(1.31,1.81)
**Oesophagus**	**risedronate sodium**	16(0.7%)	31(0.3%)	2.54(1.27,5.05)	0.008	7	0.66(0.24,1.81)	3.29(1.61,6.75)

Table S3 (cont)

Cancer	Medicine	No. (%) cases	No. (%) controls	OR (95%CI)	p-value	Median ††	Low use v non-use: OR (95%CI)	High use v non-use: OR (95%CI)
Oesophagus	vitamin B	91(3.8%)	229(2.2%)	1.54(1.18,2.01)	0.001	5	1.20(0.92,1.56)	1.56(1.20,2.04)
Oesophagus	warfarin	103(4.3%)	283(2.7%)	1.43(1.11,1.84)	0.005	13	0.95(0.69,1.31)	1.56(1.17,2.08)
Non-Hodgkin's lymphoma	amoxicillin	86(4.1%)	277(2.9%)	1.46(1.12,1.92)	0.006	2	1.28(1.13,1.45)	1.55(1.29,1.85)
Non-Hodgkin's lymphoma	azathioprine	19(0.9%)	27(0.3%)	2.58(1.31,5.08)	0.006	15	1.46(0.60,3.53)	2.87(1.31,6.29)
Non-Hodgkin's lymphoma	gabapentin	16(0.8%)	27(0.3%)	2.86(1.47,5.58)	0.002	3	0.82(0.36,1.87)	2.73(1.48,5.06)
Non-Hodgkin's lymphoma	methotrexate	14(0.7%)	18(0.2%)	3.41(1.57,7.41)	0.002	11	2.74(1.11,6.78)	3.12(1.28,7.56)
Non-Hodgkin's lymphoma	phenoxymethylpenicillin	10(0.5%)	10(0.1%)	3.77(1.51,9.45)	0.005	1	1.20(0.96,1.48)	1.42(0.99,2.04)
Leukaemia	allopurinol	64(3.2%)	166(1.9%)	1.68(1.24,2.27)	0.001	14	1.38(0.93,2.05)	1.75(1.22,2.52)
Leukaemia	oxybutynin	25(1.3%)	46(0.5%)	2.35(1.41,3.92)	0.001	4	1.14(0.65,2.01)	2.19(1.33,3.61)
Leukaemia	oxytetracycline	19(1.0%)	34(0.4%)	2.67(1.49,4.79)	0.001	1	1.10(0.78,1.56)	1.47(0.99,2.19)
Leukaemia	paracetamol	459(23.0%)	1,605(18.2%)	1.26(1.10,1.44)	0.001	5	1.04(0.91,1.19)	1.27(1.10,1.47)
Oral (inc. Head,neck & nasal)	betamethasone*	42(2.9%)	116(1.7%)	1.69(1.15,2.48)	0.007	1	1.02(0.81,1.29)	1.37(1.09,1.72)
Oral (inc. Head,neck & nasal)	folic acid §	31(6.7%)	32(1.5%)	3.79(2.09,6.89)	<0.001	4	1.50(0.79,2.82)	4.03(2.27,7.17)
Oral (inc. Head,neck & nasal)	vitamin A	15(1.0%)	12(0.2%)	3.84(1.68,8.79)	0.001	1	1.07(0.49,2.33)	2.28(1.21,4.28)
Oral (inc. Head,neck & nasal)	vitamin B	121(8.2%)	132(2.0%)	3.09(2.31,4.14)	<0.001	4	2.31(1.72,3.11)	3.23(2.43,4.29)
Oral (inc. Head,neck & nasal)	vitamin C	18(1.2%)	14(0.2%)	4.43(2.05,9.56)	<0.001	2	1.21(0.62,2.34)	2.54(1.31,4.92)
Stomach	cimetidine	63(4.4%)	180(2.9%)	1.52(1.11,2.07)	0.008	4	1.32(0.98,1.79)	1.56(1.15,2.11)
Ovary §	cyclopenthiazide	13(0.9%)	17(0.3%)	3.31(1.54,7.15)	0.002	12	1.91(0.69,5.33)	3.26(1.20,8.88)
Ovary §	phenytoin	13(0.9%)	19(0.3%)	3.87(1.83,8.18)	<0.001	27	2.48(0.97,6.31)	4.67(1.62,13.46)
Ovary §	vitamin D*	13(0.9%)	24(0.4%)	2.66(1.30,5.45)	0.007	2	0.87(0.42,1.82)	2.53(1.44,4.44)
Kidney	hydrochlorothiazide	39(2.9%)	70(1.2%)	2.26(1.46,3.48)	<0.001	13	1.50(0.86,2.62)	2.50(1.45,4.31)
Kidney	nystatin*	19(1.4%)	34(0.6%)	2.25(1.23,4.13)	0.009	1	0.90(0.66,1.24)	1.22(0.84,1.78)
Kidney	perindopril	45(3.3%)	84(1.4%)	1.87(1.25,2.81)	0.003	10	1.46(0.92,2.32)	1.85(1.15,2.97)
Pancreas	clopidogrel	35(3.3%)	66(1.4%)	2.14(1.34,3.42)	0.001	7	1.05(0.60,1.83)	2.22(1.35,3.66)
Pancreas	hydrochlorothiazide	31(2.9%)	79(1.7%)	1.84(1.16,2.90)	0.009	15	1.26(0.69,2.31)	1.78(1.01,3.15)
Pancreas	metoclopramide	12(1.1%)	16(0.3%)	3.08(1.40,6.76)	0.005	1	1.01(0.62,1.64)	1.85(1.14,3.03)
Pancreas	nicotine	11(1.0%)	13(0.3%)	3.61(1.55,8.43)	0.003	2	1.91(1.24,2.94)	1.97(1.18,3.28)
Cervix & other gynae §	cimetidine	28(2.7%)	68(1.4%)	1.87(1.17,2.98)	0.009	3	1.33(0.89,2.00)	1.61(1.05,2.48)
Uterus §	atenolol	186(18.4%)	610(13.0%)	1.40(1.14,1.71)	0.001	17	1.26(0.99,1.60)	1.54(1.21,1.96)
Uterus §	bendroflumethiazide	212(21.0%)	756(16.1%)	1.33(1.10,1.61)	0.003	14	1.08(0.86,1.34)	1.53(1.22,1.90)
Uterus §	bismuth*	5(0.5%)	4(0.1%)	6.56(1.64,26.17)	0.008	1	0.65(0.36,1.17)	2.37(1.16,4.83)
Uterus §	dextropropoxyphene	103(10.2%)	341(7.3%)	1.43(1.11,1.83)	0.005	3	1.04(0.81,1.34)	1.23(0.96,1.58)
Uterus §	doxazosin	35(3.5%)	72(1.5%)	2.21(1.43,3.43)	<0.001	9	1.24(0.73,2.13)	2.23(1.37,3.64)
Uterus §	enalapril	45(4.5%)	108(2.3%)	1.78(1.21,2.62)	0.003	14	1.16(0.69,1.94)	2.23(1.43,3.47)
Uterus §	losartan potassium	28(2.8%)	66(1.4%)	2.00(1.24,3.23)	0.005	13	1.26(0.68,2.35)	2.05(1.17,3.62)
Uterus §	peru balsam*	5(0.5%)	4(0.1%)	6.56(1.64,26.17)	0.008	1	0.60(0.33,1.10)	2.36(1.16,4.82)
Uterus §	salicylic acid*	7(0.7%)	9(0.2%)	4.31(1.53,12.08)	0.006	1	0.75(0.41,1.38)	1.83(1.02,3.27)
Larynx	cimetidine	38(5.1%)	79(2.4%)	2.12(1.36,3.32)	0.001	4	0.71(0.42,1.20)	1.97(1.27,3.05)

Table S3 (cont)

Cancer	Medicine	No. (%) cases	No. (%) controls	OR (95%CI)	p-value	Median ††	Low use v non-use: OR (95%CI)	High use v non-use: OR (95%CI)
**Larynx**	**vitamin B**	45(6.1%)	71(2.1%)	2.20(1.41,3.41)	<0.001	6	2.19(1.40,3.40)	2.66(1.68,4.21)
**Brain and CNS**	**carbamazepine**	16(2.2%)	31(0.9%)	3.02(1.59,5.71)	0.001	6	1.43(0.69,2.97)	3.37(1.72,6.62)
**Myeloma**	**mometasone***	9(1.7%)	14(0.6%)	3.45(1.40,8.50)	0.007	1	1.25(0.71,2.21)	1.78(1.01,3.15)
**Liver**	**furosemide**	96(18.2%)	166(7.1%)	1.98(1.35,2.91)	0.001	12	1.14(0.73,1.78)	1.89(1.22,2.94)
**Liver**	**mometasone***	10(1.9%)	22(0.9%)	3.82(1.42,10.26)	0.008	1	1.15(0.59,2.26)	1.75(0.88,3.44)
**Liver**	**pravastatin**	24(4.5%)	47(2.0%)	2.26(1.24,4.11)	0.008	12	1.15(0.45,2.97)	2.66(1.31,5.38)
**Thyroid**	**levothyroxine**	30(8.8%)	69(4.3%)	2.30(1.40,3.76)	0.001	18	1.57(0.79,3.13)	2.87(1.57,5.24)
**Thyroid**	**progestogen-HRT §**	18(7.1%)	47(3.9%)	2.52(1.33,4.78)	0.005	3	1.18(0.70,2.00)	2.19(1.22,3.94)
**Anal**	**doxazosin**	13(4.7%)	23(1.8%)	3.19(1.44,7.06)	0.004	11	1.68(0.62,4.57)	3.37(1.42,7.97)
**Anal**	**nicotine**	5(1.8%)	3(0.2%)	9.95(2.22,44.53)	0.003	2	1.21(0.54,2.70)	2.74(1.11,6.75)
**Signals associated with decreased cancer risk**								
**Breast §**	**carbamazepine**	78(0.6%)	494(0.9%)	0.70(0.55,0.90)	0.005	5	1.11(0.91,1.35)	0.70(0.55,0.90)
**Breast §**	**iron**	204(1.7%)	1,024(1.8%)	0.78(0.67,0.92)	0.003	2	0.96(0.87,1.06)	0.84(0.74,0.94)
**Breast §**	**prednisolone**	176(1.4%)	1,008(1.8%)	0.78(0.66,0.92)	0.004	2	1.02(0.92,1.13)	0.85(0.75,0.97)
**Breast §**	**trimethoprim**	91(0.7%)	541(0.9%)	0.72(0.57,0.91)	0.005	1	1.03(0.96,1.11)	0.94(0.85,1.02)
**Lung**	**benzalkonium***	18(0.2%)	118(0.3%)	0.49(0.29,0.83)	0.008	1	1.20(0.98,1.48)	0.71(0.54,0.94)
**Lung**	**dimeticone***	17(0.2%)	119(0.3%)	0.46(0.27,0.80)	0.005	1	1.15(0.93,1.42)	0.75(0.57,0.98)
**Lung**	**levodopa**	21(0.2%)	193(0.5%)	0.38(0.24,0.61)	<0.001	19	0.66(0.41,1.05)	0.34(0.18,0.64)
**Colorectal**	**diclofenac**	336(3.9%)	1,986(5.2%)	0.71(0.63,0.80)	<0.001	2	0.97(0.90,1.06)	0.84(0.76,0.92)
**Colorectal**	**lactulose**	263(3.0%)	1,179(3.1%)	0.78(0.68,0.90)	0.001	3	1.03(0.92,1.15)	0.81(0.71,0.92)
**Colorectal**	**misoprostol**	77(0.9%)	453(1.2%)	0.69(0.53,0.88)	0.003	2	0.84(0.70,1.00)	0.81(0.67,0.98)
**Colorectal**	**naproxen**	97(1.1%)	572(1.5%)	0.72(0.57,0.89)	0.003	2	0.89(0.76,1.03)	0.78(0.66,0.94)
**Colorectal**	**senna**	126(1.5%)	591(1.6%)	0.73(0.60,0.90)	0.003	2	0.97(0.83,1.13)	0.72(0.61,0.86)
**Prostate §§**	**benzalkonium***	8(0.1%)	77(0.2%)	0.36(0.17,0.77)	0.009	1	0.95(0.72,1.25)	0.75(0.53,1.07)
**Prostate §§**	**bumetanide**	32(0.4%)	201(0.6%)	0.57(0.38,0.86)	0.007	12	1.05(0.71,1.56)	0.43(0.26,0.73)
**Prostate §§**	**citalopram**	49(0.7%)	352(1.1%)	0.59(0.43,0.81)	0.001	5	1.03(0.81,1.30)	0.59(0.43,0.81)
**Prostate §§**	**dimeticone***	8(0.1%)	79(0.2%)	0.36(0.17,0.77)	0.009	1	0.97(0.73,1.28)	0.72(0.50,1.02)
**Prostate §§**	**furosemide**	435(5.8%)	2,181(6.8%)	0.70(0.61,0.79)	<0.001	12	0.91(0.80,1.03)	0.69(0.59,0.79)
**Prostate §§**	**iron**	92(1.2%)	424(1.3%)	0.72(0.57,0.92)	0.008	3	1.08(0.92,1.27)	0.72(0.58,0.89)
**Prostate §§**	**lactulose**	172(2.3%)	819(2.6%)	0.68(0.57,0.81)	<0.001	2	0.93(0.81,1.06)	0.70(0.60,0.82)
**Prostate §§**	**lidocaine***	8(0.1%)	68(0.2%)	0.35(0.16,0.75)	0.007	1	1.15(0.86,1.55)	0.69(0.47,1.00)
**Prostate §§**	**risperidone**	5(0.1%)	67(0.2%)	0.27(0.11,0.70)	0.007	8	0.40(0.18,0.89)	0.35(0.13,0.91)
**Prostate §§**	**senna**	84(1.1%)	415(1.3%)	0.70(0.55,0.90)	0.005	2	0.87(0.73,1.04)	0.63(0.51,0.78)
**Prostate §§**	**vitamin D**	69(0.9%)	378(1.2%)	0.66(0.50,0.88)	0.004	6	1.02(0.80,1.30)	0.69(0.52,0.93)
**Bladder**	**iron**	55(1.7%)	263(1.9%)	0.64(0.47,0.88)	0.006	2	0.84(0.66,1.06)	0.70(0.55,0.89)
**Bladder**	**lactulose**	108(3.3%)	465(3.3%)	0.73(0.58,0.91)	0.006	2	1.01(0.84,1.21)	0.83(0.69,1.01)

Table S3 (cont)

Cancer	Medicine	No. (%) cases	No. (%) controls	OR (95%CI)	p-value	Median ††	Low use v non-use: OR (95%CI)	High use v non-use: OR (95%CI)
**Malignant melanoma**	**gliclazide**	22(0.9%)	153(1.4%)	0.51(0.31,0.84)	0.009	16	0.57(0.33,1.00)	0.44(0.23,0.85)
**Non-Hodgkin's lymphoma**	**vitamin B**	29(1.4%)	179(1.9%)	0.51(0.34,0.78)	0.002	5	1.06(0.78,1.43)	0.52(0.34,0.79)
**Stomach**	**finasteride §§**	11(1.3%)	89(2.5%)	0.41(0.21,0.81)	0.010	10	0.74(0.40,1.36)	0.36(0.16,0.80)
**Ovary §**	**nifedipine**	32(2.3%)	216(3.3%)	0.56(0.38,0.83)	0.004	11	0.71(0.48,1.07)	0.64(0.41,0.99)
**Ovary §**	**progestogen-contraceptive**	13(0.9%)	140(2.1%)	0.44(0.23,0.81)	0.009	3	0.64(0.42,0.98)	0.45(0.27,0.74)
**Uterus §**	**estrogen-HRT**	93(9.2%)	650(13.8%)	0.68(0.53,0.87)	0.002	7	0.76(0.59,0.98)	0.70(0.54,0.92)
**Larynx**	**diclofenac**	20(2.7%)	153(4.6%)	0.48(0.29,0.78)	0.003	2	1.12(0.84,1.49)	0.71(0.50,1.00)
**Larynx**	**doxazosin**	8(1.1%)	63(1.9%)	0.31(0.14,0.71)	0.006	14	0.49(0.22,1.12)	0.24(0.09,0.69)

† comorbidities include diabetes, myocardial infarction, coronary heart disease, heart failure, peripheral vascular disease, dementia, cerebrovascular disease, chronic obstructive pulmonary disease, osteoporosis, rheumatological disease, renal disease, liver disease, irritable bowel disease, human immunodeficiency viruses and hemiplegia/paraplegia, plus potential site-specific confounders; OR odds ratio; CI confidence interval; CNS: central nervous system; medicines systemic unless otherwise indicated* (local); HRT: hormone replacement therapy; § analyses restricted to females only; §§ analyses restricted to males only; low use: patient received more than I item of medicine and <=median number of items; high use: patient received > median number of items of medicine; †† among control users; - no cases with >=6 prescriptions in category

Table S4: Comorbidity† & smoking adjusted signals: exposure >=6 prescriptions

Cancer	Medicine	No. (%) cases	No. (%) controls	OR (95%CI)	p-value	Median ††	Low use v non-use: OR (95%CI)	High use v non-use: OR (95%CI)
**Signals associated with increased cancer risk**								
**Breast §**	**estrogen (HRT)**	1,368(13.9%)	5,344(11.9%)	1.32(1.23,1.43)	<0.001	7	1.13(1.05,1.23)	1.38(1.27,1.50)
**Breast §**	**metronidazole***	15(0.2%)	32(0.1%)	2.36(1.25,4.46)	0.008	1	1.25(1.00,1.56)	1.29(0.97,1.72)
**Breast §**	**progestogen (HRT)**	1,074(10.9%)	3,755(8.4%)	1.48(1.37,1.61)	<0.001	4	1.04(0.95,1.13)	1.48(1.37,1.61)
**Lung**	**amitriptyline**	418(5.3%)	1,261(3.8%)	1.25(1.09,1.44)	0.001	3	1.14(1.01,1.29)	1.25(1.10,1.42)
**Lung**	**amoxicillin**	722(9.1%)	1,632(4.9%)	1.36(1.21,1.53)	<0.001	2	1.22(1.13,1.31)	1.45(1.33,1.58)
**Lung**	**azathioprine**	45(0.6%)	92(0.3%)	2.30(1.49,3.56)	<0.001	11	1.10(0.61,1.99)	2.50(1.54,4.05)
**Lung**	**cimetidine**	376(4.7%)	1,005(3.0%)	1.37(1.18,1.58)	<0.001	4	1.33(1.15,1.53)	1.38(1.20,1.59)
**Lung**	**dihydrocodeine**	653(8.2%)	1,791(5.4%)	1.28(1.15,1.44)	<0.001	2	1.10(1.00,1.22)	1.20(1.09,1.33)
**Lung**	**fluticasone***	552(6.9%)	1,216(3.7%)	1.41(1.24,1.61)	<0.001	5	1.04(0.90,1.21)	1.42(1.24,1.62)
**Lung**	**ipratropium***	661(8.3%)	816(2.5%)	1.56(1.35,1.79)	<0.001	8	1.21(1.04,1.41)	1.69(1.45,1.97)
**Lung**	**nitrazepam**	139(1.7%)	284(0.9%)	1.58(1.22,2.05)	0.001	10	1.04(0.77,1.41)	1.66(1.27,2.19)
**Lung**	**phenoxymethylpenicillin**	21(0.3%)	44(0.1%)	2.89(1.52,5.50)	0.001	1	0.99(0.87,1.13)	1.23(0.99,1.54)
**Lung**	**quinine**	369(4.6%)	916(2.8%)	1.31(1.13,1.52)	<0.001	5	0.96(0.82,1.13)	1.30(1.12,1.52)
**Lung**	**salbutamol***	1,453(18.3%)	2,854(8.6%)	1.36(1.24,1.50)	<0.001	9	1.28(1.16,1.42)	1.44(1.29,1.60)
**Lung**	**salmeterol***	630(7.9%)	1,104(3.3%)	1.51(1.32,1.72)	<0.001	12	1.37(1.17,1.60)	1.52(1.31,1.78)
**Lung**	**tiotropium***	297(3.7%)	333(1.0%)	1.52(1.25,1.85)	<0.001	7	1.60(1.30,1.96)	1.66(1.34,2.06)
**Colorectal**	**allopurinol**	208(3.1%)	628(2.2%)	1.33(1.12,1.59)	0.001	17	1.18(0.93,1.50)	1.33(1.08,1.64)
**Colorectal**	**aminophylline**	30(0.4%)	72(0.2%)	1.89(1.17,3.05)	0.009	10	1.26(0.67,2.36)	1.75(1.03,2.96)
**Colorectal**	**dipyridamole**	116(1.7%)	336(1.2%)	1.44(1.13,1.83)	0.004	10	1.10(0.82,1.46)	1.40(1.06,1.83)
**Colorectal**	**tiotropium***	77(1.1%)	251(0.9%)	1.49(1.11,1.99)	0.007	7	1.24(0.90,1.71)	1.37(0.99,1.90)
**Prostate §§**	**alfuzosin**	103(1.7%)	222(0.9%)	1.63(1.25,2.13)	<0.001	5	1.65(1.27,2.16)	1.67(1.28,2.18)
**Prostate §§**	**atorvastatin**	452(7.6%)	1,783(7.3%)	1.25(1.11,1.42)	<0.001	17	1.15(0.98,1.33)	1.30(1.12,1.52)
**Prostate §§**	**clioquinol***	22(0.4%)	48(0.2%)	2.09(1.19,3.66)	0.010	1	1.29(1.04,1.59)	1.42(1.09,1.85)
**Bladder**	**amoxicillin**	146(5.6%)	424(4.0%)	1.45(1.16,1.81)	0.001	2	1.09(0.97,1.22)	1.39(1.20,1.61)
**Bladder**	**cefalexin**	30(1.2%)	61(0.6%)	1.99(1.23,3.20)	0.005	1	1.36(1.12,1.65)	1.39(1.10,1.75)
**Bladder**	**celecoxib**	41(1.6%)	115(1.1%)	1.78(1.20,2.63)	0.004	2	1.18(0.88,1.58)	1.53(1.11,2.10)
**Bladder**	**ranitidine**	204(7.8%)	601(5.6%)	1.28(1.07,1.54)	0.008	6	1.20(1.00,1.44)	1.32(1.09,1.59)
**Bladder**	**trimethoprim**	48(1.8%)	102(1.0%)	2.04(1.39,3.00)	<0.001	1	1.78(1.51,2.09)	2.16(1.79,2.62)
**Oesophagus**	**alginic acid**	152(8.0%)	402(5.0%)	1.53(1.22,1.91)	<0.001	5	1.23(0.98,1.54)	1.57(1.25,1.96)
**Oesophagus**	**azathioprine**	14(0.7%)	18(0.2%)	4.15(1.87,9.22)	<0.001	9	2.69(1.12,6.47)	4.45(1.74,11.41)
**Oesophagus**	**lansoprazole**	178(9.3%)	472(5.9%)	1.59(1.30,1.95)	<0.001	4	1.08(0.87,1.34)	1.61(1.32,1.97)
**Oesophagus**	**omeprazole**	242(12.7%)	719(9.0%)	1.40(1.17,1.66)	<0.001	6	1.14(0.95,1.37)	1.46(1.21,1.75)
**Oesophagus**	**prednisolone**	66(3.5%)	164(2.0%)	1.68(1.19,2.38)	0.003	2	1.09(0.84,1.42)	1.52(1.15,2.01)
**Oesophagus**	**risedronate sodium**	15(0.8%)	29(0.4%)	2.69(1.28,5.67)	0.009	7	0.45(0.13,1.58)	3.71(1.70,8.10)
**Non-Hodgkin's lymphoma**	**amoxicillin**	83(5.1%)	262(3.7%)	1.50(1.13,2.00)	0.006	2	1.18(1.02,1.36)	1.43(1.18,1.74)
**Non-Hodgkin's lymphoma**	**gabapentin**	15(0.9%)	27(0.4%)	3.41(1.67,6.96)	0.001	3	0.95(0.41,2.19)	3.08(1.61,5.91)

Table S4 (cont)

Cancer	Medicine	No. (%) cases	No. (%) controls	OR (95%CI)	p-value	Median ††	Low use v non-use: OR (95%CI)	High use v non-use: OR (95%CI)
**Leukaemia**	**metoclopramide**	16(1.1%)	34(0.5%)	2.61(1.34,5.11)	0.005	1	0.42(0.24,0.73)	1.13(0.69,1.85)
**Leukaemia**	**oxybutynin**	21(1.5%)	37(0.6%)	2.69(1.42,5.08)	0.002	4	1.23(0.59,2.57)	2.74(1.47,5.10)
**Leukaemia**	**oxytetracycline**	15(1.0%)	26(0.4%)	3.01(1.49,6.07)	0.002	1	1.29(0.87,1.92)	1.52(0.96,2.39)
**Leukaemia**	**paracetamol**	382(26.4%)	1,298(20.9%)	1.34(1.14,1.57)	<0.001	5	1.06(0.90,1.25)	1.37(1.15,1.63)
**Oral (inc. Head,neck & nasal)**	**hydrochlorothiazide**	17(1.4%)	44(0.9%)	2.56(1.34,4.89)	0.005	8	0.82(0.26,2.62)	3.34(1.68,6.64)
**Oral (inc. Head,neck & nasal)**	**hydrocortisone***	60(5.1%)	172(3.3%)	1.66(1.17,2.35)	0.004	2	0.92(0.76,1.13)	1.18(0.91,1.53)
**Oral (inc. Head,neck & nasal)**	**vitamin B**	104(8.8%)	107(2.1%)	2.55(1.79,3.63)	<0.001	4	2.01(1.44,2.82)	2.72(1.93,3.84)
**Oral (inc. Head,neck & nasal)**	**vitamin C**	15(1.3%)	11(0.2%)	3.63(1.42,9.25)	0.007	2	0.92(0.46,1.85)	2.49(1.09,5.70)
**Ovary §**	**phenytoin**	10(0.9%)	15(0.3%)	4.31(1.74,10.65)	0.002	27	3.21(1.02,10.08)	4.81(1.40,16.51)
**Stomach**	**chlorphenamine**	10(0.9%)	10(0.2%)	4.72(1.64,13.56)	0.004	1	1.32(0.83,2.12)	1.45(0.78,2.70)
**Kidney**	**hydrochlorothiazide**	37(3.3%)	62(1.3%)	2.61(1.63,4.18)	<0.001	13	1.82(1.02,3.25)	2.73(1.51,4.97)
**Kidney**	**perindopril**	44(4.0%)	81(1.7%)	1.94(1.25,2.99)	0.003	10	1.56(0.93,2.61)	1.97(1.20,3.25)
**Pancreas**	**clopidogrel**	35(3.9%)	63(1.7%)	2.37(1.45,3.86)	0.001	7	1.02(0.57,1.84)	2.45(1.45,4.13)
**Pancreas**	**lorazepam**	10(1.1%)	14(0.4%)	3.59(1.41,9.18)	0.008	12	1.74(0.55,5.49)	4.11(1.43,11.81)
**Pancreas**	**methotrexate**	9(1.0%)	9(0.2%)	4.39(1.57,12.26)	0.005	14	1.25(0.32,4.90)	4.66(1.50,14.48)
**Pancreas**	**quinine**	46(5.1%)	104(2.7%)	1.78(1.18,2.68)	0.006	5	0.49(0.28,0.86)	1.68(1.11,2.54)
**Uterus §**	**bendroflumethiazide**	194(23.1%)	699(18.1%)	1.32(1.07,1.63)	0.009	14	0.99(0.77,1.28)	1.53(1.20,1.95)
**Uterus §**	**doxazosin**	33(3.9%)	70(1.8%)	1.88(1.18,3.00)	0.008	9	1.07(0.59,1.94)	1.99(1.19,3.33)
**Uterus §**	**enalapril**	45(5.4%)	92(2.4%)	2.35(1.53,3.60)	<0.001	14	1.34(0.75,2.41)	2.67(1.65,4.31)
**Uterus §**	**loperamide**	12(1.4%)	30(0.8%)	2.88(1.36,6.09)	0.006	1	1.02(0.64,1.65)	1.35(0.75,2.42)
**Uterus §**	**salicylic acid***	7(0.8%)	8(0.2%)	6.85(2.15,21.83)	0.001	1	0.89(0.47,1.69)	2.29(1.25,4.23)
**Myeloma**	**chlortalidone**	11(2.5%)	22(1.2%)	3.06(1.32,7.12)	0.009	14	1.15(0.39,3.36)	3.68(1.46,9.31)
**Liver**	**furosemide**	89(19.8%)	143(7.4%)	2.48(1.58,3.88)	<0.001	12	1.19(0.73,1.96)	2.23(1.35,3.69)
**Thyroid**	**progestogen (HRT) §**	17(8.5%)	42(4.5%)	2.55(1.27,5.11)	0.008	3	1.19(0.67,2.10)	2.05(1.09,3.84)
**Anal**	**bisoprolol**	12(5.3%)	24(2.5%)	3.30(1.34,8.14)	0.010	14	1.69(0.62,4.64)	3.10(1.01,9.45)
**Anal**	**nicotine**	5(2.2%)	3(0.3%)	8.07(1.69,38.46)	0.009	2	0.93(0.41,2.13)	1.85(0.73,4.70)
**Signals associated with decreased cancer risk**								
**Breast §**	**Iron**	164(1.7%)	854(1.9%)	0.72(0.60,0.86)	<0.001	2	0.97(0.86,1.08)	0.80(0.70,0.92)
**Breast §**	**trazodone**	50(0.5%)	365(0.8%)	0.62(0.46,0.85)	0.003	3	0.75(0.60,0.96)	0.72(0.55,0.95)
**Breast §**	**trimethoprim**	82(0.8%)	493(1.1%)	0.71(0.55,0.90)	0.005	1	1.03(0.95,1.11)	0.93(0.84,1.02)
**Colorectal**	**diclofenac**	293(4.3%)	1,694(5.9%)	0.72(0.63,0.83)	<0.001	2	0.94(0.86,1.03)	0.84(0.75,0.93)
**Colorectal**	**lactulose**	207(3.1%)	896(3.1%)	0.77(0.65,0.91)	0.003	3	0.98(0.86,1.11)	0.80(0.68,0.93)
**Colorectal**	**naproxen**	85(1.3%)	487(1.7%)	0.71(0.56,0.91)	0.007	2	0.87(0.74,1.03)	0.77(0.63,0.94)

Table S4 (cont)

Cancer	Medicine	No. (%) cases	No. (%) controls	OR (95%CI)	p-value	Median ††	Low use v non-use: OR (95%CI)	High use v non-use: OR (95%CI)
**Colorectal**	**oxybutynin**	38(0.6%)	227(0.8%)	0.60(0.42,0.87)	0.006	4	1.04(0.79,1.36)	0.66(0.47,0.93)
**Prostate §§**	**calcium**	78(1.3%)	390(1.6%)	0.67(0.51,0.89)	0.006	7	0.90(0.69,1.17)	0.68(0.50,0.93)
**Prostate §§**	**citalopram**	41(0.7%)	319(1.3%)	0.53(0.37,0.75)	<0.001	5	0.98(0.76,1.27)	0.53(0.37,0.75)
**Prostate §§**	**furosemide**	362(6.1%)	1,761(7.2%)	0.74(0.64,0.85)	<0.001	12	0.98(0.85,1.13)	0.71(0.60,0.84)
**Prostate §§**	**lactulose**	138(2.3%)	631(2.6%)	0.70(0.57,0.86)	0.001	2	0.95(0.82,1.10)	0.73(0.62,0.88)
**Prostate §§**	**senna**	67(1.1%)	328(1.3%)	0.67(0.50,0.90)	0.007	2	0.91(0.75,1.10)	0.61(0.48,0.78)
**Prostate §§**	**vitamin D**	61(1.0%)	342(1.4%)	0.60(0.44,0.82)	0.001	6	0.99(0.76,1.29)	0.60(0.43,0.83)
**Bladder**	**pantoprazole**	5(0.2%)	43(0.4%)	0.27(0.10,0.71)	0.008	7	2.08(1.16,3.72)	0.22(0.08,0.66)
**Bladder**	**sotalol**	5(0.2%)	63(0.6%)	0.25(0.09,0.71)	0.010	13	0.19(0.05,0.82)	0.18(0.04,0.78)
**Oesophagus**	**aspirin high dose**	28(1.5%)	167(2.1%)	0.52(0.33,0.83)	0.006	10	0.88(0.57,1.38)	0.53(0.31,0.89)
**Non-Hodgkin's lymphoma**	**vitamin B**	26(1.6%)	143(2.0%)	0.51(0.32,0.81)	0.004	5	0.96(0.68,1.35)	0.50(0.32,0.81)
**Leukaemia**	**calcium**	42(2.9%)	205(3.3%)	0.56(0.37,0.85)	0.007	7	0.83(0.56,1.23)	0.50(0.32,0.79)
**Stomach**	**diclofenac***	3(0.3%)	44(0.9%)	0.18(0.05,0.63)	0.007	1	0.97(0.61,1.56)	0.60(0.33,1.07)
**Kidney**	**digoxin**	15(1.3%)	83(1.8%)	0.43(0.23,0.80)	0.008	19	0.79(0.41,1.53)	0.38(0.17,0.86)

† comorbidities include diabetes, myocardial infarction, coronary heart disease, heart failure, peripheral vascular disease, dementia, cerebrovascular disease, chronic obstructive pulmonary disease, osteoporosis, rheumatological disease, renal disease, liver disease, irritable bowel disease, human immunodeficiency viruses and hemiplegia/paraplegia, plus potential site-specific confounders; OR odds ratio; CI confidence interval; medicines systemic unless otherwise indicated* (local); HRT: hormone replacement therapy; § analyses restricted to females only; §§ analyses restricted to males only; low use: patient received more than I item of medicine and <=median number of items; high use: patient received > median number of items of medicine; †† among control users; - no cases with >=6 prescriptions in category

Table S5: Sensitivity analyses for comorbidity† adjusted signals: exposure any prescription

		1 year lag (main analysis)		2 year lag		Exclude matched sets where cases have additional cancer diagnosis within 12 months		Adjusted for comorbidities, smoking & alcohol	
**Cancer**	**Medicine**	**OR (95%CI)**	**p-value**	**OR (95%CI)**	**p-value**	**OR (95%CI)**	**p-value**	**OR (95%CI)**	**p-value**
**Signals associated with increased cancer risk**									
**Breast §**	**estrogen-HRT**	1.26(1.19,1.33)	<0.001	1.27(1.20,1.35)	<0.001	1.26(1.19,1.33)	<0.001	1.22(1.14,1.31)	<0.001
**Breast §**	**metronidazole***	1.25(1.06,1.48)	0.007	1.27(1.06,1.52)	0.009	1.26(1.06,1.48)	0.007	1.24(1.02,1.51)	0.031
**Breast §**	**progestogen-HRT**	1.28(1.21,1.35)	<0.001	1.30(1.23,1.38)	<0.001	1.28(1.21,1.35)	<0.001	1.23(1.15,1.31)	<0.001
**Lung**	**amoxicillin**	1.40(1.33,1.48)	<0.001	1.36(1.28,1.43)	<0.001	1.40(1.32,1.48)	<0.001	1.28(1.19,1.38)	<0.001
**Lung**	**azathioprine**	1.56(1.14,2.13)	0.005	1.64(1.18,2.29)	0.003	1.56(1.14,2.14)	0.006	1.99(1.32,2.99)	0.001
**Lung**	**cimetidine**	1.39(1.27,1.51)	<0.001	1.39(1.27,1.52)	<0.001	1.39(1.27,1.52)	<0.001	1.35(1.20,1.52)	<0.001
**Lung**	**clarithromycin**	1.34(1.23,1.46)	<0.001	1.35(1.23,1.48)	<0.001	1.33(1.22,1.45)	<0.001	1.30(1.17,1.45)	<0.001
**Lung**	**codeine**	1.31(1.24,1.38)	<0.001	1.23(1.16,1.30)	<0.001	1.31(1.24,1.39)	<0.001	1.21(1.13,1.31)	<0.001
**Lung**	**dihydrocodeine**	1.29(1.21,1.38)	<0.001	1.27(1.18,1.36)	<0.001	1.29(1.21,1.38)	<0.001	1.16(1.06,1.27)	0.001
**Lung**	**folic acid §**	1.49(1.27,1.75)	<0.001	1.51(1.27,1.80)	<0.001	1.51(1.28,1.77)	<0.001	1.43(1.15,1.76)	0.001
**Lung**	**ipratropium***	1.58(1.43,1.74)	<0.001	1.60(1.44,1.78)	<0.001	1.59(1.44,1.76)	<0.001	1.42(1.25,1.63)	<0.001
**Lung**	**nitrazepam**	1.35(1.14,1.60)	<0.001	1.37(1.15,1.63)	<0.001	1.34(1.13,1.59)	0.001	1.55(1.23,1.96)	<0.001
**Lung**	**nystatin**	1.27(1.12,1.44)	<0.001	1.29(1.12,1.49)	<0.001	1.26(1.11,1.42)	<0.001	1.21(1.03,1.42)	0.019
**Lung**	**paracetamol**	1.34(1.27,1.42)	<0.001	1.27(1.20,1.34)	<0.001	1.35(1.28,1.43)	<0.001	1.24(1.15,1.34)	<0.001
**Lung**	**salbutamol***	1.44(1.34,1.54)	<0.001	1.36(1.27,1.46)	<0.001	1.44(1.34,1.55)	<0.001	1.39(1.27,1.53)	<0.001
**Lung**	**salmeterol***	1.34(1.21,1.48)	<0.001	1.31(1.18,1.46)	<0.001	1.34(1.21,1.48)	<0.001	1.39(1.21,1.58)	<0.001
**Lung**	**tiotropium***	1.75(1.52,2.02)	<0.001	1.64(1.38,1.94)	<0.001	1.77(1.53,2.04)	<0.001	1.63(1.37,1.95)	<0.001
**Colorectal**	**allopurinol**	1.27(1.11,1.46)	0.001	1.31(1.13,1.51)	<0.001	1.24(1.08,1.43)	0.002	1.26(1.06,1.50)	0.010
**Colorectal**	**prednisolone***	1.31(1.12,1.54)	0.001	1.28(1.07,1.52)	0.007	1.32(1.12,1.54)	0.001	1.18(0.97,1.43)	0.090
**Prostate §§**	**cerivastatin**	1.43(1.10,1.87)	0.008	1.40(1.06,1.85)	0.019	1.44(1.10,1.89)	0.007	1.27(0.91,1.77)	0.168
**Prostate §§**	**clioquinol***	1.30(1.11,1.52)	0.001	1.34(1.13,1.58)	0.001	1.32(1.13,1.54)	0.001	1.31(1.09,1.57)	0.004
**Bladder**	**celecoxib**	1.40(1.14,1.73)	0.002	1.38(1.10,1.74)	0.006	1.40(1.13,1.74)	0.002	1.29(1.01,1.66)	0.040
**Bladder**	**dexamethasone***	1.30(1.10,1.54)	0.003	1.22(1.01,1.48)	0.041	1.29(1.08,1.53)	0.005	1.19(0.97,1.46)	0.104
**Bladder**	**nicotine**	2.04(1.65,2.51)	<0.001	1.97(1.55,2.50)	<0.001	2.03(1.64,2.51)	<0.001	1.65(1.28,2.13)	<0.001
**Bladder**	**trimethoprim**	1.96(1.73,2.21)	<0.001	1.56(1.36,1.78)	<0.001	1.95(1.72,2.20)	<0.001	1.95(1.68,2.26)	<0.001
**Malignant melanoma**	**chloramphenicol***	1.28(1.08,1.51)	0.003	1.30(1.09,1.55)	0.004	1.28(1.09,1.51)	0.003	1.04(0.85,1.27)	0.701
**Malignant melanoma**	**clopidogrel**	1.60(1.13,2.27)	0.008	1.61(1.08,2.40)	0.019	1.56(1.09,2.22)	0.014	1.51(1.01,2.27)	0.045
**Malignant melanoma**	**flucloxacillin**	1.28(1.11,1.46)	<0.001	1.28(1.10,1.48)	0.001	1.27(1.11,1.46)	0.001	1.27(1.07,1.50)	0.006
**Oesophagus**	**alginic acid**	1.40(1.22,1.62)	<0.001	1.36(1.17,1.59)	<0.001	1.42(1.22,1.64)	<0.001	1.35(1.12,1.63)	0.002
**Oesophagus**	**azathioprine**	2.47(1.41,4.35)	0.002	2.42(1.30,4.51)	0.005	2.47(1.40,4.34)	0.002	2.46(1.17,5.20)	0.018
**Oesophagus**	**cisapride**	2.03(1.27,3.25)	0.003	1.97(1.23,3.18)	0.005	2.04(1.27,3.26)	0.003	2.17(1.13,4.18)	0.020
**Oesophagus**	**gramicidin***	1.52(1.11,2.09)	0.008	1.55(1.10,2.17)	0.012	1.52(1.11,2.09)	0.009	1.42(0.96,2.11)	0.080
**Oesophagus**	**lansoprazole**	1.35(1.17,1.55)	<0.001	1.28(1.10,1.49)	0.001	1.33(1.15,1.53)	<0.001	1.26(1.06,1.50)	0.009
**Oesophagus**	**nicotine**	1.50(1.16,1.94)	0.002	1.35(1.01,1.80)	0.043	1.51(1.17,1.96)	0.002	1.18(0.87,1.60)	0.277
**Oesophagus**	**nystatin**	1.58(1.21,2.06)	0.001	1.68(1.25,2.25)	0.001	1.56(1.19,2.04)	0.001	1.76(1.27,2.43)	0.001
**Oesophagus**	**nystatin***	1.29(1.08,1.55)	0.005	1.34(1.11,1.63)	0.003	1.27(1.06,1.53)	0.010	1.44(1.16,1.79)	0.001

Table S5 (cont)

		1 year lag (main analysis)		2 year lag		Exclude matched sets where cases have additional cancer diagnosis within 12 months		Adjusted for comorbidities, smoking & alcohol	
**Cancer**	**Medicine**	**OR (95%CI)**	**p-value**	**OR (95%CI)**	**p-value**	**OR (95%CI)**	**p-value**	**OR (95%CI)**	**p-value**
**Oesophagus**	**omeprazole**	1.39(1.23,1.57)	<0.001	1.31(1.15,1.50)	<0.001	1.39(1.22,1.57)	<0.001	1.25(1.07,1.46)	0.006
**Oesophagus**	**triamcinolone***	1.47(1.13,1.91)	0.004	1.42(1.07,1.88)	0.016	1.48(1.14,1.93)	0.003	1.44(1.03,2.00)	0.033
**Oesophagus**	**vitamin B**	1.36(1.12,1.65)	0.002	1.32(1.07,1.62)	0.010	1.37(1.12,1.66)	0.002	1.13(0.87,1.46)	0.352
**Non-Hodgkin's lymphoma**	**amoxicillin**	1.34(1.19,1.50)	<0.001	1.29(1.14,1.46)	<0.001	1.36(1.21,1.52)	<0.001	1.22(1.05,1.42)	0.008
**Non-Hodgkin's lymphoma**	**betamethasone***	1.37(1.19,1.57)	<0.001	1.40(1.21,1.63)	<0.001	1.37(1.19,1.57)	<0.001	1.23(1.03,1.47)	0.021
**Non-Hodgkin's lymphoma**	**chlorhexidine***	1.67(1.20,2.31)	0.002	1.66(1.15,2.39)	0.007	1.69(1.22,2.35)	0.002	1.27(0.81,1.98)	0.298
**Non-Hodgkin's lymphoma**	**clarithromycin**	1.33(1.09,1.63)	0.005	1.21(0.97,1.52)	0.093	1.32(1.08,1.62)	0.007	1.30(1.02,1.66)	0.033
**Non-Hodgkin's lymphoma**	**clobetasol***	1.73(1.25,2.38)	0.001	1.78(1.26,2.52)	0.001	1.71(1.23,2.36)	0.001	1.50(1.00,2.25)	0.048
**Non-Hodgkin's lymphoma**	**erythromycin**	1.26(1.07,1.49)	0.006	1.21(1.01,1.44)	0.035	1.29(1.09,1.52)	0.003	1.25(1.02,1.53)	0.032
**Non-Hodgkin's lymphoma**	**hydroxyzine**	1.75(1.16,2.66)	0.008	1.74(1.07,2.81)	0.024	1.67(1.09,2.56)	0.018	1.75(1.07,2.87)	0.027
**Non-Hodgkin's lymphoma**	**methotrexate**	2.93(1.50,5.69)	0.002	3.22(1.61,6.44)	0.001	3.01(1.54,5.89)	0.001	3.66(1.58,8.45)	0.002
**Non-Hodgkin's lymphoma**	**prednisolone**	1.37(1.13,1.66)	0.001	1.33(1.08,1.64)	0.007	1.36(1.12,1.65)	0.002	1.45(1.14,1.83)	0.002
**Non-Hodgkin's lymphoma**	**prochlorperazine**	1.30(1.08,1.58)	0.007	1.32(1.07,1.63)	0.009	1.31(1.08,1.59)	0.007	1.39(1.10,1.75)	0.006
**Leukaemia**	**allopurinol**	1.57(1.20,2.06)	0.001	1.44(1.07,1.93)	0.015	1.56(1.18,2.05)	0.002	1.88(1.32,2.68)	0.001
**Leukaemia**	**amoxicillin**	1.29(1.14,1.45)	<0.001	1.22(1.07,1.38)	0.002	1.28(1.13,1.44)	<0.001	1.15(0.98,1.35)	0.088
**Leukaemia**	**trimethoprim**	1.26(1.06,1.49)	0.010	1.20(0.99,1.45)	0.064	1.25(1.05,1.49)	0.013	1.35(1.08,1.68)	0.009
**Oral (inc. Head,neck & nasal)**	**codeine**	1.29(1.12,1.49)	<0.001	1.28(1.11,1.49)	0.001	1.27(1.10,1.47)	0.001	1.10(0.91,1.32)	0.336
**Oral (inc. Head,neck & nasal)**	**flucloxacillin**	1.36(1.13,1.62)	0.001	1.31(1.08,1.60)	0.007	1.39(1.16,1.67)	<0.001	1.30(1.04,1.64)	0.024
**Oral (inc. Head,neck & nasal)**	**folic acid §**	2.52(1.64,3.89)	<0.001	2.42(1.52,3.85)	<0.001	2.51(1.62,3.88)	<0.001	2.38(1.29,4.39)	0.006
**Oral (inc. Head,neck & nasal)**	**nystatin**	2.06(1.49,2.85)	<0.001	1.94(1.35,2.79)	<0.001	2.06(1.48,2.85)	<0.001	1.73(1.15,2.61)	0.009
**Oral (inc. Head,neck & nasal)**	**vitamin B**	2.75(2.22,3.41)	<0.001	2.73(2.17,3.43)	<0.001	2.79(2.24,3.47)	<0.001	2.10(1.57,2.79)	<0.001
**Oral (inc. Head,neck & nasal)**	**vitamin D**	1.69(1.24,2.30)	0.001	1.78(1.26,2.51)	0.001	1.69(1.24,2.31)	0.001	1.65(1.10,2.49)	0.015
**Stomach**	**cimetidine**	1.44(1.15,1.79)	0.001	1.40(1.12,1.76)	0.003	1.42(1.13,1.78)	0.002	4.08(1.54,10.81)	0.005
**Stomach**	**clarithromycin**	1.35(1.08,1.69)	0.009	1.22(0.95,1.56)	0.114	1.35(1.07,1.70)	0.011	1.91(0.94,3.89)	0.075
**Stomach**	**digoxin**	1.49(1.10,2.01)	0.010	1.43(1.03,1.97)	0.031	1.52(1.12,2.07)	0.007	1.19(0.90,1.59)	0.220
**Stomach**	**vitamin B**	1.54(1.21,1.95)	<0.001	1.56(1.21,2.00)	0.001	1.56(1.22,1.98)	<0.001	1.11(0.85,1.46)	0.436
**Ovary §**	**phenytoin**	3.24(1.62,6.50)	0.001	3.52(1.74,7.12)	<0.001	3.27(1.63,6.55)	0.001	1.57(1.08,2.29)	0.019
**Ovary §**	**rabeprazole**	2.18(1.22,3.89)	0.009	2.68(1.46,4.93)	0.002	2.20(1.23,3.94)	0.008	1.98(1.45,2.70)	<0.001
**Kidney**	**amoxicillin**	1.28(1.11,1.48)	0.001	1.19(1.03,1.39)	0.020	1.29(1.11,1.49)	0.001	1.28(1.07,1.53)	0.007
**Kidney**	**hydrochlorothiazide**	1.93(1.30,2.86)	0.001	2.01(1.35,3.00)	0.001	2.02(1.36,3.01)	0.001	2.29(1.41,3.71)	0.001
**Kidney**	**perindopril**	1.63(1.16,2.30)	0.005	1.52(1.03,2.23)	0.035	1.73(1.22,2.44)	0.002	1.78(1.19,2.67)	0.005
**Pancreas**	**betamethasone***	1.34(1.11,1.62)	0.003	1.31(1.07,1.61)	0.008	1.34(1.11,1.62)	0.003	1.27(0.96,1.67)	0.093
**Pancreas**	**nicotine**	1.94(1.38,2.73)	<0.001	1.44(0.98,2.12)	0.064	1.99(1.41,2.80)	<0.001	1.13(0.83,1.52)	0.440
**Cervix & other gynae §**	**dihydrocodeine**	1.39(1.11,1.73)	0.004	1.33(1.05,1.69)	0.019	1.38(1.10,1.72)	0.006	1.35(1.07,1.70)	0.012
**Cervix & other gynae §**	**ranitidine**	1.37(1.08,1.73)	0.010	1.31(1.02,1.69)	0.035	1.38(1.08,1.75)	0.009	1.18(0.78,1.78)	0.435
**Uterus §**	**atenolol**	1.39(1.15,1.67)	0.001	1.34(1.11,1.62)	0.003	1.41(1.17,1.70)	<0.001	1.31(1.05,1.65)	0.019

Table S5 (cont)

		1 year lag (main analysis)		2 year lag		Exclude matched sets where cases have additional cancer diagnosis within 12 months		Adjusted for comorbidities, smoking & alcohol	
**Cancer**	**Medicine**	**OR (95%CI)**	**p-value**	**OR (95%CI)**	**p-value**	**OR (95%CI)**	**p-value**	**OR (95%CI)**	**p-value**
**Uterus §**	**bendroflumethiazide**	1.27(1.07,1.51)	0.005	1.32(1.10,1.57)	0.002	1.27(1.07,1.51)	0.006	1.24(1.01,1.53)	0.041
**Uterus §**	**bisoprolol**	1.82(1.21,2.74)	0.004	1.94(1.24,3.02)	0.004	1.79(1.18,2.74)	0.007	1.64(1.03,2.61)	0.037
**Uterus §**	**doxazosin**	1.68(1.17,2.43)	0.005	1.78(1.21,2.63)	0.003	1.67(1.15,2.43)	0.007	1.46(0.94,2.26)	0.088
**Uterus §**	**enalapril**	1.66(1.18,2.33)	0.003	1.78(1.26,2.52)	0.001	1.77(1.26,2.50)	0.001	2.31(1.52,3.52)	<0.001
**Uterus §**	**ibuprofen***	1.53(1.14,2.06)	0.005	1.73(1.26,2.38)	0.001	1.54(1.15,2.08)	0.004	1.24(0.86,1.79)	0.241
**Uterus §**	**mefenamic acid**	1.72(1.22,2.42)	0.002	1.48(1.03,2.13)	0.032	1.78(1.27,2.51)	0.001	1.54(1.01,2.35)	0.046
**Uterus §**	**tranexamic acid**	1.87(1.25,2.79)	0.002	1.34(0.85,2.12)	0.212	1.96(1.31,2.93)	0.001	1.27(0.79,2.02)	0.325
**Larynx**	**benzydamine***	2.33(1.34,4.04)	0.003	1.71(0.90,3.23)	0.101	2.11(1.18,3.79)	0.012	1.49(0.78,2.86)	0.228
**Larynx**	**dextropropoxyphene**	1.51(1.19,1.92)	0.001	1.52(1.19,1.95)	0.001	1.53(1.20,1.96)	0.001	2.35(1.07,5.14)	0.033
**Larynx**	**paracetamol**	1.45(1.20,1.76)	<0.001	1.36(1.12,1.65)	0.002	1.48(1.22,1.81)	<0.001	1.60(1.15,2.24)	0.006
**Larynx**	**vitamin B**	2.40(1.72,3.36)	<0.001	2.24(1.54,3.25)	<0.001	2.54(1.80,3.58)	<0.001	1.30(0.98,1.72)	0.067
**Brain and CNS**	**carbamazepine**	2.20(1.34,3.62)	0.002	1.76(1.01,3.06)	0.047	2.27(1.37,3.73)	0.001	1.49(0.95,2.33)	0.083
**Myeloma**	**amoxicillin**	1.53(1.21,1.92)	<0.001	1.56(1.23,1.98)	<0.001	1.56(1.24,1.97)	<0.001	1.33(0.99,1.79)	0.060
**Myeloma**	**ciprofloxacin**	1.73(1.17,2.55)	0.006	1.68(1.09,2.58)	0.019	1.84(1.24,2.72)	0.002	1.58(0.99,2.54)	0.056
**Thyroid**	**gabapentin**	5.13(1.71,15.37)	0.003	4.05(1.17,14.09)	0.028	5.00(1.67,15.01)	0.004	3.99(1.01,15.82)	0.049
**Thyroid**	**levothyroxine**	2.18(1.37,3.48)	0.001	2.45(1.50,4.01)	<0.001	2.27(1.42,3.64)	0.001	2.72(1.43,5.17)	0.002
**Anal**	**clonidine**	3.87(1.52,9.85)	0.005	5.16(1.87,14.27)	0.002	5.08(1.89,13.67)	0.001	7.31(2.16,24.74)	0.001
**Anal**	**doxazosin**	2.48(1.27,4.86)	0.008	3.83(1.83,8.04)	<0.001	2.40(1.17,4.96)	0.018	2.99(1.25,7.14)	0.014
**Signals associated with decreased cancer risk**									
**Breast §**	**trazodone**	0.79(0.67,0.93)	0.005	0.80(0.67,0.96)	0.015	0.80(0.68,0.94)	0.007	0.75(0.62,0.92)	0.005
**Lung**	**fluocinolone***	0.67(0.52,0.88)	0.003	0.68(0.52,0.90)	0.008	0.68(0.52,0.88)	0.004	0.66(0.47,0.92)	0.014
**Lung**	**levodopa**	0.50(0.35,0.73)	<0.001	0.40(0.26,0.61)	<0.001	0.49(0.33,0.71)	<0.001	0.66(0.41,1.06)	0.087
**Lung**	**nitrofurantoin**	0.74(0.60,0.93)	0.008	0.74(0.58,0.95)	0.018	0.72(0.58,0.91)	0.005	0.73(0.56,0.96)	0.024
**Lung**	**progestogen-contraceptive §**	0.62(0.45,0.86)	0.004	0.57(0.41,0.80)	0.001	0.64(0.46,0.88)	0.007	0.55(0.36,0.86)	0.008
**Lung**	**risperidone**	0.55(0.36,0.84)	0.005	0.57(0.36,0.90)	0.017	0.56(0.37,0.85)	0.006	0.57(0.33,1.01)	0.054
**Colorectal**	**levodopa**	0.60(0.43,0.85)	0.004	0.61(0.42,0.88)	0.009	0.62(0.44,0.87)	0.007	0.74(0.48,1.14)	0.170
**Colorectal**	**lofepramine**	0.71(0.55,0.92)	0.009	0.74(0.56,0.97)	0.028	0.73(0.56,0.94)	0.016	0.68(0.49,0.94)	0.021
**Colorectal**	**meloxicam**	0.76(0.63,0.92)	0.005	0.77(0.62,0.95)	0.014	0.78(0.64,0.94)	0.010	0.74(0.59,0.93)	0.008
**Colorectal**	**metoclopramide**	0.78(0.68,0.91)	0.001	0.77(0.65,0.91)	0.002	0.80(0.69,0.93)	0.003	0.78(0.65,0.93)	0.005
**Colorectal**	**nitrofurantoin**	0.71(0.56,0.91)	0.006	0.55(0.41,0.73)	<0.001	0.71(0.56,0.91)	0.006	0.65(0.48,0.87)	0.003
**Prostate §§**	**levodopa**	0.47(0.30,0.72)	0.001	0.48(0.30,0.78)	0.003	0.47(0.30,0.72)	0.001	0.44(0.25,0.80)	0.007
**Prostate §§**	**risperidone**	0.37(0.20,0.70)	0.002	0.42(0.21,0.84)	0.013	0.40(0.22,0.75)	0.004	0.44(0.20,0.96)	0.038
**Prostate §§**	**senna**	0.76(0.66,0.87)	<0.001	0.73(0.62,0.85)	<0.001	0.75(0.65,0.87)	<0.001	0.78(0.65,0.92)	0.004
**Bladder**	**iron**	0.77(0.64,0.91)	0.003	0.72(0.59,0.88)	0.001	0.77(0.65,0.92)	0.005	0.81(0.65,1.01)	0.056
**Malignant melanoma**	**dipyridamole**	0.42(0.22,0.80)	0.008	0.35(0.17,0.72)	0.005	0.43(0.22,0.81)	0.009	0.61(0.29,1.27)	0.187
**Malignant melanoma**	**gliclazide**	0.51(0.32,0.80)	0.004	0.49(0.30,0.79)	0.004	0.53(0.34,0.83)	0.006	0.41(0.24,0.69)	0.001
**Oesophagus**	**estrogen-HRT §**	0.59(0.43,0.79)	<0.001	0.61(0.46,0.83)	0.001	0.60(0.45,0.82)	0.001	0.56(0.39,0.81)	0.002

Table S5 (cont)

		1 year lag (main analysis)		2 year lag		Exclude matched sets where cases have additional cancer diagnosis within 12 months		Adjusted for comorbidities, smoking & alcohol	
**Cancer**	**Medicine**	**OR (95%CI)**	**p-value**	**OR (95%CI)**	**p-value**	**OR (95%CI)**	**p-value**	**OR (95%CI)**	**p-value**
**Non-Hodgkin's lymphoma**	**progestogen-contraceptive §**	0.56(0.36,0.86)	0.008	0.51(0.32,0.80)	0.004	0.54(0.35,0.85)	0.007	0.54(0.32,0.93)	0.027
**Oral (inc. Head,neck & nasal)**	**doxycycline**	0.45(0.30,0.66)	<0.001	0.45(0.29,0.69)	<0.001	0.43(0.28,0.64)	<0.001	0.41(0.25,0.67)	<0.001
**Oral (inc. Head,neck & nasal)**	**ispaghula**	0.63(0.47,0.85)	0.002	0.62(0.45,0.85)	0.003	0.65(0.48,0.87)	0.005	0.72(0.49,1.04)	0.083
**Ovary §**	**loratadine**	0.64(0.46,0.89)	0.008	0.71(0.51,1.00)	0.050	0.63(0.45,0.88)	0.007	0.70(0.47,1.04)	0.076
**Ovary §**	**progestogen-contraceptive**	0.55(0.39,0.78)	0.001	0.52(0.37,0.75)	<0.001	0.56(0.39,0.79)	0.001	0.47(0.30,0.72)	0.001
**Cervix & other gynae §**	**terbinafine**	0.18(0.07,0.49)	0.001	0.16(0.05,0.51)	0.002	0.19(0.07,0.52)	0.001	0.19(0.07,0.53)	0.002
**Uterus §**	**estrogen-HRT**	0.73(0.60,0.89)	0.002	0.71(0.58,0.87)	0.001	0.71(0.58,0.87)	0.001	0.81(0.64,1.03)	0.081
**Uterus §**	**progestogen-contraceptive**	0.37(0.19,0.69)	0.002	0.38(0.20,0.72)	0.003	0.37(0.20,0.70)	0.002	0.40(0.20,0.80)	0.010
**Larynx**	**doxazosin**	0.36(0.19,0.70)	0.002	0.43(0.22,0.84)	0.014	0.37(0.19,0.71)	0.003	0.43(0.19,0.98)	0.044
**Larynx**	**terbinafine**	0.32(0.14,0.75)	0.009	0.33(0.13,0.84)	0.020	0.33(0.14,0.77)	0.010	0.31(0.10,0.99)	0.048

† comorbidities include diabetes, myocardial infarction, coronary heart disease, heart failure, peripheral vascular disease, dementia, cerebrovascular disease, chronic obstructive pulmonary disease, osteoporosis, rheumatological disease, renal disease, liver disease, irritable bowel disease, human immunodeficiency viruses and hemiplegia/paraplegia, plus potential site-specific confounders; OR odds ratio; CI confidence interval; CNS: central nervous system; medicines systemic unless otherwise indicated* (local); HRT: hormone replacement therapy; § females only; §§ males only

Table S6: Sensitivity analyses for comorbidity† & smoking adjusted signals: exposure any prescription

		1 year lag (main analysis)		2 year lag		Exclude matched sets where cases have additional cancer diagnosis within 12 months		1 year lag with multiple imputation		Adjusted for comorbidities smoking & alcohol	
**Cancer**	**Medicine**	**OR (95%CI)**	**p-value**	**OR (95%CI)**	**p-value**	**OR (95%CI)**	**p-value**	**OR (95%CI)**	**p-value**	**OR (95%CI)**	**p-value**
**Signals associated with increased cancer risk**											
**Breast §**	**bisoprolol**	1.30(1.11,1.51)	0.001	1.27(1.07,1.51)	0.007	1.32(1.13,1.54)	<0.001	1.34(1.16,1.54)	<0.001	1.30(1.10,1.54)	0.002
**Breast §**	**metronidazole***	1.26(1.06,1.51)	0.010	1.32(1.09,1.60)	0.005	1.26(1.05,1.50)	0.012	1.25(1.06,1.47)	0.009	1.24(1.02,1.51)	0.030
**Breast §**	**progestogen (HRT)**	1.25(1.17,1.33)	<0.001	1.28(1.20,1.37)	<0.001	1.25(1.18,1.33)	<0.001	1.28(1.21,1.35)	<0.001	1.23(1.15,1.31)	<0.001
**Lung**	**amoxicillin**	1.29(1.21,1.37)	<0.001	1.26(1.18,1.35)	<0.001	1.29(1.20,1.37)	<0.001	1.37(1.29,1.45)	<0.001	1.28(1.19,1.37)	<0.001
**Lung**	**azathioprine**	1.76(1.21,2.57)	0.003	1.96(1.30,2.95)	0.001	1.81(1.24,2.65)	0.002	1.71(1.22,2.39)	0.002	2.02(1.34,3.06)	0.001
**Lung**	**cimetidine**	1.35(1.22,1.50)	<0.001	1.29(1.16,1.44)	<0.001	1.37(1.23,1.52)	<0.001	1.30(1.19,1.43)	<0.001	1.35(1.20,1.52)	<0.001
**Lung**	**ipratropium***	1.43(1.27,1.61)	<0.001	1.49(1.31,1.69)	<0.001	1.45(1.29,1.63)	<0.001	1.46(1.31,1.62)	<0.001	1.42(1.24,1.62)	<0.001
**Lung**	**nitrazepam**	1.34(1.09,1.65)	0.005	1.44(1.15,1.79)	0.001	1.32(1.07,1.62)	0.009	1.30(1.09,1.57)	0.005	1.52(1.21,1.93)	<0.001
**Lung**	**salbutamol***	1.35(1.24,1.47)	<0.001	1.28(1.18,1.40)	<0.001	1.35(1.24,1.47)	<0.001	1.41(1.30,1.52)	<0.001	1.38(1.26,1.52)	<0.001
**Lung**	**salmeterol***	1.44(1.28,1.62)	<0.001	1.47(1.29,1.67)	<0.001	1.45(1.28,1.63)	<0.001	1.42(1.27,1.58)	<0.001	1.38(1.21,1.58)	<0.001
**Lung**	**tiotropium***	1.63(1.39,1.91)	<0.001	1.49(1.23,1.80)	<0.001	1.63(1.39,1.91)	<0.001	1.66(1.43,1.94)	<0.001	1.61(1.35,1.92)	<0.001
**Colorectal**	**allopurinol**	1.26(1.08,1.48)	0.004	1.31(1.10,1.56)	0.002	1.22(1.04,1.44)	0.015	1.26(1.10,1.45)	0.001	1.26(1.06,1.50)	0.010
**Prostate §§**	**alfuzosin**	1.66(1.37,2.01)	<0.001	1.58(1.26,1.98)	<0.001	1.62(1.33,1.97)	<0.001	1.69(1.43,2.01)	<0.001	1.63(1.32,2.02)	<0.001
**Prostate §§**	**clioquinol***	1.34(1.13,1.58)	0.001	1.36(1.13,1.64)	0.001	1.36(1.15,1.61)	<0.001	1.30(1.11,1.52)	0.001	1.31(1.09,1.57)	0.004
**Bladder**	**cefalexin**	1.37(1.17,1.60)	<0.001	1.30(1.09,1.55)	0.003	1.35(1.15,1.58)	<0.001	1.43(1.24,1.65)	<0.001	1.42(1.19,1.69)	<0.001
**Bladder**	**ciprofloxacin**	1.40(1.18,1.65)	<0.001	1.28(1.06,1.55)	0.012	1.38(1.16,1.63)	<0.001	1.47(1.26,1.72)	<0.001	1.30(1.08,1.56)	0.005
**Bladder**	**nicotine**	1.54(1.23,1.94)	<0.001	1.48(1.15,1.92)	0.003	1.56(1.24,1.97)	<0.001	1.67(1.34,2.07)	<0.001	1.65(1.28,2.13)	<0.001
**Bladder**	**phenoxymethylpenicillin**	1.30(1.08,1.56)	0.006	1.22(0.99,1.50)	0.064	1.31(1.08,1.58)	0.005	1.23(1.04,1.46)	0.017	1.34(1.10,1.65)	0.005
**Bladder**	**quinine**	1.28(1.06,1.54)	0.009	1.30(1.06,1.60)	0.011	1.28(1.06,1.55)	0.009	1.19(1.01,1.41)	0.043	1.37(1.11,1.68)	0.003
**Bladder**	**ranitidine**	1.26(1.10,1.44)	0.001	1.31(1.13,1.51)	<0.001	1.25(1.09,1.44)	0.002	1.24(1.10,1.40)	<0.001	1.29(1.11,1.49)	0.001
**Bladder**	**trimethoprim**	1.92(1.68,2.20)	<0.001	1.57(1.35,1.83)	<0.001	1.91(1.67,2.19)	<0.001	1.99(1.76,2.25)	<0.001	1.94(1.67,2.25)	<0.001
**Malignant melanoma**	**clopidogrel**	1.63(1.13,2.36)	0.009	1.55(1.01,2.39)	0.045	1.60(1.10,2.33)	0.014	1.61(1.14,2.28)	0.007	1.51(1.01,2.26)	0.047
**Oesophagus**	**alginic acid**	1.38(1.17,1.63)	<0.001	1.34(1.12,1.61)	0.002	1.39(1.17,1.65)	<0.001	1.41(1.21,1.63)	<0.001	1.36(1.13,1.64)	0.001
**Oesophagus**	**azathioprine**	3.39(1.79,6.42)	<0.001	3.70(1.77,7.72)	<0.001	3.29(1.73,6.25)	<0.001	2.71(1.53,4.79)	0.001	2.38(1.12,5.04)	0.024
**Oesophagus**	**ipratropium***	1.45(1.11,1.89)	0.006	1.59(1.19,2.14)	0.002	1.44(1.10,1.88)	0.009	1.40(1.10,1.76)	0.005	1.66(1.23,2.26)	0.001
**Oesophagus**	**lansoprazole**	1.33(1.14,1.55)	<0.001	1.23(1.03,1.46)	0.019	1.31(1.12,1.53)	0.001	1.34(1.16,1.54)	<0.001	1.27(1.07,1.51)	0.008
**Oesophagus**	**nystatin**	1.65(1.23,2.20)	0.001	1.86(1.34,2.58)	<0.001	1.61(1.20,2.15)	0.001	1.60(1.22,2.10)	0.001	1.76(1.27,2.44)	0.001
**Oesophagus**	**nystatin***	1.36(1.12,1.67)	0.002	1.46(1.17,1.82)	0.001	1.32(1.08,1.62)	0.007	1.34(1.11,1.60)	0.002	1.44(1.16,1.80)	0.001
**Oesophagus**	**omeprazole**	1.29(1.12,1.48)	<0.001	1.27(1.09,1.48)	0.003	1.29(1.12,1.49)	<0.001	1.39(1.23,1.58)	<0.001	1.26(1.07,1.47)	0.004
**Non-Hodgkin's lymphoma**	**betamethasone***	1.26(1.08,1.48)	0.004	1.24(1.04,1.47)	0.014	1.26(1.07,1.47)	0.004	1.37(1.19,1.57)	<0.001	1.23(1.03,1.47)	0.020
**Non-Hodgkin's lymphoma**	**clobetasol***	1.72(1.20,2.46)	0.003	1.77(1.19,2.62)	0.004	1.70(1.18,2.44)	0.004	1.73(1.25,2.39)	0.001	1.50(1.00,2.25)	0.048
**Non-Hodgkin's lymphoma**	**erythromycin**	1.28(1.07,1.54)	0.008	1.29(1.06,1.58)	0.012	1.30(1.08,1.57)	0.005	1.26(1.07,1.48)	0.006	1.25(1.02,1.53)	0.032
**Leukaemia**	**oxybutynin**	1.93(1.21,3.09)	0.006	1.77(1.06,2.95)	0.030	1.99(1.24,3.18)	0.004	1.62(1.12,2.35)	0.011	1.83(1.08,3.09)	0.024
**Oral (inc. Head,neck & nasal)**	**clobetasone***	1.41(1.10,1.81)	0.007	1.26(0.95,1.66)	0.105	1.39(1.08,1.79)	0.010	1.30(1.03,1.63)	0.027	1.38(1.04,1.83)	0.026
**Oral (inc. Head,neck & nasal)**	**nystatin**	1.92(1.33,2.77)	0.001	1.89(1.26,2.86)	0.002	1.91(1.32,2.77)	0.001	2.17(1.55,3.03)	<0.001	1.73(1.15,2.61)	0.009

Table S6 (cont)

		1 year lag (main analysis)		2 year lag		Exclude matched sets where cases have additional cancer diagnosis within 12 months		1 year lag with multiple imputation		Adjusted for comorbidities smoking & alcohol	
**Cancer**	**Medicine**	**OR (95%CI)**	**p-value**	**OR (95%CI)**	**p-value**	**OR (95%CI)**	**p-value**	**OR (95%CI)**	**p-value**	**OR (95%CI)**	**p-value**
**Oral (inc. Head,neck & nasal)**	**vitamin B**	2.33(1.82,3.00)	<0.001	2.55(1.94,3.36)	<0.001	2.39(1.85,3.08)	<0.001	2.40(1.92,3.00)	<0.001	2.09(1.57,2.79)	<0.001
**Ovary §**	**phenytoin**	3.86(1.68,8.89)	0.002	3.66(1.54,8.71)	0.003	3.84(1.67,8.87)	0.002	3.20(1.60,6.42)	0.001	4.06(1.53,10.77)	0.005
**Stomach**	**codeine**	1.35(1.15,1.59)	<0.001	1.32(1.11,1.57)	0.002	1.36(1.15,1.61)	<0.001	1.32(1.15,1.53)	<0.001	1.31(1.09,1.57)	0.004
**Stomach**	**nicotinates***	2.09(1.29,3.39)	0.003	2.38(1.40,4.05)	0.001	2.10(1.29,3.44)	0.003	1.63(1.03,2.57)	0.036	1.95(1.12,3.40)	0.018
**Stomach**	**vitamin B**	1.55(1.18,2.03)	0.002	1.66(1.23,2.23)	0.001	1.61(1.22,2.12)	0.001	1.51(1.19,1.92)	0.001	1.99(1.46,2.71)	<0.001
**Kidney**	**hydrochlorothiazide**	2.21(1.45,3.37)	<0.001	2.71(1.73,4.24)	<0.001	2.39(1.55,3.67)	<0.001	1.97(1.33,2.92)	0.001	2.30(1.42,3.72)	0.001
**Kidney**	**perindopril**	1.76(1.21,2.54)	0.003	1.62(1.05,2.48)	0.028	1.87(1.28,2.72)	0.001	1.65(1.17,2.33)	0.004	1.78(1.19,2.67)	0.005
**Pancreas**	**amoxicillin**	1.31(1.09,1.56)	0.003	1.34(1.11,1.62)	0.003	1.30(1.09,1.56)	0.004	1.44(1.23,1.69)	<0.001	1.30(1.07,1.59)	0.010
**Uterus §**	**chlortalidone**	2.14(1.32,3.49)	0.002	1.82(1.09,3.05)	0.022	2.26(1.38,3.68)	0.001	2.52(1.67,3.80)	<0.001	2.35(1.36,4.04)	0.002
**Uterus §**	**enalapril**	2.00(1.38,2.92)	<0.001	2.17(1.47,3.20)	<0.001	2.14(1.46,3.12)	<0.001	1.63(1.16,2.29)	0.005	2.30(1.51,3.51)	<0.001
**Brain and CNS**	**zinc oxide***	1.97(1.19,3.27)	0.008	2.24(1.29,3.91)	0.004	1.92(1.14,3.22)	0.014	1.71(1.08,2.71)	0.023	2.08(1.19,3.63)	0.010
**Larynx**	**dextropropoxyphene**	1.54(1.14,2.08)	0.004	1.67(1.22,2.28)	0.001	1.54(1.13,2.09)	0.006	1.50(1.16,1.95)	0.002	1.59(1.14,2.23)	0.007
**Larynx**	**paracetamol**	1.40(1.10,1.79)	0.007	1.28(0.99,1.65)	0.057	1.44(1.12,1.86)	0.004	1.35(1.09,1.66)	0.005	1.29(0.98,1.71)	0.073
**Larynx**	**vitamin B**	1.80(1.21,2.68)	0.004	1.84(1.17,2.90)	0.009	1.82(1.21,2.74)	0.004	2.00(1.39,2.88)	<0.001	1.47(0.94,2.31)	0.093
**Myeloma**	**amoxicillin**	1.44(1.11,1.86)	0.006	1.46(1.11,1.93)	0.007	1.47(1.13,1.91)	0.004	1.54(1.23,1.94)	<0.001	1.34(0.99,1.81)	0.054
**Myeloma**	**clioquinol***	2.49(1.30,4.75)	0.006	2.38(1.21,4.69)	0.012	2.52(1.32,4.80)	0.005	2.17(1.22,3.89)	0.009	2.32(1.12,4.81)	0.024
**Myeloma**	**paracetamol**	1.51(1.16,1.96)	0.002	1.38(1.05,1.81)	0.022	1.55(1.19,2.02)	0.001	1.76(1.40,2.20)	<0.001	1.70(1.25,2.31)	0.001
**Thyroid**	**clarithromycin**	2.10(1.28,3.46)	0.003	1.82(1.06,3.10)	0.029	2.14(1.30,3.52)	0.003	1.80(1.13,2.88)	0.014	1.70(0.95,3.05)	0.074
**Thyroid**	**flucloxacillin**	1.70(1.16,2.49)	0.006	1.84(1.22,2.77)	0.004	1.64(1.12,2.41)	0.012	1.63(1.14,2.32)	0.007	1.66(1.06,2.60)	0.027
**Thyroid**	**folic acid §**	2.77(1.32,5.82)	0.007	0.66(0.19,2.26)	0.506	3.02(1.42,6.41)	0.004	1.94(1.00,3.78)	0.052	2.17(0.91,5.17)	0.080
**Thyroid**	**gabapentin**	5.84(1.82,18.79)	0.003	5.17(1.36,19.66)	0.016	5.48(1.71,17.59)	0.004	5.03(1.65,15.31)	0.004	3.91(0.99,15.44)	0.052
**Thyroid**	**levothyroxine**	2.21(1.27,3.83)	0.005	2.57(1.43,4.61)	0.002	2.27(1.30,3.97)	0.004	2.07(1.29,3.32)	0.002	2.68(1.41,5.08)	0.003
**Thyroid**	**mefenamic acid**	2.30(1.25,4.25)	0.008	2.22(1.19,4.14)	0.012	2.24(1.21,4.15)	0.010	1.69(0.96,2.99)	0.068	1.98(0.98,4.01)	0.059
**Anal**	**clonidine**	4.49(1.61,12.50)	0.004	5.39(1.82,16.02)	0.002	6.44(2.12,19.56)	0.001	4.44(1.73,11.42)	0.002	7.33(2.17,24.82)	0.001
**Anal**	**doxazosin**	2.76(1.34,5.71)	0.006	3.01(1.37,6.59)	0.006	2.56(1.18,5.53)	0.017	2.56(1.30,5.03)	0.006	3.03(1.27,7.24)	0.013
**Anal**	**theophylline**	9.08(1.78,46.43)	0.008	5.65(1.04,30.59)	0.044	2.19(0.36,13.30)	0.394	4.36(1.19,15.89)	0.026	8.67(1.24,60.81)	0.030
**Signals associated with decreased cancer risk**											
**Breast §**	**trazodone**	0.74(0.62,0.89)	0.001	0.76(0.62,0.93)	0.009	0.75(0.62,0.89)	0.002	0.79(0.67,0.93)	0.005	0.75(0.62,0.92)	0.005
**Lung**	**fluocinolone***	0.65(0.48,0.89)	0.007	0.65(0.47,0.91)	0.013	0.66(0.49,0.90)	0.009	0.68(0.51,0.91)	0.009	0.66(0.47,0.92)	0.015
**Colorectal**	**metoclopramide**	0.77(0.65,0.91)	0.002	0.75(0.63,0.90)	0.002	0.79(0.67,0.92)	0.004	0.78(0.67,0.91)	0.001	0.78(0.65,0.93)	0.005

Table S6 (cont)

		1 year lag (main analysis)		2 year lag		Exclude matched sets where cases have additional cancer diagnosis within 12 months		1 year lag with multiple imputation		Adjusted for comorbidities smoking & alcohol	
**Cancer**	**Medicine**	**OR (95%CI)**	**p-value**	**OR (95%CI)**	**p-value**	**OR (95%CI)**	**p-value**	**OR (95%CI)**	**p-value**	**OR (95%CI)**	**p-value**
**Colorectal**	**nitrofurantoin**	0.66(0.51,0.87)	0.003	0.50(0.36,0.69)	<0.001	0.66(0.50,0.86)	0.002	0.71(0.56,0.90)	0.005	0.65(0.48,0.86)	0.003
**Prostate §§**	**levodopa**	0.44(0.26,0.75)	0.003	0.55(0.30,1.00)	0.048	0.44(0.26,0.75)	0.003	0.47(0.30,0.73)	0.001	0.44(0.25,0.80)	0.007
**Prostate §§**	**risperidone**	0.38(0.18,0.78)	0.009	0.61(0.29,1.29)	0.195	0.38(0.18,0.78)	0.009	0.37(0.20,0.70)	0.002	0.44(0.20,0.96)	0.039
**Prostate §§**	**senna**	0.77(0.66,0.90)	0.001	0.77(0.64,0.92)	0.004	0.77(0.66,0.90)	0.001	0.76(0.66,0.87)	<0.001	0.78(0.65,0.92)	0.004
**Bladder**	**sotalol**	0.19(0.07,0.52)	0.001	0.20(0.07,0.55)	0.002	0.16(0.05,0.51)	0.002	0.25(0.11,0.55)	0.001	0.13(0.04,0.44)	0.001
**Malignant melanoma**	**gliclazide**	0.49(0.30,0.80)	0.005	0.39(0.22,0.69)	0.001	0.50(0.30,0.82)	0.007	0.50(0.32,0.79)	0.003	0.41(0.24,0.69)	0.001
**Oesophagus**	**diazepam**	0.73(0.58,0.91)	0.005	0.73(0.57,0.93)	0.012	0.73(0.58,0.91)	0.005	0.77(0.63,0.95)	0.013	0.77(0.60,0.97)	0.030
**Oesophagus**	**estrogen (HRT) §**	0.61(0.44,0.84)	0.003	0.59(0.42,0.83)	0.003	0.62(0.44,0.86)	0.004	0.61(0.45,0.82)	0.001	0.56(0.39,0.81)	0.002
**Non-Hodgkin's lymphoma**	**progestogen (cont) §**	0.49(0.30,0.81)	0.005	0.41(0.24,0.71)	0.001	0.47(0.28,0.78)	0.003	0.56(0.36,0.86)	0.008	0.54(0.32,0.93)	0.027
**Oral (inc. Head,neck & nasal)**	**doxycycline**	0.43(0.28,0.67)	<0.001	0.43(0.27,0.69)	0.001	0.41(0.26,0.64)	<0.001	0.46(0.31,0.68)	<0.001	0.40(0.24,0.66)	<0.001
**Ovary §**	**progestogen (cont)**	0.56(0.38,0.81)	0.003	0.51(0.34,0.76)	0.001	0.56(0.38,0.82)	0.003	0.55(0.39,0.78)	0.001	0.47(0.30,0.72)	0.001
**Cervix & other gynae §**	**terbinafine**	0.20(0.07,0.54)	0.002	0.18(0.05,0.58)	0.004	0.21(0.07,0.58)	0.003	0.17(0.06,0.48)	0.001	0.19(0.07,0.54)	0.002
**Uterus §**	**progestogen (cont)**	0.37(0.19,0.71)	0.003	0.38(0.19,0.74)	0.005	0.38(0.20,0.73)	0.004	0.35(0.19,0.67)	0.001	0.40(0.20,0.80)	0.010

† comorbidities include diabetes, myocardial infarction, coronary heart disease, heart failure, peripheral vascular disease, dementia, cerebrovascular disease, chronic obstructive pulmonary disease, osteoporosis, rheumatological disease, renal disease, liver disease, irritable bowel disease, human immunodeficiency viruses and hemiplegia/paraplegia, plus potential site-specific confounders; OR odds ratio; CI confidence interval; CNS: central nervous system; medicines systemic unless otherwise indicated* (local); HRT: hormone replacement therapy; § females only; §§ males only

Table S7: Sensitivity analyses for comorbidity† adjusted signals: exposure >=6 prescriptions

		1 year lag (main analysis)		2 year lag		Exclude matched sets where cases have additional cancer diagnosis within 12 months		Adjusted for comorbidities, smoking & alcohol	
**Cancer**	**Medicine**	**OR (95%CI)**	**p-value**	**OR (95%CI)**	**p-value**	**OR (95%CI)**	**p-value**	**OR (95%CI)**	**p-value**
**Signals associated with increased cancer risk**									
**Breast §**	**estrogen-HRT**	1.36(1.27,1.45)	<0.001	1.31(1.22,1.41)	<0.001	1.35(1.26,1.45)	<0.001	1.32(1.21,1.43)	<0.001
**Breast §**	**metronidazole***	2.21(1.21,4.03)	0.010	2.86(1.47,5.56)	0.002	2.21(1.21,4.03)	0.010	2.10(1.04,4.24)	0.038
**Breast §**	**progestogen-HRT**	1.50(1.40,1.62)	<0.001	1.47(1.36,1.59)	<0.001	1.50(1.39,1.61)	<0.001	1.47(1.34,1.61)	<0.001
**Lung**	**amitriptyline**	1.27(1.13,1.43)	<0.001	1.24(1.09,1.40)	0.001	1.28(1.14,1.45)	<0.001	1.25(1.08,1.46)	0.003
**Lung**	**amoxicillin**	1.44(1.30,1.60)	<0.001	1.45(1.29,1.63)	<0.001	1.44(1.30,1.60)	<0.001	1.38(1.22,1.58)	<0.001
**Lung**	**azathioprine**	1.88(1.30,2.70)	0.001	1.75(1.18,2.59)	0.005	1.85(1.28,2.67)	0.001	2.42(1.51,3.87)	<0.001
**Lung**	**cimetidine**	1.46(1.29,1.66)	<0.001	1.45(1.28,1.65)	<0.001	1.48(1.31,1.67)	<0.001	1.40(1.19,1.65)	<0.001
**Lung**	**diazepam**	1.29(1.11,1.50)	0.001	1.35(1.15,1.58)	<0.001	1.29(1.11,1.50)	0.001	1.04(0.85,1.27)	0.698
**Lung**	**dihydrocodeine**	1.40(1.27,1.54)	<0.001	1.42(1.28,1.57)	<0.001	1.40(1.27,1.54)	<0.001	1.26(1.11,1.43)	<0.001
**Lung**	**folic acid §**	1.50(1.21,1.86)	<0.001	1.58(1.24,2.01)	<0.001	1.51(1.21,1.88)	<0.001	1.41(1.06,1.88)	0.018
**Lung**	**ipratropium***	1.63(1.45,1.84)	<0.001	1.58(1.39,1.80)	<0.001	1.65(1.46,1.86)	<0.001	1.56(1.33,1.83)	<0.001
**Lung**	**nitrazepam**	1.49(1.21,1.84)	<0.001	1.48(1.19,1.84)	<0.001	1.48(1.20,1.82)	<0.001	1.89(1.40,2.56)	<0.001
**Lung**	**quinine**	1.25(1.10,1.42)	0.001	1.22(1.06,1.40)	0.005	1.25(1.10,1.42)	<0.001	1.35(1.14,1.59)	<0.001
**Lung**	**salbutamol***	1.41(1.30,1.53)	<0.001	1.36(1.24,1.48)	<0.001	1.42(1.31,1.54)	<0.001	1.39(1.24,1.55)	<0.001
**Lung**	**salmeterol***	1.37(1.22,1.53)	<0.001	1.25(1.10,1.42)	0.001	1.37(1.22,1.54)	<0.001	1.42(1.23,1.65)	<0.001
**Lung**	**temazepam**	1.33(1.15,1.54)	<0.001	1.37(1.17,1.59)	<0.001	1.33(1.15,1.54)	<0.001	1.27(1.04,1.55)	0.018
**Lung**	**tiotropium***	1.64(1.37,1.97)	<0.001	1.59(1.27,1.99)	<0.001	1.66(1.39,1.99)	<0.001	1.54(1.24,1.91)	<0.001
**Lung**	**vitamin D***	1.45(1.10,1.91)	0.009	1.33(0.98,1.80)	0.065	1.45(1.10,1.92)	0.008	1.14(0.79,1.66)	0.477
**Colorectal**	**allopurinol**	1.35(1.16,1.57)	<0.001	1.37(1.17,1.61)	<0.001	1.31(1.12,1.53)	0.001	1.33(1.10,1.60)	0.004
**Colorectal**	**aminophylline**	1.93(1.31,2.83)	0.001	2.07(1.38,3.11)	<0.001	1.93(1.31,2.83)	0.001	2.38(1.40,4.05)	0.001
**Colorectal**	**bisacodyl**	1.79(1.24,2.59)	0.002	1.94(1.30,2.89)	0.001	1.84(1.27,2.66)	0.001	1.79(1.08,2.96)	0.023
**Colorectal**	**dipyridamole**	1.45(1.17,1.80)	0.001	1.54(1.22,1.95)	<0.001	1.46(1.18,1.81)	0.001	1.40(1.06,1.83)	0.016
**Colorectal**	**mesalazine**	1.65(1.18,2.30)	0.003	1.88(1.31,2.69)	0.001	1.66(1.18,2.33)	0.003	1.51(0.96,2.37)	0.071
**Colorectal**	**perindopril**	1.31(1.10,1.56)	0.003	1.31(1.07,1.60)	0.009	1.30(1.09,1.56)	0.004	1.31(1.06,1.61)	0.011
**Colorectal**	**phenytoin**	1.58(1.12,2.22)	0.009	1.56(1.09,2.22)	0.014	1.52(1.07,2.16)	0.018	1.63(1.01,2.61)	0.044
**Prostate §§**	**cerivastatin**	1.59(1.16,2.20)	0.004	1.57(1.12,2.21)	0.010	1.58(1.14,2.19)	0.006	1.60(1.08,2.36)	0.019
**Prostate §§**	**clioquinol***	2.06(1.24,3.42)	0.005	2.35(1.36,4.04)	0.002	2.06(1.24,3.42)	0.005	1.94(1.06,3.56)	0.032
**Bladder**	**amoxicillin**	1.39(1.13,1.72)	0.002	1.41(1.11,1.80)	0.005	1.39(1.12,1.71)	0.002	1.34(1.05,1.70)	0.019
**Bladder**	**nicotine**	2.59(1.38,4.85)	0.003	2.43(1.13,5.23)	0.023	2.43(1.28,4.63)	0.007	2.30(1.16,4.56)	0.017
**Bladder**	**trimethoprim**	1.80(1.25,2.58)	0.001	1.49(0.97,2.29)	0.066	1.83(1.27,2.65)	0.001	2.12(1.41,3.20)	<0.001
**Malignant melanoma**	**clopidogrel**	1.86(1.21,2.88)	0.005	1.83(1.10,3.05)	0.020	1.80(1.15,2.81)	0.010	1.78(1.08,2.93)	0.024
**Oesophagus**	**alginic acid**	1.52(1.26,1.84)	<0.001	1.52(1.25,1.86)	<0.001	1.54(1.27,1.86)	<0.001	1.51(1.17,1.94)	0.001
**Oesophagus**	**beclometasone***	1.37(1.14,1.65)	0.001	1.31(1.08,1.60)	0.007	1.37(1.13,1.66)	0.001	1.34(1.06,1.70)	0.016
**Oesophagus**	**cisapride**	2.89(1.41,5.92)	0.004	2.54(1.18,5.46)	0.017	2.90(1.42,5.95)	0.004	2.91(1.10,7.70)	0.031
**Oesophagus**	**lansoprazole**	1.67(1.39,2.01)	<0.001	1.69(1.38,2.06)	<0.001	1.65(1.37,1.99)	<0.001	1.52(1.21,1.91)	<0.001
**Oesophagus**	**omeprazole**	1.45(1.24,1.69)	<0.001	1.50(1.27,1.78)	<0.001	1.45(1.24,1.70)	<0.001	1.41(1.15,1.72)	0.001

Table S7 (cont)

		1 year lag (main analysis)		2 year lag		Exclude matched sets where cases have additional cancer diagnosis within 12 months		Adjusted for comorbidities, smoking & alcohol	
**Cancer**	**Medicine**	**OR (95%CI)**	**p-value**	**OR (95%CI)**	**p-value**	**OR (95%CI)**	**p-value**	**OR (95%CI)**	**p-value**
**Oesophagus**	**risedronate sodium**	2.54(1.27,5.05)	0.008	2.46(1.12,5.39)	0.025	2.43(1.20,4.90)	0.013	2.67(1.16,6.18)	0.021
**Oesophagus**	**vitamin B**	1.54(1.18,2.01)	0.001	1.65(1.24,2.20)	0.001	1.58(1.21,2.06)	0.001	1.33(0.94,1.87)	0.108
**Oesophagus**	**warfarin**	1.43(1.11,1.84)	0.005	1.41(1.07,1.85)	0.014	1.43(1.11,1.84)	0.006	1.52(1.10,2.09)	0.010
**Non-Hodgkin's lymphoma**	**amoxicillin**	1.46(1.12,1.92)	0.006	1.70(1.25,2.32)	0.001	1.52(1.15,2.00)	0.003	1.40(1.02,1.93)	0.039
**Non-Hodgkin's lymphoma**	**azathioprine**	2.58(1.31,5.08)	0.006	2.57(1.28,5.18)	0.008	2.66(1.35,5.25)	0.005	2.42(0.99,5.93)	0.053
**Non-Hodgkin's lymphoma**	**gabapentin**	2.86(1.47,5.58)	0.002	2.14(0.94,4.90)	0.070	2.97(1.51,5.81)	0.002	4.28(1.87,9.81)	0.001
**Non-Hodgkin's lymphoma**	**methotrexate**	3.41(1.57,7.41)	0.002	3.60(1.54,8.43)	0.003	3.41(1.57,7.43)	0.002	3.26(1.20,8.82)	0.020
**Non-Hodgkin's lymphoma**	**phenoxymethylpenicillin**	3.77(1.51,9.45)	0.005	4.09(1.47,11.39)	0.007	3.77(1.51,9.45)	0.005	3.39(0.93,12.37)	0.064
**Leukaemia**	**allopurinol**	1.68(1.24,2.27)	0.001	1.57(1.12,2.19)	0.009	1.65(1.21,2.24)	0.002	1.83(1.24,2.69)	0.002
**Leukaemia**	**oxybutynin**	2.35(1.41,3.92)	0.001	2.25(1.29,3.94)	0.004	2.35(1.41,3.93)	0.001	2.68(1.30,5.52)	0.007
**Leukaemia**	**oxytetracycline**	2.67(1.49,4.79)	0.001	3.19(1.74,5.84)	<0.001	2.52(1.39,4.56)	0.002	2.74(1.25,5.97)	0.011
**Leukaemia**	**paracetamol**	1.26(1.10,1.44)	0.001	1.22(1.05,1.41)	0.007	1.26(1.10,1.45)	0.001	1.36(1.13,1.63)	0.001
**Oral (inc. Head,neck & nasal)**	**betamethasone***	1.69(1.15,2.48)	0.007	1.63(1.07,2.48)	0.023	1.75(1.19,2.58)	0.005	2.13(1.30,3.48)	0.003
**Oral (inc. Head,neck & nasal)**	**folic acid §**	3.79(2.09,6.89)	<0.001	2.78(1.43,5.38)	0.002	3.66(1.99,6.72)	<0.001	3.55(1.51,8.33)	0.004
**Oral (inc. Head,neck & nasal)**	**vitamin A**	3.84(1.68,8.79)	0.001	2.90(1.10,7.63)	0.031	3.83(1.67,8.79)	0.002	3.81(1.25,11.55)	0.018
**Oral (inc. Head,neck & nasal)**	**vitamin B**	3.09(2.31,4.14)	<0.001	3.08(2.23,4.25)	<0.001	3.12(2.32,4.21)	<0.001	2.07(1.40,3.06)	<0.001
**Oral (inc. Head,neck & nasal)**	**vitamin C**	4.43(2.05,9.56)	<0.001	2.63(1.03,6.74)	0.044	4.72(2.14,10.38)	<0.001	3.73(1.29,10.81)	0.015
**Stomach**	**cimetidine**	1.52(1.11,2.07)	0.008	1.46(1.06,2.03)	0.022	1.55(1.13,2.13)	0.007	2.35(0.72,7.69)	0.157
**Ovary §**	**cyclopenthiazide**	3.31(1.54,7.15)	0.002	3.77(1.70,8.36)	0.001	3.33(1.54,7.18)	0.002	4.36(1.51,12.59)	0.006
**Ovary §**	**phenytoin**	3.87(1.83,8.18)	<0.001	3.48(1.62,7.47)	0.001	3.90(1.85,8.24)	<0.001	3.02(1.30,7.06)	0.011
**Ovary §**	**vitamin D***	2.66(1.30,5.45)	0.007	3.47(1.55,7.78)	0.002	2.84(1.38,5.85)	0.005	1.19(0.78,1.80)	0.425
**Kidney**	**hydrochlorothiazide**	2.26(1.46,3.48)	<0.001	2.55(1.63,4.00)	<0.001	2.34(1.51,3.61)	<0.001	2.28(1.35,3.85)	0.002
**Kidney**	**nystatin***	2.25(1.23,4.13)	0.009	2.45(1.30,4.62)	0.006	2.25(1.22,4.13)	0.009	1.76(0.86,3.61)	0.123
**Kidney**	**perindopril**	1.87(1.25,2.81)	0.003	1.98(1.26,3.13)	0.003	2.00(1.32,3.03)	0.001	1.74(1.09,2.77)	0.020
**Pancreas**	**clopidogrel**	2.14(1.34,3.42)	0.001	1.81(1.05,3.10)	0.032	2.07(1.29,3.33)	0.003	1.75(0.95,3.24)	0.075
**Pancreas**	**hydrochlorothiazide**	1.84(1.16,2.90)	0.009	1.72(1.08,2.75)	0.023	1.71(1.07,2.73)	0.025	2.49(1.45,4.30)	0.001
**Pancreas**	**metoclopramide**	3.08(1.40,6.76)	0.005	3.43(1.49,7.88)	0.004	3.04(1.38,6.70)	0.006	1.95(1.11,3.44)	0.021
**Pancreas**	**nicotine**	3.61(1.55,8.43)	0.003	3.38(1.12,10.22)	0.031	4.46(1.84,10.79)	0.001	2.24(0.84,5.97)	0.107
**Cervix & other gynae §**	**cimetidine**	1.87(1.17,2.98)	0.009	1.98(1.21,3.23)	0.007	1.79(1.10,2.92)	0.020	1.92(0.74,5.00)	0.183
**Uterus §**	**atenolol**	1.40(1.14,1.71)	0.001	1.42(1.15,1.75)	0.001	1.43(1.17,1.75)	0.001	1.29(1.01,1.65)	0.044
**Uterus §**	**bendroflumethiazide**	1.33(1.10,1.61)	0.003	1.35(1.11,1.65)	0.003	1.34(1.10,1.62)	0.003	1.33(1.06,1.68)	0.015
**Uterus §**	**bismuth***	6.56(1.64,26.17)	0.008	17.32(1.54,194.62)	0.021	6.73(1.69,26.90)	0.007	6.83(1.12,41.51)	0.037
**Uterus §**	**dextropropoxyphene**	1.43(1.11,1.83)	0.005	1.41(1.09,1.83)	0.010	1.38(1.07,1.78)	0.013	1.37(0.99,1.89)	0.057
**Uterus §**	**doxazosin**	2.21(1.43,3.43)	<0.001	2.22(1.37,3.61)	0.001	2.26(1.45,3.53)	<0.001	1.91(1.14,3.19)	0.014
**Uterus §**	**enalapril**	1.78(1.21,2.62)	0.003	2.02(1.35,3.01)	0.001	1.89(1.28,2.79)	0.001	2.49(1.54,4.03)	<0.001

Table S7 (cont)

		1 year lag (main analysis)		2 year lag		Exclude matched sets where cases have additional cancer diagnosis within 12 months		Adjusted for comorbidities, smoking & alcohol	
**Cancer**	**Medicine**	**OR (95%CI)**	**p-value**	**OR (95%CI)**	**p-value**	**OR (95%CI)**	**p-value**	**OR (95%CI)**	**p-value**
**Uterus §**	**losartan potassium**	2.00(1.24,3.23)	0.005	2.17(1.28,3.68)	0.004	2.01(1.24,3.25)	0.004	2.06(1.18,3.59)	0.011
**Uterus §**	**peru balsam***	6.56(1.64,26.17)	0.008	17.32(1.54,194.62)	0.021	6.73(1.69,26.90)	0.007	6.83(1.12,41.51)	0.037
**Uterus §**	**salicylic acid***	4.31(1.53,12.08)	0.006	8.12(2.44,26.96)	0.001	4.31(1.53,12.09)	0.006	7.75(2.20,27.28)	0.001
**Larynx**	**cimetidine**	2.12(1.36,3.32)	0.001	2.32(1.46,3.70)	<0.001	2.06(1.30,3.27)	0.002	1.87(0.81,4.32)	0.143
**Larynx**	**vitamin B**	2.20(1.41,3.41)	<0.001	2.16(1.33,3.51)	0.002	2.30(1.46,3.62)	<0.001	2.43(1.28,4.61)	0.007
**Brain and CNS**	**carbamazepine**	3.02(1.59,5.71)	0.001	2.80(1.43,5.48)	0.003	3.21(1.68,6.12)	<0.001	1.23(0.67,2.24)	0.504
**Myeloma**	**mometasone***	3.45(1.40,8.50)	0.007	3.26(1.18,8.99)	0.023	4.02(1.61,10.09)	0.003	2.54(0.70,9.16)	0.155
**Liver**	**furosemide**	1.98(1.35,2.91)	0.001	1.91(1.28,2.85)	0.002	2.08(1.41,3.07)	<0.001	2.46(1.48,4.10)	0.001
**Liver**	**mometasone***	3.82(1.42,10.26)	0.008	2.92(1.00,8.53)	0.050	3.82(1.42,10.27)	0.008	3.12(1.01,9.68)	0.049
**Liver**	**pravastatin**	2.26(1.24,4.11)	0.008	2.14(1.11,4.12)	0.023	2.26(1.24,4.13)	0.008	2.08(1.05,4.12)	0.036
**Thyroid**	**levothyroxine**	2.30(1.40,3.76)	0.001	2.42(1.42,4.12)	0.001	2.40(1.46,3.96)	0.001	2.46(1.26,4.80)	0.008
**Thyroid**	**progestogen-HRT §**	2.52(1.33,4.78)	0.005	2.32(1.15,4.65)	0.018	2.52(1.33,4.78)	0.005	1.99(0.88,4.54)	0.100
**Anal**	**doxazosin**	3.19(1.44,7.06)	0.004	2.98(1.22,7.30)	0.017	3.13(1.37,7.17)	0.007	2.74(1.01,7.39)	0.047
**Anal**	**nicotine**	9.95(2.22,44.53)	0.003	5.43(0.70,42.17)	0.106	9.34(1.86,46.78)	0.007	5.79(0.93,35.94)	0.059
**Signals associated with decreased cancer risk**									
**Breast §**	**carbamazepine**	0.70(0.55,0.90)	0.005	0.66(0.50,0.86)	0.003	0.71(0.56,0.91)	0.007	0.81(0.60,1.10)	0.172
**Breast §**	**iron**	0.78(0.67,0.92)	0.003	0.72(0.60,0.87)	<0.001	0.78(0.66,0.92)	0.003	0.72(0.59,0.89)	0.002
**Breast §**	**prednisolone**	0.78(0.66,0.92)	0.004	0.78(0.65,0.95)	0.012	0.78(0.66,0.93)	0.006	0.82(0.66,1.01)	0.061
**Breast §**	**trimethoprim**	0.72(0.57,0.91)	0.005	0.78(0.60,1.02)	0.069	0.72(0.57,0.91)	0.006	0.71(0.54,0.92)	0.011
**Lung**	**benzalkonium***	0.49(0.29,0.83)	0.008	0.45(0.24,0.82)	0.009	0.51(0.30,0.86)	0.012	0.62(0.31,1.24)	0.174
**Lung**	**dimeticone***	0.46(0.27,0.80)	0.005	0.41(0.22,0.77)	0.006	0.48(0.28,0.82)	0.008	0.56(0.27,1.15)	0.112
**Lung**	**levodopa**	0.38(0.24,0.61)	<0.001	0.32(0.18,0.56)	<0.001	0.36(0.22,0.58)	<0.001	0.47(0.26,0.85)	0.013
**Colorectal**	**diclofenac**	0.71(0.63,0.80)	<0.001	0.71(0.62,0.81)	<0.001	0.71(0.63,0.81)	<0.001	0.68(0.59,0.79)	<0.001
**Colorectal**	**lactulose**	0.78(0.68,0.90)	0.001	0.77(0.65,0.90)	0.001	0.78(0.68,0.91)	0.001	0.76(0.62,0.92)	0.005
**Colorectal**	**misoprostol**	0.69(0.53,0.88)	0.003	0.64(0.49,0.84)	0.001	0.70(0.54,0.89)	0.005	0.56(0.40,0.78)	0.001
**Colorectal**	**naproxen**	0.72(0.57,0.89)	0.003	0.66(0.52,0.85)	0.001	0.73(0.58,0.91)	0.005	0.72(0.56,0.94)	0.017
**Colorectal**	**senna**	0.73(0.60,0.90)	0.003	0.75(0.60,0.95)	0.015	0.72(0.59,0.89)	0.002	0.77(0.59,1.00)	0.047
**Prostate §§**	**benzalkonium***	0.36(0.17,0.77)	0.009	0.33(0.14,0.79)	0.013	0.38(0.18,0.82)	0.014	0.37(0.14,0.98)	0.044
**Prostate §§**	**bumetanide**	0.57(0.38,0.86)	0.007	0.50(0.32,0.78)	0.002	0.60(0.40,0.90)	0.013	0.75(0.46,1.20)	0.227
**Prostate §§**	**citalopram**	0.59(0.43,0.81)	0.001	0.57(0.39,0.82)	0.002	0.58(0.42,0.80)	0.001	0.59(0.41,0.86)	0.006
**Prostate §§**	**dimeticone***	0.36(0.17,0.77)	0.009	0.33(0.14,0.79)	0.013	0.38(0.18,0.82)	0.014	0.39(0.15,1.01)	0.053
**Prostate §§**	**furosemide**	0.70(0.61,0.79)	<0.001	0.68(0.59,0.78)	<0.001	0.70(0.62,0.79)	<0.001	0.74(0.63,0.87)	<0.001
**Prostate §§**	**iron**	0.72(0.57,0.92)	0.008	0.64(0.48,0.86)	0.003	0.71(0.56,0.90)	0.006	0.76(0.56,1.04)	0.084
**Prostate §§**	**lactulose**	0.68(0.57,0.81)	<0.001	0.70(0.57,0.86)	0.001	0.67(0.56,0.81)	<0.001	0.70(0.56,0.88)	0.003
**Prostate §§**	**lidocaine***	0.35(0.16,0.75)	0.007	0.37(0.16,0.85)	0.019	0.35(0.16,0.75)	0.007	0.52(0.20,1.31)	0.166
**Prostate §§**	**risperidone**	0.27(0.11,0.70)	0.007	0.21(0.06,0.71)	0.012	0.29(0.11,0.75)	0.011	0.31(0.09,1.08)	0.065

Table S7 (cont)

		1 year lag (main analysis)		2 year lag		Exclude matched sets where cases have additional cancer diagnosis within 12 months		Adjusted for comorbidities, smoking & alcohol	
**Cancer**	**Medicine**	**OR (95%CI)**	**p-value**	**OR (95%CI)**	**p-value**	**OR (95%CI)**	**p-value**	**OR (95%CI)**	**p-value**
**Prostate §§**	**senna**	0.70(0.55,0.90)	0.005	0.69(0.52,0.92)	0.012	0.70(0.54,0.90)	0.006	0.73(0.54,0.99)	0.046
**Prostate §§**	**vitamin D**	0.66(0.50,0.88)	0.004	0.66(0.47,0.94)	0.019	0.64(0.48,0.86)	0.003	0.62(0.44,0.87)	0.005
**Bladder**	**iron**	0.64(0.47,0.88)	0.006	0.65(0.46,0.93)	0.018	0.64(0.46,0.87)	0.005	0.63(0.42,0.95)	0.028
**Bladder**	**lactulose**	0.73(0.58,0.91)	0.006	0.70(0.54,0.90)	0.006	0.72(0.57,0.90)	0.005	0.81(0.60,1.07)	0.140
**Malignant melanoma**	**gliclazide**	0.51(0.31,0.84)	0.009	0.60(0.36,1.03)	0.062	0.53(0.32,0.88)	0.014	0.45(0.25,0.80)	0.007
**Non-Hodgkin's lymphoma**	**vitamin B**	0.51(0.34,0.78)	0.002	0.55(0.35,0.86)	0.009	0.52(0.34,0.79)	0.002	0.58(0.34,0.98)	0.041
**Ovary §**	**nifedipine**	0.56(0.38,0.83)	0.004	0.62(0.41,0.93)	0.021	0.57(0.39,0.85)	0.006	0.59(0.37,0.96)	0.034
**Ovary §**	**progestogen-contraceptive**	0.44(0.23,0.81)	0.009	0.33(0.16,0.69)	0.003	0.46(0.25,0.87)	0.016	0.50(0.26,0.99)	0.047
**Stomach**	**finasteride §§**	0.41(0.21,0.81)	0.010	0.32(0.15,0.70)	0.004	0.42(0.21,0.82)	0.012	0.43(0.19,0.96)	0.038
**Uterus §**	**estrogen-HRT**	0.68(0.53,0.87)	0.002	0.72(0.56,0.93)	0.012	0.68(0.53,0.88)	0.003	0.75(0.56,0.99)	0.045
**Larynx**	**diclofenac**	0.48(0.29,0.78)	0.003	0.53(0.31,0.90)	0.019	0.49(0.30,0.81)	0.006	0.62(0.33,1.15)	0.128
**Larynx**	**doxazosin**	0.31(0.14,0.71)	0.006	0.33(0.14,0.80)	0.014	0.31(0.14,0.71)	0.006	0.37(0.13,1.06)	0.064

† comorbidities include diabetes, myocardial infarction, coronary heart disease, heart failure, peripheral vascular disease, dementia, cerebrovascular disease, chronic obstructive pulmonary disease, osteoporosis, rheumatological disease, renal disease, liver disease, irritable bowel disease, human immunodeficiency viruses and hemiplegia/paraplegia, plus potential site-specific confounders; OR odds ratio; CI confidence interval; CNS: central nervous system; medicines systemic unless otherwise indicated* (local); HRT: hormone replacement therapy; § females only; §§ males only

Table S8: Sensitivity analyses for comorbidity† & smoking adjusted signals: exposure >=6 prescriptions

		1 year lag (main analysis)		2 year lag		Exclude matched sets where cases have additional cancer diagnosis within 12 months		1 year lag with multiple imputation		Adjusted for comorbidities smoking & alcohol	
**Cancer**	**Medicine**	**OR (95%CI)**	**p-value**	**OR (95%CI)**	**p-value**	**OR (95%CI)**	**p-value**	**OR (95%CI)**	**p-value**	**OR (95%CI)**	**p-value**
**Signals associated with increased cancer risk**											
**Breast §**	**estrogen (HRT)**	1.32(1.23,1.43)	<0.001	1.30(1.20,1.41)	<0.001	1.32(1.23,1.43)	<0.001	1.36(1.27,1.45)	<0.001	1.32(1.21,1.43)	<0.001
**Breast §**	**metronidazole***	2.36(1.25,4.46)	0.008	3.46(1.66,7.21)	0.001	2.36(1.25,4.46)	0.008	2.20(1.20,4.01)	0.010	2.10(1.04,4.24)	0.038
**Breast §**	**progestogen (HRT)**	1.48(1.37,1.61)	<0.001	1.45(1.32,1.58)	<0.001	1.48(1.37,1.61)	<0.001	1.50(1.40,1.62)	<0.001	1.47(1.34,1.61)	<0.001
**Lung**	**amitriptyline**	1.25(1.09,1.44)	0.001	1.22(1.05,1.41)	0.010	1.27(1.10,1.45)	0.001	1.19(1.05,1.35)	0.008	1.26(1.08,1.46)	0.003
**Lung**	**amoxicillin**	1.36(1.21,1.53)	<0.001	1.38(1.21,1.58)	<0.001	1.36(1.20,1.53)	<0.001	1.39(1.24,1.56)	<0.001	1.38(1.21,1.57)	<0.001
**Lung**	**azathioprine**	2.30(1.49,3.56)	<0.001	2.36(1.45,3.83)	0.001	2.24(1.44,3.47)	<0.001	2.11(1.43,3.11)	<0.001	2.46(1.54,3.96)	<0.001
**Lung**	**cimetidine**	1.37(1.18,1.58)	<0.001	1.26(1.07,1.47)	0.004	1.38(1.19,1.60)	<0.001	1.32(1.16,1.51)	<0.001	1.41(1.20,1.66)	<0.001
**Lung**	**dihydrocodeine**	1.28(1.15,1.44)	<0.001	1.30(1.15,1.47)	<0.001	1.28(1.14,1.43)	<0.001	1.31(1.18,1.45)	<0.001	1.26(1.11,1.43)	<0.001
**Lung**	**fluticasone***	1.41(1.24,1.61)	<0.001	1.35(1.16,1.56)	<0.001	1.42(1.24,1.62)	<0.001	1.40(1.23,1.58)	<0.001	1.38(1.19,1.60)	<0.001
**Lung**	**ipratropium***	1.56(1.35,1.79)	<0.001	1.55(1.33,1.81)	<0.001	1.59(1.38,1.83)	<0.001	1.56(1.37,1.77)	<0.001	1.56(1.33,1.83)	<0.001
**Lung**	**nitrazepam**	1.58(1.22,2.05)	0.001	1.66(1.27,2.19)	<0.001	1.58(1.22,2.04)	0.001	1.44(1.15,1.81)	0.002	1.85(1.36,2.51)	<0.001
**Lung**	**phenoxymethylpenicillin**	2.89(1.52,5.50)	0.001	2.96(1.47,5.97)	0.002	2.89(1.52,5.51)	0.001	2.05(1.14,3.68)	0.016	3.47(1.73,6.99)	<0.001
**Lung**	**quinine**	1.31(1.13,1.52)	<0.001	1.32(1.12,1.56)	0.001	1.32(1.13,1.53)	<0.001	1.28(1.12,1.47)	<0.001	1.35(1.14,1.59)	<0.001
**Lung**	**salbutamol***	1.36(1.24,1.50)	<0.001	1.30(1.17,1.44)	<0.001	1.37(1.25,1.51)	<0.001	1.39(1.27,1.52)	<0.001	1.38(1.24,1.54)	<0.001
**Lung**	**salmeterol***	1.51(1.32,1.72)	<0.001	1.38(1.19,1.60)	<0.001	1.52(1.33,1.73)	<0.001	1.50(1.33,1.70)	<0.001	1.42(1.23,1.65)	<0.001
**Lung**	**tiotropium***	1.52(1.25,1.85)	<0.001	1.44(1.13,1.84)	0.003	1.54(1.26,1.87)	<0.001	1.54(1.28,1.87)	<0.001	1.52(1.22,1.88)	<0.001
**Colorectal**	**allopurinol**	1.33(1.12,1.59)	0.001	1.34(1.11,1.62)	0.003	1.29(1.08,1.53)	0.005	1.33(1.14,1.55)	<0.001	1.33(1.10,1.61)	0.004
**Colorectal**	**aminophylline**	1.89(1.17,3.05)	0.009	2.07(1.26,3.42)	0.004	1.88(1.16,3.03)	0.010	1.87(1.27,2.75)	0.001	2.39(1.40,4.06)	0.001
**Colorectal**	**dipyridamole**	1.44(1.13,1.83)	0.004	1.50(1.14,1.97)	0.004	1.44(1.12,1.83)	0.004	1.44(1.16,1.78)	0.001	1.40(1.07,1.84)	0.016
**Colorectal**	**tiotropium***	1.49(1.11,1.99)	0.007	1.21(0.83,1.77)	0.314	1.51(1.13,2.03)	0.005	1.34(1.01,1.78)	0.040	1.43(1.04,1.96)	0.028
**Prostate §§**	**alfuzosin**	1.63(1.25,2.13)	<0.001	1.55(1.13,2.12)	0.006	1.55(1.18,2.04)	0.002	1.66(1.31,2.10)	<0.001	1.71(1.28,2.29)	<0.001
**Prostate §§**	**atorvastatin**	1.25(1.11,1.42)	<0.001	1.29(1.12,1.48)	<0.001	1.26(1.11,1.43)	<0.001	1.25(1.11,1.40)	<0.001	1.26(1.10,1.44)	0.001
**Prostate §§**	**clioquinol***	2.09(1.19,3.66)	0.010	2.69(1.45,4.99)	0.002	2.09(1.20,3.67)	0.010	2.08(1.25,3.45)	0.005	1.94(1.06,3.57)	0.032
**Bladder**	**amoxicillin**	1.45(1.16,1.81)	0.001	1.45(1.11,1.87)	0.005	1.46(1.16,1.83)	0.001	1.37(1.12,1.69)	0.003	1.33(1.04,1.69)	0.022
**Bladder**	**cefalexin**	1.99(1.23,3.20)	0.005	1.95(1.11,3.46)	0.021	1.97(1.21,3.20)	0.006	1.83(1.17,2.88)	0.009	1.84(1.07,3.15)	0.027
**Bladder**	**celecoxib**	1.78(1.20,2.63)	0.004	1.87(1.20,2.92)	0.006	1.74(1.17,2.60)	0.007	1.59(1.09,2.32)	0.015	1.63(1.06,2.50)	0.027
**Bladder**	**ranitidine**	1.28(1.07,1.54)	0.008	1.30(1.06,1.59)	0.012	1.26(1.04,1.52)	0.017	1.24(1.05,1.46)	0.010	1.36(1.11,1.66)	0.003
**Bladder**	**trimethoprim**	2.04(1.39,3.00)	<0.001	1.78(1.12,2.84)	0.015	2.07(1.40,3.06)	<0.001	1.82(1.27,2.63)	0.001	2.14(1.42,3.23)	<0.001
**Oesophagus**	**alginic acid**	1.53(1.22,1.91)	<0.001	1.65(1.29,2.11)	<0.001	1.54(1.22,1.93)	<0.001	1.53(1.26,1.85)	<0.001	1.50(1.17,1.93)	0.001
**Oesophagus**	**azathioprine**	4.15(1.87,9.22)	<0.001	4.41(1.80,10.80)	0.001	4.13(1.86,9.16)	<0.001	2.86(1.43,5.73)	0.003	3.08(1.19,7.97)	0.020
**Oesophagus**	**lansoprazole**	1.59(1.30,1.95)	<0.001	1.55(1.23,1.95)	<0.001	1.57(1.28,1.93)	<0.001	1.63(1.36,1.97)	<0.001	1.54(1.22,1.93)	<0.001
**Oesophagus**	**omeprazole**	1.40(1.17,1.66)	<0.001	1.45(1.19,1.77)	<0.001	1.39(1.16,1.66)	<0.001	1.46(1.24,1.71)	<0.001	1.42(1.16,1.73)	0.001
**Oesophagus**	**prednisolone**	1.68(1.19,2.38)	0.003	1.50(1.02,2.21)	0.037	1.69(1.18,2.41)	0.004	1.25(0.92,1.71)	0.157	1.70(1.16,2.50)	0.007
**Oesophagus**	**risedronate sodium**	2.69(1.28,5.67)	0.009	3.11(1.30,7.46)	0.011	2.53(1.18,5.41)	0.017	2.68(1.32,5.41)	0.006	2.67(1.16,6.16)	0.021

Table S8 (cont)

		1 year lag (main analysis)		2 year lag		Exclude matched sets where cases have additional cancer diagnosis within 12 months		1 year lag with multiple imputation		Adjusted for comorbidities smoking & alcohol	
**Cancer**	**Medicine**	**OR (95%CI)**	**p-value**	**OR (95%CI)**	**p-value**	**OR (95%CI)**	**p-value**	**OR (95%CI)**	**p-value**	**OR (95%CI)**	**p-value**
**Non-Hodgkin's lymphoma**	**amoxicillin**	1.50(1.13,2.00)	0.006	1.77(1.28,2.46)	0.001	1.57(1.17,2.09)	0.002	1.45(1.11,1.91)	0.007	1.40(1.02,1.93)	0.039
**Non-Hodgkin's lymphoma**	**gabapentin**	3.41(1.67,6.96)	0.001	2.72(1.13,6.57)	0.026	3.54(1.72,7.28)	0.001	2.83(1.45,5.51)	0.002	4.27(1.86,9.78)	0.001
**Leukaemia**	**metoclopramide**	2.61(1.34,5.11)	0.005	2.31(1.05,5.08)	0.037	2.74(1.40,5.40)	0.003	2.09(1.15,3.78)	0.015	2.48(1.23,5.00)	0.011
**Leukaemia**	**oxybutynin**	2.69(1.42,5.08)	0.002	2.50(1.26,4.99)	0.009	2.67(1.42,5.05)	0.002	2.35(1.41,3.93)	0.001	2.68(1.30,5.52)	0.007
**Leukaemia**	**oxytetracycline**	3.01(1.49,6.07)	0.002	3.05(1.45,6.42)	0.003	2.83(1.38,5.80)	0.004	2.66(1.48,4.78)	0.001	2.74(1.25,5.98)	0.011
**Leukaemia**	**paracetamol**	1.34(1.14,1.57)	<0.001	1.29(1.08,1.54)	0.005	1.34(1.14,1.58)	<0.001	1.26(1.10,1.44)	0.001	1.36(1.13,1.63)	0.001
**Oral (inc. Head,neck & nasal)**	**hydrochlorothiazide**	2.56(1.34,4.89)	0.005	2.54(1.27,5.09)	0.009	2.61(1.36,5.00)	0.004	2.29(1.29,4.08)	0.005	2.45(1.19,5.04)	0.015
**Oral (inc. Head,neck & nasal)**	**hydrocortisone***	1.66(1.17,2.35)	0.004	1.38(0.93,2.06)	0.113	1.66(1.18,2.36)	0.004	1.50(1.09,2.06)	0.014	1.68(1.14,2.46)	0.009
**Oral (inc. Head,neck & nasal)**	**vitamin B**	2.55(1.79,3.63)	<0.001	2.60(1.76,3.85)	<0.001	2.60(1.81,3.74)	<0.001	2.60(1.92,3.54)	<0.001	2.07(1.40,3.07)	<0.001
**Oral (inc. Head,neck & nasal)**	**vitamin C**	3.63(1.42,9.25)	0.007	1.89(0.62,5.83)	0.265	3.61(1.41,9.25)	0.007	3.84(1.73,8.51)	0.001	3.74(1.29,10.84)	0.015
**Ovary §**	**phenytoin**	4.31(1.74,10.65)	0.002	3.40(1.30,8.89)	0.013	4.31(1.74,10.66)	0.002	3.83(1.81,8.10)	<0.001	4.34(1.50,12.54)	0.007
**Stomach**	**chlorphenamine**	4.72(1.64,13.56)	0.004	3.56(1.19,10.65)	0.023	3.85(1.26,11.75)	0.018	2.99(1.25,7.16)	0.014	4.51(1.30,15.65)	0.018
**Kidney**	**hydrochlorothiazide**	2.61(1.63,4.18)	<0.001	3.32(2.00,5.53)	<0.001	2.73(1.70,4.40)	<0.001	2.30(1.49,3.55)	<0.001	2.29(1.35,3.87)	0.002
**Kidney**	**perindopril**	1.94(1.25,2.99)	0.003	2.00(1.22,3.29)	0.006	2.10(1.35,3.28)	0.001	1.88(1.25,2.83)	0.003	1.73(1.09,2.76)	0.021
**Pancreas**	**clopidogrel**	2.37(1.45,3.86)	0.001	1.86(1.05,3.29)	0.033	2.27(1.38,3.73)	0.001	2.04(1.27,3.27)	0.003	2.49(1.44,4.29)	0.001
**Pancreas**	**lorazepam**	3.59(1.41,9.18)	0.008	2.74(1.02,7.35)	0.046	3.58(1.40,9.16)	0.008	2.96(1.27,6.92)	0.012	4.06(1.32,12.52)	0.015
**Pancreas**	**methotrexate**	4.39(1.57,12.26)	0.005	3.93(1.33,11.62)	0.013	4.26(1.52,11.90)	0.006	3.23(1.20,8.69)	0.020	5.16(1.49,17.95)	0.010
**Pancreas**	**quinine**	1.78(1.18,2.68)	0.006	1.51(0.95,2.38)	0.079	1.71(1.13,2.60)	0.011	1.57(1.08,2.27)	0.017	1.76(1.11,2.80)	0.016
**Uterus §**	**bendroflumethiazide**	1.32(1.07,1.63)	0.009	1.29(1.03,1.61)	0.027	1.32(1.06,1.63)	0.011	1.29(1.07,1.57)	0.008	1.34(1.06,1.68)	0.014
**Uterus §**	**doxazosin**	1.88(1.18,3.00)	0.008	2.11(1.26,3.54)	0.005	1.84(1.15,2.95)	0.011	2.17(1.40,3.36)	0.001	1.91(1.15,3.19)	0.013
**Uterus §**	**enalapril**	2.35(1.53,3.60)	<0.001	2.62(1.69,4.06)	<0.001	2.53(1.64,3.89)	<0.001	1.78(1.21,2.62)	0.004	2.49(1.54,4.02)	<0.001
**Uterus §**	**loperamide**	2.88(1.36,6.09)	0.006	3.48(1.54,7.91)	0.003	2.61(1.21,5.64)	0.015	2.30(1.16,4.55)	0.017	3.09(1.35,7.08)	0.008
**Uterus §**	**salicylic acid***	6.85(2.15,21.83)	0.001	9.95(2.73,36.24)	<0.001	6.92(2.16,22.16)	0.001	4.33(1.53,12.23)	0.006	7.79(2.23,27.23)	0.001
**Myeloma**	**chlortalidone**	3.06(1.32,7.12)	0.009	3.26(1.38,7.72)	0.007	3.00(1.29,6.96)	0.011	1.96(0.94,4.08)	0.071	3.34(1.28,8.72)	0.014
**Liver**	**furosemide**	2.48(1.58,3.88)	<0.001	2.45(1.50,4.01)	<0.001	2.57(1.63,4.05)	<0.001	2.05(1.39,3.02)	<0.001	2.49(1.50,4.14)	<0.001
**Thyroid**	**progestogen (HRT) §**	2.55(1.27,5.11)	0.008	2.22(1.05,4.68)	0.036	2.54(1.26,5.09)	0.009	2.48(1.30,4.74)	0.006	1.97(0.86,4.48)	0.108
**Anal**	**bisoprolol**	3.30(1.34,8.14)	0.010	2.81(1.08,7.34)	0.035	2.71(1.03,7.17)	0.044	2.79(1.18,6.58)	0.019	2.41(0.85,6.83)	0.098
**Anal**	**nicotine**	8.07(1.69,38.46)	0.009	4.37(0.53,36.35)	0.173	8.36(1.47,47.39)	0.016	7.75(1.70,35.29)	0.008	5.80(0.94,35.87)	0.059
**Signals associated with decreased cancer risk**											
**Breast §**	**iron**	0.72(0.60,0.86)	<0.001	0.67(0.54,0.82)	<0.001	0.72(0.60,0.86)	<0.001	0.78(0.67,0.92)	0.002	0.72(0.59,0.89)	0.002
**Breast §**	**trazodone**	0.62(0.46,0.85)	0.003	0.64(0.44,0.91)	0.014	0.62(0.46,0.85)	0.003	0.72(0.55,0.94)	0.016	0.66(0.47,0.92)	0.013

Table S8 (cont)

		1 year lag (main analysis)		2 year lag		Exclude matched sets where cases have additional cancer diagnosis within 12 months		1 year lag with multiple imputation		Adjusted for comorbidities smoking & alcohol	
**Cancer**	**Medicine**	**OR (95%CI)**	**p-value**	**OR (95%CI)**	**p-value**	**OR (95%CI)**	**cancer**	**medicine**	**OR (95%CI)**	**p-value**	**OR (95%CI)**
**Breast §**	**trimethoprim**	0.71(0.55,0.90)	0.005	0.75(0.57,1.00)	0.051	0.71(0.55,0.90)	0.006	0.72(0.57,0.91)	0.005	0.71(0.54,0.92)	0.011
**Colorectal**	**diclofenac**	0.72(0.63,0.83)	<0.001	0.71(0.61,0.82)	<0.001	0.72(0.63,0.83)	<0.001	0.71(0.63,0.80)	<0.001	0.68(0.59,0.79)	<0.001
**Colorectal**	**lactulose**	0.77(0.65,0.91)	0.003	0.81(0.67,0.98)	0.033	0.78(0.65,0.92)	0.004	0.78(0.68,0.90)	0.001	0.76(0.62,0.92)	0.005
**Colorectal**	**naproxen**	0.71(0.56,0.91)	0.007	0.63(0.48,0.84)	0.001	0.73(0.57,0.93)	0.011	0.71(0.57,0.89)	0.003	0.72(0.55,0.94)	0.016
**Colorectal**	**oxybutynin**	0.60(0.42,0.87)	0.006	0.65(0.44,0.95)	0.028	0.60(0.42,0.87)	0.007	0.71(0.52,0.95)	0.023	0.57(0.38,0.87)	0.009
**Prostate §§**	**calcium**	0.67(0.51,0.89)	0.006	0.71(0.51,0.99)	0.046	0.68(0.51,0.90)	0.008	0.74(0.57,0.95)	0.020	0.72(0.53,0.98)	0.038
**Prostate §§**	**citalopram**	0.53(0.37,0.75)	<0.001	0.59(0.39,0.87)	0.008	0.52(0.36,0.74)	<0.001	0.58(0.43,0.80)	0.001	0.59(0.41,0.86)	0.006
**Prostate §§**	**furosemide**	0.74(0.64,0.85)	<0.001	0.73(0.62,0.86)	<0.001	0.75(0.65,0.86)	<0.001	0.69(0.61,0.78)	<0.001	0.74(0.63,0.86)	<0.001
**Prostate §§**	**lactulose**	0.70(0.57,0.86)	0.001	0.73(0.58,0.93)	0.010	0.69(0.56,0.85)	0.001	0.68(0.57,0.81)	<0.001	0.70(0.56,0.88)	0.003
**Prostate §§**	**senna**	0.67(0.50,0.90)	0.007	0.73(0.53,1.02)	0.069	0.67(0.50,0.90)	0.007	0.70(0.54,0.90)	0.005	0.73(0.54,0.99)	0.046
**Prostate §§**	**vitamin D**	0.60(0.44,0.82)	0.001	0.61(0.41,0.89)	0.011	0.59(0.43,0.81)	0.001	0.66(0.50,0.88)	0.004	0.62(0.44,0.87)	0.005
**Bladder**	**pantoprazole**	0.27(0.10,0.71)	0.008	0.31(0.10,0.92)	0.035	0.27(0.10,0.71)	0.008	0.37(0.15,0.91)	0.030	0.31(0.11,0.86)	0.024
**Bladder**	**sotalol**	0.25(0.09,0.71)	0.010	0.20(0.06,0.68)	0.010	0.21(0.06,0.68)	0.010	0.36(0.16,0.81)	0.014	0.18(0.05,0.61)	0.006
**Oesophagus**	**aspirin high dose**	0.52(0.33,0.83)	0.006	0.53(0.32,0.88)	0.014	0.55(0.35,0.88)	0.013	0.59(0.40,0.87)	0.007	0.59(0.36,0.97)	0.038
**Non-Hodgkin's lymphoma**	**vitamin B**	0.51(0.32,0.81)	0.004	0.58(0.35,0.95)	0.029	0.50(0.31,0.80)	0.004	0.51(0.33,0.77)	0.002	0.58(0.34,0.97)	0.040
**Leukaemia**	**calcium**	0.56(0.37,0.85)	0.007	0.40(0.24,0.68)	0.001	0.57(0.38,0.88)	0.010	0.68(0.48,0.97)	0.034	0.55(0.35,0.87)	0.011
**Stomach**	**diclofenac***	0.18(0.05,0.63)	0.007	0.23(0.07,0.78)	0.019	0.20(0.06,0.69)	0.011	0.41(0.17,0.98)	0.045	0.20(0.06,0.72)	0.014
**Kidney**	**digoxin**	0.43(0.23,0.80)	0.008	0.41(0.20,0.84)	0.014	0.39(0.20,0.75)	0.005	0.58(0.35,0.95)	0.031	0.44(0.22,0.88)	0.020

† comorbidities include diabetes, myocardial infarction, coronary heart disease, heart failure, peripheral vascular disease, dementia, cerebrovascular disease, chronic obstructive pulmonary disease, osteoporosis, rheumatological disease, renal disease, liver disease, irritable bowel disease, human immunodeficiency viruses and hemiplegia/paraplegia, plus potential site-specific confounders; OR odds ratio; CI confidence interval; medicines systemic unless otherwise indicated* (local); HRT: hormone replacement therapy; § females only; §§ males only
