# Supplementary material for: A systematic assessment of the association between frequently prescribed medicines and the risk of common cancers: a series of nested case-control studies
Source: BMC Med. 2021 Jan 26;19:22. doi: 10.1186/s12916-020-01891-5 (PMC7836181; doi:10.1186/s12916-020-01891-5)
Supplement: Supplementary file 3 — Additional file 3: Fig. S1. MWAS plots for comorbidity adjusted analyses: exposure any prescription. Fig. S2. MWAS plots for comorbidity & smoking adjusted analyses: exposure any prescription. [file 12916_2020_1891_MOESM3_ESM.pdf]

Supplementary Figures

Index of Legends

Fig S1: MWAS plots for comorbidity adjusted analyses: exposure any prescription

Fig S2: MWAS plots for comorbidity & smoking adjusted analyses: exposure any prescription

Fig S1: MWAS plots for comorbidity adjusted analyses: exposure any prescription

MWAS plot: breast cancer
Comorbidity adjusted analysis (exposure any prescription)

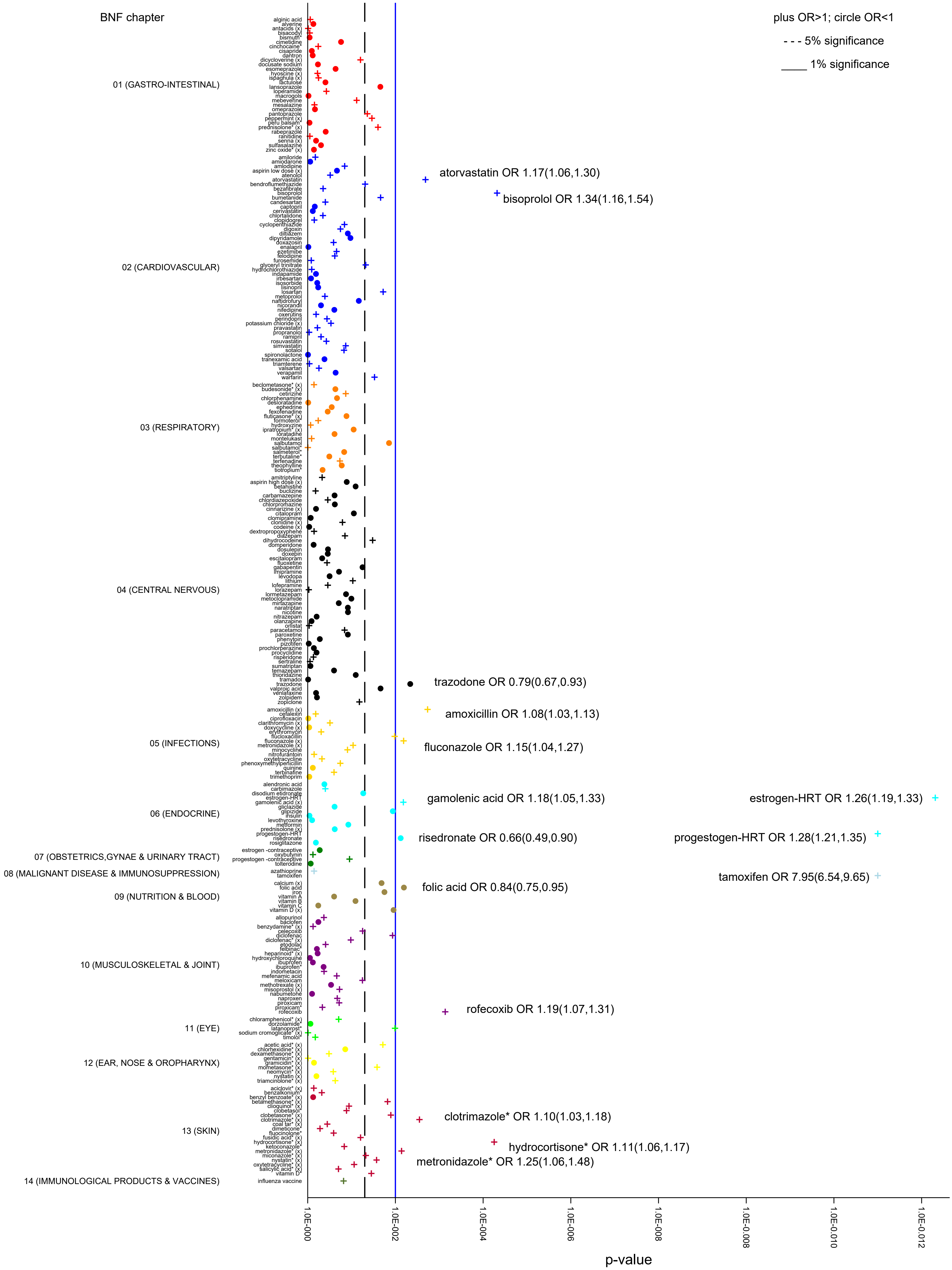

* : local medicine; females only included in this analysis; (l): local; (x) most prevalent chapter

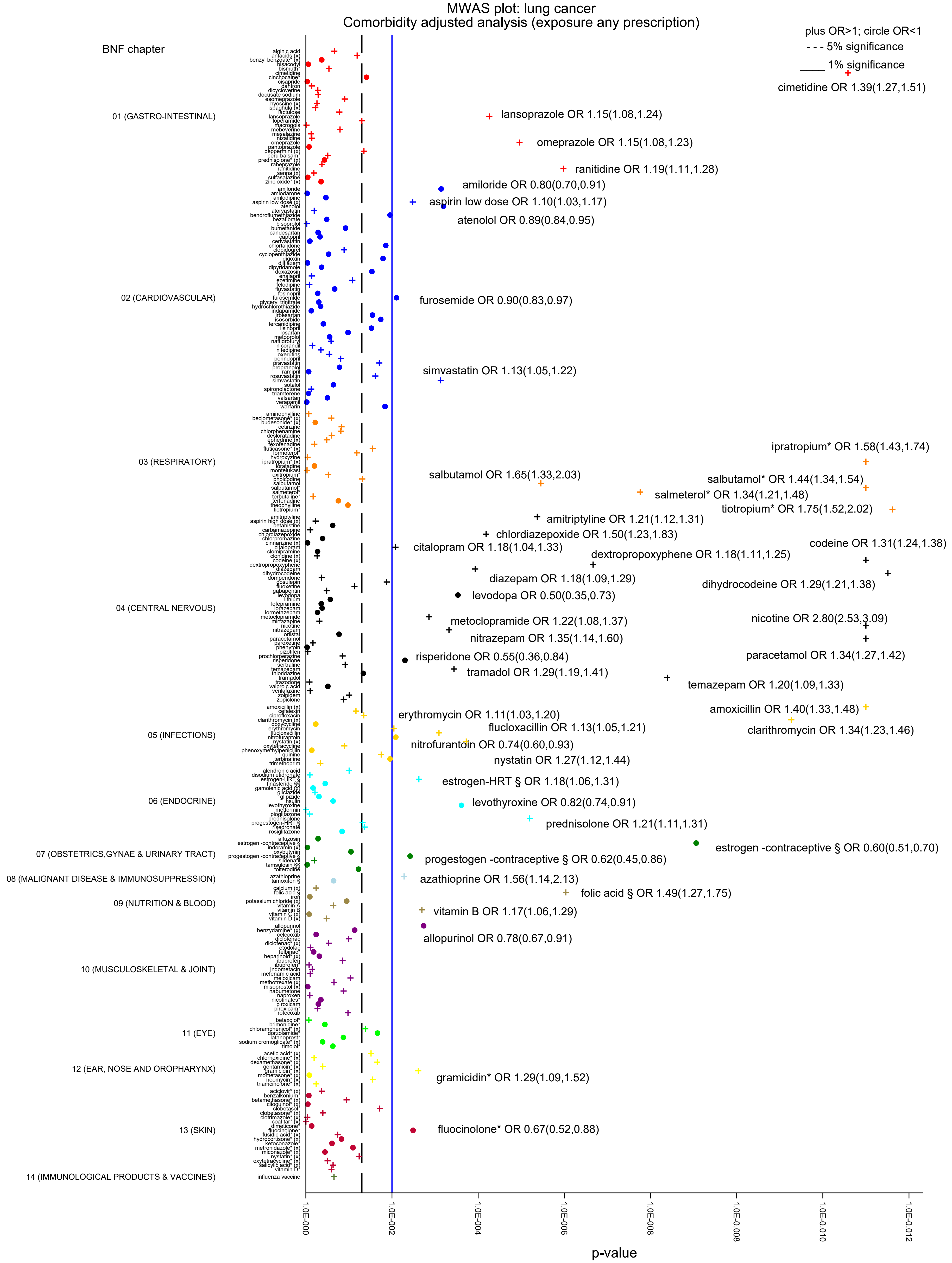

* : local medicine; § females only; §§ males only; (x) most prevalent chapter

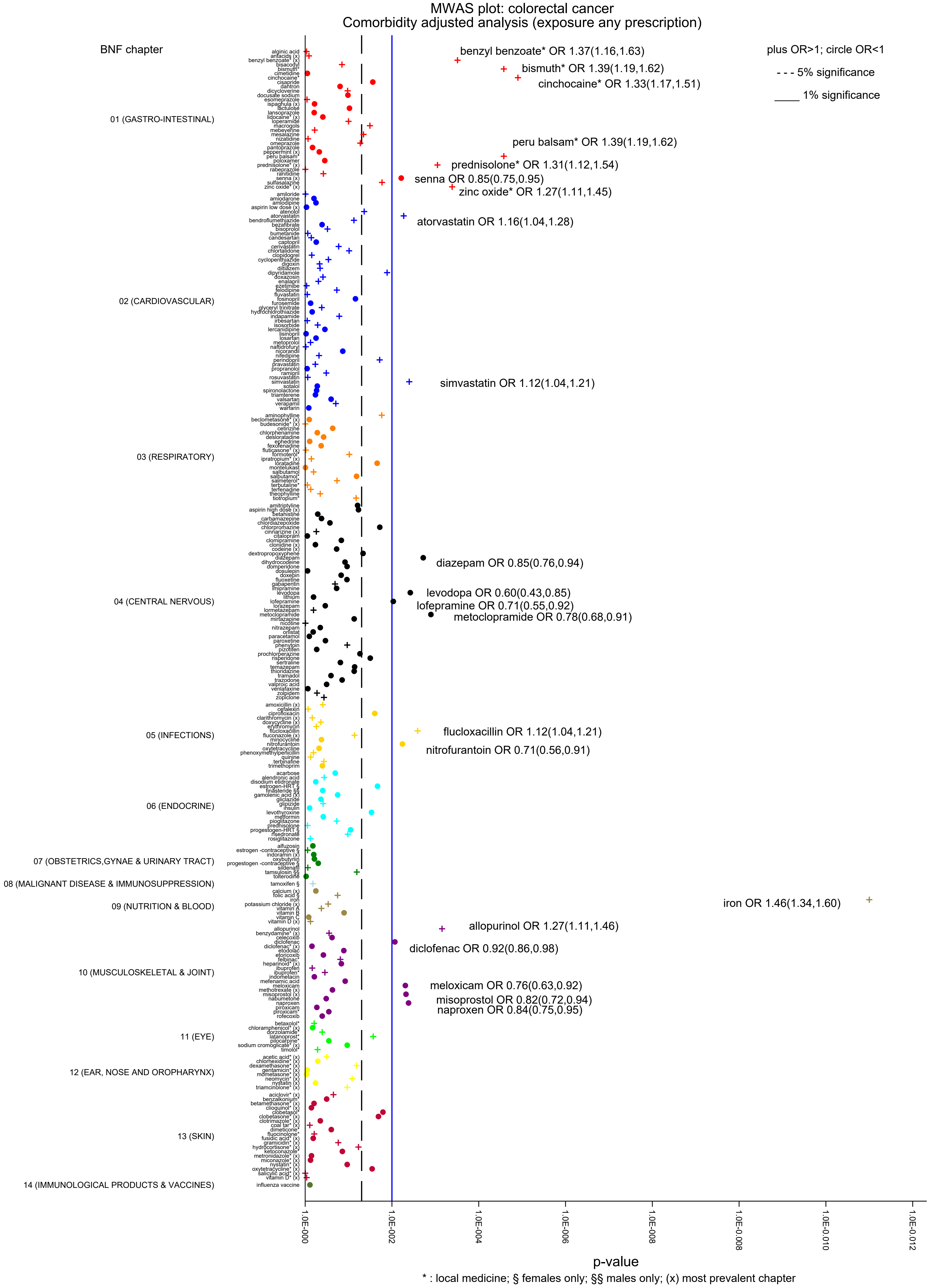
MWAS plot: colorectal cancer
Comorbidity adjusted analysis (exposure any prescription)
BNF chapter
plus OR>1; circle OR<1
- - - 5% significance
____ 1% significance
benzyl benzoate* OR 1.37(1.16,1.63)
bismuth* OR 1.39(1.19,1.62)
cinchocaine* OR 1.33(1.17,1.51)
peru balsam* OR 1.39(1.19,1.62)
prednisolone* OR 1.31(1.12,1.54)
senna OR 0.85(0.75,0.95)
zinc oxide* OR 1.27(1.11,1.45)
atorvastatin OR 1.16(1.04,1.28)
simvastatin OR 1.12(1.04,1.21)
diazepam OR 0.85(0.76,0.94)
levodopa OR 0.60(0.43,0.85)
lofepramine OR 0.71(0.55,0.92)
metoclopramide OR 0.78(0.68,0.91)
flucloxacillin OR 1.12(1.04,1.21)
nitrofurantoin OR 0.71(0.56,0.91)
iron OR 1.46(1.34,1.60)
allopurinol OR 1.27(1.11,1.46)
diclofenac OR 0.92(0.86,0.98)
meloxicam OR 0.76(0.63,0.92)
misoprostol OR 0.82(0.72,0.94)
naproxen OR 0.84(0.75,0.95)
01 (GASTRO-INTESTINAL)
02 (CARDIOVASCULAR)
03 (RESPIRATORY)
04 (CENTRAL NERVOUS)
05 (INFECTIONS)
06 (ENDOCRINE)
07 (OBSTETRICS,GYNAE & URINARY TRACT)
08 (MALIGNANT DISEASE & IMMUNOSUPPRESSION)
09 (NUTRITION & BLOOD)
10 (MUSCULOSKELETAL & JOINT)
11 (EYE)
12 (EAR, NOSE AND OROPHARYNX)
13 (SKIN)
14 (IMMUNOLOGICAL PRODUCTS & VACCINES)
1.0E-000
1.0E-002
1.0E-004
1.0E-006
1.0E-008
1.0E-008
1.0E-010
1.0E-012
p-value
* : local medicine; § females only; §§ males only; (x) most prevalent chapter

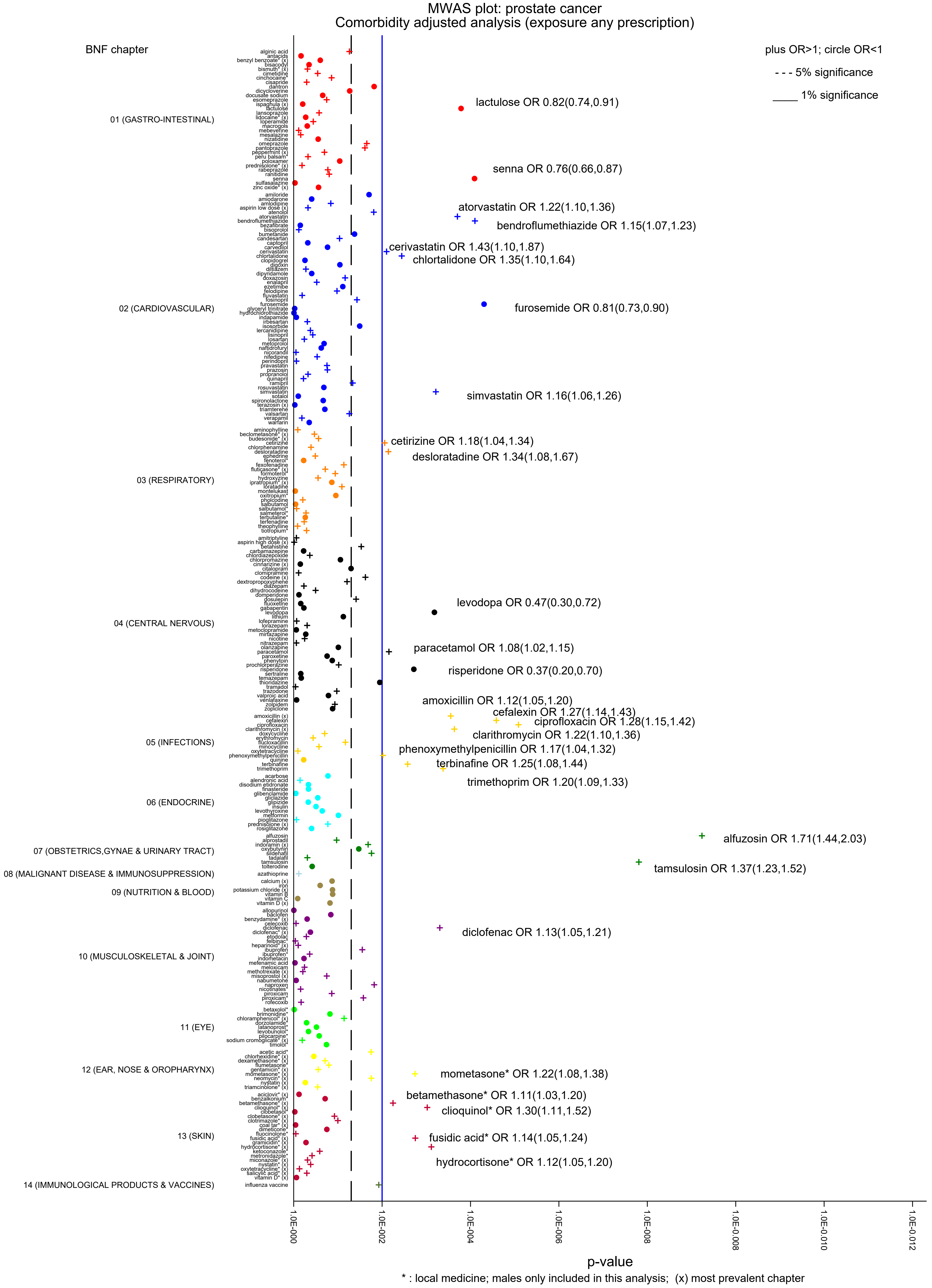
MWAS plot: prostate cancer
Comorbidity adjusted analysis (exposure any prescription)
BNF chapter
plus OR>1; circle OR<1
- - - 5% significance
____ 1% significance
01 (GASTRO-INTESTINAL)
lactulose OR 0.82(0.74,0.91)
senna OR 0.76(0.66,0.87)
02 (CARDIOVASCULAR)
atorvastatin OR 1.22(1.10,1.36)
bendroflumethiazide OR 1.15(1.07,1.23)
cerivastatin OR 1.43(1.10,1.87)
chlortalidone OR 1.35(1.10,1.64)
furosemide OR 0.81(0.73,0.90)
simvastatin OR 1.16(1.06,1.26)
03 (RESPIRATORY)
cetirizine OR 1.18(1.04,1.34)
desloratadine OR 1.34(1.08,1.67)
04 (CENTRAL NERVOUS)
levodopa OR 0.47(0.30,0.72)
paracetamol OR 1.08(1.02,1.15)
risperidone OR 0.37(0.20,0.70)
05 (INFECTIONS)
amoxicillin OR 1.12(1.05,1.20)
cefalexin OR 1.27(1.14,1.43)
ciprofloxacin OR 1.28(1.15,1.42)
clarithromycin OR 1.22(1.10,1.36)
phenoxymethylpenicillin OR 1.17(1.04,1.32)
terbinafine OR 1.25(1.08,1.44)
trimethoprim OR 1.20(1.09,1.33)
06 (ENDOCRINE)
07 (OBSTETRICS,GYNAE & URINARY TRACT)
alfuzosin OR 1.71(1.44,2.03)
tamsulosin OR 1.37(1.23,1.52)
08 (MALIGNANT DISEASE & IMMUNOSUPPRESSION)
09 (NUTRITION & BLOOD)
10 (MUSCULOSKELETAL & JOINT)
diclofenac OR 1.13(1.05,1.21)
11 (EYE)
12 (EAR, NOSE & OROPHARYNX)
mometasone* OR 1.22(1.08,1.38)
13 (SKIN)
betamethasone* OR 1.11(1.03,1.20)
clioquinol* OR 1.30(1.11,1.52)
fusidic acid* OR 1.14(1.05,1.24)
hydrocortisone* OR 1.12(1.05,1.20)
14 (IMMUNOLOGICAL PRODUCTS & VACCINES)
1.0E-000
1.0E-002
1.0E-004
1.0E-006
1.0E-008
1.0E-008
1.0E-010
1.0E-012
p-value
* : local medicine; males only included in this analysis; (x) most prevalent chapter

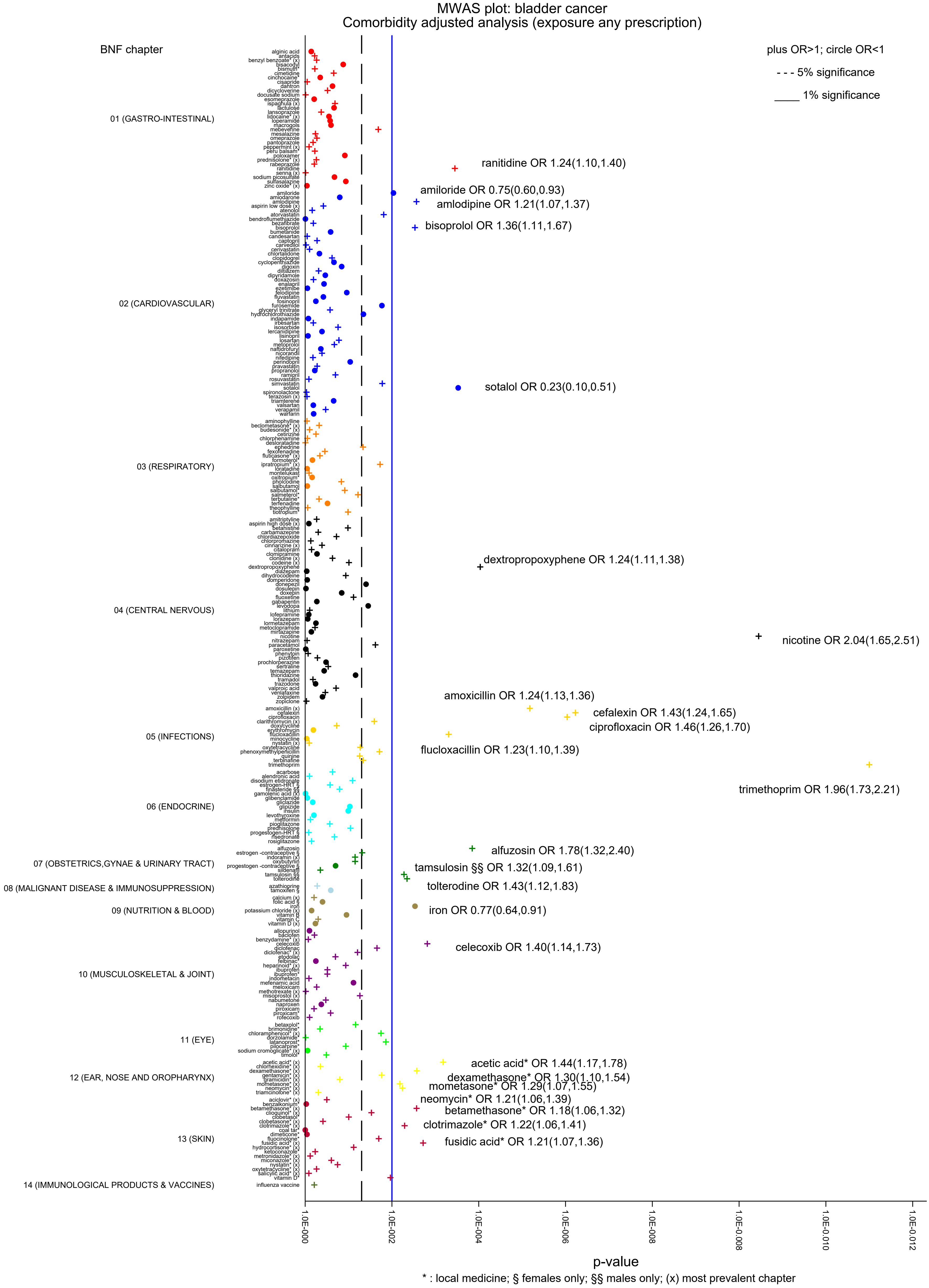
MWAS plot: bladder cancer
Comorbidity adjusted analysis (exposure any prescription)
BNF chapter
plus OR>1; circle OR<1
- - - 5% significance
____ 1% significance
01 (GASTRO-INTESTINAL)
02 (CARDIOVASCULAR)
03 (RESPIRATORY)
04 (CENTRAL NERVOUS)
05 (INFECTIONS)
06 (ENDOCRINE)
07 (OBSTETRICS,GYNAE & URINARY TRACT)
08 (MALIGNANT DISEASE & IMMUNOSUPPRESSION)
09 (NUTRITION & BLOOD)
10 (MUSCULOSKELETAL & JOINT)
11 (EYE)
12 (EAR, NOSE AND OROPHARYNX)
13 (SKIN)
14 (IMMUNOLOGICAL PRODUCTS & VACCINES)
ranitidine OR 1.24(1.10,1.40)
amiloride OR 0.75(0.60,0.93)
amlodipine OR 1.21(1.07,1.37)
bisoprolol OR 1.36(1.11,1.67)
sotalol OR 0.23(0.10,0.51)
dextropropoxyphene OR 1.24(1.11,1.38)
nicotine OR 2.04(1.65,2.51)
amoxicillin OR 1.24(1.13,1.36)
cefalexin OR 1.43(1.24,1.65)
ciprofloxacin OR 1.46(1.26,1.70)
flucloxacillin OR 1.23(1.10,1.39)
trimethoprim OR 1.96(1.73,2.21)
alfuzosin OR 1.78(1.32,2.40)
tamsulosin §§ OR 1.32(1.09,1.61)
tolterodine OR 1.43(1.12,1.83)
iron OR 0.77(0.64,0.91)
celecoxib OR 1.40(1.14,1.73)
acetic acid* OR 1.44(1.17,1.78)
dexamethasone* OR 1.30(1.10,1.54)
mometasone* OR 1.29(1.07,1.55)
neomycin* OR 1.21(1.06,1.39)
betamethasone* OR 1.18(1.06,1.32)
clotrimazole* OR 1.22(1.06,1.41)
fusidic acid* OR 1.21(1.07,1.36)
1.0E-000
1.0E-002
1.0E-004
1.0E-006
1.0E-008
1.0E-010
1.0E-012
p-value
* : local medicine; § females only; §§ males only; (x) most prevalent chapter

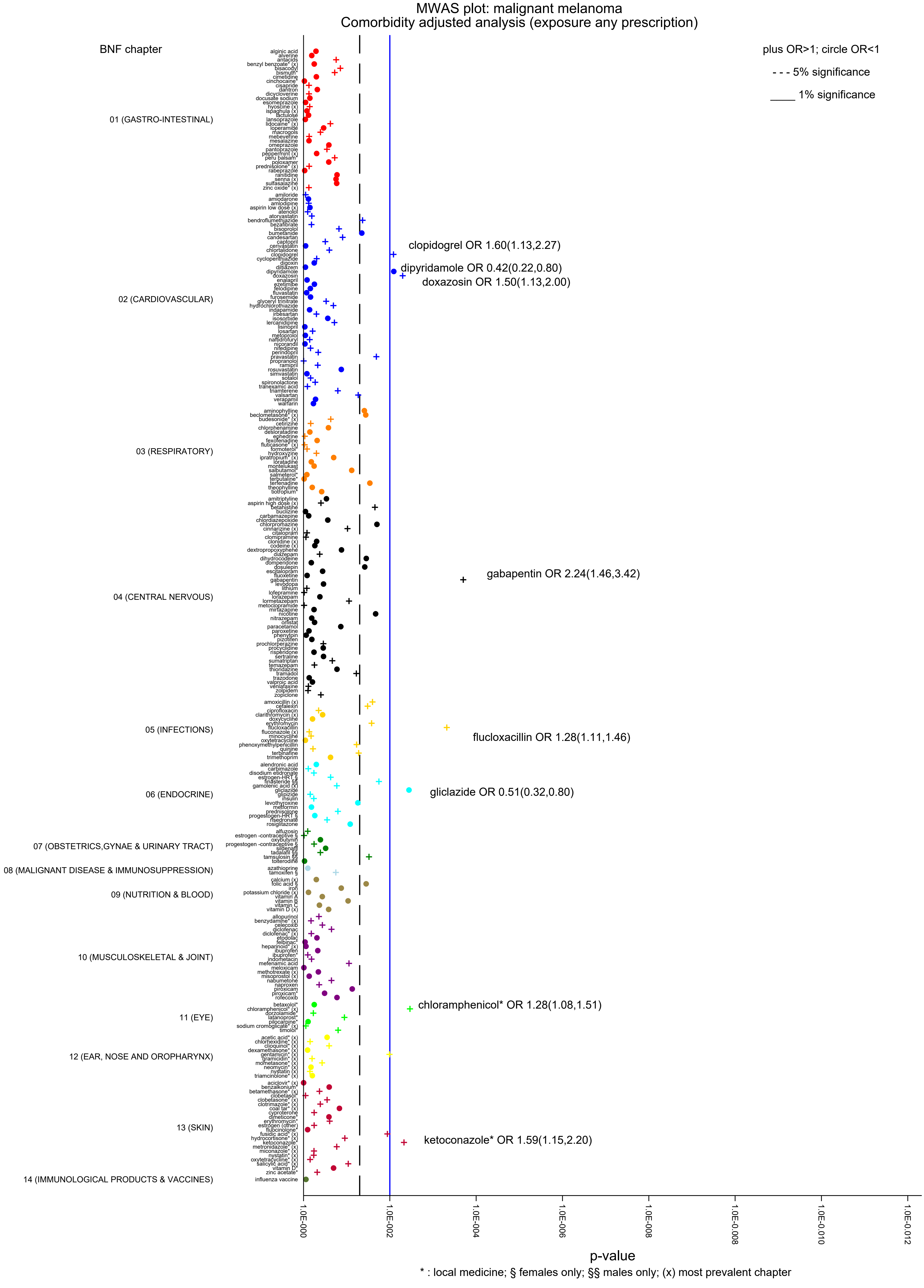
MWAS plot: malignant melanoma
Comorbidity adjusted analysis (exposure any prescription)
BNF chapter
plus OR>1; circle OR<1
- - - 5% significance
____ 1% significance
01 (GASTRO-INTESTINAL)
02 (CARDIOVASCULAR)
03 (RESPIRATORY)
04 (CENTRAL NERVOUS)
05 (INFECTIONS)
06 (ENDOCRINE)
07 (OBSTETRICS,GYNAE & URINARY TRACT)
08 (MALIGNANT DISEASE & IMMUNOSUPPRESSION)
09 (NUTRITION & BLOOD)
10 (MUSCULOSKELETAL & JOINT)
11 (EYE)
12 (EAR, NOSE AND OROPHARYNX)
13 (SKIN)
14 (IMMUNOLOGICAL PRODUCTS & VACCINES)
clopidogrel OR 1.60(1.13,2.27)
dipyridamole OR 0.42(0.22,0.80)
doxazosin OR 1.50(1.13,2.00)
gabapentin OR 2.24(1.46,3.42)
flucloxacillin OR 1.28(1.11,1.46)
gliclazide OR 0.51(0.32,0.80)
chloramphenicol* OR 1.28(1.08,1.51)
ketoconazole* OR 1.59(1.15,2.20)
1.0E-000
1.0E-002
1.0E-004
1.0E-006
1.0E-008
1.0E-0.008
1.0E-0.010
1.0E-0.012
p-value
* : local medicine; § females only; §§ males only; (x) most prevalent chapter

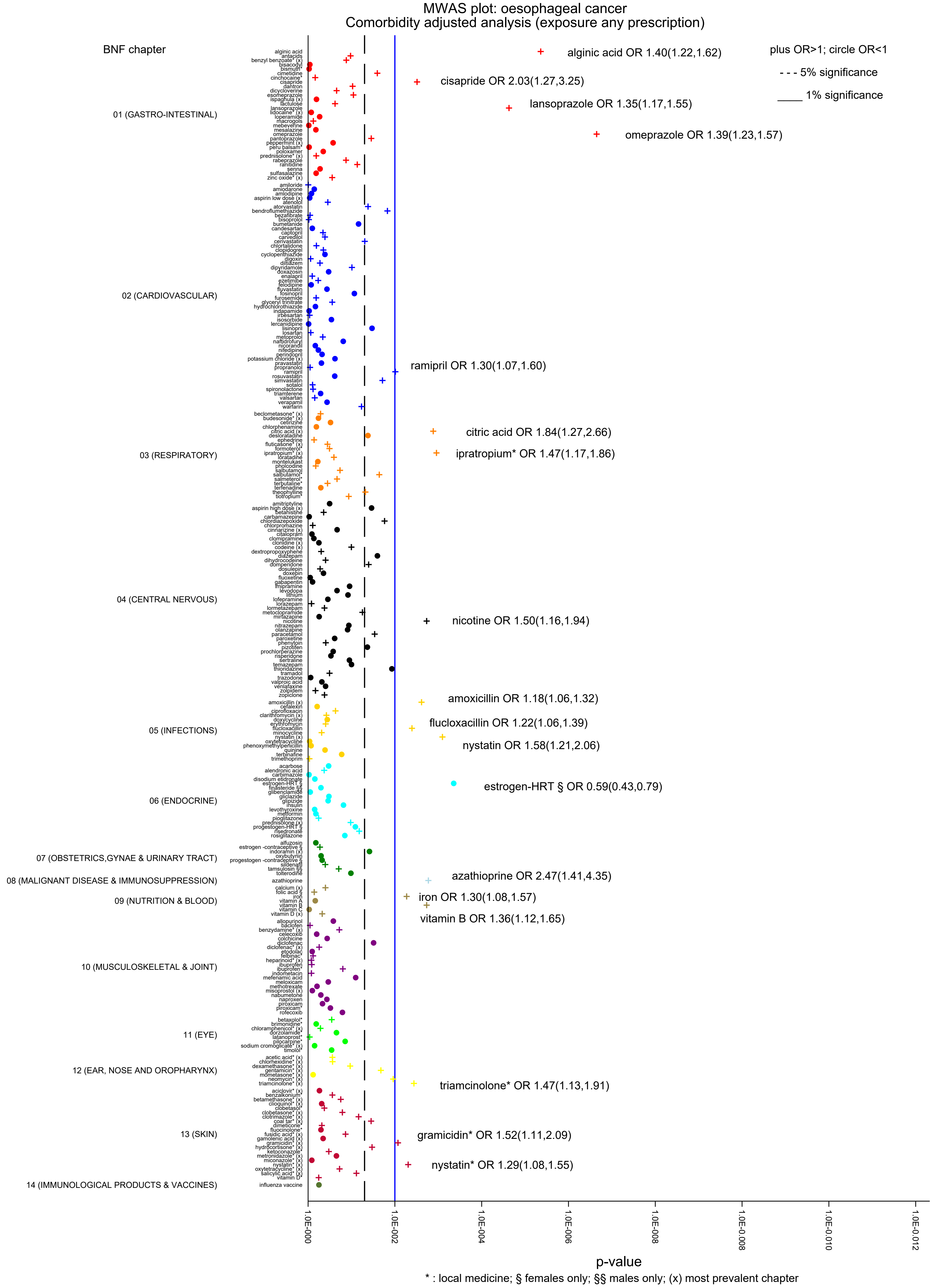
MWAS plot: oesophageal cancer
Comorbidity adjusted analysis (exposure any prescription)
BNF chapter
plus OR>1; circle OR<1
- - - 5% significance
____ 1% significance
alginic acid OR 1.40(1.22,1.62)
cisapride OR 2.03(1.27,3.25)
lansoprazole OR 1.35(1.17,1.55)
omeprazole OR 1.39(1.23,1.57)
01 (GASTRO-INTESTINAL)
02 (CARDIOVASCULAR)
ramipril OR 1.30(1.07,1.60)
03 (RESPIRATORY)
citric acid OR 1.84(1.27,2.66)
ipratropium* OR 1.47(1.17,1.86)
04 (CENTRAL NERVOUS)
nicotine OR 1.50(1.16,1.94)
05 (INFECTIONS)
amoxicillin OR 1.18(1.06,1.32)
flucloxacillin OR 1.22(1.06,1.39)
nystatin OR 1.58(1.21,2.06)
06 (ENDOCRINE)
estrogen-HRT § OR 0.59(0.43,0.79)
07 (OBSTETRICS,GYNAE & URINARY TRACT)
08 (MALIGNANT DISEASE & IMMUNOSUPPRESSION)
azathioprine OR 2.47(1.41,4.35)
09 (NUTRITION & BLOOD)
iron OR 1.30(1.08,1.57)
vitamin B OR 1.36(1.12,1.65)
10 (MUSCULOSKELETAL & JOINT)
11 (EYE)
12 (EAR, NOSE AND OROPHARYNX)
triamcinolone* OR 1.47(1.13,1.91)
13 (SKIN)
gramicidin* OR 1.52(1.11,2.09)
nystatin* OR 1.29(1.08,1.55)
14 (IMMUNOLOGICAL PRODUCTS & VACCINES)
1.0E-000
1.0E-002
1.0E-004
1.0E-006
1.0E-008
1.0E-0.008
1.0E-0.010
1.0E-0.012
p-value
* : local medicine; § females only; §§ males only; (x) most prevalent chapter

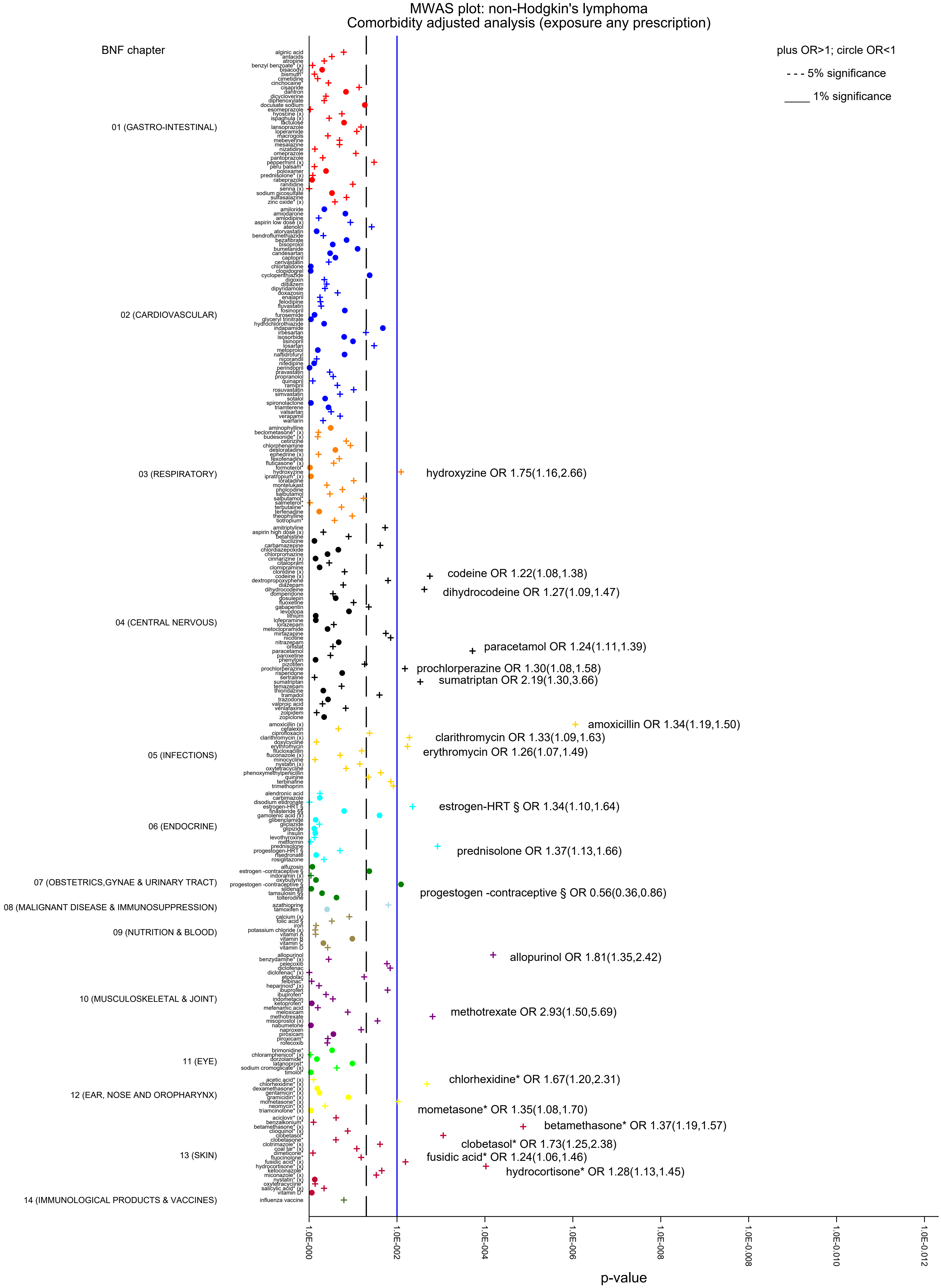

* : local medicine; § females only; §§ males only; (x) most prevalent chapter

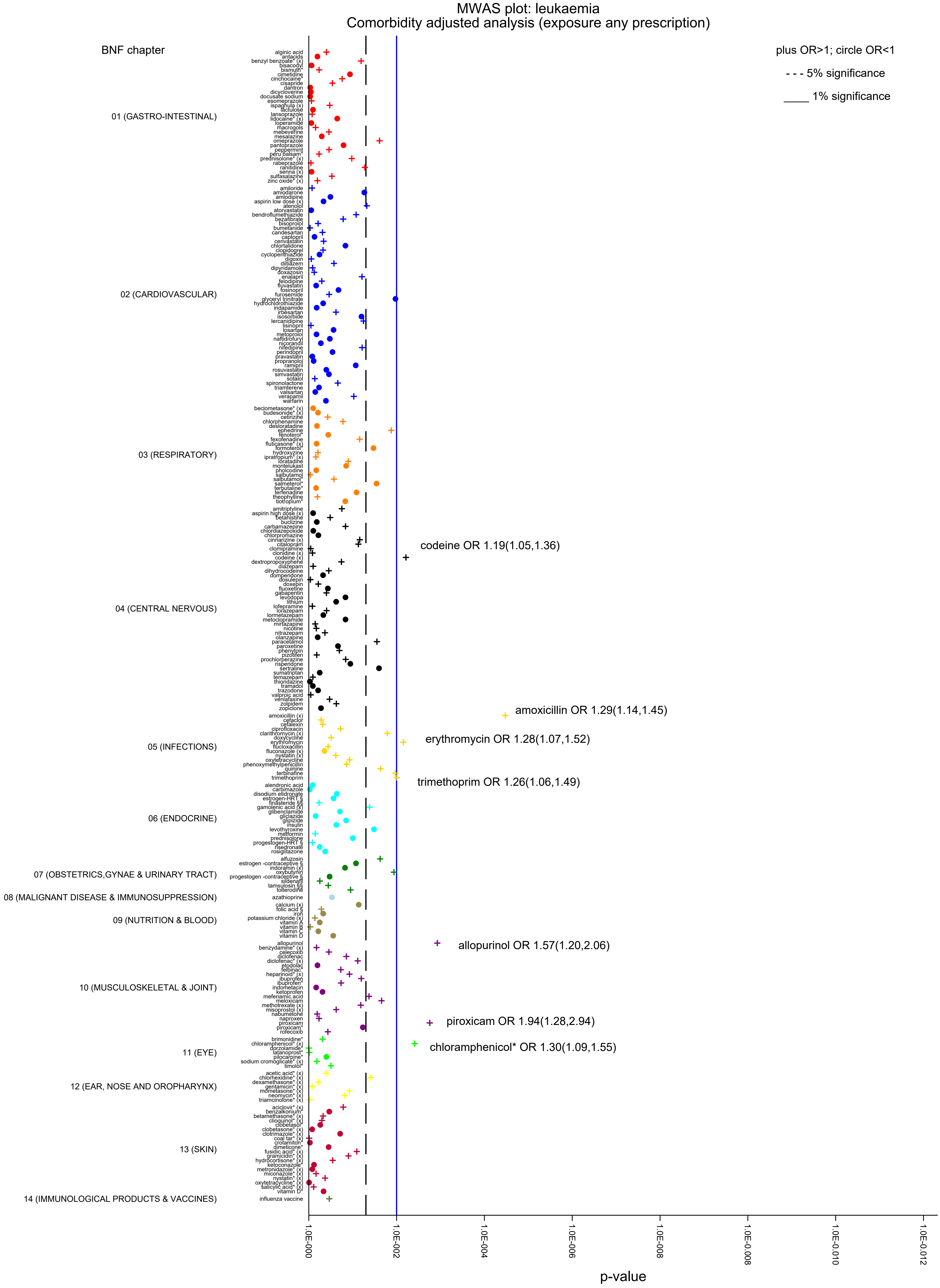

* : local medicine; § females only; §§ males only; (x) most prevalent chapter

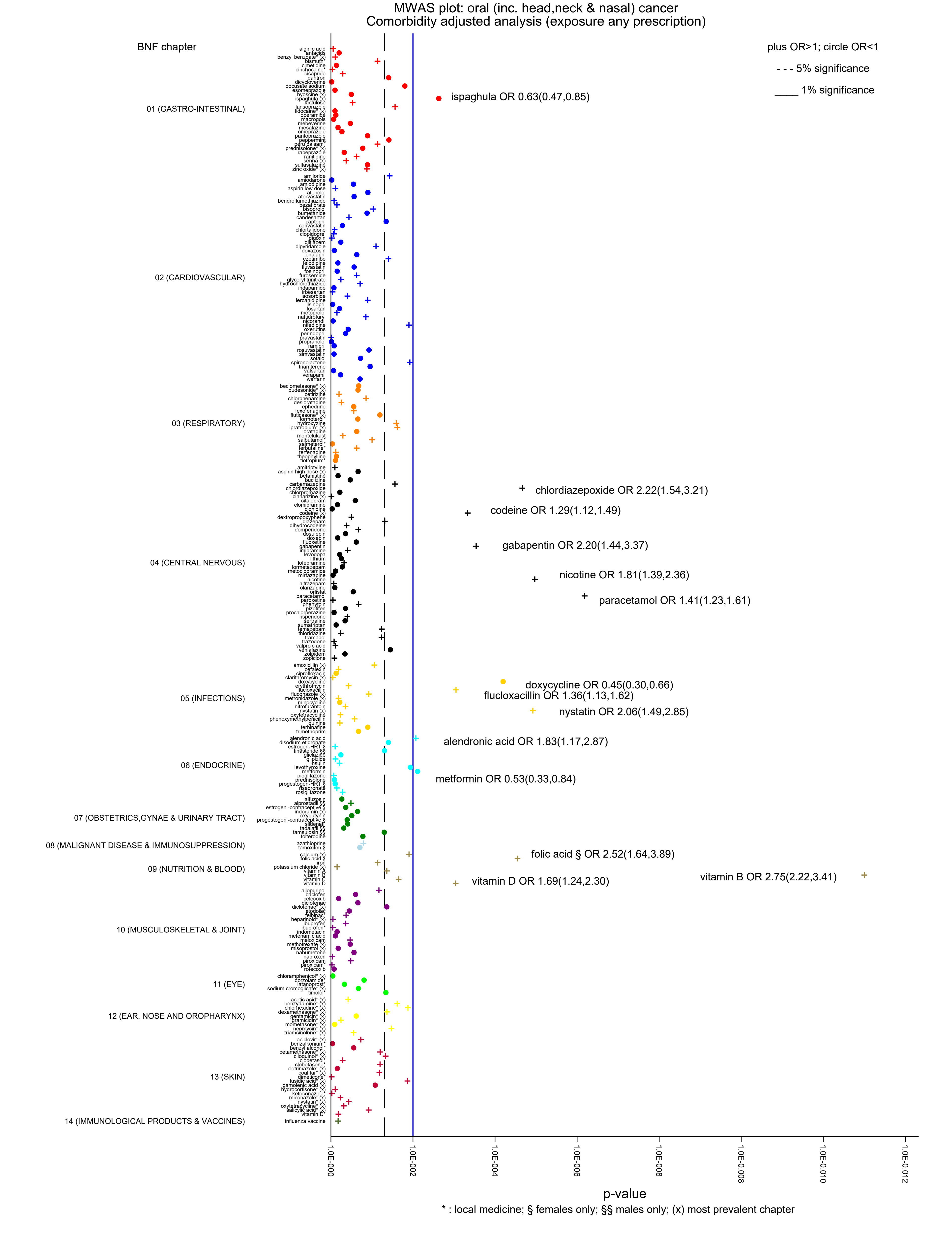
MWAS plot: oral (inc. head,neck & nasal) cancer
Comorbidity adjusted analysis (exposure any prescription)
BNF chapter
plus OR>1; circle OR<1
- - - 5% significance
____ 1% significance
ispaghula OR 0.63(0.47,0.85)
01 (GASTRO-INTESTINAL)
02 (CARDIOVASCULAR)
03 (RESPIRATORY)
chlordiazepoxide OR 2.22(1.54,3.21)
codeine OR 1.29(1.12,1.49)
gabapentin OR 2.20(1.44,3.37)
nicotine OR 1.81(1.39,2.36)
paracetamol OR 1.41(1.23,1.61)
04 (CENTRAL NERVOUS)
doxycycline OR 0.45(0.30,0.66)
flucloxacillin OR 1.36(1.13,1.62)
nystatin OR 2.06(1.49,2.85)
05 (INFECTIONS)
alendronic acid OR 1.83(1.17,2.87)
06 (ENDOCRINE)
metformin OR 0.53(0.33,0.84)
07 (OBSTETRICS,GYNAE & URINARY TRACT)
08 (MALIGNANT DISEASE & IMMUNOSUPPRESSION)
folic acid § OR 2.52(1.64,3.89)
09 (NUTRITION & BLOOD)
vitamin B OR 2.75(2.22,3.41)
vitamin D OR 1.69(1.24,2.30)
10 (MUSCULOSKELETAL & JOINT)
11 (EYE)
12 (EAR, NOSE AND OROPHARYNX)
13 (SKIN)
14 (IMMUNOLOGICAL PRODUCTS & VACCINES)
1.0E-000
1.0E-002
1.0E-004
1.0E-006
1.0E-008
1.0E-010
1.0E-012
p-value
* : local medicine; § females only; §§ males only; (x) most prevalent chapter

MWAS plot: ovarian cancer
Comorbidity adjusted analysis (exposure any prescription)

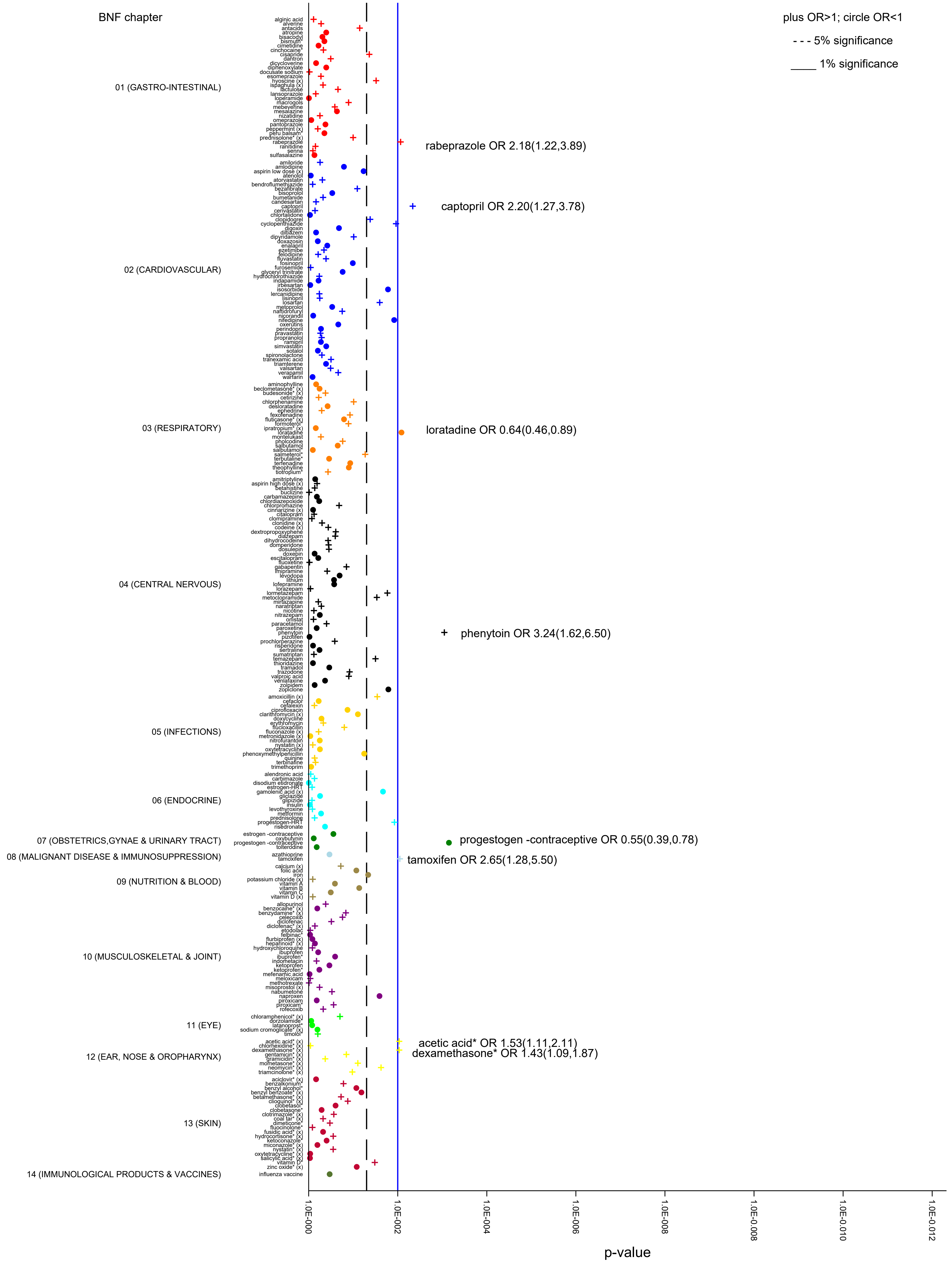

* : local medicine; females only included in this analysis; (l): local; (x) most prevalent chapter

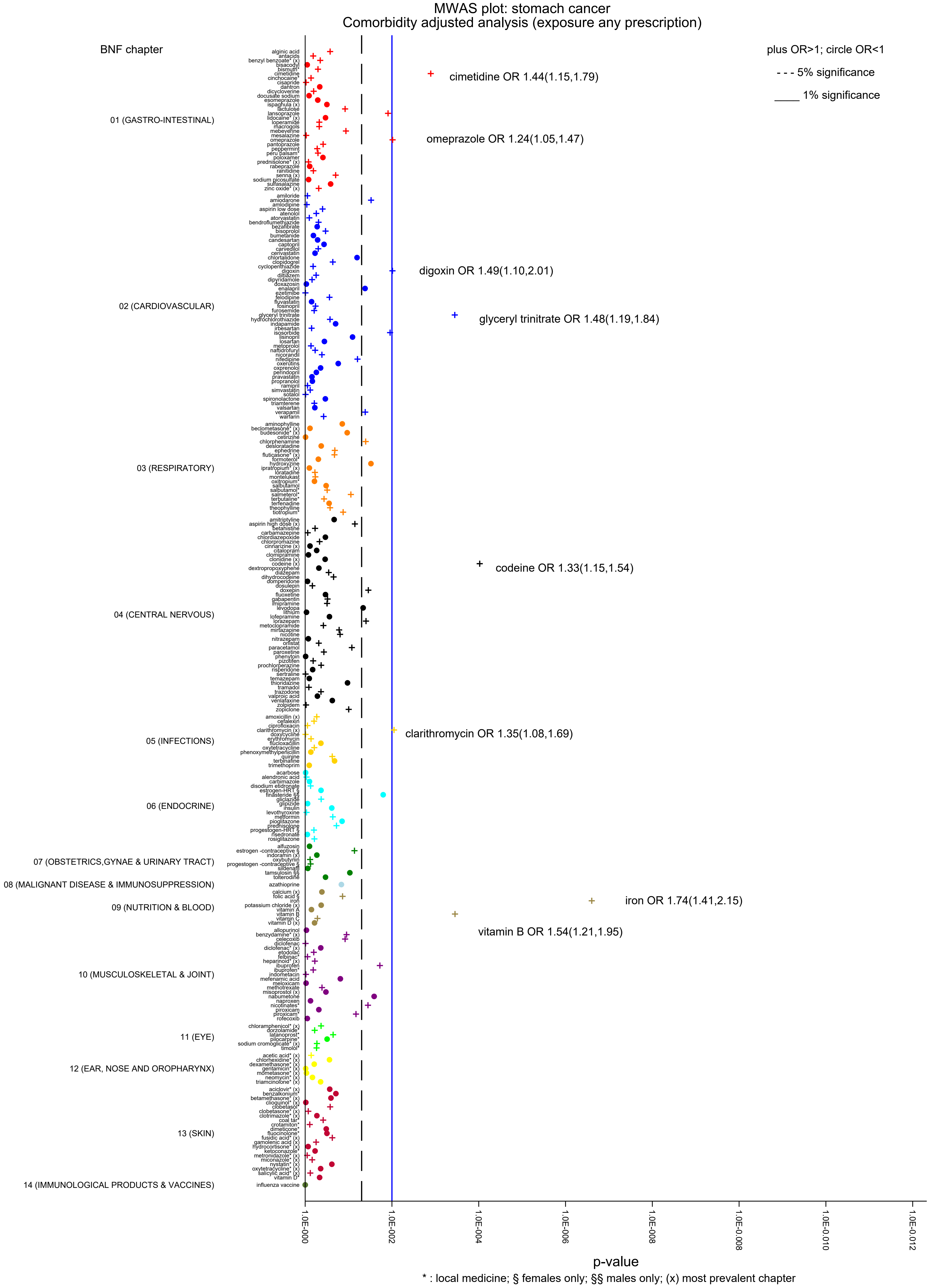

MWAS plot: stomach cancer
Comorbidity adjusted analysis (exposure any prescription)
BNF chapter
plus OR>1; circle OR<1
- - - 5% significance
____ 1% significance
cimetidine OR 1.44(1.15,1.79)
omeprazole OR 1.24(1.05,1.47)
digoxin OR 1.49(1.10,2.01)
glyceryl trinitrate OR 1.48(1.19,1.84)
codeine OR 1.33(1.15,1.54)
clarithromycin OR 1.35(1.08,1.69)
iron OR 1.74(1.41,2.15)
vitamin B OR 1.54(1.21,1.95)
01 (GASTRO-INTESTINAL)
02 (CARDIOVASCULAR)
03 (RESPIRATORY)
04 (CENTRAL NERVOUS)
05 (INFECTIONS)
06 (ENDOCRINE)
07 (OBSTETRICS,GYNAE & URINARY TRACT)
08 (MALIGNANT DISEASE & IMMUNOSUPPRESSION)
09 (NUTRITION & BLOOD)
10 (MUSCULOSKELETAL & JOINT)
11 (EYE)
12 (EAR, NOSE AND OROPHARYNX)
13 (SKIN)
14 (IMMUNOLOGICAL PRODUCTS & VACCINES)
1.0E-000
1.0E-002
1.0E-004
1.0E-006
1.0E-008
1.0E-008
1.0E-010
1.0E-012
p-value
* : local medicine; § females only; §§ males only; (x) most prevalent chapter

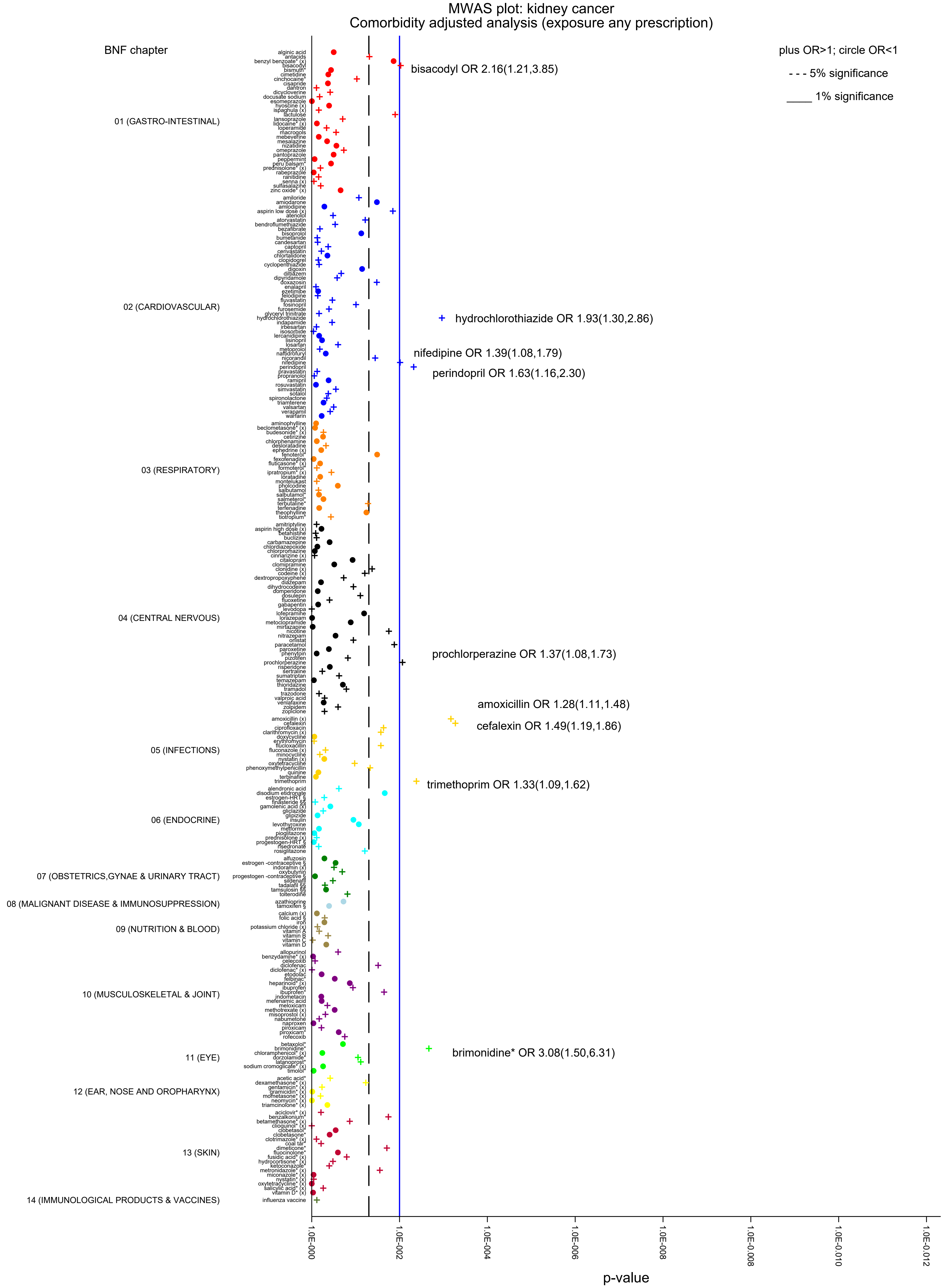

* : local medicine; § females only; §§ males only; (x) most prevalent chapter

MWAS plot: cervical & other gynae cancer
Comorbidity adjusted analysis (exposure any prescription)

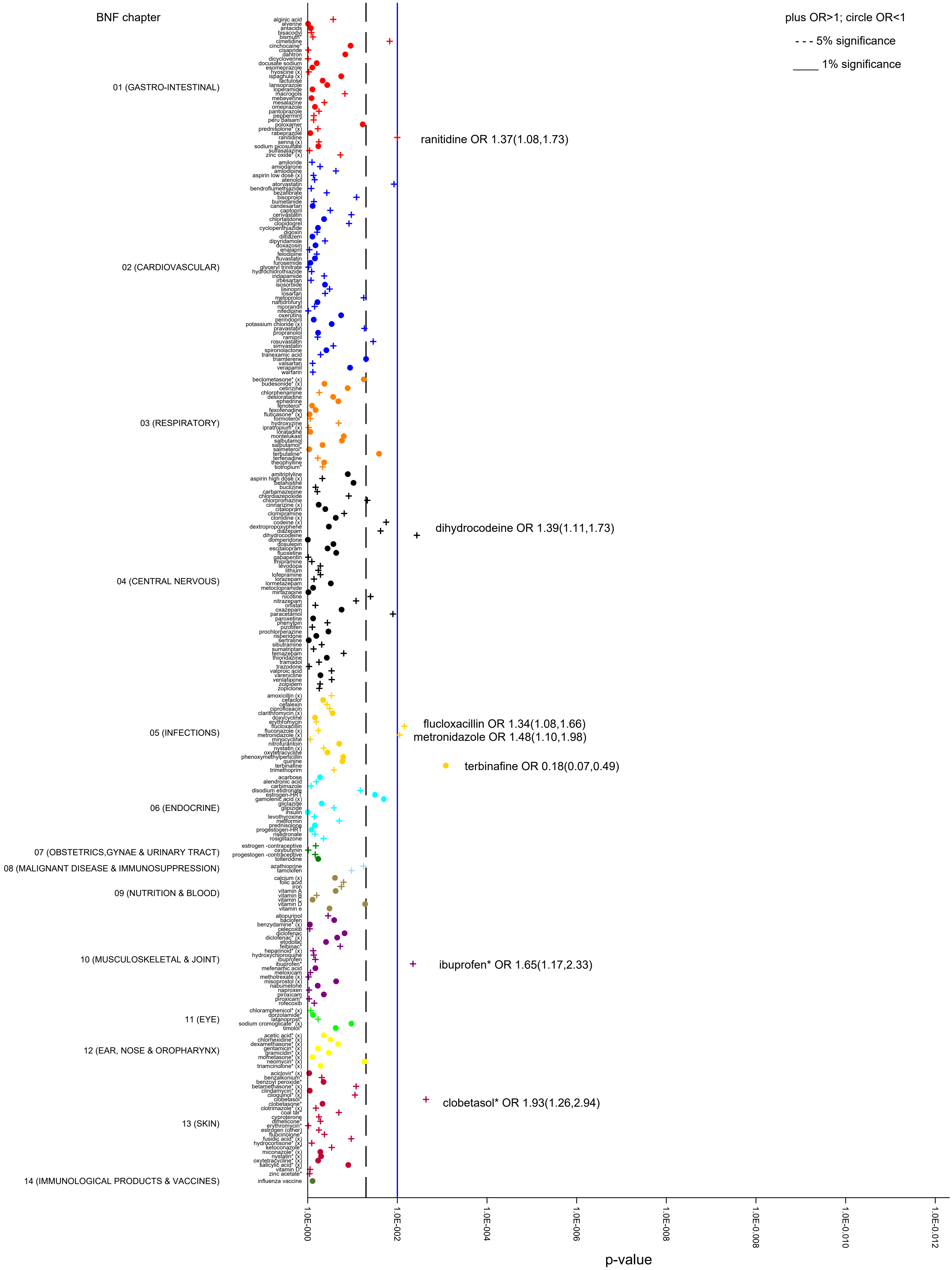

* : local medicine; females only included in this analysis; (l): local; (x) most prevalent chapter

MWAS plot: pancreatic cancer
Comorbidity adjusted analysis (exposure any prescription)

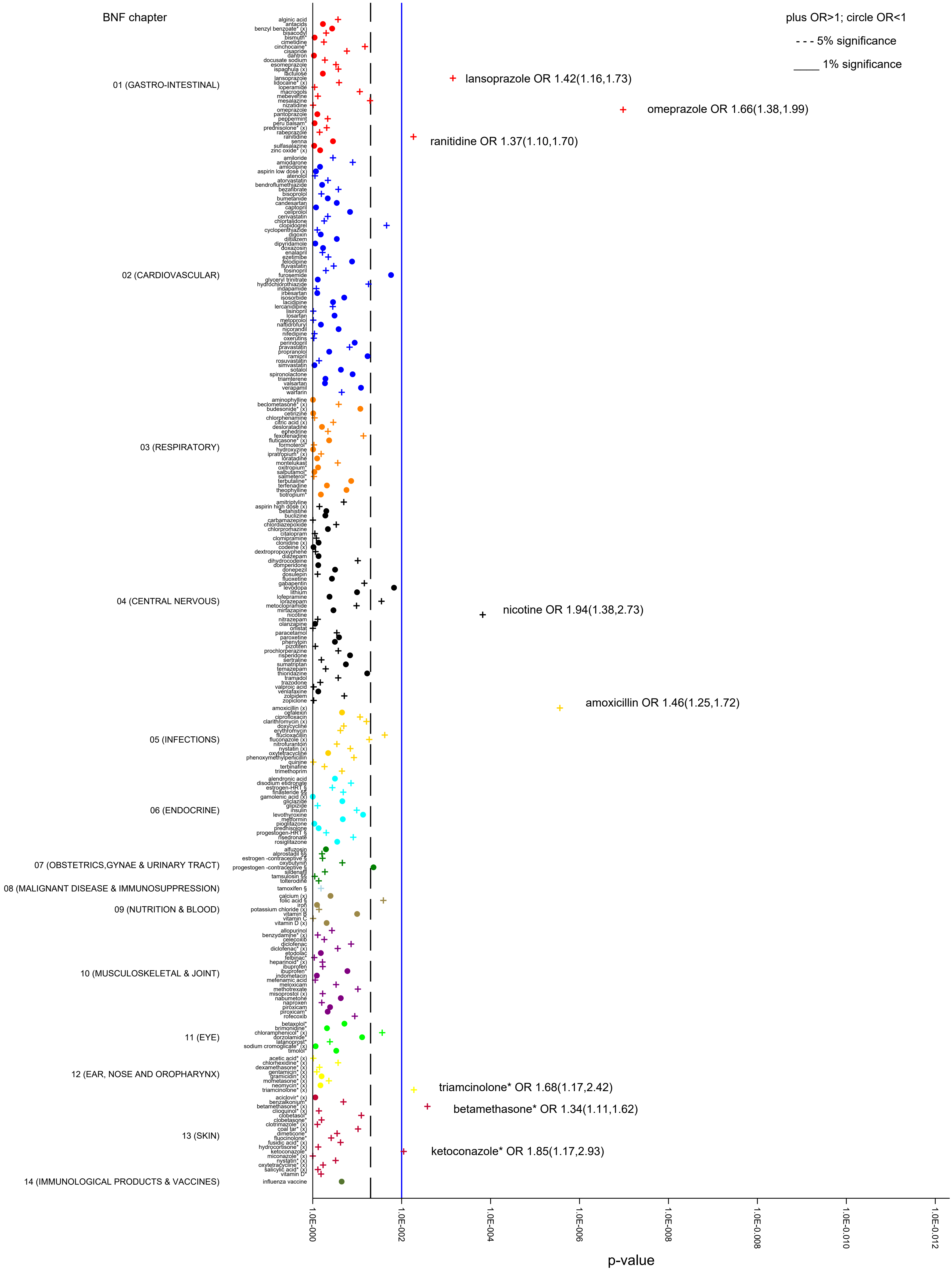

* : local medicine; § females only; §§ males only; (x) most prevalent chapter

MWAS plot: cancer of the uterus
Comorbidity adjusted analysis (exposure any prescription)

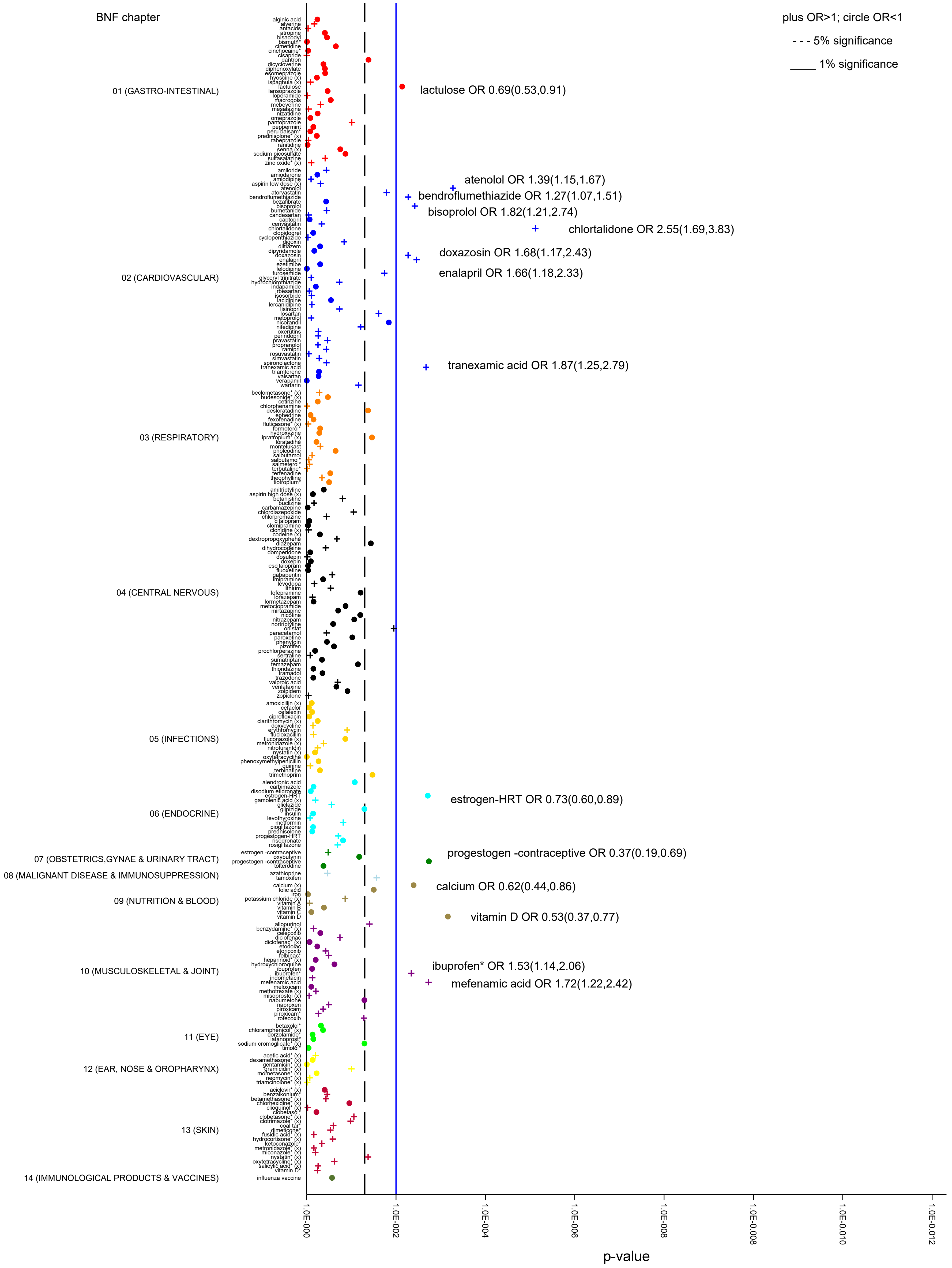

* : local medicine; females only included in this analysis; (l): local; (x) most prevalent chapter

MWAS plot: cancer of the brain and central nervous system
Comorbidity adjusted analysis (exposure any prescription)

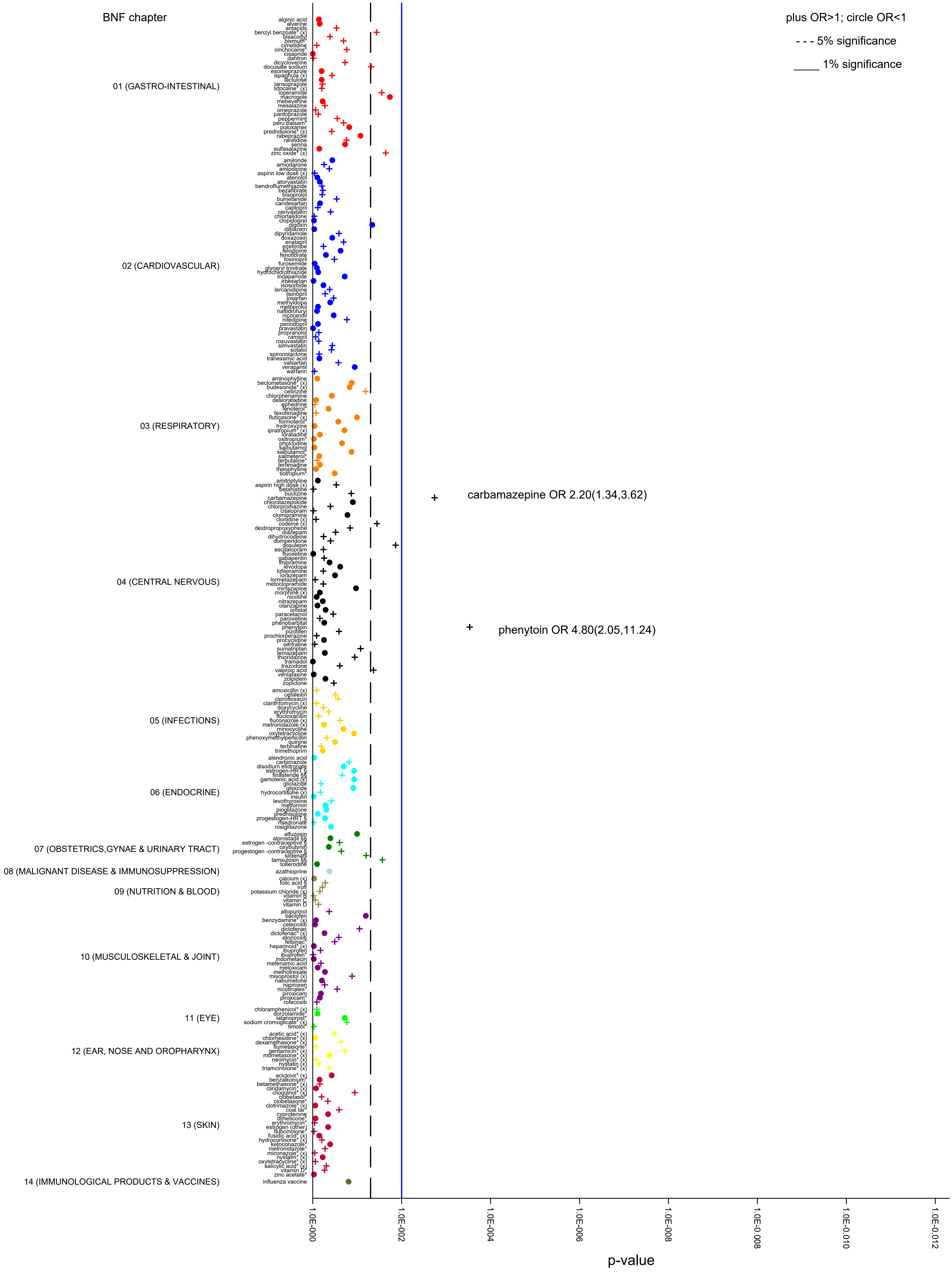

* : local medicine; § females only; §§ males only; (x) most prevalent chapter

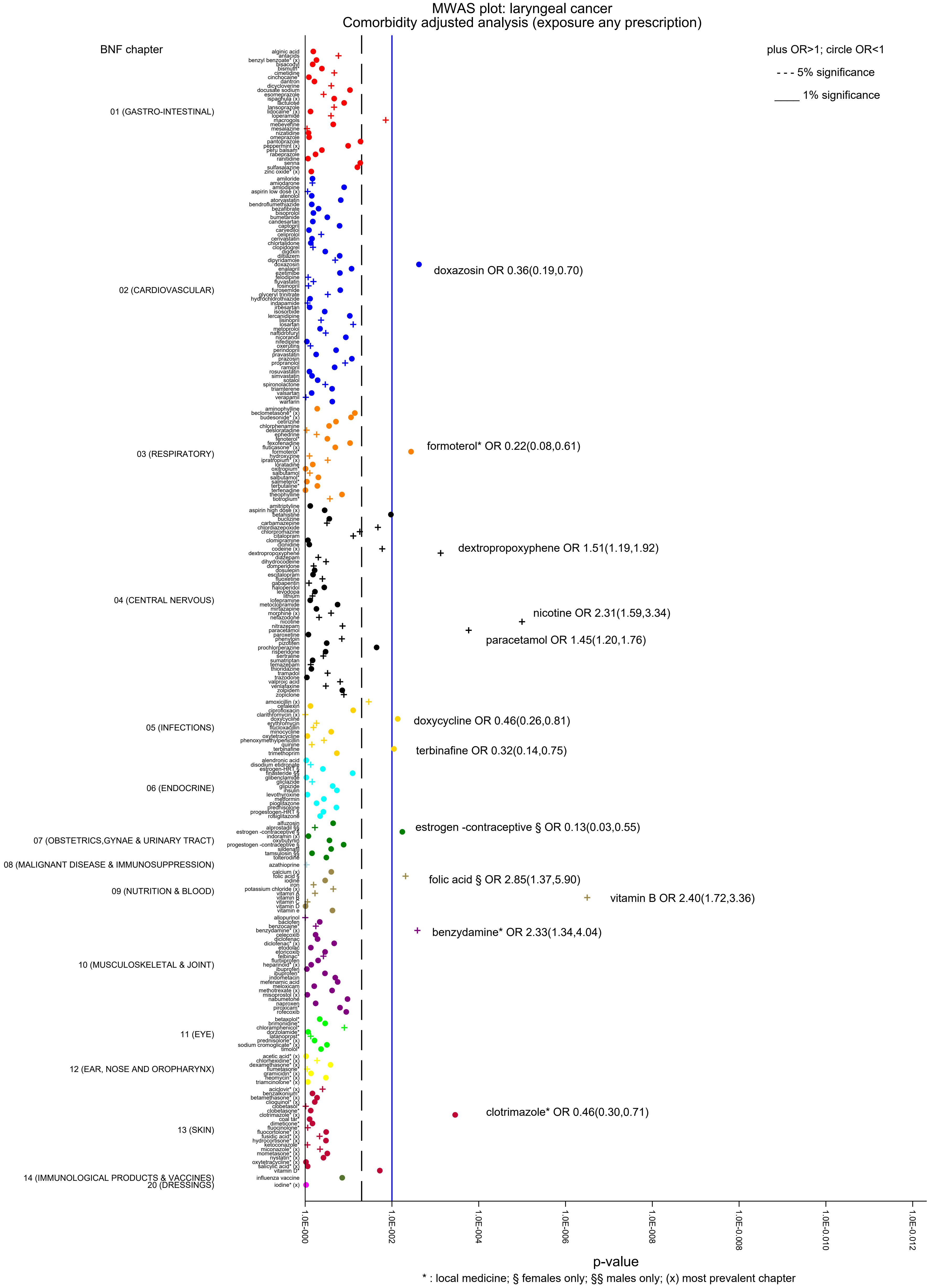
MWAS plot: laryngeal cancer
Comorbidity adjusted analysis (exposure any prescription)
BNF chapter
plus OR>1; circle OR<1
- - - 5% significance
____ 1% significance
01 (GASTRO-INTESTINAL)
02 (CARDIOVASCULAR)
03 (RESPIRATORY)
04 (CENTRAL NERVOUS)
05 (INFECTIONS)
06 (ENDOCRINE)
07 (OBSTETRICS,GYNAE & URINARY TRACT)
08 (MALIGNANT DISEASE & IMMUNOSUPPRESSION)
09 (NUTRITION & BLOOD)
10 (MUSCULOSKELETAL & JOINT)
11 (EYE)
12 (EAR, NOSE AND OROPHARYNX)
13 (SKIN)
14 (IMMUNOLOGICAL PRODUCTS & VACCINES)
20 (DRESSINGS)
doxazosin OR 0.36(0.19,0.70)
formoterol* OR 0.22(0.08,0.61)
dextropropoxyphene OR 1.51(1.19,1.92)
nicotine OR 2.31(1.59,3.34)
paracetamol OR 1.45(1.20,1.76)
doxycycline OR 0.46(0.26,0.81)
terbinafine OR 0.32(0.14,0.75)
estrogen -contraceptive § OR 0.13(0.03,0.55)
folic acid § OR 2.85(1.37,5.90)
vitamin B OR 2.40(1.72,3.36)
benzydamine* OR 2.33(1.34,4.04)
clotrimazole* OR 0.46(0.30,0.71)
1.0E-000
1.0E-002
1.0E-004
1.0E-006
1.0E-008
1.0E-008
1.0E-010
1.0E-012
p-value
* : local medicine; § females only; §§ males only; (x) most prevalent chapter

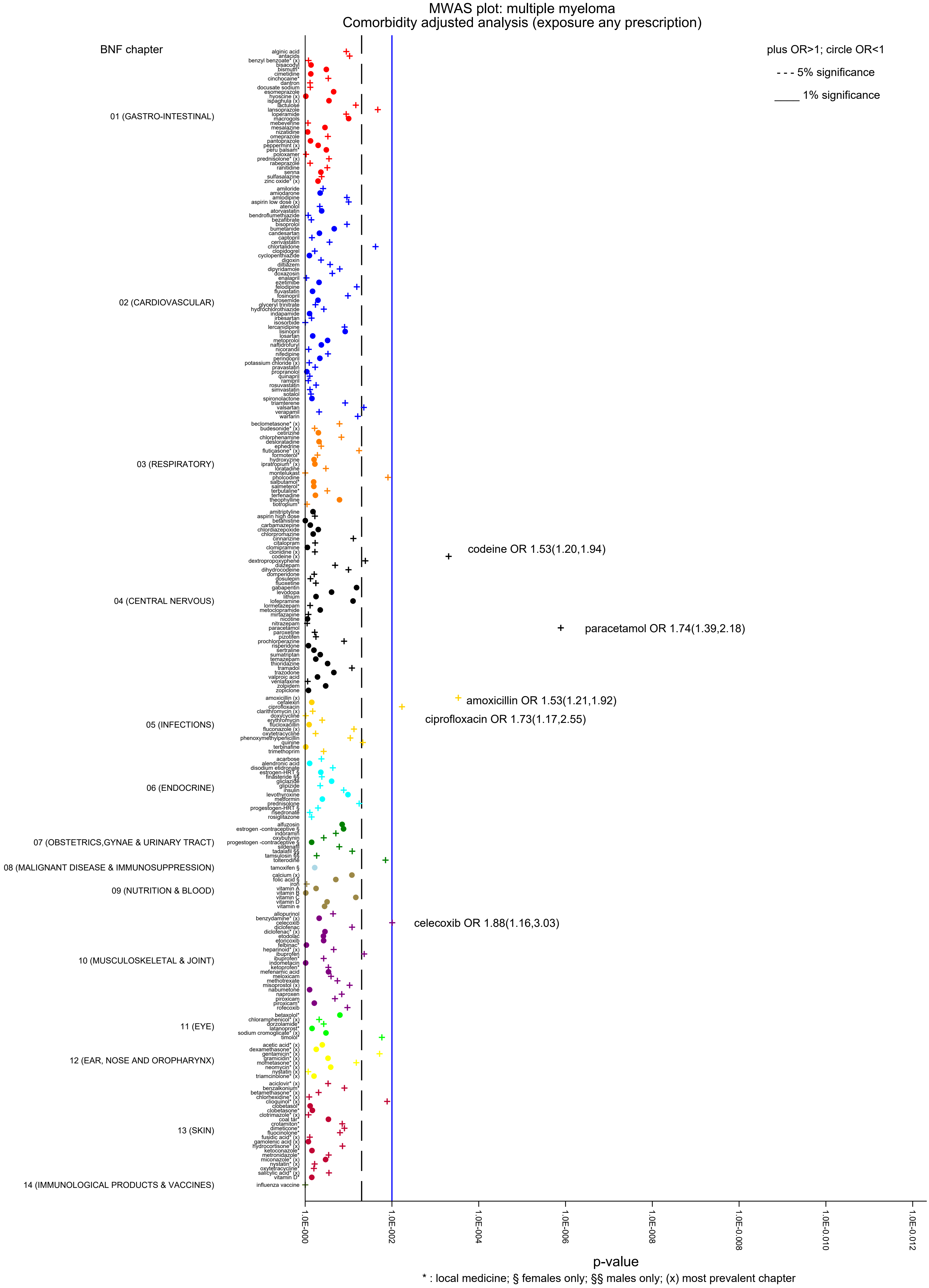

MWAS plot: multiple myeloma
Comorbidity adjusted analysis (exposure any prescription)
BNF chapter
plus OR>1; circle OR<1
- - - 5% significance
____ 1% significance
01 (GASTRO-INTESTINAL)
02 (CARDIOVASCULAR)
03 (RESPIRATORY)
04 (CENTRAL NERVOUS)
05 (INFECTIONS)
06 (ENDOCRINE)
07 (OBSTETRICS,GYNAE & URINARY TRACT)
08 (MALIGNANT DISEASE & IMMUNOSUPPRESSION)
09 (NUTRITION & BLOOD)
10 (MUSCULOSKELETAL & JOINT)
11 (EYE)
12 (EAR, NOSE AND OROPHARYNX)
13 (SKIN)
14 (IMMUNOLOGICAL PRODUCTS & VACCINES)
codeine OR 1.53(1.20,1.94)
paracetamol OR 1.74(1.39,2.18)
amoxicillin OR 1.53(1.21,1.92)
ciprofloxacin OR 1.73(1.17,2.55)
celecoxib OR 1.88(1.16,3.03)
1.0E-000
1.0E-002
1.0E-004
1.0E-006
1.0E-008
1.0E-0.008
1.0E-0.010
1.0E-0.012
p-value
* : local medicine; § females only; §§ males only; (x) most prevalent chapter

MWAS plot: liver cancer
Comorbidity adjusted analysis (exposure any prescription)

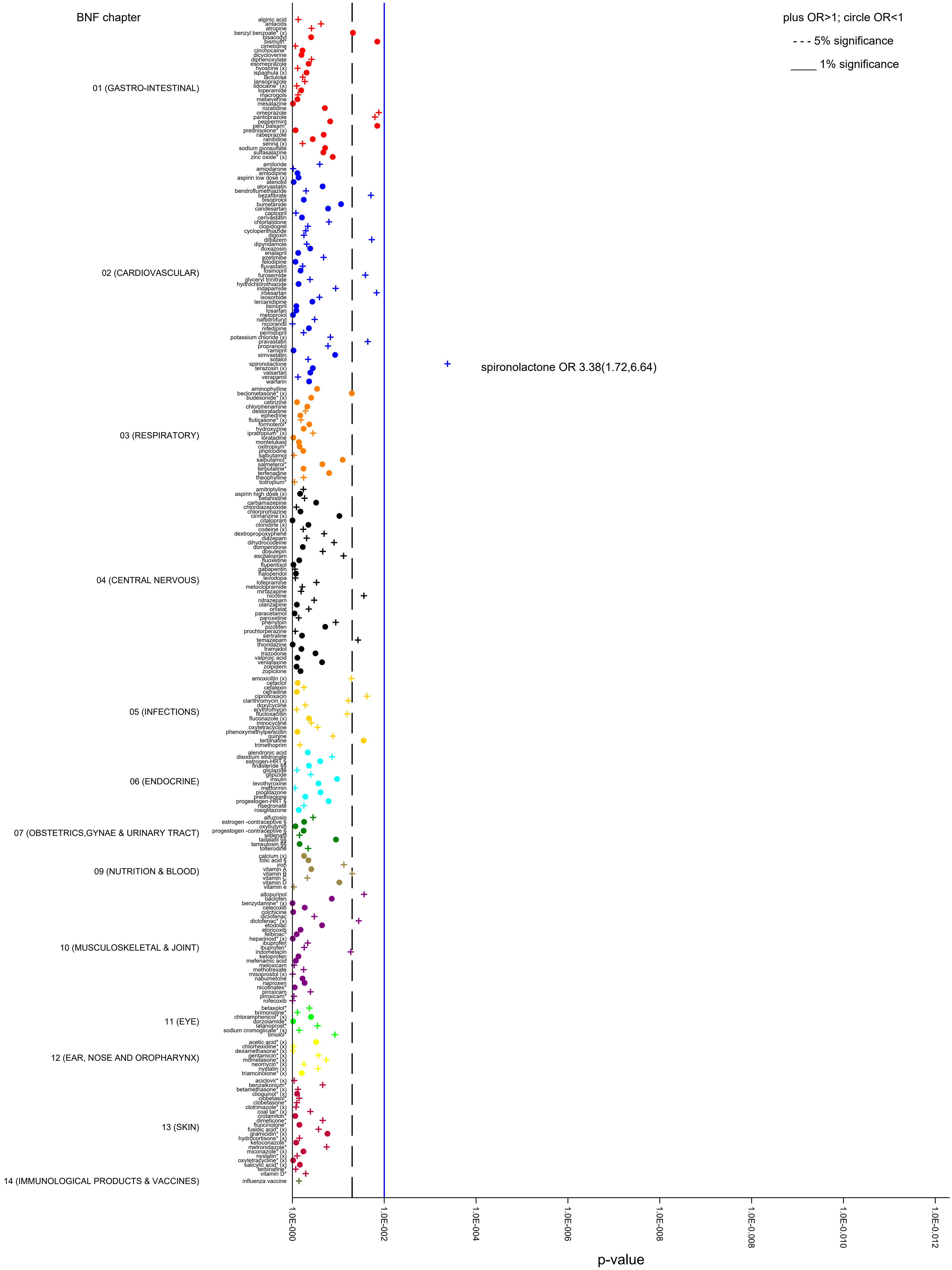

* : local medicine; § females only; §§ males only; (x) most prevalent chapter

MWAS plot: thyroid cancer
Comorbidity adjusted analysis (exposure any prescription)

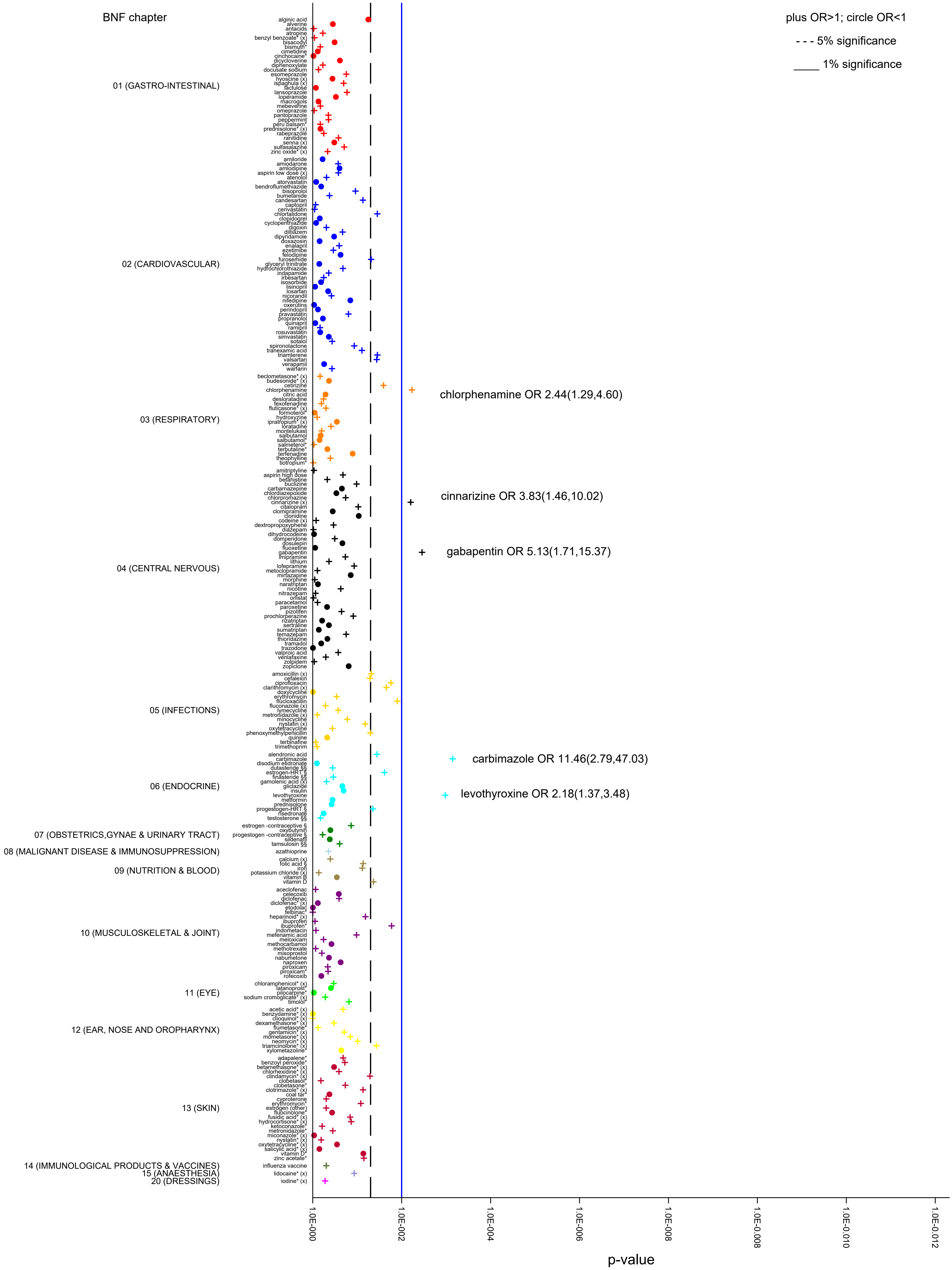

* : local medicine; § females only; §§ males only; (x) most prevalent chapter

MWAS plot: anal cancer
Comorbidity adjusted analysis (exposure any prescription)

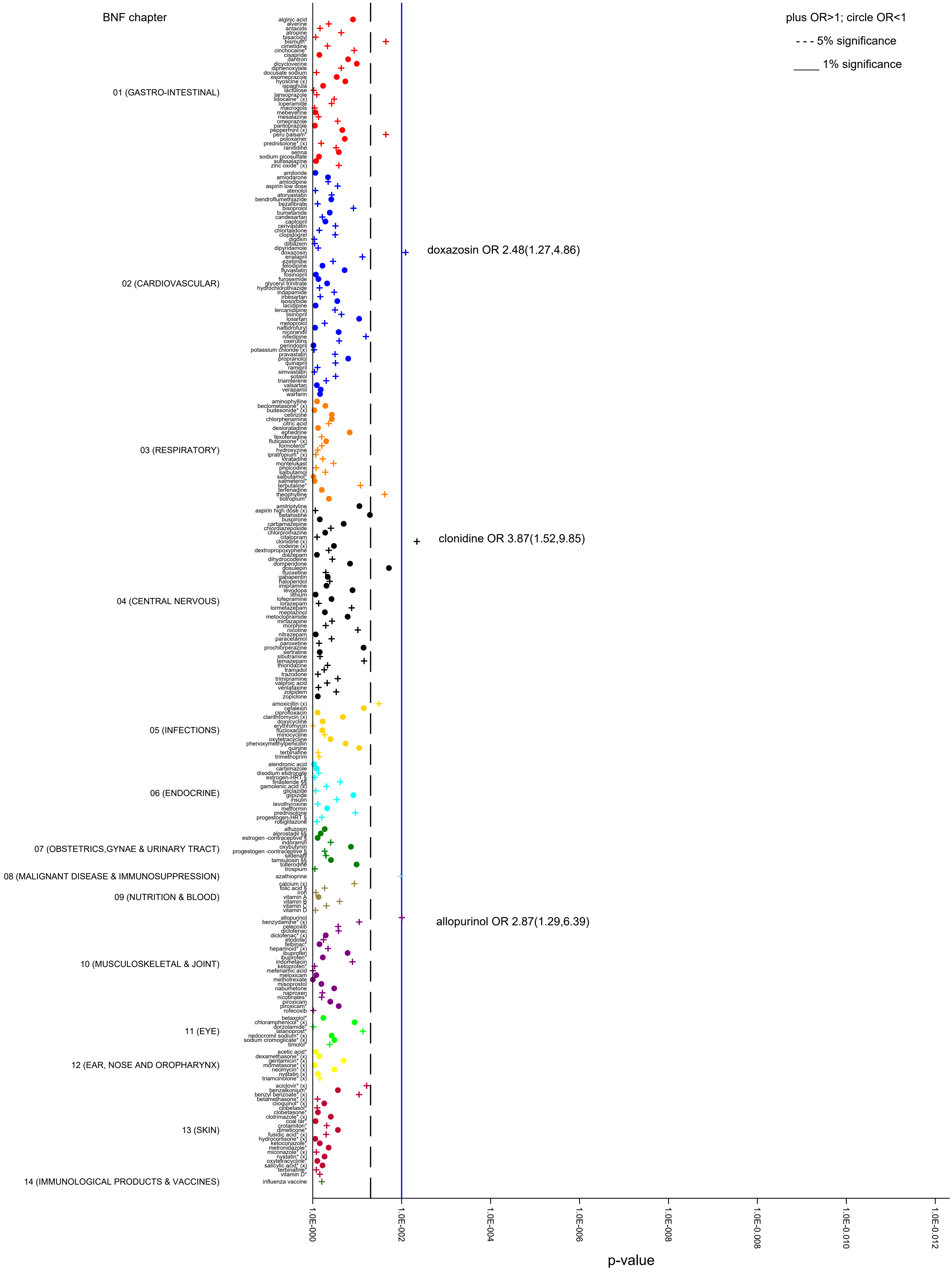

* : local medicine; § females only; §§ males only; (x) most prevalent chapter

Fig S2: MWAS plots for comorbidity & smoking adjusted analyses: exposure any prescription

MWAS plot: breast cancer
Comorbidity & smoking adjusted analysis (exposure any prescription)

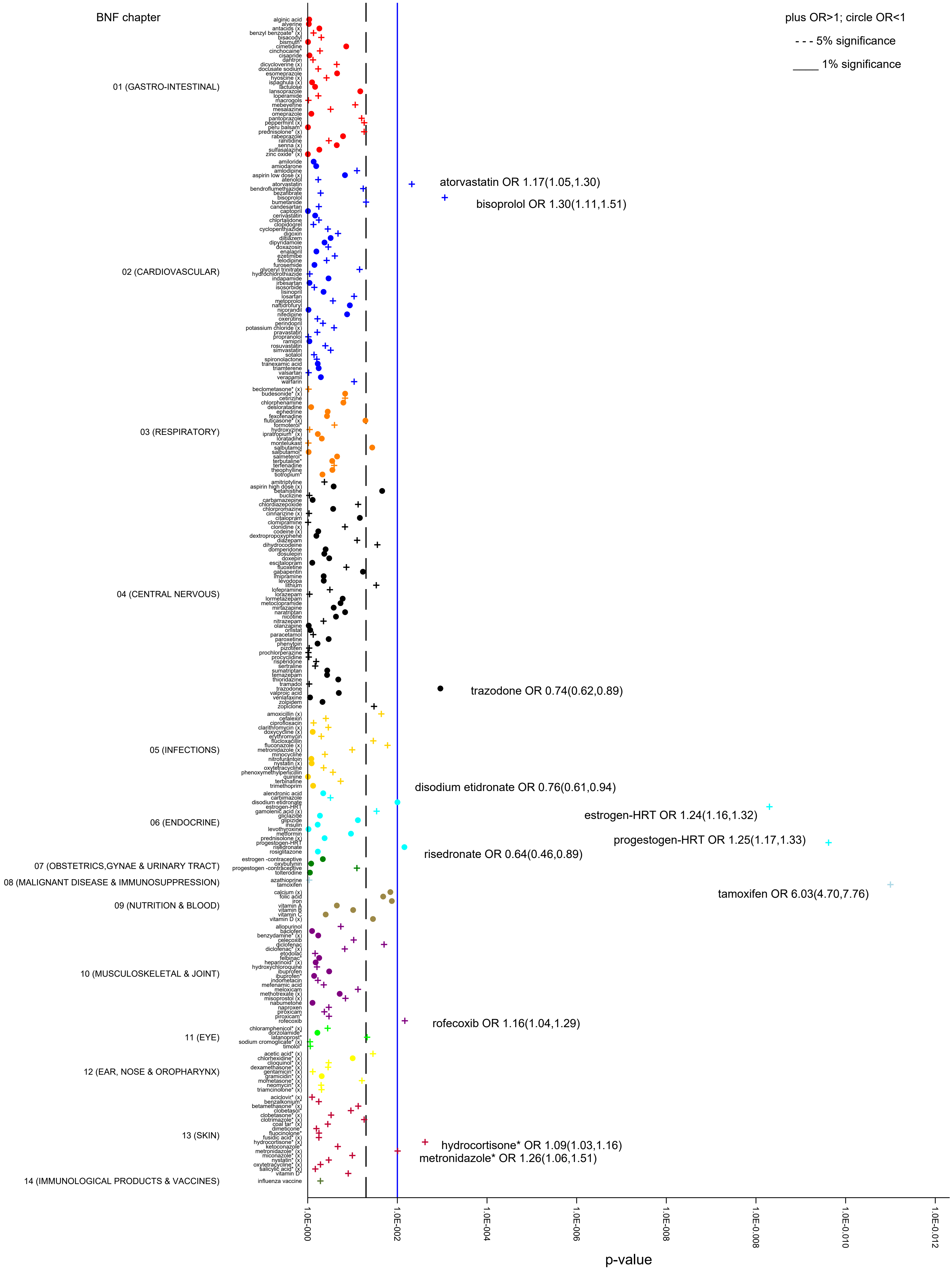

* : local medicine; females only included in this analysis; (l): local; (x) most prevalent chapter

MWAS plot: lung cancer
Comorbidity & smoking adjusted analysis (exposure any prescription)

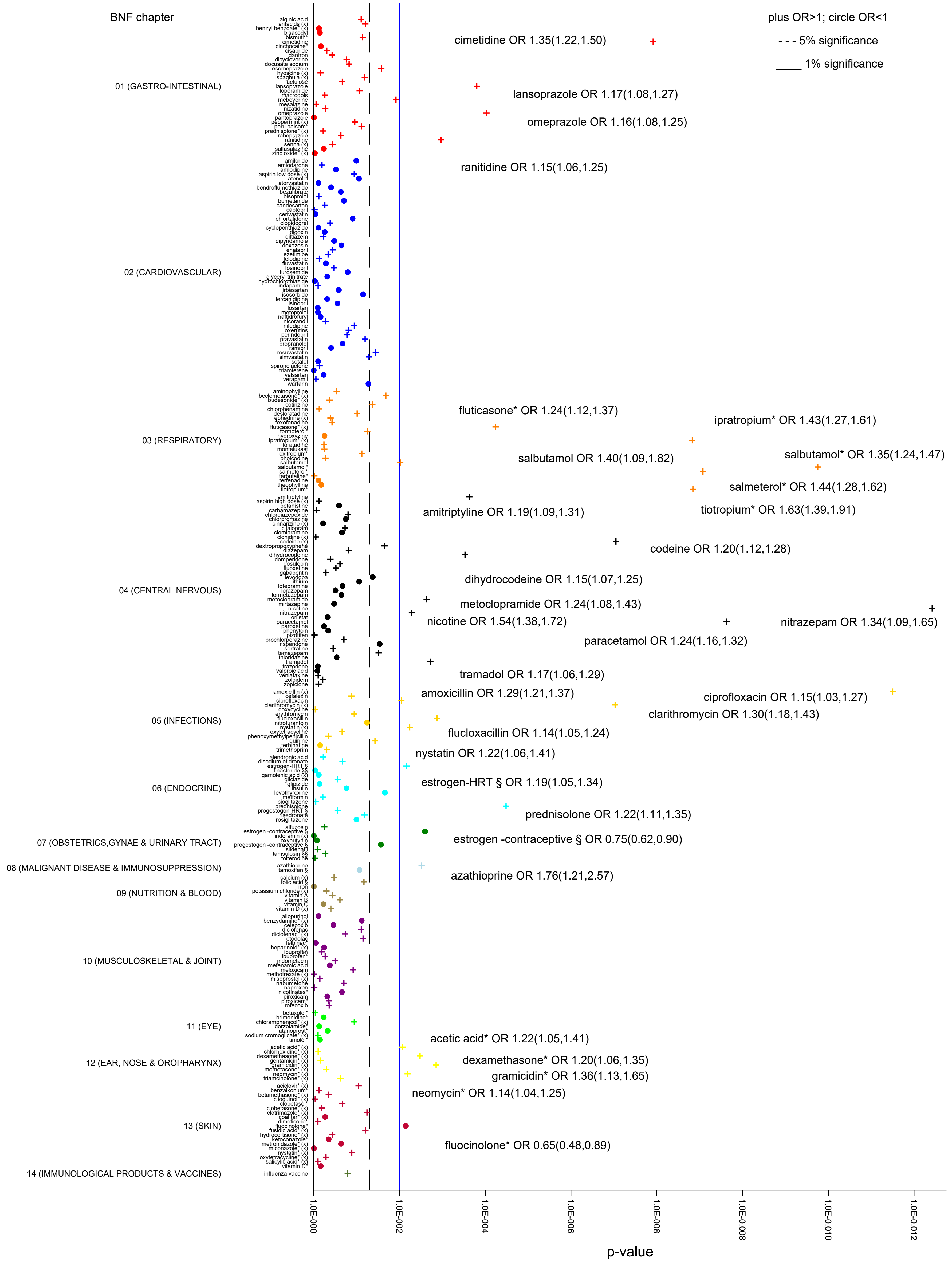

* : local medicine; § females only; §§ males only; (x) most prevalent chapter

MWAS plot: colorectal cancer
Comorbidity & smoking adjusted analysis (exposure any prescription)

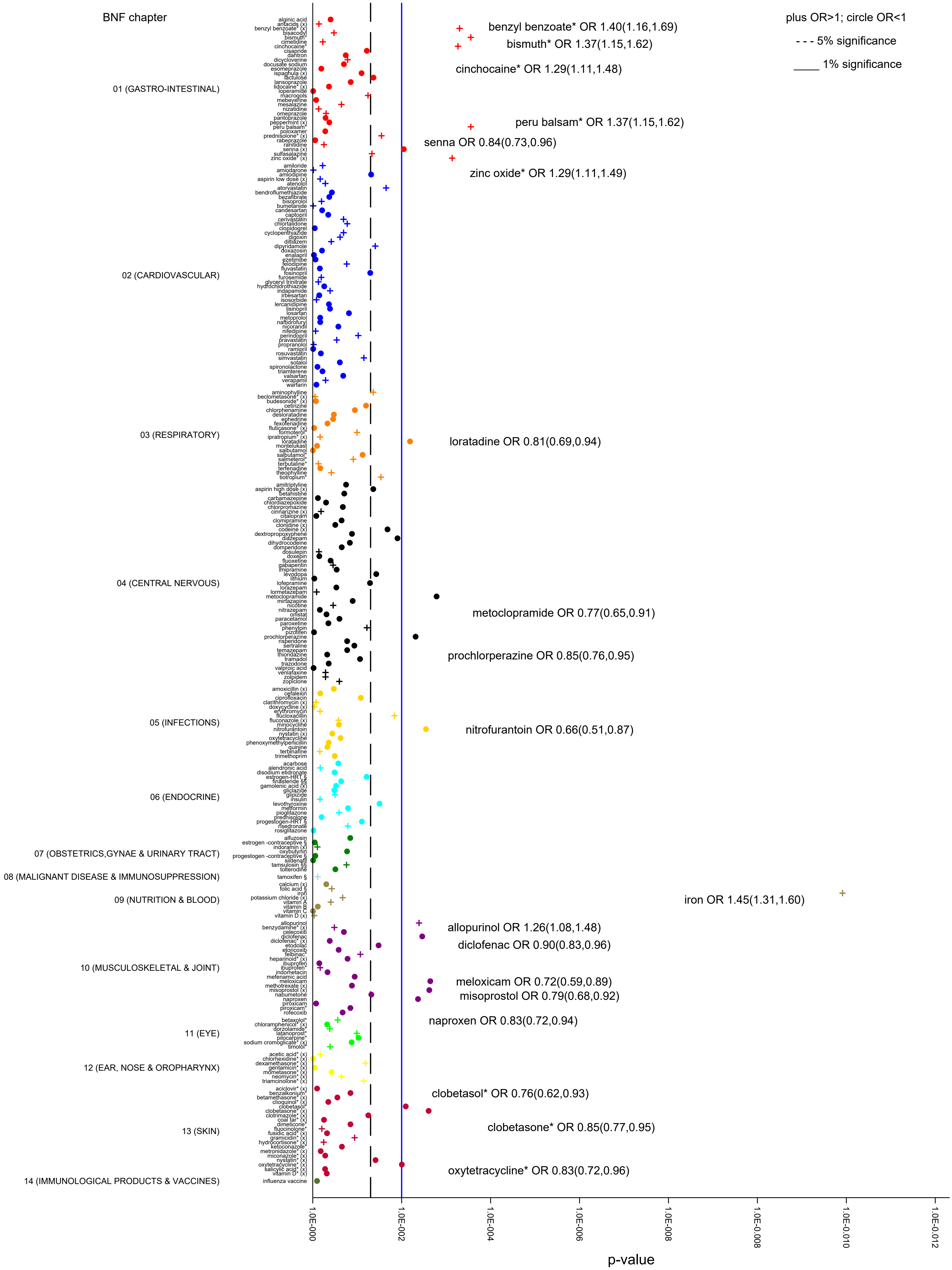

* : local medicine; § females only; §§ males only; (x) most prevalent chapter

MWAS plot: prostate cancer
Comorbidity & smoking adjusted analysis (exposure any prescription)

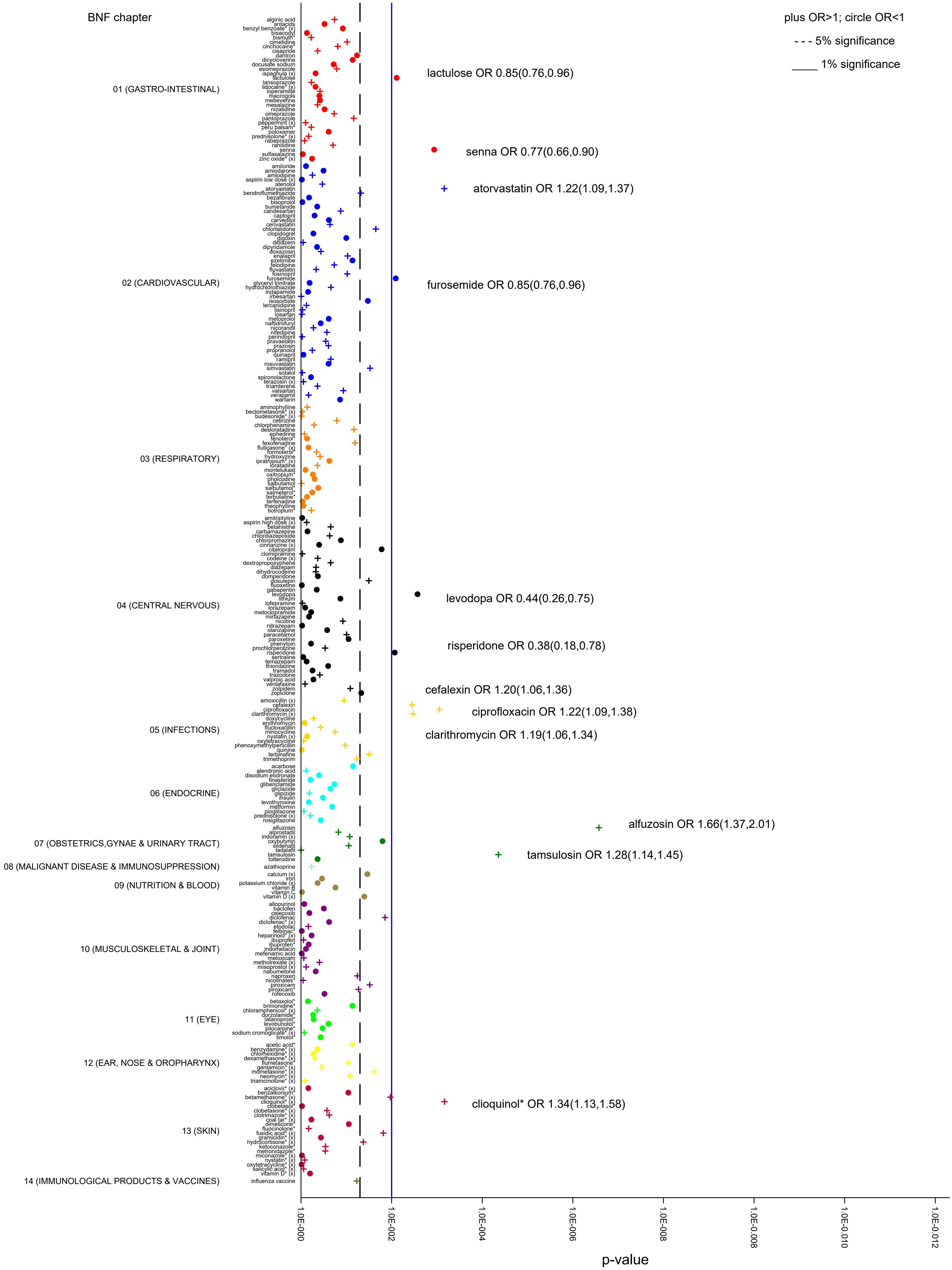

* : local medicine; males only included in this analysis; (l): local; (x) most prevalent chapter

MWAS plot: bladder cancer
Comorbidity & smoking adjusted analysis (exposure any prescription)

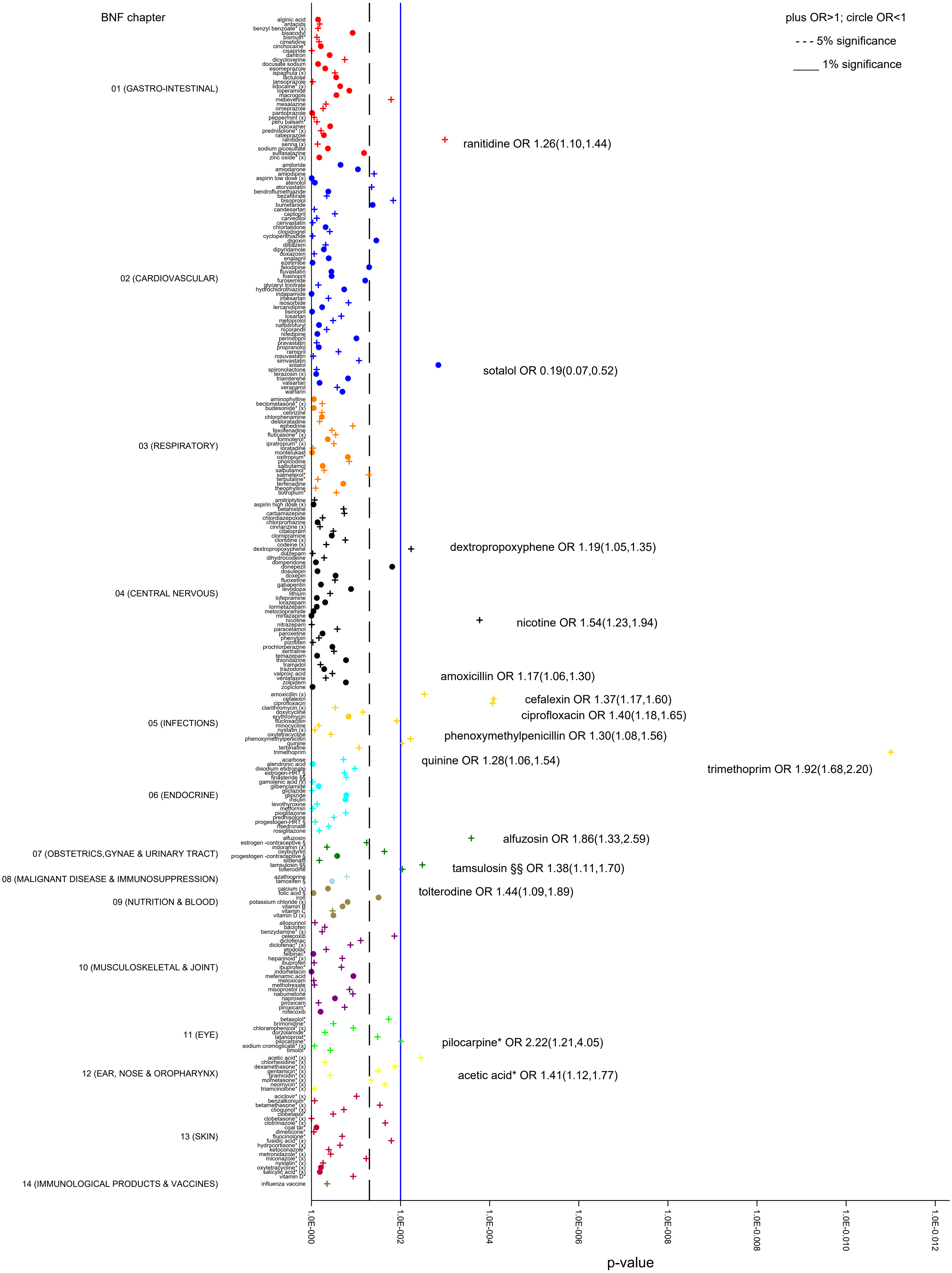

* : local medicine; § females only; §§ males only; (x) most prevalent chapter

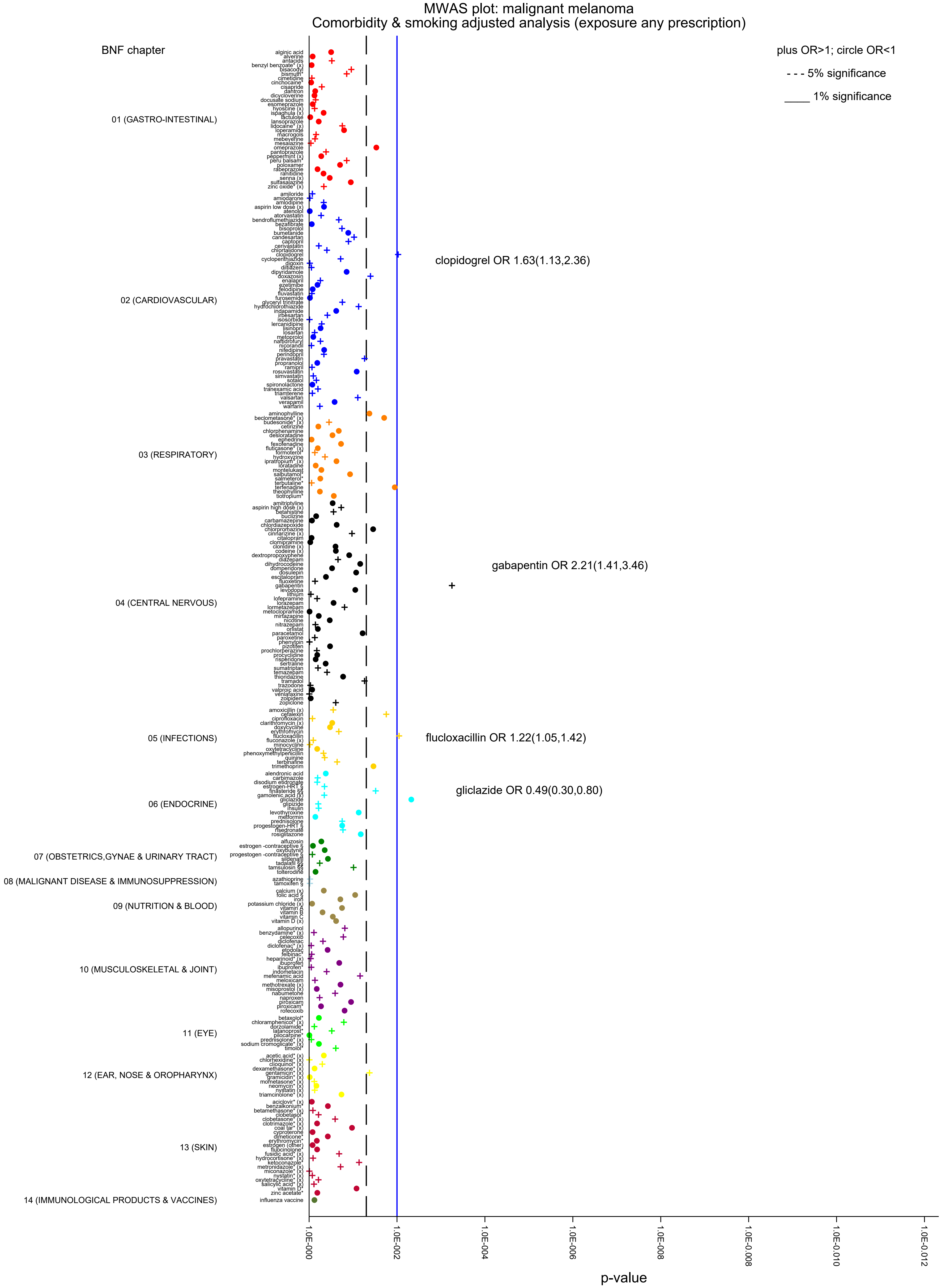

* : local medicine; § females only; §§ males only; (x) most prevalent chapter

MWAS plot: oesophageal cancer
Comorbidity & smoking adjusted analysis (exposure any prescription)

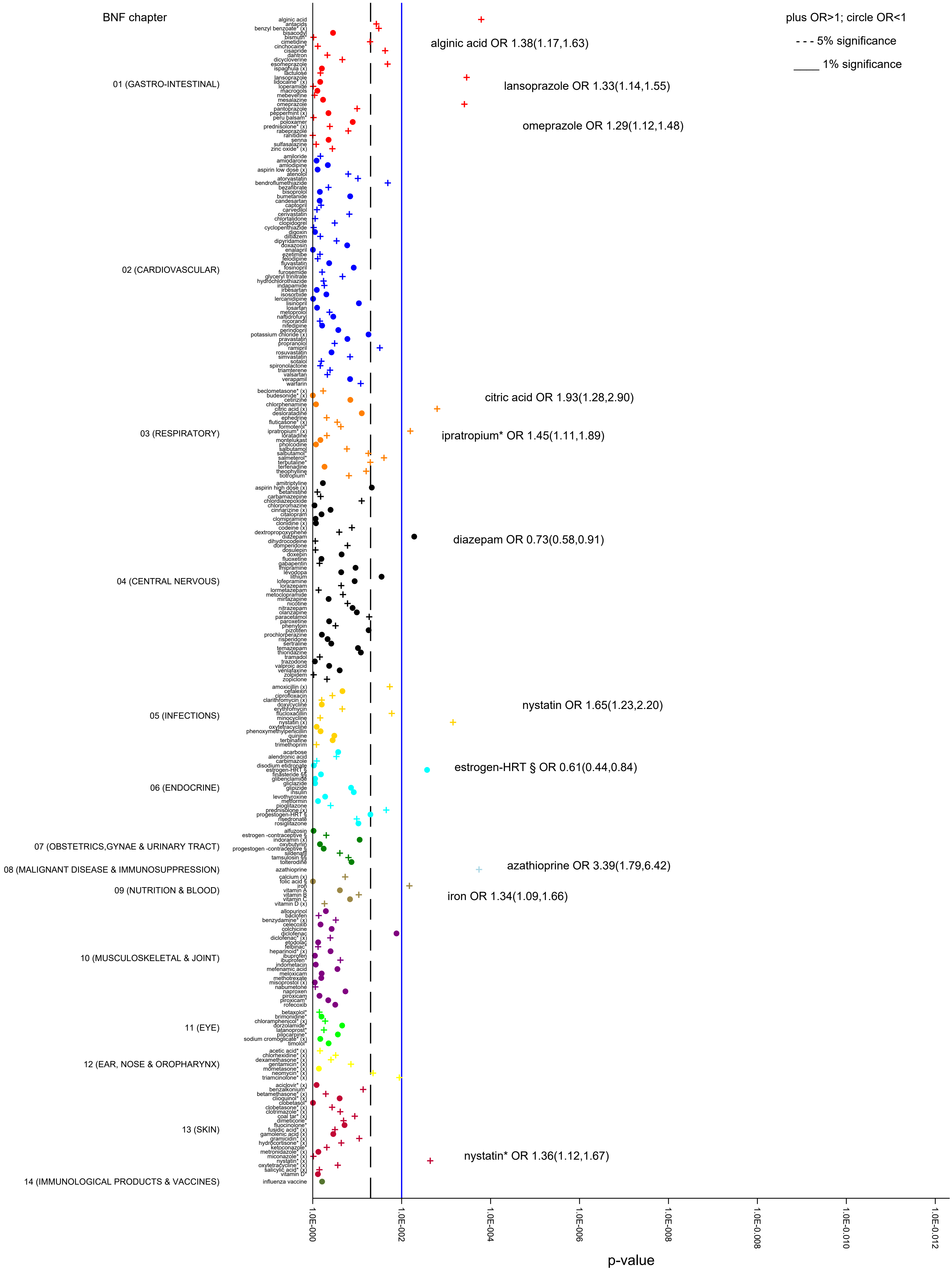

* : local medicine; § females only; §§ males only; (x) most prevalent chapter

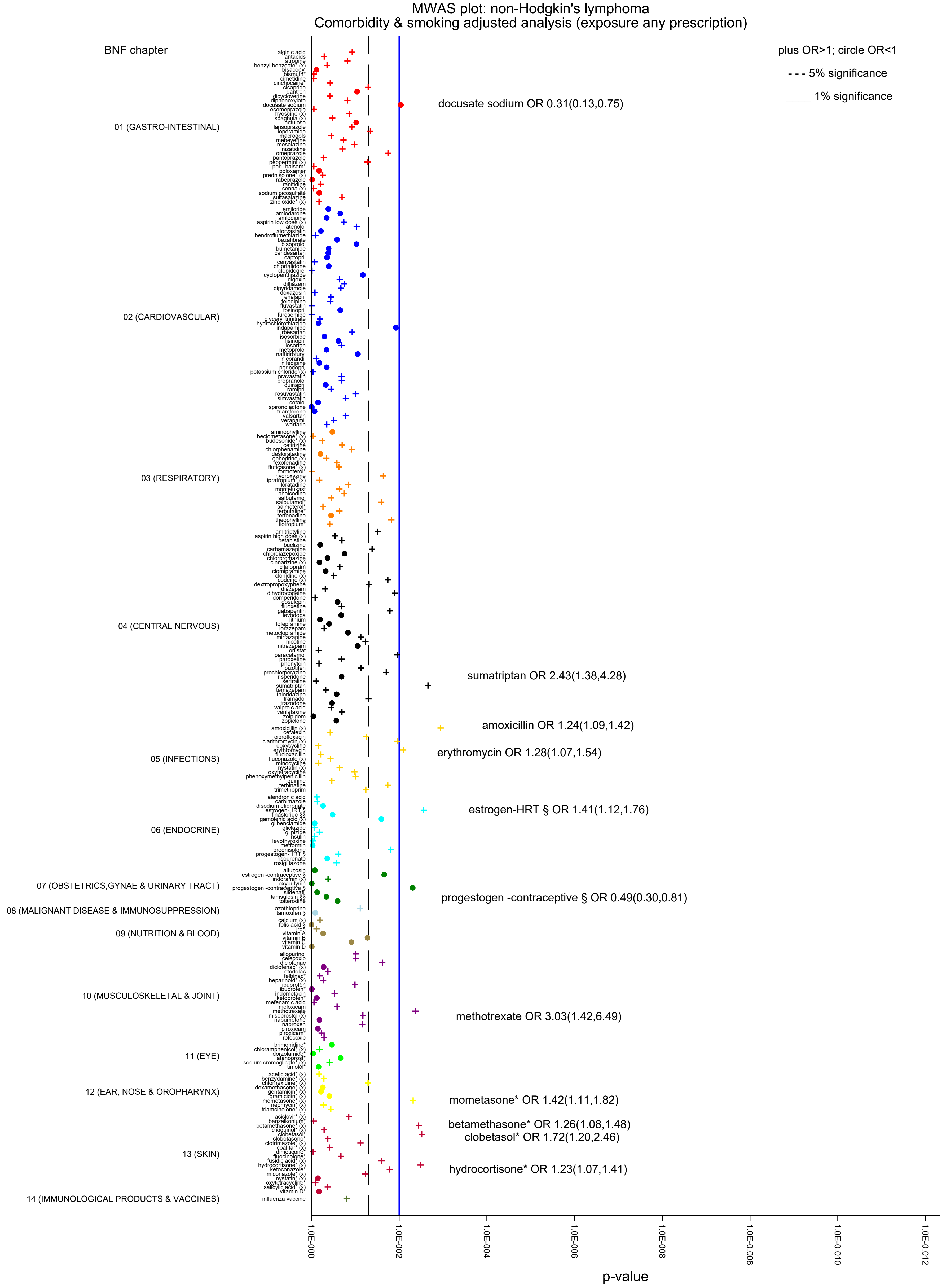

* : local medicine; § females only; §§ males only; (x) most prevalent chapter

MWAS plot: leukaemia
Comorbidity & smoking adjusted analysis (exposure any prescription)

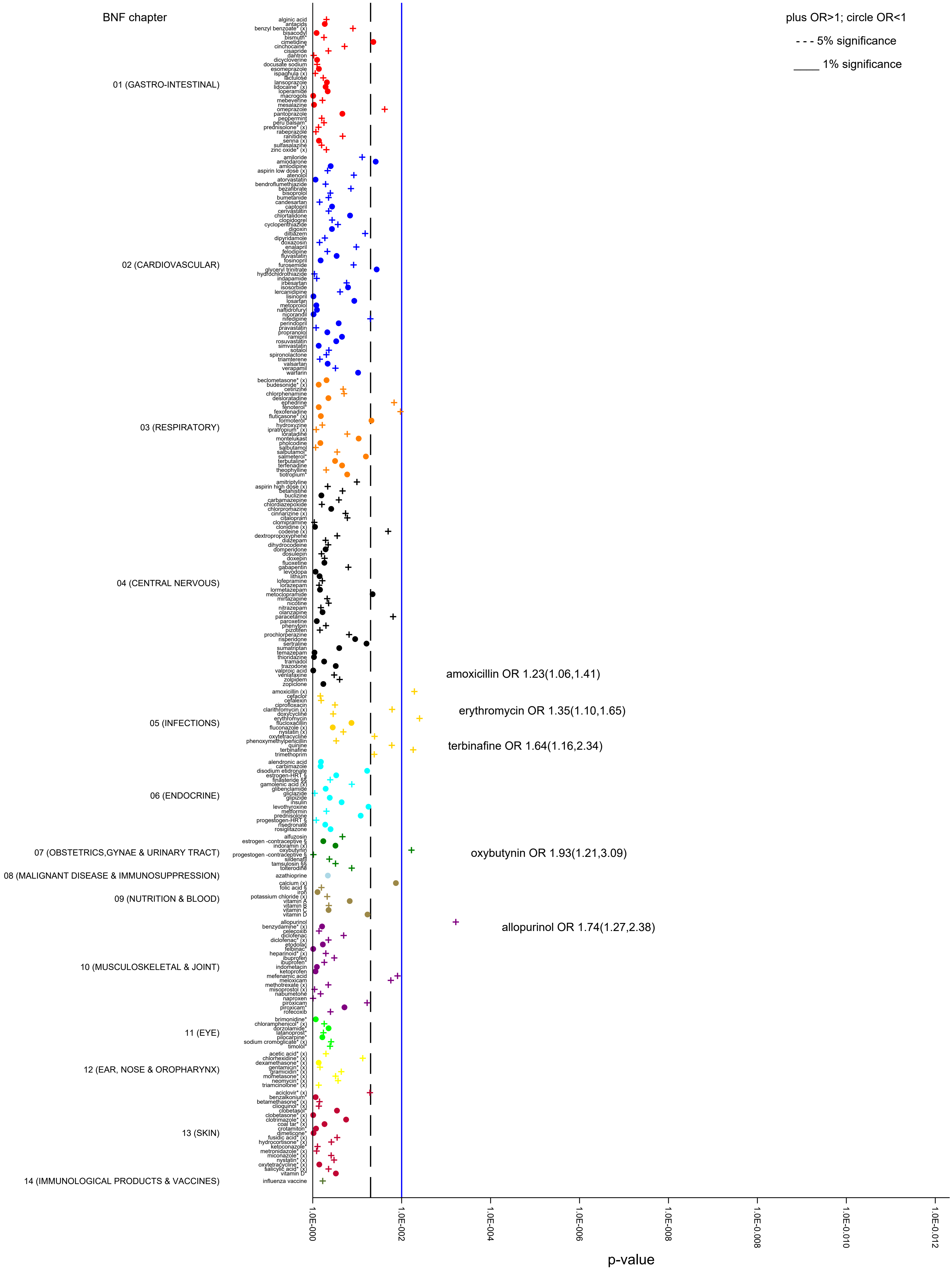

* : local medicine; § females only; §§ males only; (x) most prevalent chapter

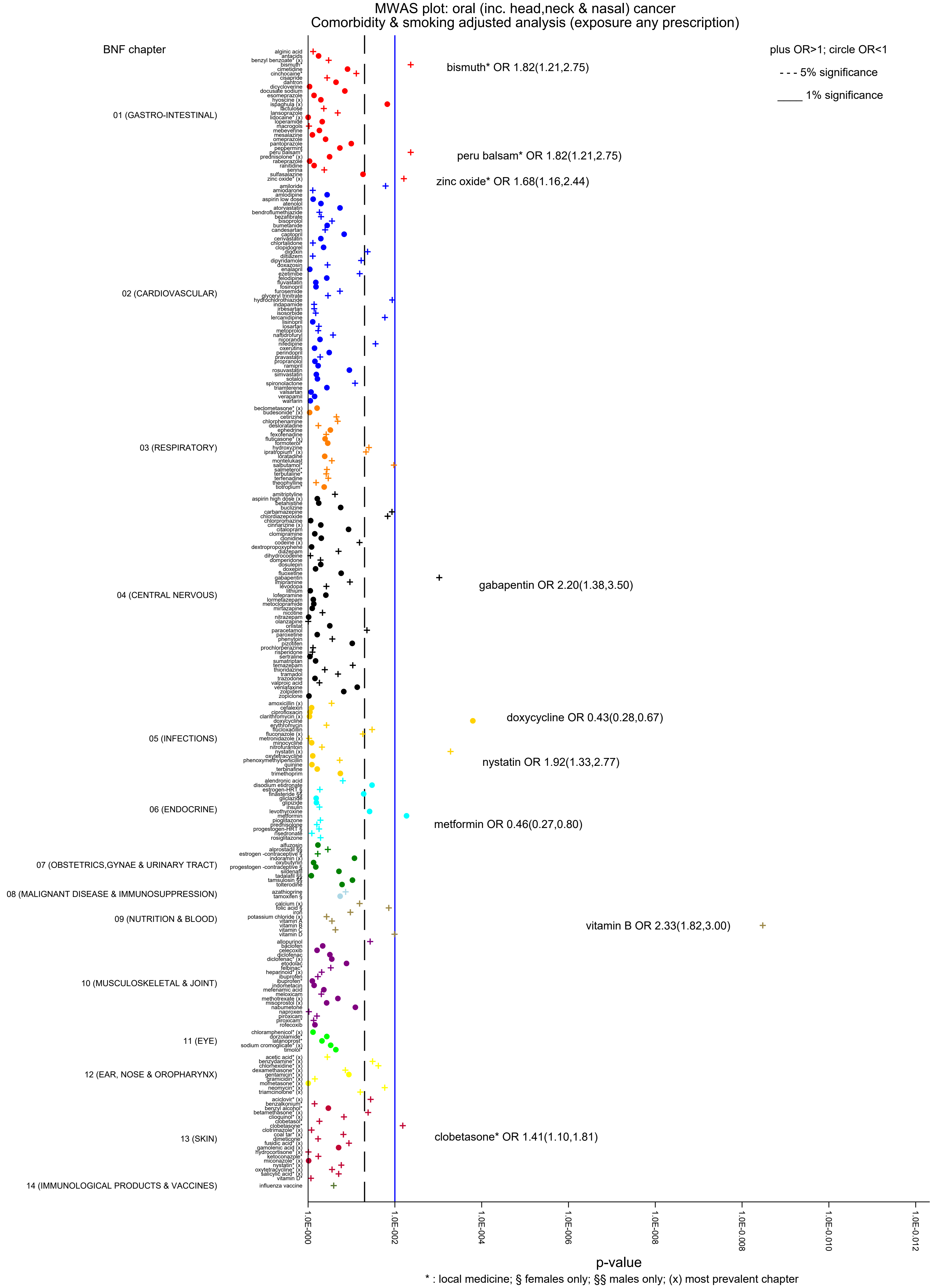

* : local medicine; § females only; §§ males only; (x) most prevalent chapter

MWAS plot: ovarian cancer
Comorbidity & smoking adjusted analysis (exposure any prescription)

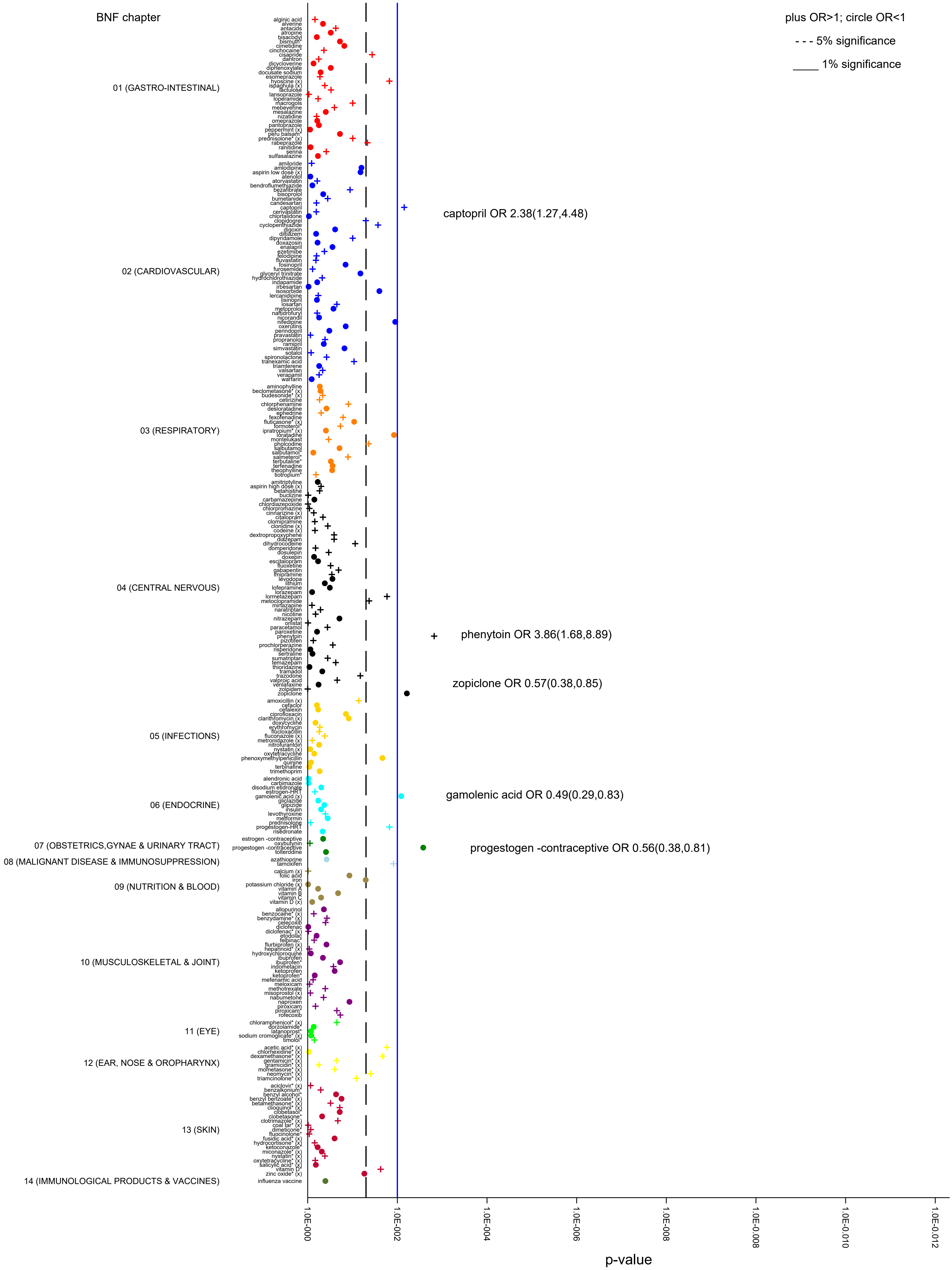

* : local medicine; females only included in this analysis; (l): local; (x) most prevalent chapter

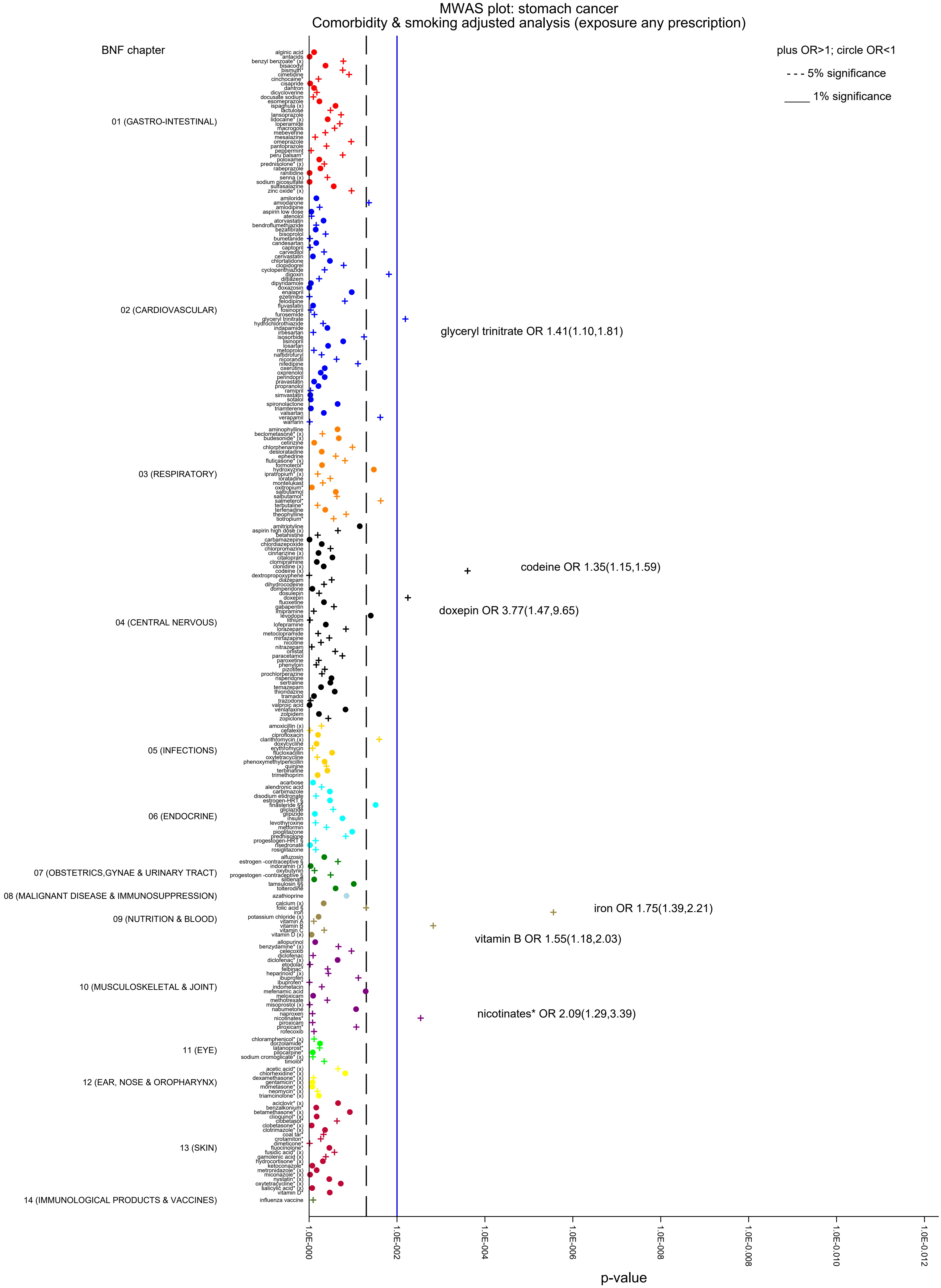

* : local medicine; § females only; §§ males only; (x) most prevalent chapter

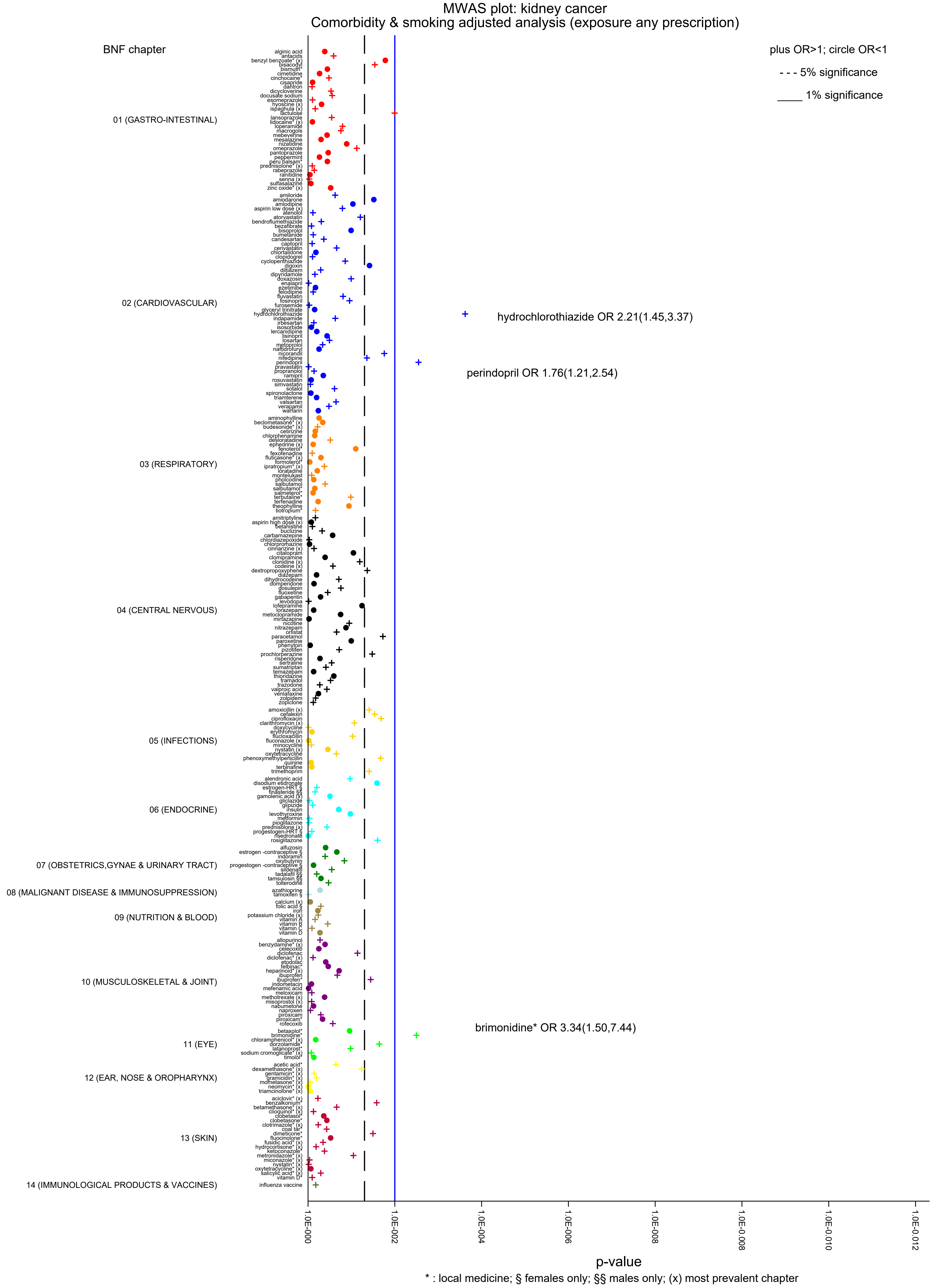
MWAS plot: kidney cancer
Comorbidity & smoking adjusted analysis (exposure any prescription)
BNF chapter
plus OR>1; circle OR<1
- - - 5% significance
____ 1% significance
01 (GASTRO-INTESTINAL)
02 (CARDIOVASCULAR)
03 (RESPIRATORY)
04 (CENTRAL NERVOUS)
05 (INFECTIONS)
06 (ENDOCRINE)
07 (OBSTETRICS,GYNAE & URINARY TRACT)
08 (MALIGNANT DISEASE & IMMUNOSUPPRESSION)
09 (NUTRITION & BLOOD)
10 (MUSCULOSKELETAL & JOINT)
11 (EYE)
12 (EAR, NOSE & OROPHARYNX)
13 (SKIN)
14 (IMMUNOLOGICAL PRODUCTS & VACCINES)
hydrochlorothiazide OR 2.21(1.45,3.37)
perindopril OR 1.76(1.21,2.54)
brimonidine* OR 3.34(1.50,7.44)
1.0E-000
1.0E-002
1.0E-004
1.0E-006
1.0E-008
1.0E-010
1.0E-012
p-value
* : local medicine; § females only; §§ males only; (x) most prevalent chapter

MWAS plot: cervical & other gynae cancers
Comorbidity & smoking adjusted analysis (exposure any prescription)

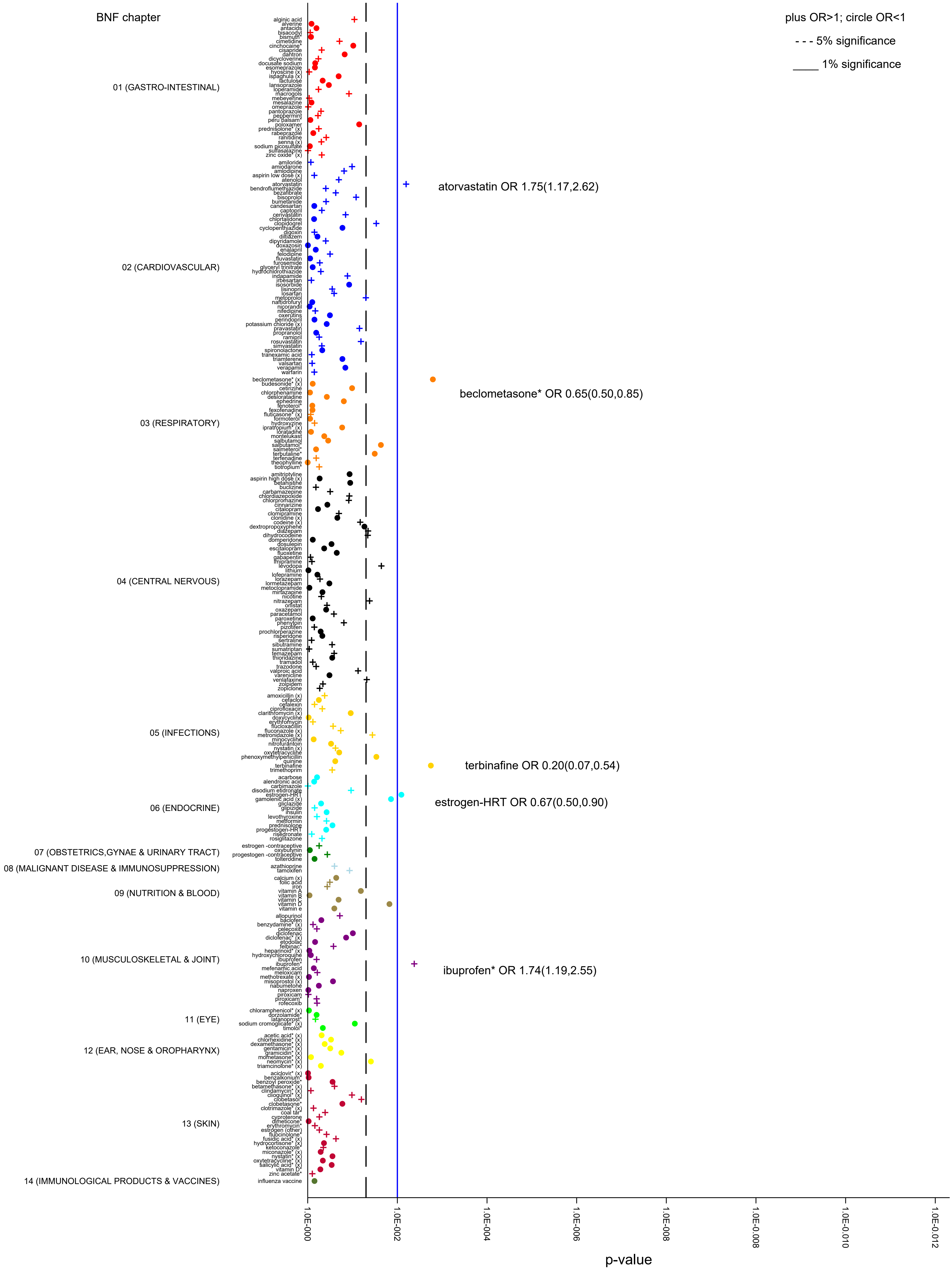

* : local medicine; females only included in this analysis; (l): local; (x) most prevalent chapter

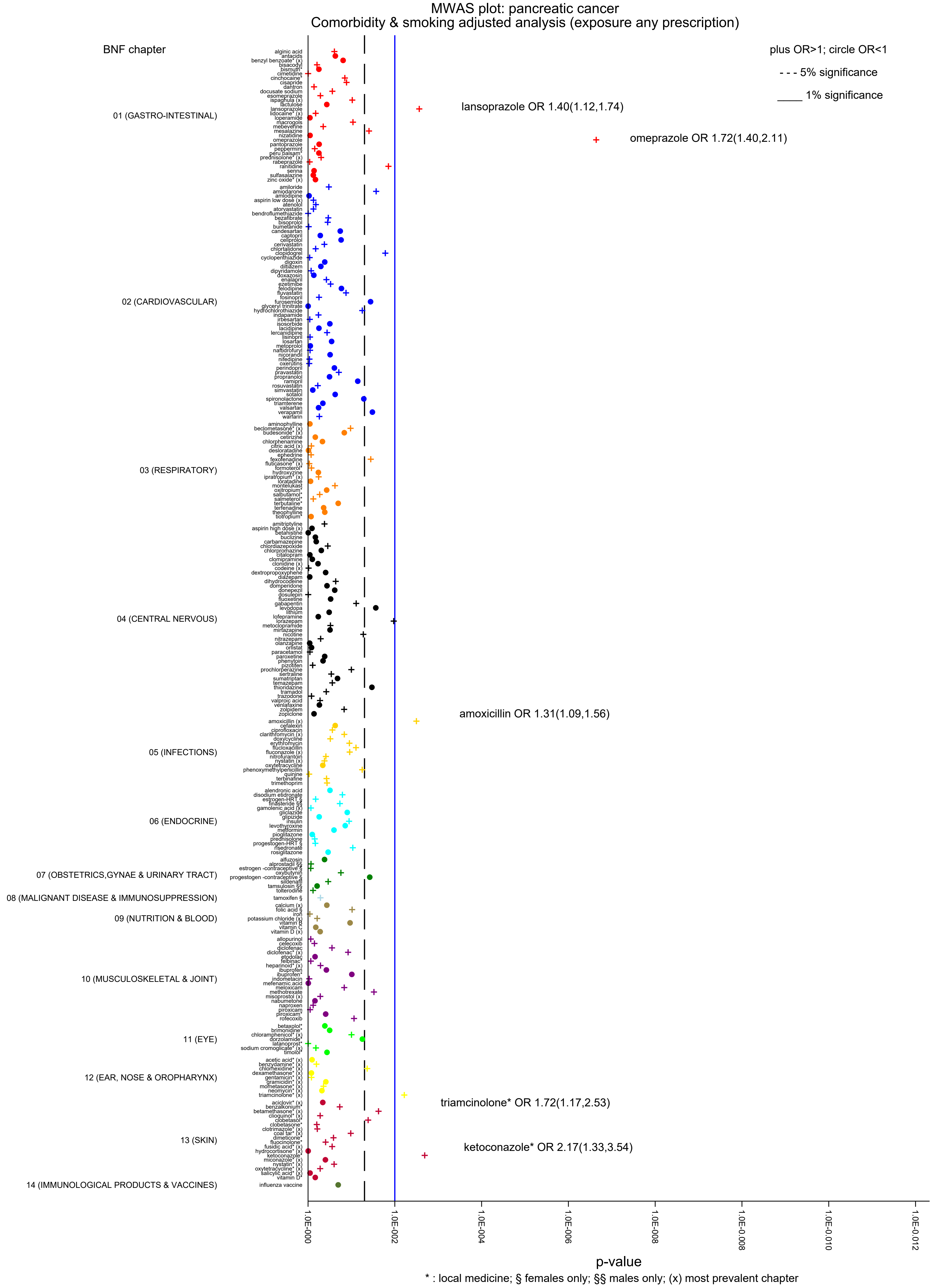
MWAS plot: pancreatic cancer
Comorbidity & smoking adjusted analysis (exposure any prescription)
BNF chapter
plus OR>1; circle OR<1
- - - 5% significance
____ 1% significance
lansoprazole OR 1.40(1.12,1.74)
omeprazole OR 1.72(1.40,2.11)
amoxicillin OR 1.31(1.09,1.56)
triamcinolone* OR 1.72(1.17,2.53)
ketoconazole* OR 2.17(1.33,3.54)
01 (GASTRO-INTESTINAL)
02 (CARDIOVASCULAR)
03 (RESPIRATORY)
04 (CENTRAL NERVOUS)
05 (INFECTIONS)
06 (ENDOCRINE)
07 (OBSTETRICS,GYNAE & URINARY TRACT)
08 (MALIGNANT DISEASE & IMMUNOSUPPRESSION)
09 (NUTRITION & BLOOD)
10 (MUSCULOSKELETAL & JOINT)
11 (EYE)
12 (EAR, NOSE & OROPHARYNX)
13 (SKIN)
14 (IMMUNOLOGICAL PRODUCTS & VACCINES)
1.0E-000
1.0E-002
1.0E-004
1.0E-006
1.0E-008
1.0E-0.008
1.0E-0.010
1.0E-0.012
p-value
* : local medicine; § females only; §§ males only; (x) most prevalent chapter

MWAS plot: cancer of the uterus
Comorbidity & smoking adjusted analysis (exposure any prescription)

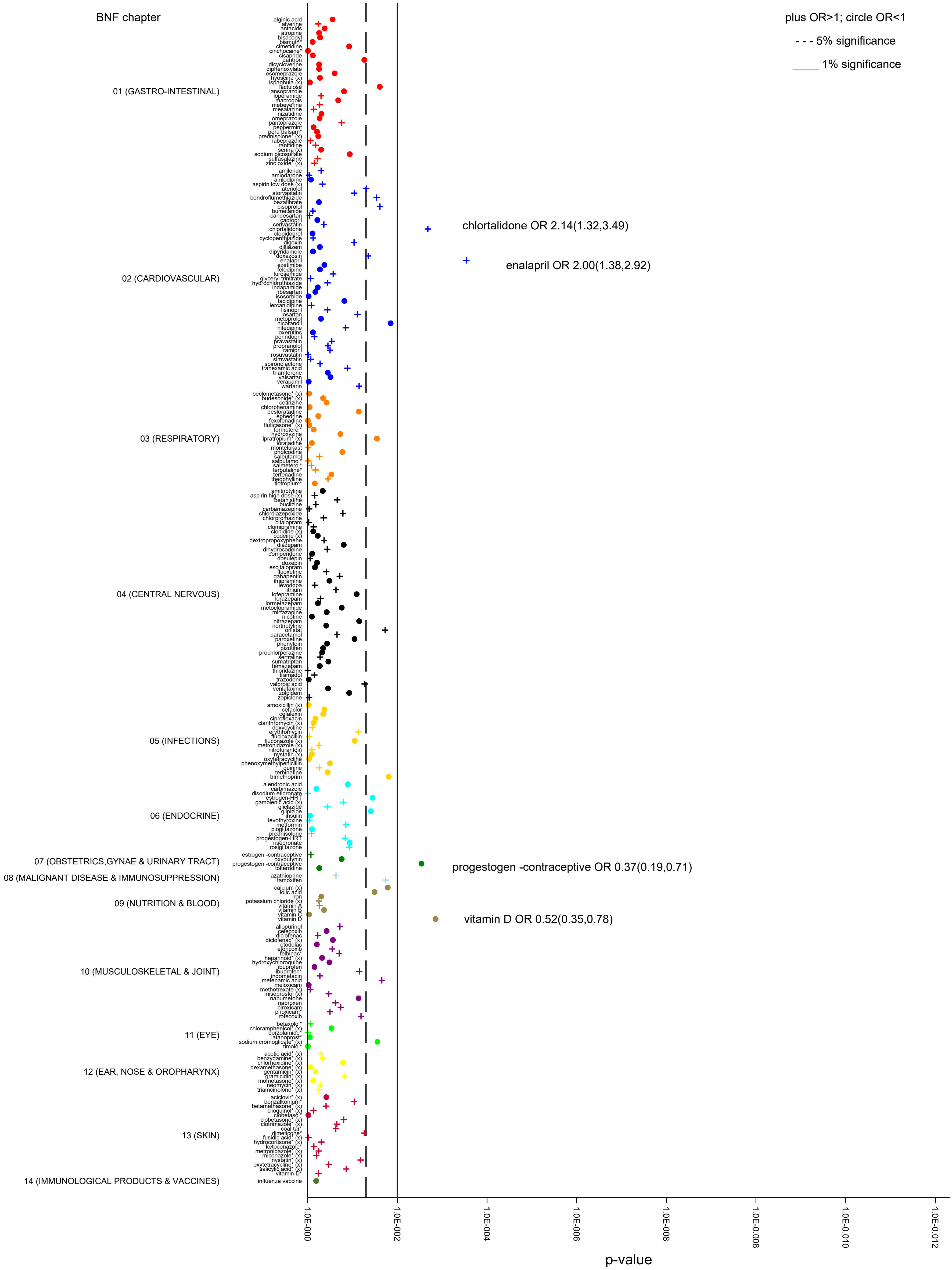

* : local medicine; females only included in this analysis; (l): local; (x) most prevalent chapter

MWAS plot: cancer of the brain and central nervous system
Comorbidity & smoking adjusted analysis (exposure any prescription)

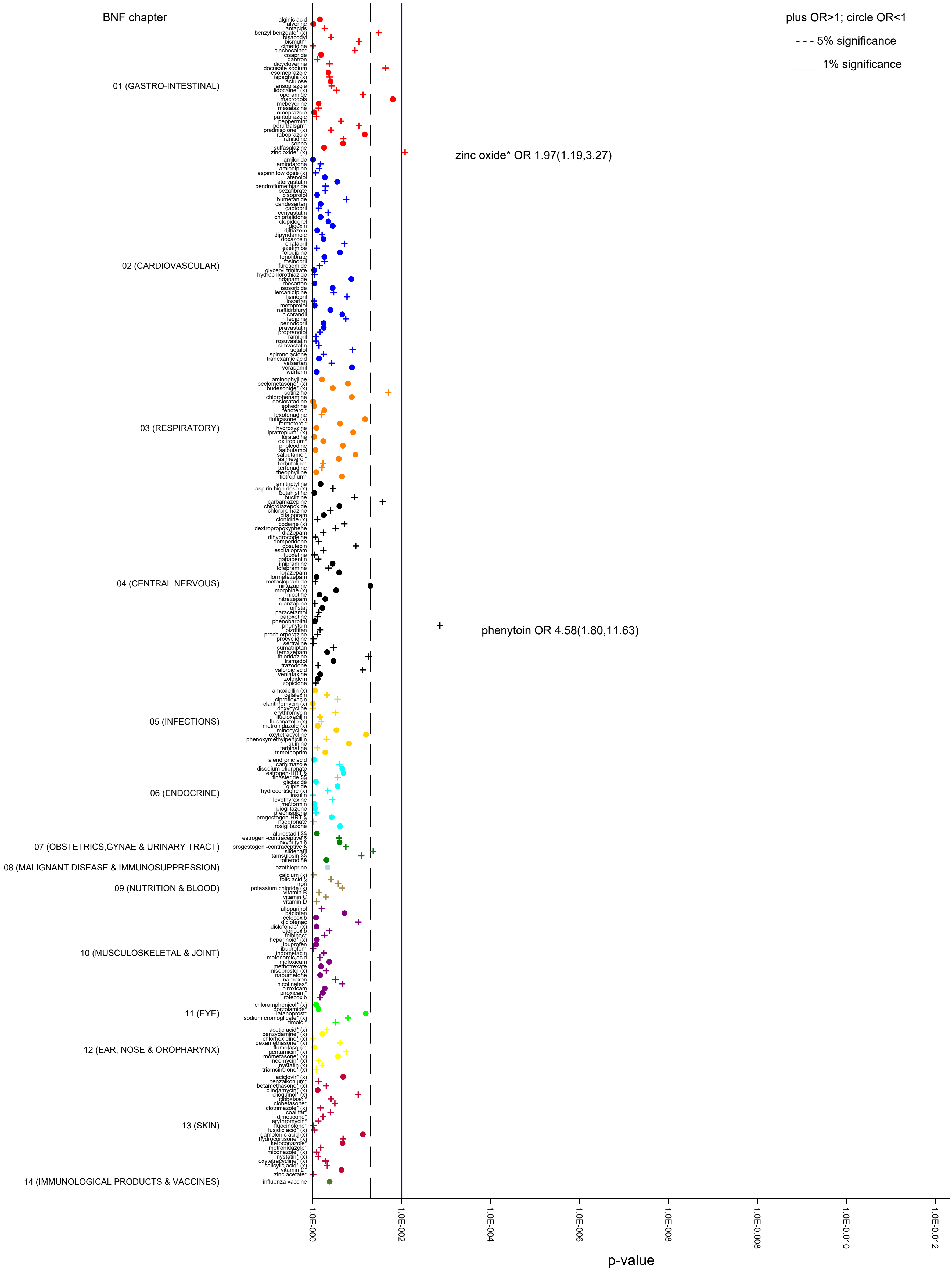

* : local medicine; § females only; §§ males only; (x) most prevalent chapter

MWAS plot: laryngeal cancer
Comorbidity & smoking adjusted analysis (exposure any prescription)

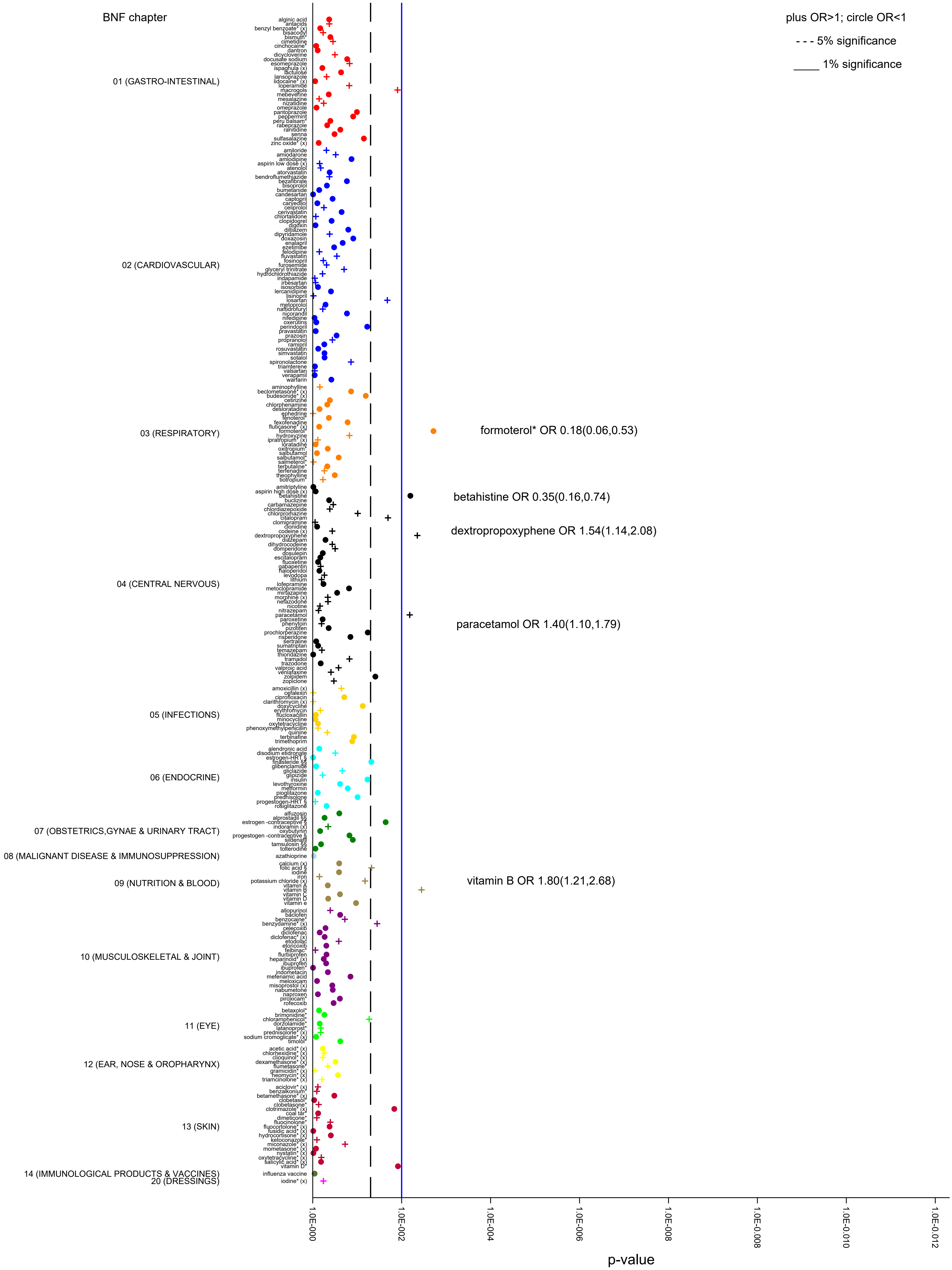

* : local medicine; § females only; §§ males only; (x) most prevalent chapter

MWAS plot: multiple myeloma
Comorbidity & smoking adjusted analysis (exposure any prescription)

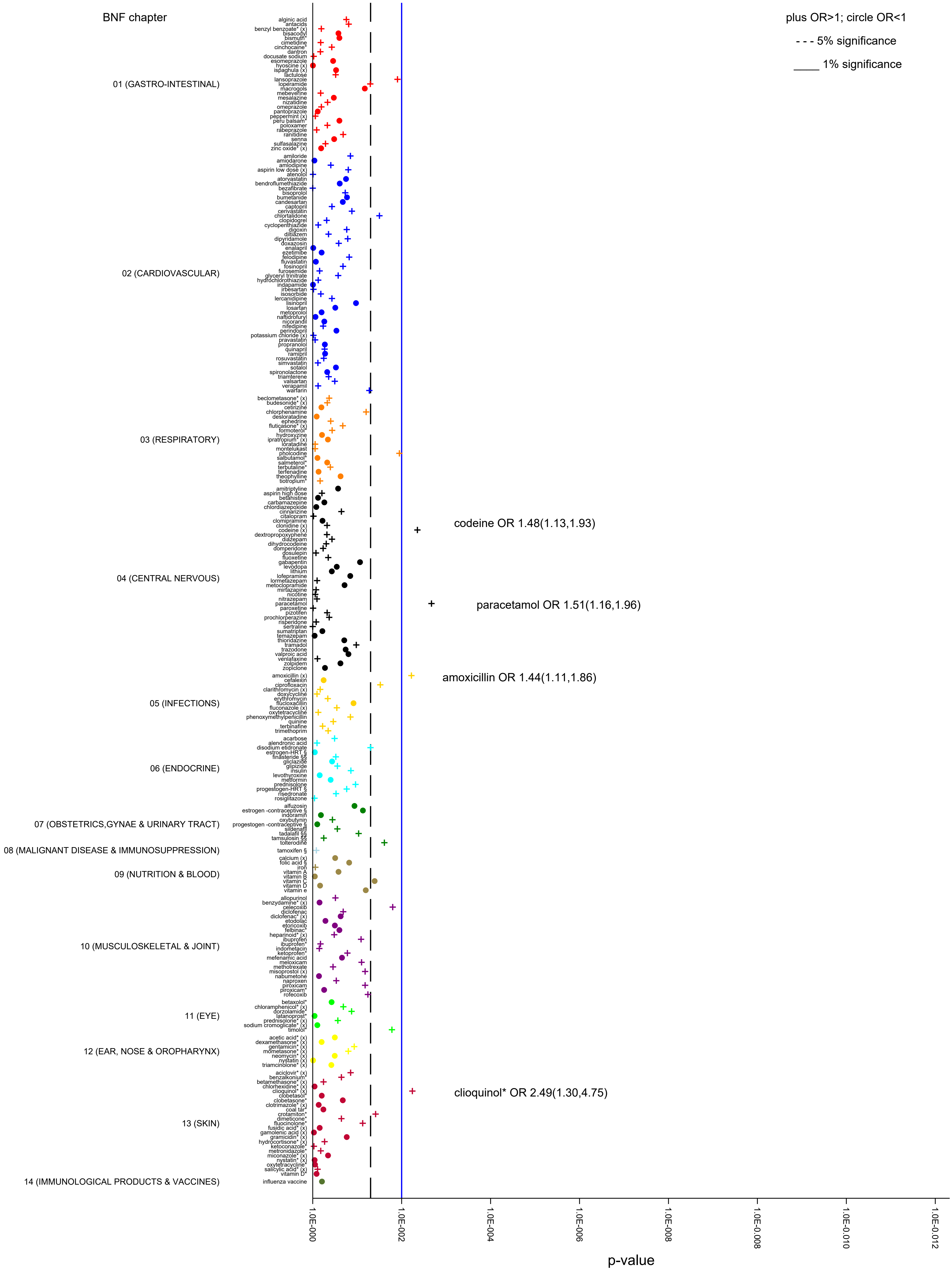

* : local medicine; § females only; §§ males only; (x) most prevalent chapter

MWAS plot: liver cancer
Comorbidity & smoking adjusted analysis (exposure any prescription)

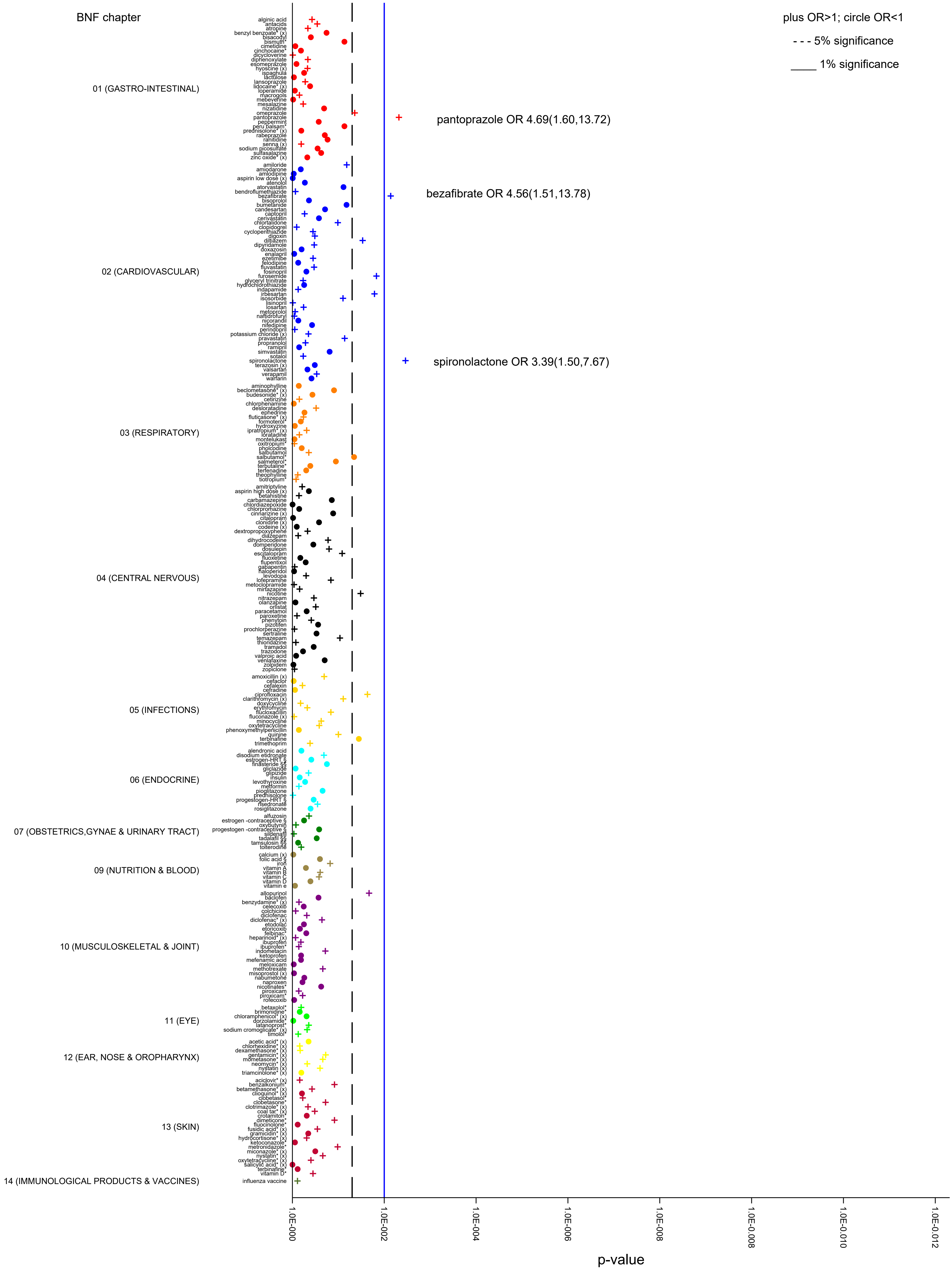

* : local medicine; § females only; §§ males only; (x) most prevalent chapter

MWAS plot: thyroid cancer
Comorbidity & smoking adjusted analysis (exposure any prescription)

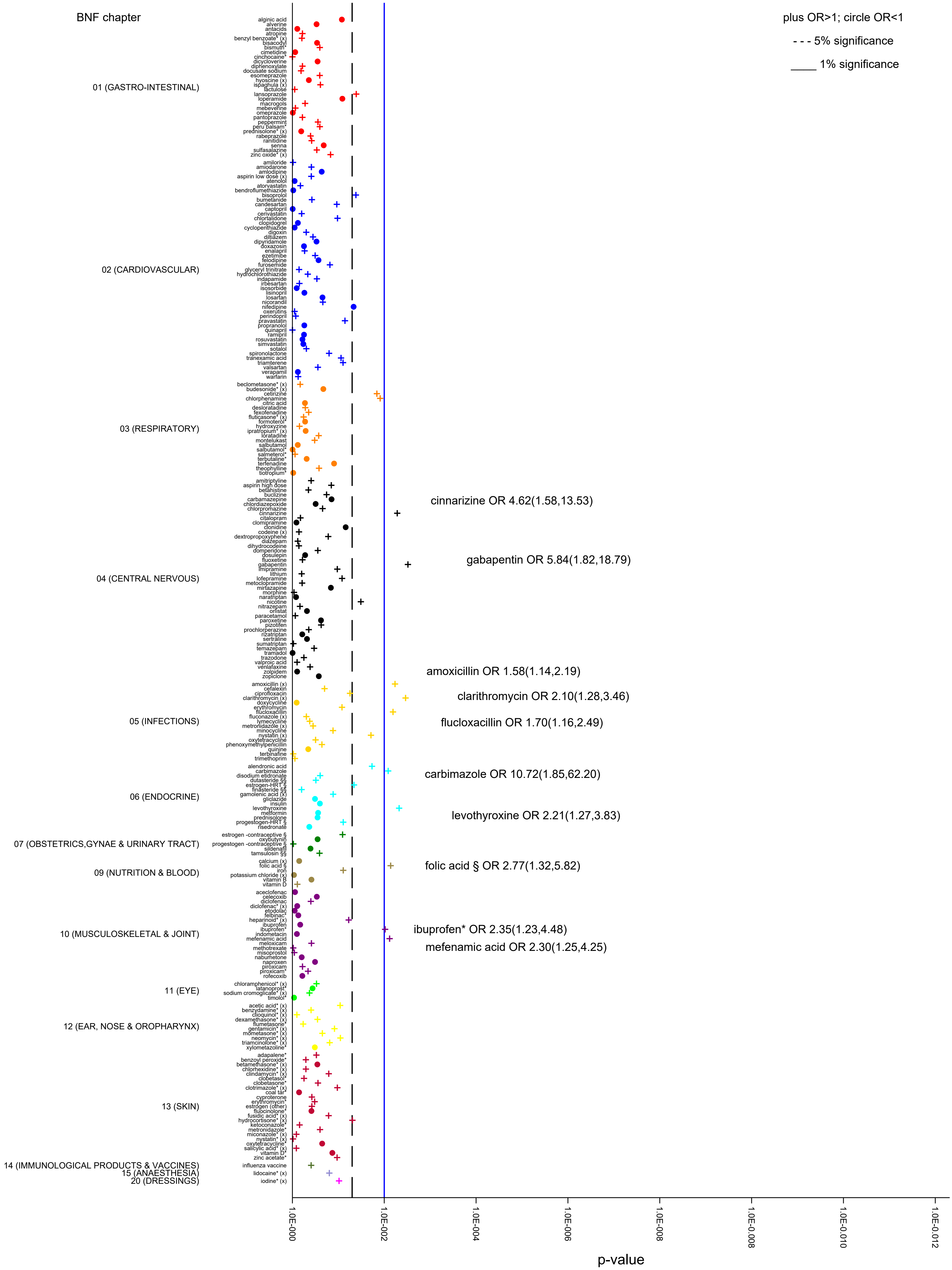

* : local medicine; § females only; §§ males only; (x) most prevalent chapter

MWAS plot: anal cancer
Comorbidity & smoking adjusted analysis (exposure any prescription)

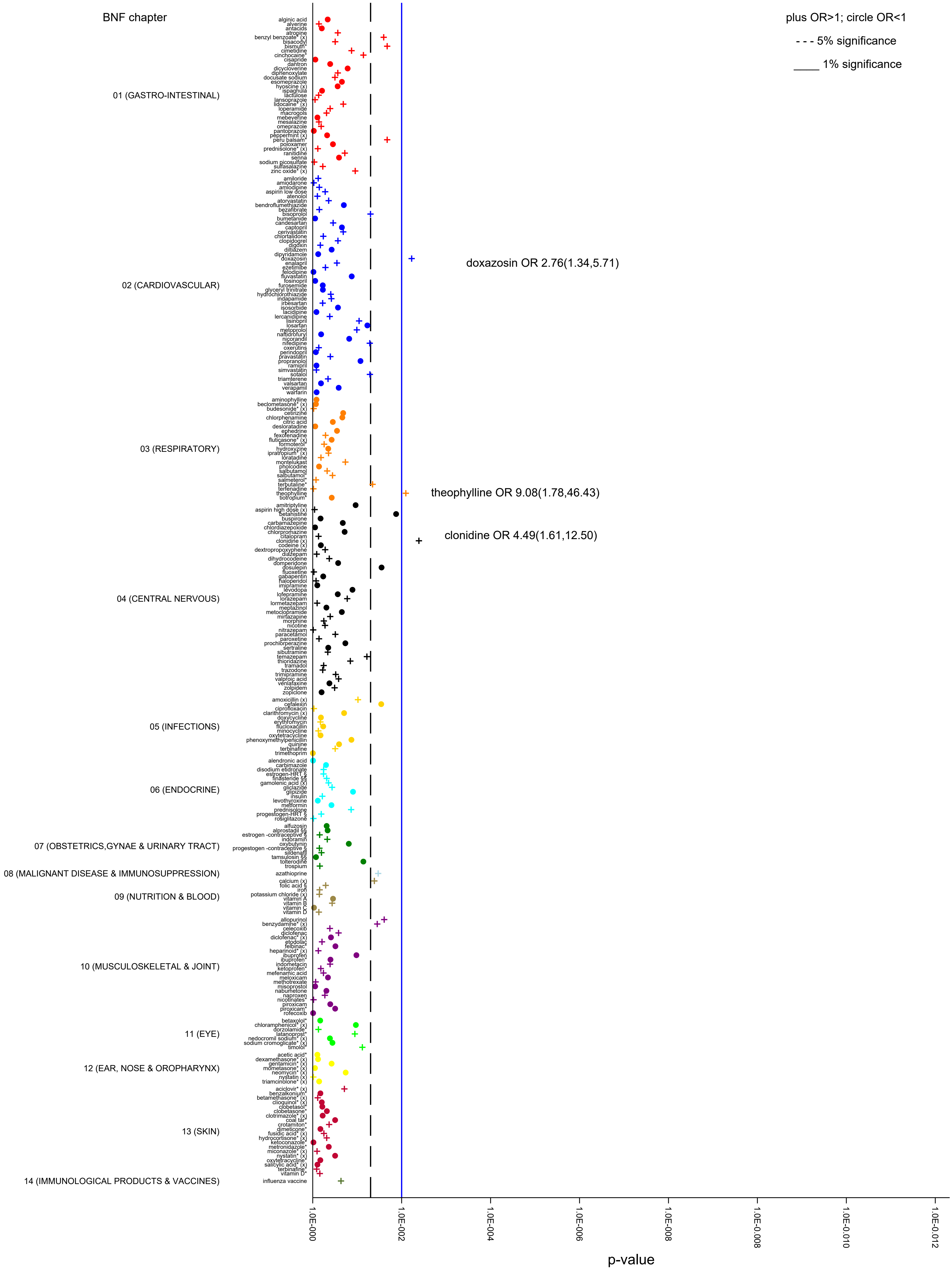

* : local medicine; § females only; §§ males only; (x) most prevalent chapter
